# Exo1 protects DNA nicks from ligation to promote crossover formation during meiosis

**DOI:** 10.1371/journal.pbio.3002085

**Published:** 2023-04-20

**Authors:** Michael Gioia, Lisette Payero, Sagar Salim, Ghanim Fajish V., Amamah F. Farnaz, Gianno Pannafino, Jun Jie Chen, V. P. Ajith, Sherikat Momoh, Michelle Scotland, Vandana Raghavan, Carol M. Manhart, Akira Shinohara, K. T. Nishant, Eric Alani

**Affiliations:** 1 Department of Molecular Biology and Genetics, Cornell University, Ithaca, New York, United States of America; 2 School of Biology, Indian Institute of Science Education and Research Thiruvananthapuram, Trivandrum, India; 3 Institute for Protein Research, Osaka University, Suita, Osaka, Japan; 4 Department of Chemistry, Temple University, Philadelphia, Pennsylvania, United States of America; 5 Center for High-Performance Computing, Indian Institute of Science Education and Research Thiruvananthapuram, Trivandrum, India; Memorial Sloan-Kettering Cancer Center, UNITED STATES

## Abstract

In most sexually reproducing organisms crossing over between chromosome homologs during meiosis is essential to produce haploid gametes. Most crossovers that form in meiosis in budding yeast result from the biased resolution of double Holliday junction (dHJ) intermediates. This dHJ resolution step involves the actions of Rad2/XPG family nuclease Exo1 and the Mlh1-Mlh3 mismatch repair endonuclease. Here, we provide genetic evidence in baker’s yeast that Exo1 promotes meiotic crossing over by protecting DNA nicks from ligation. We found that structural elements in Exo1 that interact with DNA, such as those required for the bending of DNA during nick/flap recognition, are critical for its role in crossing over. Consistent with these observations, meiotic expression of the Rad2/XPG family member Rad27 partially rescued the crossover defect in *exo1* null mutants, and meiotic overexpression of Cdc9 ligase reduced the crossover levels of *exo1* DNA-binding mutants to levels that approached the *exo1* null. In addition, our work identified a role for Exo1 in crossover interference. Together, these studies provide experimental evidence for Exo1-protected nicks being critical for the formation of meiotic crossovers and their distribution.

## Abstract 2

Crossovers between homologous chromosomes in meiosis result from biased resolution of Holliday junction intermediates, but can DNA nicks present in Holliday junctions provide the appropriate information? This study presents genetic evidence that the nuclease Exo1 promotes meiotic crossing over by protecting DNA nicks from ligation.

## Introduction

In most eukaryotes, including budding yeast and humans, the accurate segregation of homologous chromosomes during the first reductional division (Meiosis I) requires the formation of crossovers between homologs. Physical linkages created by crossovers and sister chromosome cohesions distal to the crossover site are critical for proper segregation of chromosome pairs during Meiosis I [1–3]. The inability to establish these physical connections can lead to improper chromosome segregation and aneuploidy, and in humans is thought to be an important cause of birth defects and miscarriages [2,4,5].

**Fig 1:**
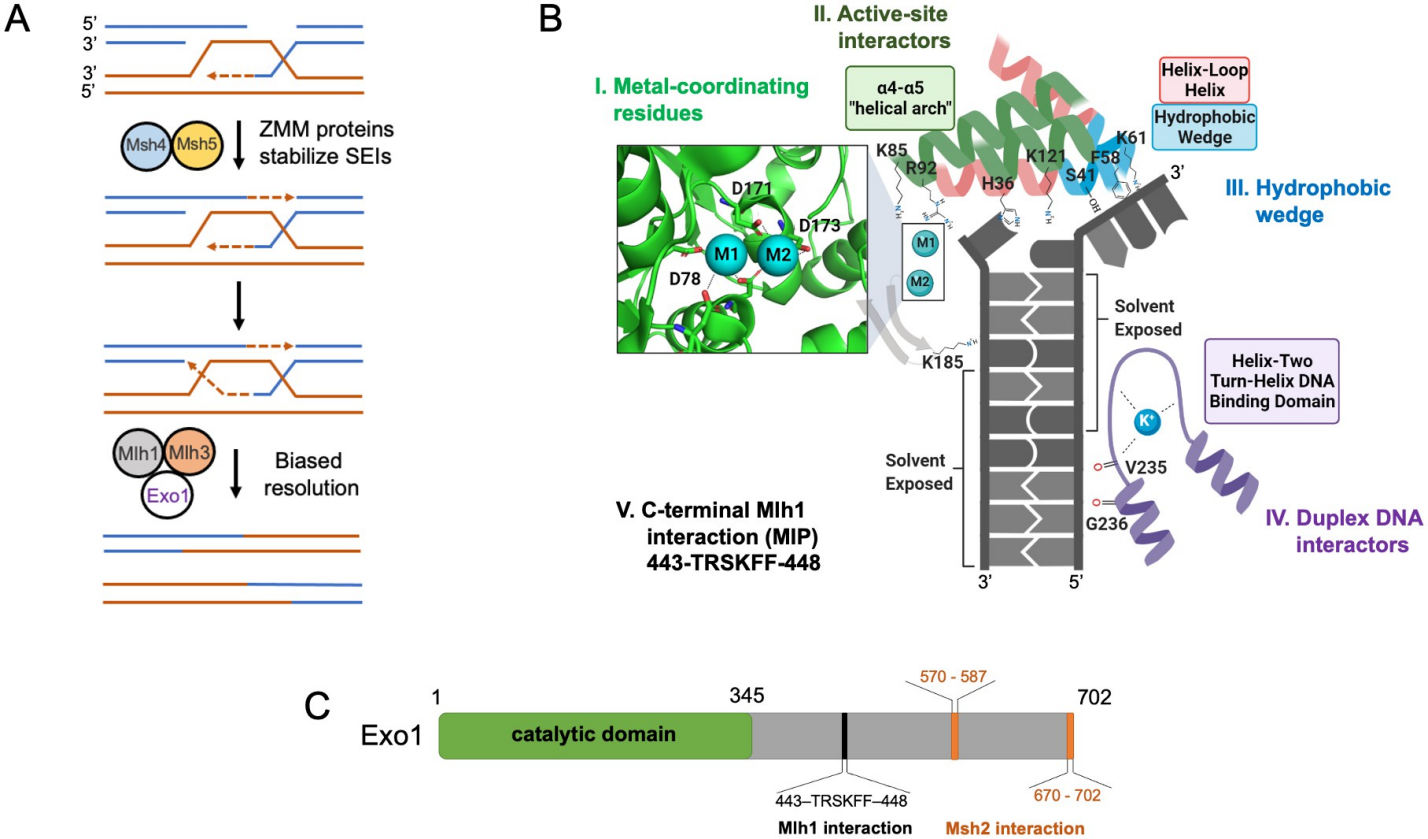
Metal binding, active site interactions, and DNA contact sites of human EXO1 based on the crystal structure of the EXO1-5′ recessed DNA complex. (A) Canonical model showing roles for Msh4-Msh5, Mlh1-Mlh3, and Exo1 in meiotic crossover resolution. See text for details. (B) Close-up of the EXO1 active site using crystal structure PDB #3QEA [26]. We highlight the following residues (positions indicated for *S*. *cerevisiae* Exo1) that were mutated in this study (S1 Fig): Group I; acidic residues (D78, D171, D173) that coordinate the 2 metal ions. Group II; residues that are part of the α4-α5 helical arch involved in fraying (H36, K85, K121) and coordinating the scissile bond adjacent to the catalytic metals that interact with the active site (R92). Group III; S41, F58, K61, that are part of a hydrophobic wedge which induces the sharp bend in DNA at the site of a nick. Group IV; residues that interact with duplex DNA (K185, G236). Group V; residues (F447, F448) in a region of yeast Exo1 that interact with Mlh1. The *exo1-F447A*,*F448A* allele is abbreviated in the text as *exo1-MIP*. Image was created with BioRender.com. (C) Outline of the 702 amino acid *S*. *cerevisiae* Exo1 protein. The N-terminal catalytic domain aligned with the N-terminal human EXO1 catalytic domain (amino acids 1–352; [26]) is presented, as well as a motif critical for interaction with Mlh1 [27], and 2 redundant motifs important for interactions with the mismatch repair factor Msh2 [28]. Gray color represents the unstructured C-terminal tail of Exo1.

In baker’s yeast crossover formation in meiotic prophase is initiated through the genome-wide formation of approximately 150 to 200 Spo11-induced double-strand breaks (DSBs; [6,7]). These breaks are resected in a 5′ to 3′ direction to form 3′ single-stranded tails [8,9]. Strand exchange proteins coat the single-stranded tails and promote their invasion into homologous sequences in the unbroken homolog [2]. In the major Class I crossover pathway, a D-loop intermediate is stabilized by ZMM proteins including Zip2-Zip4-Spo16 and Msh4-Msh5 to form a single-end invasion intermediate (SEI; Fig 1A; [10–15]). This recombination intermediate forms concomitantly with the synaptonemal complex, a structure that is thought to remove chromosomal tangles and interlocks during the homology search process [9,16,17]. DNA synthesis from the SEI, followed by second-end capture, results in the formation of the double Holliday junction (dHJ) intermediate. The dHJ is thought to be stabilized by Msh4-Msh5 and is resolved in a biased orientation to form approximately 90 crossovers (COs) in the yeast genome that are distributed so that they are evenly spaced, with every homolog pair receiving at least 1 crossover (Fig 1A; [12,18–25]).

How are dHJs resolved into crossovers in the Class I pathway? The DNA mismatch repair (MMR) endonuclease Mlh1-Mlh3 and the XPG/Rad2 family 5′ to 3′ exonuclease Exo1 act in meiotic crossover resolution, with *mlh3Δ* and *exo1Δ* single and double mutant strains displaying similar defects in crossing over [25,29,30]. Biochemical analyses of Mlh1-Mlh3 indicated that its endonuclease activity is required for its role in crossover formation, but not as a structure-specific nuclease that symmetrically cleaves Holliday junctions [31–34]. Exo1 acts in many steps in DNA metabolism including in DNA replication, telomere maintenance, homologous recombination, and DNA mismatch repair. It displays a 5′ to 3′ exonuclease activity that results in the formation of 3′ single-stranded ends and a 5′ flap endonuclease activity. Exo1 contains an N-terminal Rad2/XPG nuclease domain that is conserved in Rad2/XPG family members (Fig 1B and 1C; [35–37]). In meiosis *exo1Δ* strains display a defect in the 5′ to 3′ resection of Spo11-induced DSBs (from an average of 800 nt resected in *wild-type* to 270 nt in *exo1Δ*) and a meiotic crossover defect. Despite such defects, *exo1Δ* mutants display wild-type timing and levels of meiotic recombination intermediates, including dHJs [30]. Curiously, an *exo1* mutation (*D173A*) that disrupts a metal-binding site critical for nuclease function was shown to have only a minimal impact on meiotic crossing over despite conferring a defect in 5′ to 3′ resection that was similar to *exo1Δ* [30]. In fact, *Exo1*^*D173A/D173A*^ mice are fertile, whereas *Exo1*^*-/-*^ are sterile [38]. Lastly, genetic analysis showed that disruption of a conserved Mlh1-Interaction Protein sequence (MIP box; Fig 1C) in the Exo1 C-terminal domain conferred intermediate defects in meiotic crossing over, suggesting that Exo1 promotes meiotic crossovers through interactions with Mlh1 and possibly other factors [30,35,39,40]. Together, these analyses suggested that Exo1’s interactions with Mlh1-Mlh3, but not its nuclease function, are critical for crossover formation [30,41,42].

The above observations have provided hints on how Mlh1-Mlh3 and Exo1 could act to resolve dHJs in a biased manner to form crossovers. Additional information was obtained from whole-genome sequencing of spore clones obtained by sporulating diploid yeast created by mating haploid yeast strains containing high levels of sequence divergence. By analyzing the sequence of heteroduplex DNA tracts in the spore clones, Marsolier-Kergoat and colleagues [43] and Martini and colleagues [44] inferred a model in which meiotic crossover resolution is biased towards DNA synthesis tracts. In this model, nicks maintained at the ends of synthesis tracts could direct biased and asymmetric cleavage of the dHJ by recruiting a nick-binding protein that acts in the resolution mechanism. Analysis of recombination events in the mouse has also led to a model that nicked dHJs are precursors to meiotic crossovers [45]. These studies and recent biochemical studies have led to proposals that Mlh1-Mlh3 interact with Exo1, Msh4-Msh5, and the DNA polymerase processivity factor PCNA to resolve dHJs in a biased fashion to form crossovers [46–48]. A component of some of these proposals is that DNA signals present in dHJ intermediates are critical for such resolution. Here, we provide genetic evidence that Exo1 acts to protect DNA from being ligated in recombination intermediates during the formation of crossover products. We also show that it acts to ensure that meiotic crossovers are widely spaced for proper chromosome segregation in the Meiosis I division. These observations provide evidence for dynamic and distinct roles for Exo1 in crossover placement and for maintaining a nicked recombination intermediate for the resolution of dHJs into crossovers.

## Results

### Identifying residues in Exo1 important for facilitating meiotic crossing over

We took advantage of previous biochemical and structural analyses of human and yeast EXO1 family proteins (Exo1, Fen1/Rad27, XPG/Rad2; S1 Table) to examine domains of Exo1 for roles in meiotic crossing over. We focused primarily on the crystal structure of human EXO1 with 5′ recessed DNA (PDB #3QE9) that identified 2 metals in the catalytic site of the Exo1-DNA structure, with residue D171 assisting D173 in coordinating 1 metal, and residue D78 coordinating the other, to hydrolyze the phosphodiester backbone of DNA (**Group I**; Fig 1B, S1 and S2 Figs; [26,49–52]). The crystal structure also identified residues in Exo1 that interact with and position DNA in an orientation to be cleaved [26]. For example, residues H36, K85, R92, and K121 (**Group II**) contribute to the fraying of the duplex DNA bases away from its complement and reside within an α4-α5 helical arch microdomain that forms part of the Exo1 active site (Fig 1B and S1 Fig). Furthermore, the structure showed that Exo1 makes key contacts with DNA through several domains (Fig 1B). A crucial component of Rad2/XPG members is the hydrophobic wedge (Fig 1B, **Group III**), a structurally conserved domain that induces a sharp bend at a double strand-single strand DNA junction and gives the enzyme family its specificity for gapped/nicked DNA substrates [26,53]. Several residues within the wedge motif form hydrophobic interactions with the non-substrate strand, as well as 2 lysine residues that appear to coordinate this portion of the non-substrate strand through hydrogen bonding (Fig 1B and S1 Fig). In addition, several conserved residues (K185, G236, **Group IV**) stabilize an interaction with Exo1 and the pre-nick duplex DNA (Fig 1 and S1 Fig). G236 is a conserved residue present in a helix-two turn-helix motif that hydrogen bonds with duplex DNA away from the active site and is presumed to facilitate exonuclease processivity as the protein moves along the DNA backbone [26,54]. K185 is part of a small hairpin loop that directly contacts the DNA backbone of the substrate strand away from the active site and is thought to be critical for recognition of duplex DNA [26,55].

In addition to the structural work outlined above, our work was guided by mutational studies of human and yeast EXO1 family proteins that showed that Groups I, II, and III mutant proteins displayed strong and often severe defects in exo- and endonuclease activity (S1 Table). For example, mutations of Group I or II residues (H36A, K85A, R92A, D173A) conferred strong defects in Exo1 nuclease activity [26]. A Group IV mutation, *exo1-K185A*, was shown in baker’s yeast to confer elevated sensitivity to DNA-damaging agents, and the mutant protein showed reduced exonuclease activity [55]. A second Group IV mutation, *exo1-G236D*, conferred defects in Exo1-dependent DNA mismatch repair in baker’s yeast [39].

### Mutations in metal coordinating and active site residues in Exo1 do not disrupt meiotic crossing over

We tested mutations in Groups I to IV residues for their effect on meiotic crossing over at the *CEN8-THR1* interval located on chromosome VIII using a spore-autonomous fluorescence assay (Fig 3A; approximately 39% single crossovers in *wild-type*, 20% in *exo1Δ*; [56]) and by tetrad analysis at 4 consecutive intervals on Chromosome XV (Fig 4A; 104.9 cM map distance in *wild-type*, 54.7 cM in *exo1Δ*; [57]). The *exo1Δ*, *mlh3Δ*, and *msh5Δ* mutations conferred defects in crossing over at these 2 chromosomal regions that were similar to previous studies (Fig 4B; [25,31,57,58]). In addition, we saw a large decrease in crossing over in *exo1Δ mus81Δ* (approximately 12-fold) that was similar to *mlh3Δ mus81Δ* [57], confirming the epistatic interaction between *exo1Δ* and *mlh3Δ*. The relatively high spore viability (46%, S2 Fig) seen in *exo1Δ mus81Δ* is consistent with the viability seen for other mutants (*mlh1Δ mms4Δ*, *mlh3Δ mms4Δ;* [57,59]) in which both type I and type II crossover pathways were disrupted. Detailed explanations for the high spore viability seen in these mutants despite their significant defects in crossing over can be found in Sonntag Brown and colleagues [59].

**Fig 2:**
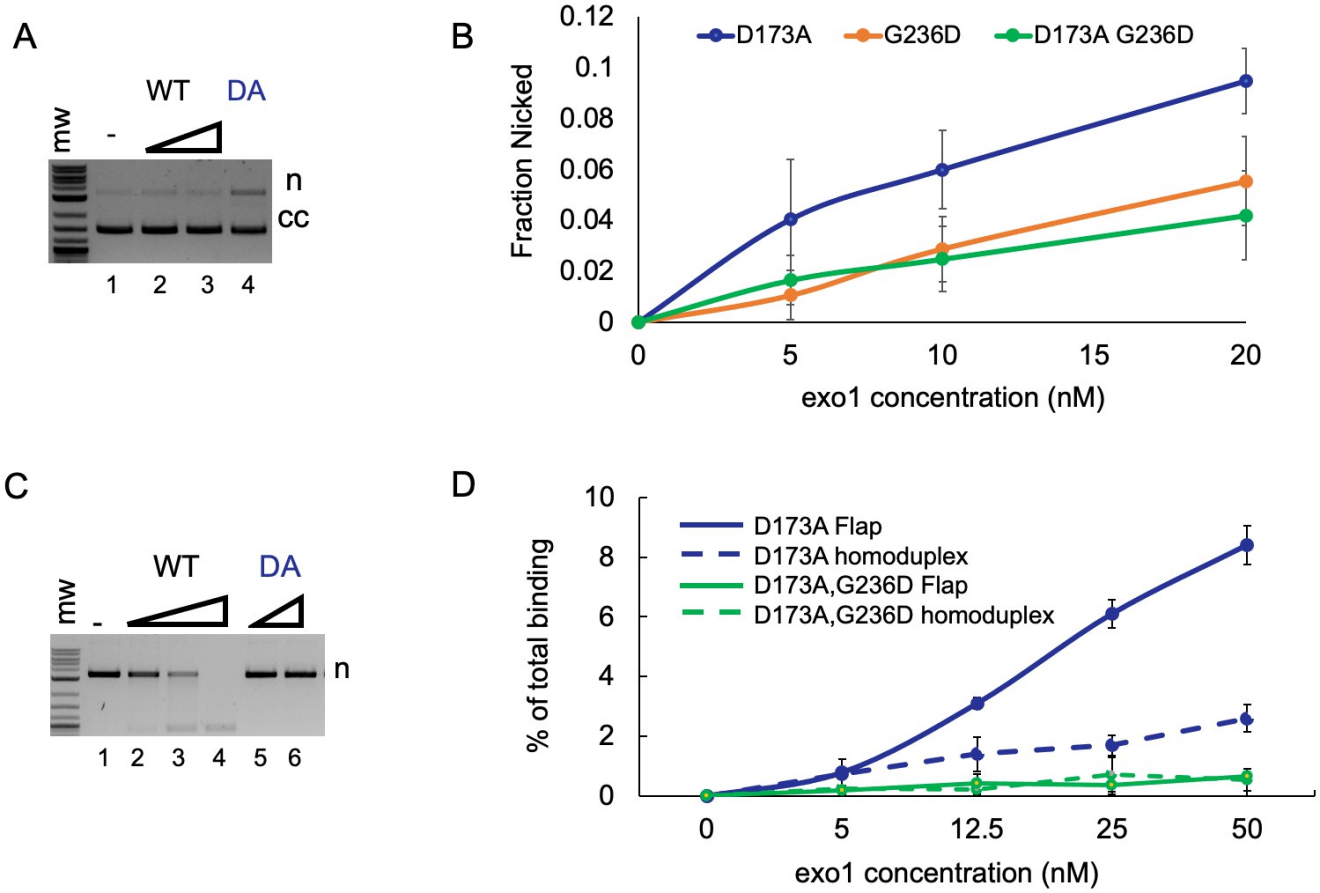
Analysis of exo1-D173A nuclease activity. (A) Endonuclease activity of Exo1 (WT) and exo1-D173A (DA; Materials and methods) on a 2.7 kb supercoiled pUC18 plasmid. Exo1 is present at 1 nM and 10 nM in lanes 2 and 3, respectively, and exo1-D173A is present at 20 nM in lane 4. It is unlikely that Exo1 nicked the closed circular substrate and then degraded it through its exonuclease activity because we saw no measurable loss of closed circular DNA (compare lanes 2 and 3 to lane 1). (B) Titration of exo1-D173A, exo1-G236D, and exo1-D173A G236D endonuclease activities on supercoiled (cc) pUC18 plasmid. Error bars represent +/- standard deviation of 4 repetitions. (C) Exonuclease activity of Exo1 (WT) and exo1-D173A (DA; Materials and methods) on a 2.7 kb pUC18 plasmid with 4 preexisting nicks. DNA products were resolved by native agarose gel. Exo1 is present at 6 nM, 12 nM, and 24 nM in lanes 2–4, and exo1-D173A is present at 20 nM and 40 nM in lanes 5–6. (D) Binding of exo1-D173A and exo1-D173A G236D to 5′ flap and homoduplex oligonucleotide DNA substrates. DNA substrates and filter binding conditions are described in the Materials and methods. Titrations were performed in 60 μl reactions containing 15 nM 5′ flap or homoduplex DNA substrate, 35 mM NaCl, 20 mM Tris 7.5, 0.04 mg/ml BSA, 0.01 mM EDTA, and 0.1 mM DTT, and the indicated concentrations of exo1. Error bars, standard deviation of 3 repetitions. Underlying data can be found in S1 Data.

**Fig 3:**
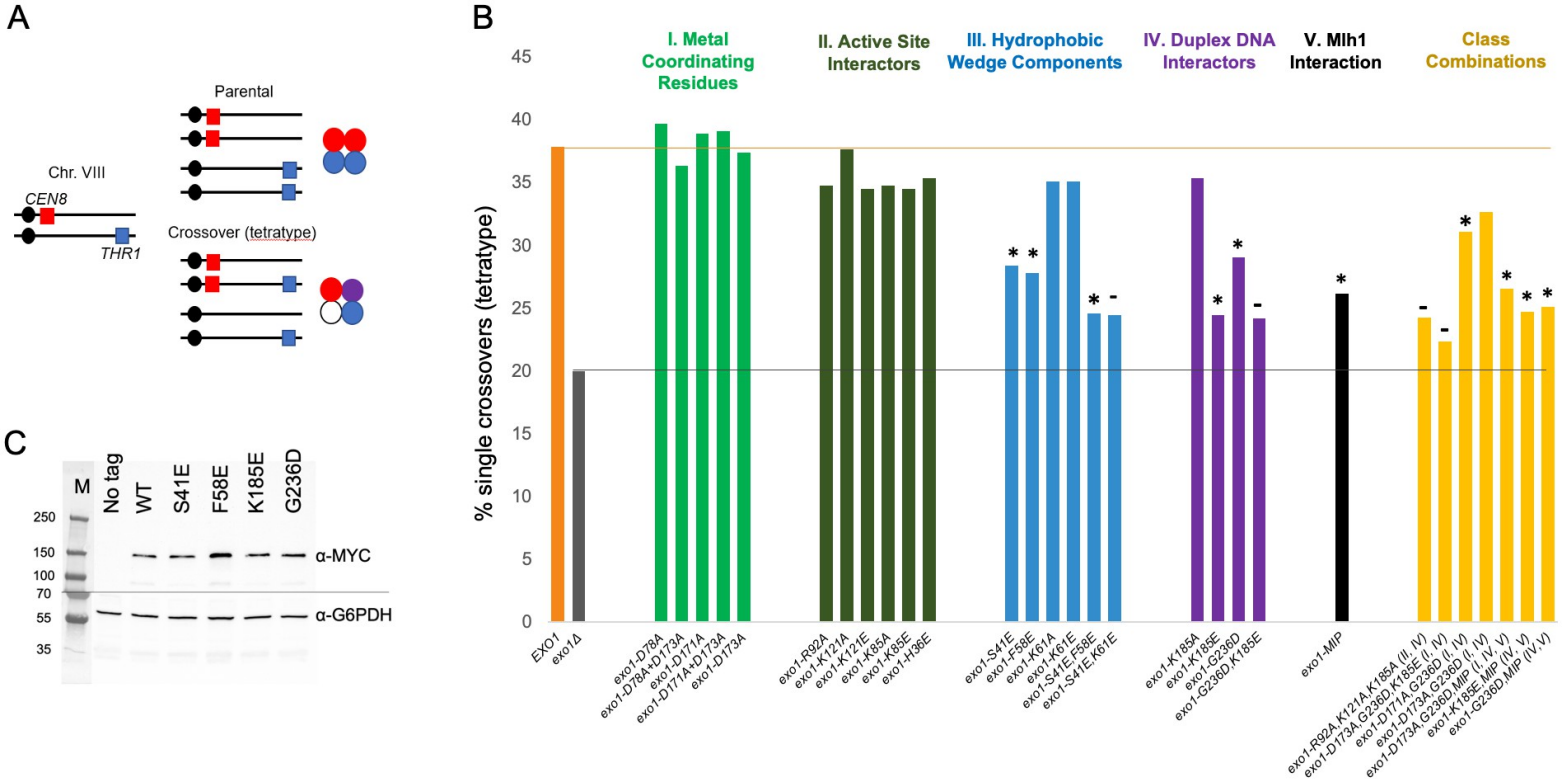
Crossing over for the indicated *exo1* strains was measured in the 20 cM *CEN8* to *THR1* interval on Chr. XV using a spore-autonomous fluorescence assay. (A) The spore-autonomous fluorescence assay was used to measure single meiotic crossover events (tetratypes) in the chromosome VIII *CEN8-THR1* interval [56]. (B) Single meiotic crossover events in the indicated strains. Mutations are separated into categories based on disruption of specified functions outlined in Fig 1B. *EXO1* and *exo1Δ* levels are indicated by green and red dashed lines, respectively, and *, statistically distinguishable from *EXO1* and *exo1Δ;* -, distinguishable from *EXO1*, but indistinguishable from *exo1Δ*. Underlying data for Panel B can be found in S2A Table and S2 Data. (C) Detection by western blot of Exo1-13MYC (WT) and the indicated mutant variants from mid-log growing yeast cultures. G6PDH is provided as a loading control. The sizes of molecular weight standards (M) are indicated. See Materials and methods for details. Underlying data for Panel C can be found in S3 Data.

**Fig 4:**
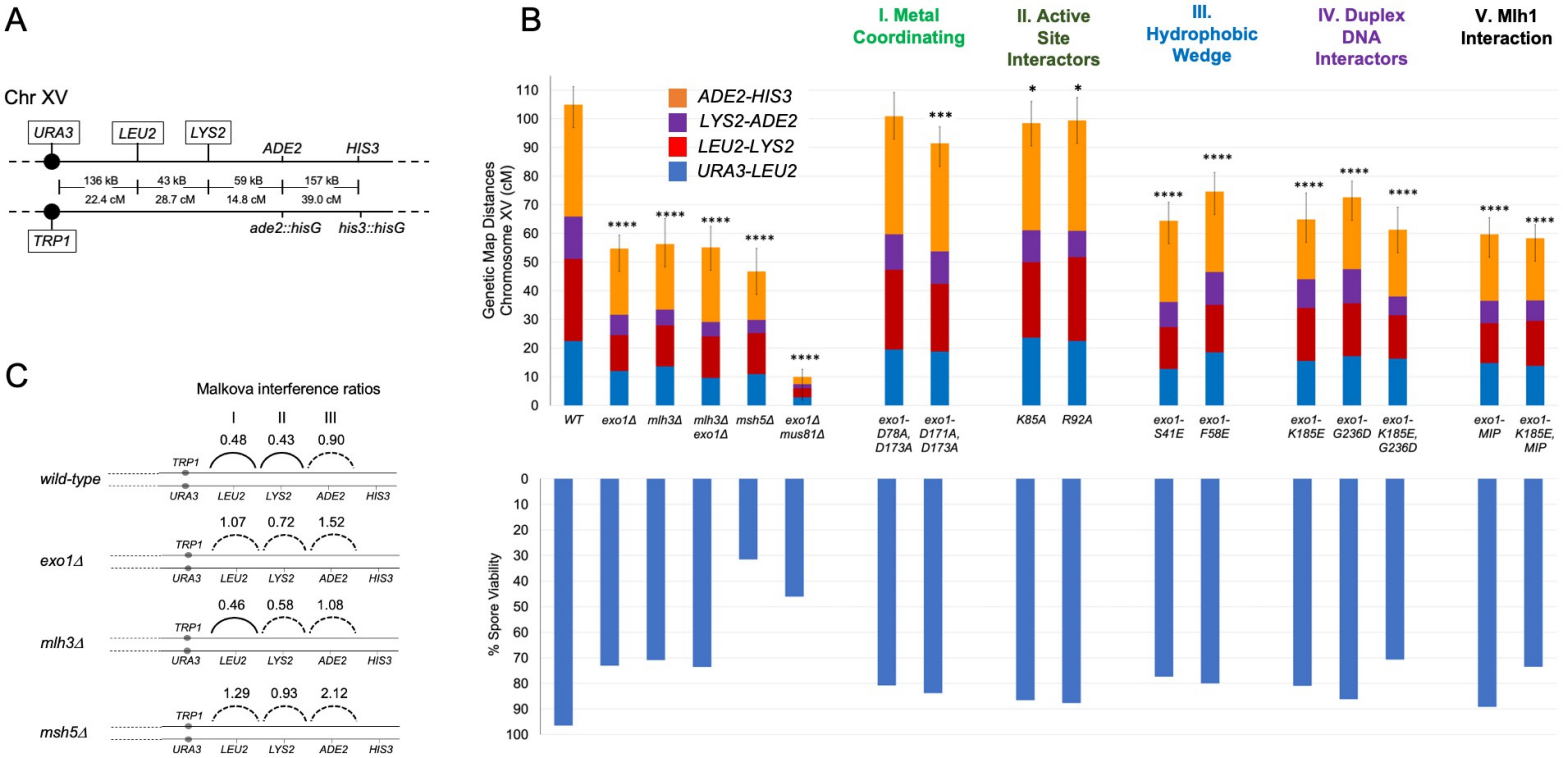
Meiotic crossover phenotypes in *exo1* mutant strains. (A) Genetic markers on chromosome XV spanning the *CENXV-HIS3* interval in the EAY1108/1112 strain background [57]. The solid circle indicates the centromere. Distances between markers in KB and cM are shown for *wild-type*. Markers that are boxed indicate insertions at chromosome XV. (B) Cumulative genetic distance (cM) in *wild-type* (*WT*) and *exo1* strains. Genetic map distances for the *URA3-HIS3* interval of chromosome XV in *wild-type* and the indicated mutant strains. Each bar is divided into sectors corresponding to genetic intervals in the *URA3-HIS3*, as measured from tetrads (T). The spore viability data obtained from tetrad analysis are shown, with the complete data set presented in S2 Fig. The asterisks indicate the number of genetic intervals (0–4) that are distinguishable from *wild-type* in the indicated genotypes as measured using standard error calculated by Stahl Laboratory Online Tools. Standard error was calculated for each interval using Stahl Online Tools. Error bars represent the cumulative standard error across all 4 intervals (https://elizabethhousworth.com/StahlLabOnlineTools/; S3 Table). Underlying data for Panel B can be found in S3 Table. (C) Interference analysis for pairs of adjacent genetic intervals on Chromosome XV in the EAY1108/EAY1102 strain background. Crossover interference was analyzed on Chromosome XV by measuring centimorgan (cM) distances in the presence and absence of a neighboring crossover [60,61]. Malkova interference is presented as a ratio of cM for crossovers present/cM for crossovers absent, with interval pairs I–III shown. Dashes indicate no detectable positive interference. Significance of differences in tetrad distribution was assessed using a G test. Statistically significant *p* values (*p* < 0.05) suggest the presence of interference in the genetic interval. Underlying data for Panel C can be found in S4A and S4B Table.

We made Group I mutations (D78A, D171A, and D173A; Fig 1B) individually or in combination to disrupt the coordination of the 2 metals in the Exo1 active site. Our work was motivated by the finding that exo1-D173A, containing a mutation in 1 metal-binding site, displayed a weak DNA nicking activity on closed circular 2.7 kb DNA substrate that was similar to the nicking activity seen for Mlh1-Mlh3. Such an activity was not detected for wild-type Exo1 (Fig 2A and 2B; approximately 10% nicking of pUC18 at 20 nM exo1-D173A compared to approximately 20% nicking at 20 nM Mlh1-Mlh3 [34]). However, disruption of either one or both metal-binding sites of Exo1 had minor if any effect on meiotic crossing over at the *CEN8-THR1* interval and at the 4 intervals on Chromosome XV, providing more conclusive evidence that Exo1 nuclease activity does not disrupt meiotic crossing over (Figs 3B and 4B; S2 and S3 Tables). We then mutated Group II residues in Exo1 that interact with and position DNA in an orientation to be cleaved [26]. As shown in Figs 3B and 4B (see also S2 and S3 Tables), the *exo1-H36E*, *exo1-K85A/E*, *exo1-R92A*, and *exo1-K121A/E* mutations (Group II) conferred very modest, if any effect on meiotic crossing over compared to *wild-type*, suggesting that coordination of the scissile bond for catalysis within the active site is also not critical for crossing over. Consistent with these observations, the dramatic loss of nuclease activity seen with human EXO1 bearing K85A, R92A, or K185A mutations ([26,55]; S1 Table) further supports the dispensability of Exo1 catalytic activity for meiotic crossing over.

### Mutations in DNA-binding domains of Exo1 confer defects in meiotic crossing over

Mutations in Exo1 residues that contact DNA, *exo1-K185E* and *exo1-G236D*, conferred significant decreases in crossover formation (71.3 cM, 29.1% tetratype in *exo1-G236D* and 64.9 cM, 24.5% tetratype in *exo1-K185E*) in the *URA3-HIS3* and *CEN8-THR1* intervals, respectively (Figs 3B and 4B; S2 and S3 Tables). Interestingly, the exo1-G236D protein displayed a very weak endonuclease activity, consistent with a DNA binding defect (Fig 2B). To test this hypothesis, we measured the binding of the *exo1-G236D* mutation in the context of the *exo1-D173A* mutation to 5′ flap and homoduplex oligonucleotide DNA substrates (Materials and methods). We performed these experiments in the *exo1-D173A* catalytic mutation background because of concerns that wild-type Exo1 would degrade oligonucleotide substrates (Fig 2C). Previous studies on human exo1-D173A showed that it bound to a 5′ flap substrate with a binding affinity similar to human EXO1 [62]. Yeast exo1-D173A displayed specificity for the 5′ flap substrate but exo1-D173A,G236D displayed very weak binding to both 5′ flap and homoduplex substrates, indicating a DNA-binding defect (Fig 2D). The hydrophobic wedge mutations *exo1-S41E* (64.4 cM, 28.4% tetratype) and *exo1-F58E* (74.6 cM, 27.8% tetratype) also conferred crossover defects, with double mutation combinations conferring more severe phenotypes (*exo1-K185E*,*G236D-*24.2% tetratype; *exo1-S41E*,*F58E-*24.6% tetratype).

We made alleles that combined Groups I to V mutations (Fig 3B; S2 Table). These mutations conferred a variety of phenotypes. For example, *exo1-R92A*,*K121A*,*K185A* (24.3% tetratype) and *exo1-D173A*,*K185E*,*G236D* (22.4% tetratype) triple mutations conferred phenotypes more similar to *exo1Δ* (20.0% tetratype) than to single group mutations. In contrast, we observed an increase in crossover frequency in an *exo1-G236D* mutant Group IV when the *D173A* Group I mutation was present (*exo1-G236D/exo1Δ*-29.1% tetratype versus *exo1-D173A*,*G236D/exo1Δ-*32.7% tetratype; *exo1-G236D/exo1-G236D*-29.9% tetratype versus *exo1-D173A*,*G236D/exo1-D173A*,*G236D*-35.7% tetratype). Lastly, single and double mutations in DNA binding and Mlh1 interaction (*exo1-G236D*, *exo1-K185E*, *exo1-MIP*, *exo1-G236D*,*MIP*, *exo1-K185E*,*MIP)* conferred very similar tetratype values (Fig 3 and S2 Table), consistent with Exo1 DNA binding and Mlh1 interactions being involved in the same functional step. Additional studies will be needed to better understand how these multiple mutations impact Exo1 function (see Discussion).

The protein stability of Exo1 was analyzed in exponentially growing vegetative cultures, focusing on Groups III and IV mutations that conferred crossover defects (*exo1-S41E*, *-F58E*, *-K185E*, *-G236D)*. We tagged the mutant alleles with 13 repeats of the MYC epitope (*MYC*_*13*_ *tag*; [63,64]) and found that strains containing the *EXO1-13MYC* allele complemented Exo1 crossover functions in the spore autonomous crossover assay (S2 Table). As shown in Fig 3C, strains bearing the Groups III and IV constructs displayed full-length Exo1 protein at levels similar to wild type, suggesting that these mutations did not disrupt Exo1 protein stability.

### exo1 strains displayed separable meiotic crossover and DNA repair defects

*exo1null* homozygous strains induced to enter meiosis showed spore viability patterns (73% spore viability; 4, 2, 0 viable tetrads > 3, 1) consistent with a Meiosis I non-disjunction phenotype (Fig 4B and S2 Fig; [41,65]). Curiously, this pattern linking defects in meiotic crossing over with meiosis I non-disjunction was not seen in strains containing *exo1* point mutations. Additionally, *exo1* mutants (*exo1-K185E*, *exo1-K185E*,*G236D*, *exo1-MIP*) that showed strong meiotic crossover defects relative to the *exo1* null displayed spore viabilities that ranged from 71% to 89%. A possible explanation for these differences is that Exo1 has multiple roles in meiosis that affect spore viability, some of which are unrelated to its pro-crossover function. In support of this hypothesis, some of the *exo1* mutations analyzed above conferred defects in DNA repair, as measured by sensitivity to methyl-methane sulfonate (MMS; S3 Fig) but conferred functional meiotic crossing over. For example, the *exo1-D78A*, *exo1-D171A*, and *exo1-D173A* catalytic mutants were sensitive to MMS but were nearly wild type for meiotic crossing over. Disparities between DNA repair and crossover phenotypes were also seen for the active site mutations *exo1-K85E* and *exo1-K121A*, the DNA-binding mutant *exo1-K185E* and the MLH interacting mutant *exo1-MIP*. These observations provide evidence that Exo1 has multiple cellular functions, with a subset acting in meiotic crossing over (see interference analysis below).

### Expression of RAD27 in meiosis partially complements the exo1null crossover defect

Based on our structure-function analysis of Exo1, we hypothesized that a protein that mimicked the DNA-binding specificity of Exo1 might complement its functions in meiotic crossing. The Rad2 family of nucleases consists of 4 evolutionarily conserved members: *RAD2/XPG* in yeast/humans respectively, *EXO1/EXO1*, *RAD27/FEN1*, and *YEN1/GEN1* [66–68]. In yeast, *RAD27* shares the highest sequence similarly with *EXO1*, and previous studies have shown that *EXO1* can complement some *RAD27* functions [69–71]. The *S*. *cerevisiae* Rad27 protein (382 amino acids in length) contains a 339 amino acid N-terminal catalytic domain homologous to the EXO1 catalytic domain (Fig 1C), followed by a C-terminal domain that contains a PCNA interaction motif and an unstructured region [66]. Rad27 is a 5′ flap endonuclease that acts in Okazaki fragment maturation, binds to DNA in a structurally analogous way by inducing a sharp bend in the DNA substrate upon protein binding [26,72,73].

**Fig 5:**
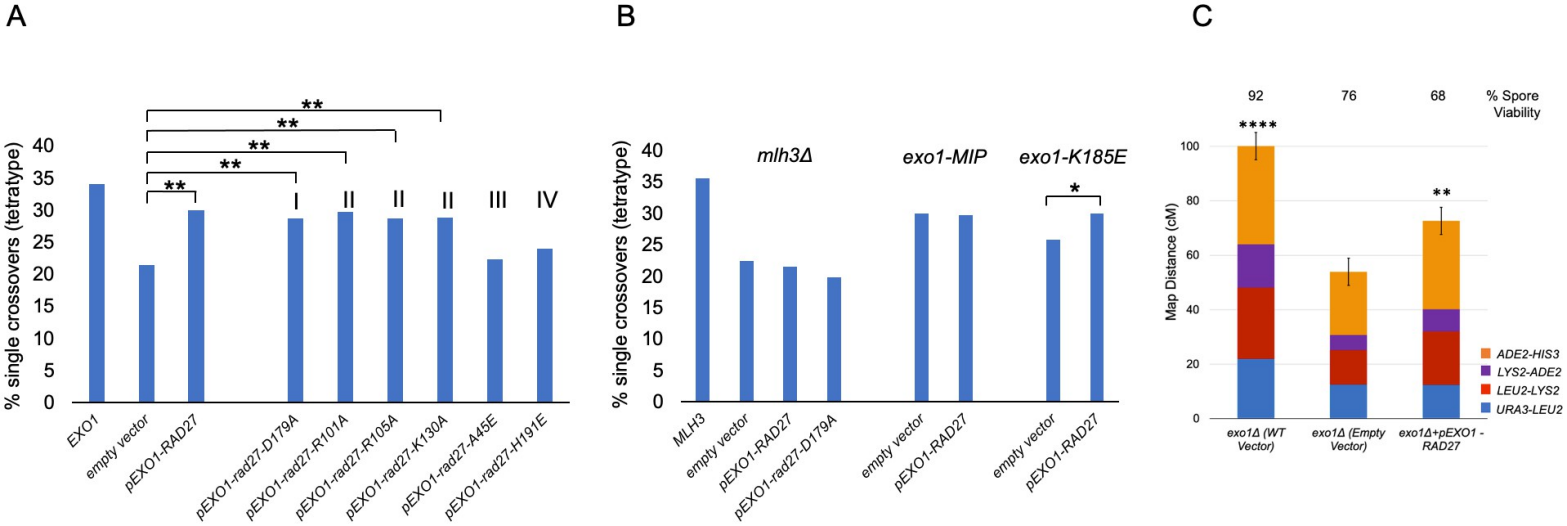
*RAD27* expressed from the *EXO1* promoter can restore crossover functions to *exo1*Δ strains. (A) *pEXO1-EXO1*, *ARS-CEN* (pEAA715), *pEXO1-RAD27*, *ARS-CEN* (pEAA720), the indicated mutant *rad27* derivatives (pEAA724, pEAA727-731), and an empty *ARS-CEN* vector (pRS416), were transformed into an *exo1Δ* strain and examined for crossing over at the *CEN8-THR1* locus. The *rad27* mutations were grouped (I, metal-coordinating; II, active-site; III, hydrophobic wedge; IV, duplex DNA) like those presented for Exo1 (Fig 1B). Significance (**p < 0.01) compared to the *exo1Δ* strain containing an empty vector was determined using a two-tailed Fisher’s Exact Test. (B) *mlh3Δ* and the indicated *exo1* strains were transformed with *pEXO1-RAD27* (pEAA720), *pEXO1-rad27-D179A* (pEAA724), and empty vector (pRS416) and examined for crossing over at the *CEN8-THR1* locus. Significance (*p < 0.05) between the indicated mutant strain containing an empty vector vs. a *RAD27* plasmid was determined using a two-tailed Fisher’s Exact Test. (C) The *pEXO1-RAD27* plasmid pEAI482 was transformed into *exo1Δ* strains (with *pEXO1*, *ARS CEN-*pEAI483, and an empty *ARS-CEN* vector pLZ259 as controls) to measure crossing over in the *URA3-HIS3* interval in the EAY1108/1112 background. Asterisks indicate the number of genetic intervals that are distinguishable from the *exo1Δ* containing the empty vector, as measured using standard error calculated through Stahl Laboratory Online Tools (https://elizabethhousworth.com/StahlLabOnlineTools/; S3 Table). Underlying data for Panels A and B can be found in S2B Table and S2 Data. Underlying data for Panel C can be found in S3 Table.

We tested if Rad27 could complement Exo1 meiotic functions, noting that a *rad27Δ*/*rad27Δ* diploid strain displays wild-type levels of meiotic crossing over as measured in the spore-autonomous fluorescence assay (S2 Table). We did not observe complementation of *exo1Δ* when *RAD27* was expressed through its native promoter. However, significant increases in crossing over were observed when *RAD27* was expressed through the *EXO1* promoter (*pEXO1-RAD27)* on both Chromosomes VIII (from 21.5% to 29.9% tetratype; Fig 5A; S2B Table) and XV (from 53.8 cM to 72.6 cM; Fig 5C; S3 Table). This complementation was likely due to the high level of meiotic expression of Rad27 through the *EXO1* promoter (S4 Fig; [74]). Furthermore, expression of the nuclease dead *pEXO1-rad27-D179A* allele [75,76] conferred similar levels of crossover complementation (Fig 5A; S2B Table). This observation encouraged us to further test our hypothesis by making 5 *rad27* mutations based on previous biochemical and structural characterization of the human homolog of Rad27, FEN1 (S1 Table; [77,78]). As shown in Fig 5A, *rad27-R101A*, *rad27-R105A*, and *rad27-K130A*, which are mutated in a domain that coordinates the scissile bond for catalysis (mutations in homologous positions in FEN1 disrupt flap cleavage; S1 Table), complemented the crossover defect in *exo1Δ*, consistent with the phenotypes exhibited by *exo1* Group II mutations. Interestingly, the *rad27-A45E* and *rad27-H191E* mutations (analogous to Groups III and IV, respectively) did not complement the *exo1Δ* crossover defect, as predicted for mutations that disrupt critical protein–DNA interactions.

We also tested if *RAD27* expression from the *EXO1* promoter could improve meiotic crossover functions of *exo1* strains bearing mutations within (*exo1-K185E*) or outside of the DNA-binding domain (*exo1-MIP*). As shown in Fig 5B, meiotic crossing over in *exo1-K185E*, but not *exo1-MIP*, was increased in cells containing *pEXO1-RAD27*. These observations are consistent with Rad27 being able to substitute for Exo1 DNA-binding functions because improved complementation by *pEXO1-RAD27* was seen in a DNA-binding mutant (*exo1-K185E*), but not in a mutant predicted to be functional for DNA binding but defective in interacting with other crossover factors (*exo1-MIP*). Lastly, we did not observe complementation of meiotic crossing over by *pEXO1-RAD27* in strains lacking functional Mlh1-Mlh3 (*mlh3Δ)*, indicating that Rad27 complementation was specific to Exo1 function and was not bypassing Mlh1-Mlh3-Exo1 dependent dHJ resolution steps.

### Interference analysis suggests a role for Exo1 prior to crossover resolution

Expression of *RAD27* under the *EXO1* promoter (*pEXO1-RAD27* plasmid) could partially complement CO defects in *exo1Δ* strains; however, this expression did not improve the meiotic spore viability or MMS resistance seen in *exo1Δ* strains, suggesting that Exo1-specific functions were likely critical to confer high spore viability (Fig 5C and S2 Fig). We performed crossover interference analysis to determine if *exo1Δ* strains showed defects in addition to those seen in DSB resection and CO resolution. Crossover interference was measured on chromosome XV using both the Malkova and coefficient of coincidence (COC) methods (S4 Table; [60,61,79]). The Malkova method calculates the map distances for a genetic interval in the presence and absence of a crossover in an adjacent interval, whereas COC measures the double crossover rate compared to the expected rate in the absence of interference. As shown in S4A Table, the COC ratios showed similar trends, and for this reason, we focused on the Malkova analysis.

The Malkova method measurements are presented as a ratio, where 0 indicates complete interference and 1 indicates no interference. Three pairs of intervals (*URA3-LEU2-LYS2*, *LEU2-LYS2-ADE2*, and *LYS2-ADE2-HIS3)* were tested for interference. In all 3 interval pairs tested, *exo1Δ* displayed a loss of interference compared to *wild-type*. Most strikingly, 2 interval pairs that displayed strong interference in *wild-type* strains (Malkova ratios of 0.48 at *URA3-LEU2-LYS2* and 0.43 at *LEU2-LYS2-ADE2)* displayed losses of interference in *exo1Δ* (1.07 and 0.72, respectively; Fig 4C). The interference defect seen in *exo1Δ* (all 3 interval pairs showed a lack of interference) was stronger than that seen in the *mlh3Δ* strain (2 intervals showed a lack of interference; also see [80]), suggesting a role for Exo1 in promoting interference independent from its association with Mlh1-Mlh3 in crossover resolution. Furthermore, *exo1Δ* displayed interference defects more similar to the defects seen for *msh4Δ* and *msh5Δ*, where all intervals showed a lack interference ([65,81–83]; Fig 4C). Interestingly, a lack of interference was observed in all 3 interval pairs in the *exo1Δ* strain containing *pEXO1-RAD27* (Malkova ratios of 1.18, 0.94, and 0.89 in interval pairs I, II, III, respectively; S4 Table), supporting the idea that *RAD27* expression in meiosis could partially complement Exo-dependent crossover functions, but not Exo1 functions needed to make evenly spaced and obligate crossovers required for accurate chromosome segregation in Meiosis I and to produce viable spores. Such Exo1 functions are likely to reside in its C-terminal tail, missing in Rad27, which contains Mlh1 and Msh2-interaction motifs (Fig 1C). Together, the data suggest a previously uncharacterized role for Exo1 in establishing crossover interference.

To test if the early resection role of Exo1 [30] could account for Exo1’s role in crossover interference, *exo1-D171A*,*D173A* and *exo1-D78A*,*D173A* catalytic mutants were analyzed for interference defects (S4A and S4B Table). These mutants displayed interference similar to, or stronger than, *wild-type* (see Discussion). In fact, the interference defect observed in *exo1Δ* was not recapitulated in any of the *exo1* alleles tested. These results suggest that Exo1 promotes interference through a mechanism that is distinct from its pro-crossover role.

### Msh5 DNA interactions and foci are not dependent on Exo1

**Fig 6:**
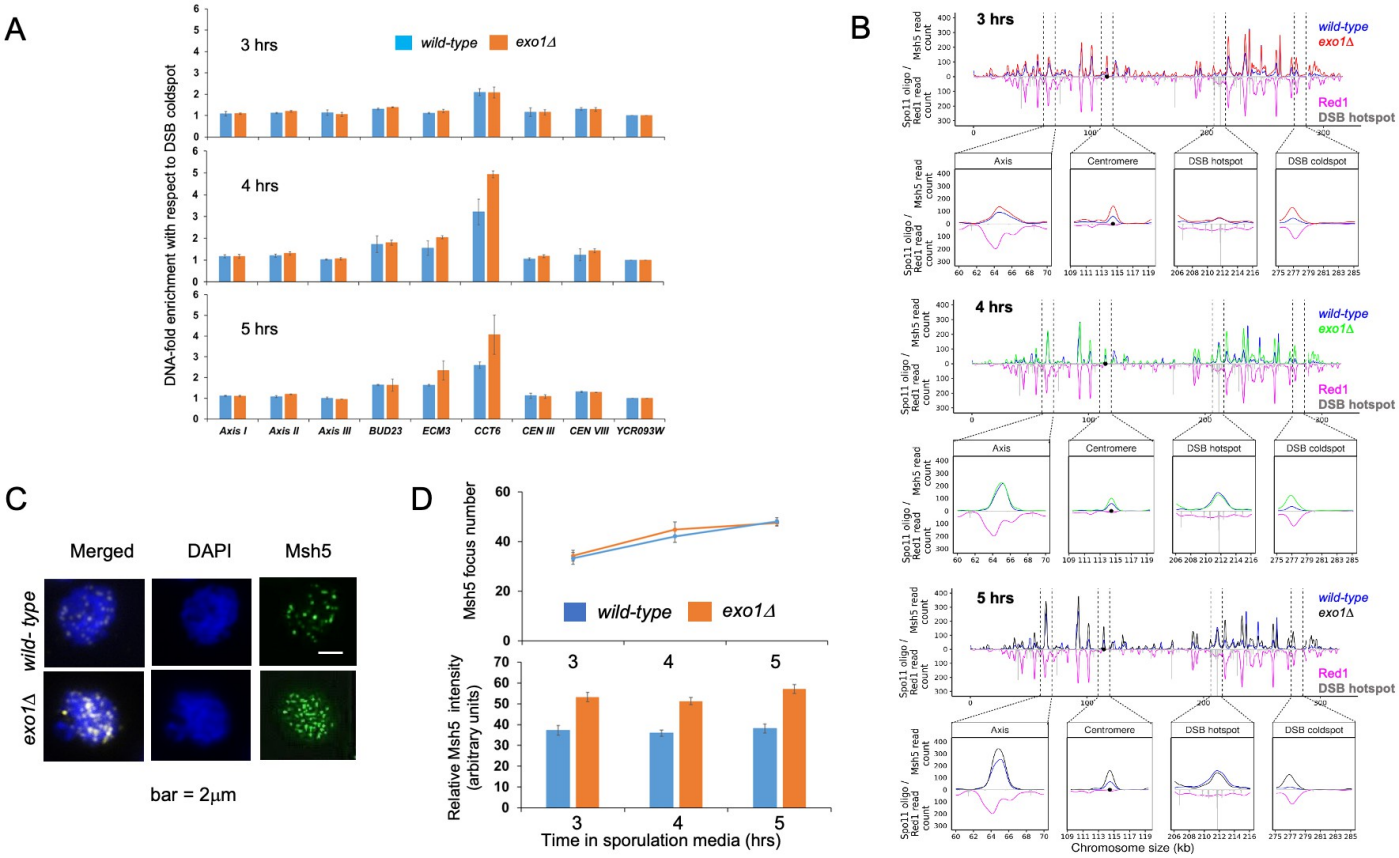
Msh5 localization to chromosomes in *wild-type* and *exo1*Δ strains. (A) ChIP-qPCR analysis of Msh5 binding at DSB hotspots (*BUD23*, *ECM3*, and *CCT6*), centromere regions (*CEN III* and *CEN VIII*), and axis regions (*Axis I*, *Axis II*, and *Axis III*) relative to DSB cold spot (*YCR093W*) in *wild-type* and *exo1Δ* at 3, 4, and 5 h after transfer of cells to sporulation media. Samples are normalized using input and plotted after dividing with the cold spot value. Error bars represent the standard deviation from 2 independent biological replicates. See Nandanan and colleagues [85] for region assignment. Underlying data for Panel A can be found in S4 Data. (B) ChIP-Seq analysis of Msh5 binding in *exo1Δ* mutant. Representative images show normalized Msh5 ChIP-seq reads (NCIS) in *exo1Δ* (2 replicates) on chromosome III at t = 3 h, 4 h, and 5 h after induction of meiosis. Zoomed in images show the centromeric region, axis region, 1 DSB hotspot (*BUD23*), and 1 DSB cold spot (*YCR093W*). Red1 and Spo11 data are from Sun and colleagues [89] and Pan and colleagues [7]. The black circle shows the centromere. Underlying data for Panel B can be found in National Center for Biotechnology Information Sequence Read Archive, accession number PRJNA780068. (C) Representative images of Msh5 staining of chromosome spreads of wild-type and the *exo1Δ* cells at 5-h incubation in sporulation media. Msh5, green; DAPI, blue. Bar indicates 2 μm. (D) Top; number of Msh5 foci was counted in Msh5-focus positive spreads at the indicated times. At each time point, 30 nuclei were counted. Mean +/- standard deviation of 3 independent time courses are shown. (D) Bottom; relative ratio of Msh5 intensity to DAPI intensity was quantified. At each time point, 30 Msh5-positive nuclei were analyzed. Mean+/- standard deviation of 3 independent time courses are shown. Underlying data for Panel D can be found in S5 Data. DSB, double-strand break.

Crossover interference involves the recruitment of ZMM proteins that stabilize and identify a set of dHJs for Class I crossover resolution. Among this class of factors is Msh4-Msh5, which stabilizes SEIs after strand invasion [12]. Interference and crossover formation are significantly reduced in both *msh5Δ* and *exo1Δ*, giving rise to the possibility that Msh5 recruitment may be impacted in an *exo1Δ* background. It is unlikely that the absence of Exo1-mediated resection impairs the localization of Msh5, as previous studies have shown that in *exo1Δ*, joint molecule formation is normal despite the roughly 50% reduction in crossovers [29,30,84]. However, the possibility remains that Exo1 could promote Msh5 recruitment more directly through interactions with meiotic DNA intermediates and/or Msh5 itself. To address this, we analysed Msh5 binding in an *exo1Δ* mutant using a combination of ChIP-qPCR, ChIP-Seq, and cytological methods. We performed ChIP-qPCR analysis of Msh5 binding in *exo1Δ* at representative DSB hotspots (*BUD23*, *ECM3*, *CCT6*), chromosomal axes (*Axis I*, *Axis II*, and *Axis III*), centromeres (*CENIII*, *CENVIII*), and a DSB cold spot (*YCRO93W;* [85]). Enhanced Msh5 binding was observed in *exo1Δ* at some of the representative DSB hotspots (*ECM3*, *CCT6*) at 4 h and 5 h relative to the *wild-type*. Msh5 binding at the axes and centromeres in *exo1Δ* was similar to *wild-type* from 3 to 5 h (Fig 6A).

Msh5 ChIP-Seq was performed in meiotic time courses in 2 independent biological replicates of *exo1Δ* mutant at 3, 4, and 5 h (Material and methods). The Msh5 ChIP-seq data was normalized using the input. Msh5 binding data from the 2 replicates were highly correlated (r > 0.88) (S5 Fig). A total of 3,448 Msh5 peaks were observed in the *exo1Δ* mutant (see Materials and methods) at 5 h post entry into meiosis (S1 File). About 70% of these peaks (2,380 peaks) overlapped with the Msh5 peaks in wild type (3,397 peaks, [85]). A representative profile of Msh5 binding on chromosome III in *exo1Δ* shows Msh5 binds to DSB hotspots, axes, and centromeres as observed in *wild-type* strains (Fig 6B). These results suggest Msh5 binding locations in *exo1Δ* and *wild-type* are similar.

Msh5 binding at overlapping peak locations (5 h) appeared higher in the *exo1Δ* mutant at all time points (3 h, 4 h, 5 h; Fig 6B and S6A Fig). To further understand this observation, we compared the strength of the *wild-type* and *exo1Δ* Msh5 ChIP-seq signals at meiotic DSB cold spots. As shown in S6A Fig, the average Msh5 reads counts at all overlapping peak locations ranged from a minimum of 48 to a maximum of 74. For the *YCR093W* cold spot [86], the average read count (+/- 100 bp from the cold spot center) was 0 in both *wild-type* and *exo1Δ* (S6B Fig). We then extended this analysis to analyze a set of 25 meiotic DSB cold spots (S6C Fig; [87,88]). The average Msh5 read counts (+/- 100 bp from the cold spot center) were very low in both *wild-type* and *exo1Δ* (minimum of 0, maximum of 3; S6C Fig). Though very low, the average read counts for the 25 cold spots were higher in *wild-type* compared to *exo1Δ* at all time points (*p* < 0.001, Wilcoxon rank sum test). In addition, the Msh5 read counts appeared more variable in the *exo1Δ* background compared to *wild-type*, making it more difficult to assess the significance of the enhanced Msh5 read count observed in *exo1Δ* (S6C Fig). Thus, while the ChIP-seq data showed that Msh5 is recruited in *exo1Δ* strains, they were less conclusive with respect to showing that Msh5 recruitment was enhanced in *exo1Δ* relative to *wild-type*.

The Msh5 ChIP studies encouraged us to perform an analysis of Msh5 foci that formed in meiosis (Fig 6C). The average numbers of Msh5 foci per cell in *exo1Δ* at 3 h (34), 4 h (45), and 5 h (48) were comparable to the number of Msh5 foci in *wild-type* at the same time points (33, 42, and 48, respectively) (Fig 6D). However, measurements of foci intensity showed that the Msh5 foci appeared brighter in *exo1Δ* (Fig 6D). These observations support the ChIP-qPCR data showing enhanced Msh5 binding at some DSB hotspot loci in *exo1Δ* mutants. Together, the ChIP and Msh5 localization studies suggest that Msh4-Msh5 localization is not dependent on either the long-range resection activity of Exo1 or interaction with Exo1. This information, in conjunction with interference analysis of *exo1* nuclease defective mutants, supports a direct role for Exo1 in establishing interference. Overall, these results suggest Msh5 binding is not defective in *exo1Δ*, and that Exo1 negatively regulates the binding of Msh4-Msh5.

### Cdc9 ligase overexpression disrupts meiotic crossing over in exo1 DNA-binding domain mutants

If a role for Exo1 in meiotic recombination involved nick binding/protection, we reasoned that meiotic overexpression of *CDC9*, the budding yeast DNA ligase involved in DNA replication, could lead to premature ligation of DNA synthesis-associated nicks critical for maintaining biased resolution. This idea was encouraged by the work of Reyes and colleagues [90] who showed that overexpression of the budding yeast ligase Cdc9 disrupted DNA mismatch repair through the premature ligation of replication-associated nicks that act as critical repair signals. Furthermore, we posited that some *exo1* DNA-binding mutants that maintained near wild-type levels of crossing over might be especially susceptible to Cdc9 overexpression.

**Fig 7:**
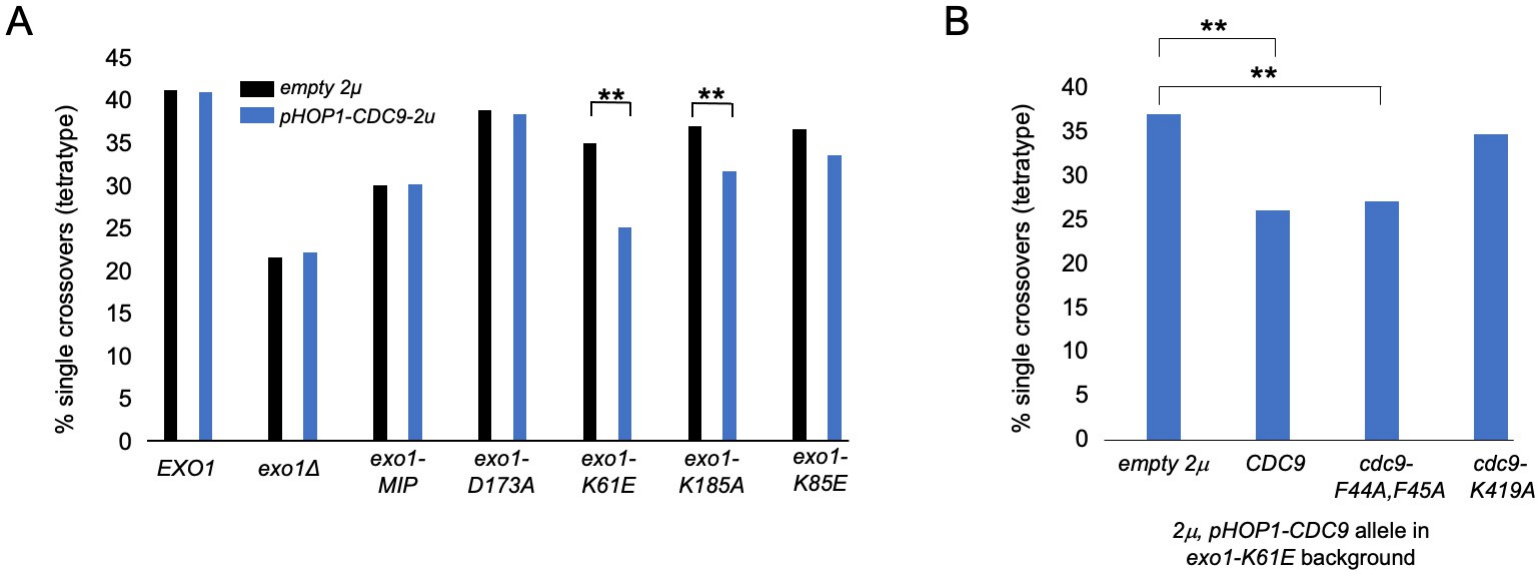
*CDC9* overexpression in meiosis disrupts the crossover functions of *exo1* DNA-binding mutants. (A) Diploid strains with the indicated *exo1* genotypes (S5 Table) were transformed with a *2μ URA3* vector containing no insert (*empty 2μ*, pRS426) or *CDC9* expressed from the *HOP1* promoter (*pHOP1-CDC9*, *2μ*, pEAM329) and then assessed for meiotic crossing over in the *CEN8-THR1* interval. Significance is shown between each *empty vector-pHOP1-CDC9* pair using a two-tailed Fisher’s exact test, with ** indicating *p* < 0.01. (B) A diploid strain containing the *exo1-K61E* allele was transformed with a *2μ URA3* vector containing no insert (empty 2μ), *CDC9*, *cdc9-F44A*,*F45A*, or *cdc9-K419A* expressed from the *HOP1* promoter and then assessed for meiotic crossing over in the *CEN8-THR1* interval. Significance is shown between each *empty 2μ vector-pHOP1-CDC9* pair using a two-tailed Fisher’s exact test, with ** indicating *p* < 0.01. Underlying data for Panel A can be found in S2C Table and S2 Data, and underlying data for Panel B can be found in S2D Table and S2 Data.

During meiosis *CDC9* expression appears to be low relative to *HOP1*, whose expression increases dramatically in meiotic prophase and remains high through dHJ resolution (approximately 6 h in meiosis; S4 Fig). We thus expressed *CDC9* under control of the *HOP1* promoter. As shown in Fig 7A, we saw no disruption of crossing over in *wild-type* or *exo1* mutants that contained intact DNA-binding domains (*EXO1*, *exo1-MIP*, *exo1-D173A*, Group I) or a statistically insignificant decrease in a mutant *(exo1-K85E*, Group II) predicted to be defective in steps post-DNA bending [26]. However, we saw modest to severe losses of crossing over in 2 *exo1* DNA-binding mutant hypomorphs. As shown in Fig 7A, *pHOP1-CDC9* reduced single crossovers in *exo1-K185A* (Group IV) from 35.3% to 31.3% and in *exo1-K61E* (Group III) from 35.1% to 25.2%. Furthermore, the loss in crossing over in the *exo1-K61E* strain due to *CDC9* overexpression was not seen when *CDC9* contained a mutation in the ligase active site that confers lethality (*K419A*; Fig 7B; [90–93]). However, this loss was still observed when *CDC9* contained the *F44A*,*F45A* mutations that disrupt interactions between Cdc9 and PCNA in vitro but do not confer lethality [94]. These observations are consistent with Cdc9 ligase activity being important for the loss of crossing over, but not Cdc9-PCNA interactions, which are thought to coordinate Okazaki fragment ligation during DNA replication [94]. Importantly, in conjunction with the *RAD27* complementation experiments, they provide evidence for a nick protection role for Exo1 in crossover formation.

## Discussion

**Fig 8:**
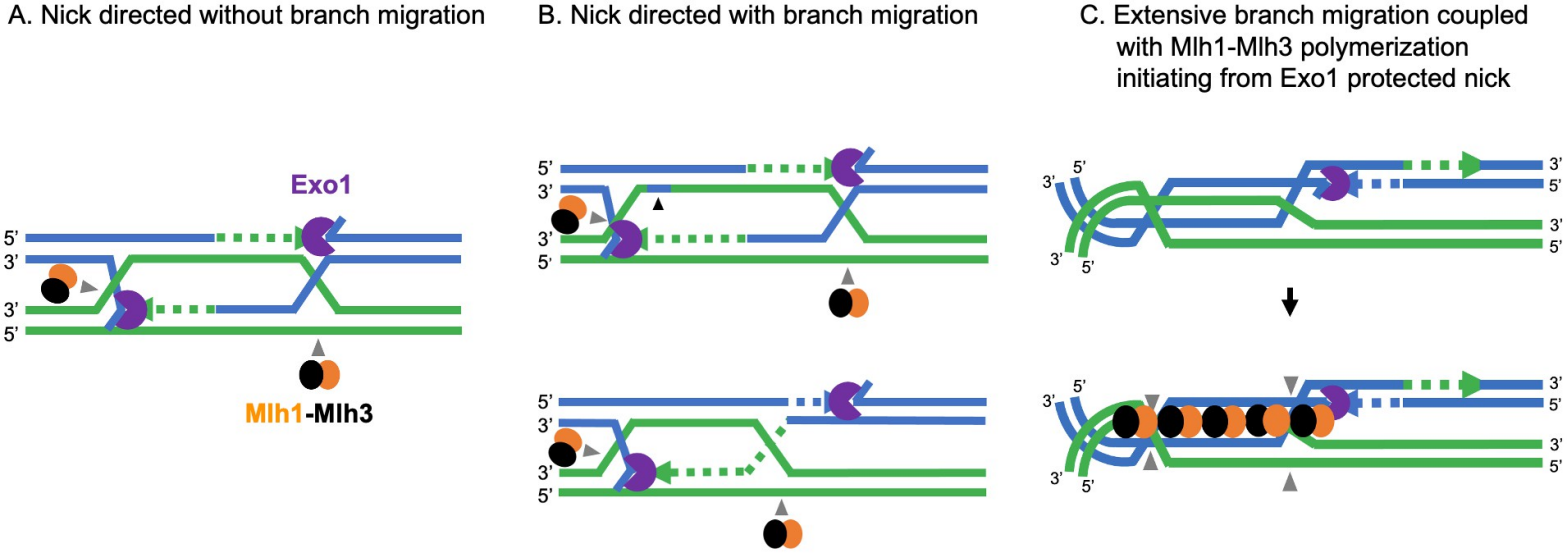
Models for biased resolution of dHJs. (A) Canonical model. In the major interference-dependent crossover pathway, a D-loop intermediate is stabilized by ZMM proteins including Msh4-Msh5 to form a SEI intermediate. DNA synthesis from the SEI, followed by second-end capture, results in the formation of the dHJ intermediate that is stabilized by Msh4-Msh5. Biased resolution of the 2 junctions results in crossover formation. In this model, Exo1 protection of the nick/flap structure recruits Mlh1-Mlh3 to nick the DNA strand opposite the Exo1 protected nick. (B) dHJ resolution through limited branch migration (reference [43]; upper panel; reference [96], lower panel). In these models, 1 or both junctions of the dHJ move prior to resolution. In the model presented in the upper panel, Exo1 protects unligated nicks resulting from DNA synthesis and recruits Mlh1-Mlh3 as in panel A. A nick translation event through resolution-independent nicks results in short patches of repair during branch migration (shown by black arrow). In the model presented in the lower panel, Exo1-protection of nicks recruits Mlh1-Mlh3 as in panel A. (C) dHJ resolution through extended branch migration [97]. Branch migration creates a substrate for Mlh1-Mlh3 polymerization [34]. In such a model, the signaling imposed by the binding of Exo1 to nicks acts at a distance. Mlh1-Mlh3 is recruited by Exo1 and forms a polymer with a specific polarity that can displace other factors or be activated upon interaction with such factors. The polymer is activated to introduce a nick on 1 strand of the duplex DNA on type II dHJs when it forms a critical length required for stability. See text for details. dHJ, double Holliday junction; SEI, single-end invasion.

In this study, we identified a role for Exo1 in meiotic crossing over that is dependent on its ability to bind to nicked/flapped DNA structures. This conclusion is supported by the finding that meiotic expression of the structurally similar Rad27 nuclease can partially compensate for the loss of crossovers in the absence of Exo1 and that meiotic overexpression of the Cdc9 ligase conferred a significant crossover defect in *exo1* DNA-binding domain mutants. Based on these observations, we propose that Exo1 acts in meiotic crossover formation by binding to nicks/flaps analogous to those created during lagging strand DNA synthesis (Fig 8). In contrast to the functions of Rad27 and Exo1 during replication, which cleave 5′ flaps in mechanisms that facilitates ligation of the resulting nick [95], the Exo1 meiotic crossover function occurs independently of nuclease activity. Such a nuclease-independent activity likely serves to protect nicks or flaps in recombination intermediates from premature ligation, ensuring their incorporation into a resolution mechanism. In addition, a nick/flap bound Exo1 could act to recruit Mlh1-Mlh3 to the dHJ. In support of this idea, work by Manhart and colleagues [34] showed that the presence of Mlh1-Mlh3 polymer at a nicked strand can direct the endonuclease to cut the opposite strand, providing a possible mechanism for how biased resolution could occur.

### Incorporating nick-protection with models of dynamic dHJs

Roles for a nicked recombination intermediate in forming meiotic crossovers have been proposed, with a summary of a few studies provided below. (1) Electron microscopy studies of Holliday junction structures purified from yeast cultures in pachytene failed to reveal open centers expected of fully ligated junctions [98], though the structure of dHJs in vivo is not well understood. (2) Nicked HJs are favorable substrates for resolution by resolvase proteins in vitro [99], and nicked HJs comprise a large proportion of Holliday junction structures observed in mutants defective in the structure-selective nucleases Yen1 and Mms4-Mus81, suggesting that they represent mitotic recombination intermediates [100]. (3) Whole-genome sequencing of meiotic spore progeny inferred that the resolution of dHJs is biased towards new DNA synthesis tracts, implying that these tracts contain distinguishing features such as nicks [43]. (4) Biochemical studies have led to models in which nicks persisting during dHJ formation could provide a substrate for continued loading of MMR/replication factors implicated in dHJ resolution (e.g., RFC, PCNA, Msh4-Msh5; [46,48]). Furthermore, Kulkarni and colleagues [48] and Cannavo and colleagues [46] showed that PCNA, which is loaded onto primer template junctions during DNA replication, promotes nicking by Msh4-Msh5 and Mlh1-Mlh3. The above observations, however, are challenging to reconcile with observations in *S*. *cerevisiae* indicating that single strands of DNA present in dHJs appear to be continuous (at least at the resolution of denaturing alkaline gels; [20,21]) and dHJs are much more dynamic than predicted based on the canonical DSB repair model ([43,96,97]; Fig 8A). It is possible that nicked recombination intermediates are not detected because they are transient, yet capable of providing signals critical for crossover formation, such as loading of PCNA. In this sense, joint molecules detected in *exo1Δ* strains [30] may be different in structure from those seen in *wild-type*.

dHJs have often been portrayed as static intermediates, constrained to the location of the initiating DSB (Fig 8A). While the nick protection mechanism proposed here can be understood in the context of a canonical model in which Exo1 recruits Mlh1-Mlh3 to nick the single-stranded DNA opposite the Exo1 protected nick (Fig 8A), recent work indicated that dHJs undergo significant branch migration in vivo. Recently, Marsolier-Kergoat and colleagues [43], Peterson and colleagues [96], and Ahuja and colleagues [97] showed in meiosis that 1 or both junctions of the dHJ can move independently or in concert prior to resolution. Marsolier-Kergoat and colleagues [43] estimated the frequency of branch migration to be on the order of 28%, and Ahuja and colleagues [97], based on a detailed analysis of a well-defined recombination hotspot containing a high density of single-nucleotide polymorphisms, inferred that approximately 50% of crossovers occurred in locations where both HJs are located on one side of the initiating DSB, with a much higher number of crossovers showing some migration.

How can nick protection be incorporated into crossover mechanisms that involve branch migration of HJs? One possibility is that nicks are translocated through “nick translation” [43]. For certain types of branch migration, this mechanism would push the nicks to a new dHJ location, allowing bias to be maintained (Fig 8B, upper panel). In one such model [43], Exo1 nick protection would occur when DNA synthesis encounters a 5′ end and resolution by Mlh1-Mlh3 would occur (Fig 8B). Alternatively, Mlh1-Mlh3 could nick at a distance from the Exo1-protected nick ([96], Fig 8B, lower panel), which could be reconciled based on previous studies showing that MLH proteins form polymers on DNA and can make multiple nicks on DNA [34,101–103]. In the Marsolier-Kergoat [43] model, the synthesis of new DNA tracts has been hypothesized to be followed by processing of the resultant 5′ flap to create a nick. Though appealing, this model needs to be balanced with our findings that the catalytic activity of Rad27 is not necessary to rescue crossing over in an *exo1Δ* strain, providing support for the catalytic activity of Exo1 being attenuated during crossover resolution.

A key aspect of extensive branch migration is that it should prevent DNA nicks from serving as substrates for biased resolution because they locate away from sites of dHJ resolution. To reconcile this observation with our analysis of Exo1, we suggest that such nicks act as substrates for the activation of an Mlh1-Mlh3 polymer (Fig 8C). Previous work showed that Mlh1-Mlh3 requires a large DNA substrate for nuclease activation and that polymerization barriers impeded its nuclease activity [34]. As such, branch migration may provide a way to move the dHJ from a constrained state that is occupied by factors that establish the dHJ such as Msh4-Msh5. In such a model, the signaling imposed by the binding of Exo1 to nicks could act across a distance, and through an initial Exo1-Mlh1-Mlh3 interaction, allowing the Mlh1-Mlh3 polymer to occupy the comparatively unconstrained DNA away from the invasion site (Fig 8C). Thus, we may consider the Exo1-nick interaction site as a nucleation point for Mlh1-Mlh3. This would add asymmetry to the polymer and ensure that Mlh1-Mlh3 nicks in a biased manner. We illustrate this within the context of a model presented by Manhart and colleagues [34], in which Mlh1-Mlh3 requires polymerization across multiple kilobases to be catalytically active to cleave type II Holliday junctions. Variations of such a model have been presented by Kulkarni and colleagues [48]. These models would also provide an explanation for the importance of Exo1-Mlh1-Mlh3 interactions during meiotic crossing over (but see below). In this model, we see Exo1-nick interactions as a means of guarding essential nicks from premature ligation. This would ensure that the dHJ remains “flexible” if needed for Mlh1-Mlh3 polymerization and activation. These models are not mutually exclusive, and further work is required to understand how resolution factors interact with mobile and static dHJs.

An additional challenge with the models presented in Fig 8 is that while Exo1 and FEN1 bind flap structures to coordinate tail removal and ligation steps, the endonuclease activities of these proteins do not appear to be required for crossover resolution. However, the finding that ligase overexpression can disrupt crossing over in *exo1* DNA-binding hypomorphs suggests that a ligatable nick serves as a critical recombination intermediate. One possibility is that there is a coordinated displacement of Exo1 by Mlh1-Mlh3 that induces Mlh1-Mlh3 nicking on the opposite strand. In such a model, there could be other processing events that remove 5′ flaps such as one involving Msh2-Msh3 recognition of the flap, followed by endonuclease cleavage by Rad1-Rad10 [104]. It is also worth noting that complementation of the *exo1Δ* strain with the *pEXO1-rad27-D179A* plasmid was observed in a *RAD27* strain background capable of removing 5′ flaps.

Does Exo1 direct Mlh1-Mlh3 nicking? A coordinated set of steps are required in meiotic recombination to promote Exo1-mediated resection of DSBs, D-loop formation, DNA polymerase-mediated synthesis of the invading 3′ strand, Exo1 protection of flaps/nicks, and ligation of cleaved dHJs. The transitions between these steps are likely to proceed through mechanisms that involve posttranslational modifications (e.g., [105]). Recent studies have shown that Exo1 has a key role in the activation of Mlh1-Mlh3 through Cdc5 Kinase [47], and a protein association/mass spectrometry study [106] suggested that Mlh1-Mlh3 meiotic interactions with Exo1 are dynamic. However, we and others have shown that the *exo1-MIP* mutant defective in Mlh1 interactions displays an intermediate defect in meiotic crossing over (this study and [30]), suggesting the possibility of other factors/structures facilitating Mlh1-Mlh3 endonuclease activation. Consistent with this, Mlh1-Mlh3 foci appear to form in meiotic prophase in the absence of Exo1 [47] and *RAD27* complementation of the *exo1Δ* crossover defect was not complete and did not improve crossover interference (S4 Table). One mechanism consistent with the above observations is that a DNA structure or protein barrier forms during meiotic recombination that activates the Mlh1-Mlh3 endonuclease, analogous to that seen for activation of type I restriction enzymes through head-on collision of 2 translocating enzymes [107]. Understanding how these transitions occur will require both in vitro reconstruction studies using purified proteins and novel in vivo approaches to identify nicks in dHJ intermediates.

### A role for Exo1 in promoting genetic interference

In baker’s yeast, the ZMM factor Zip3 is an early marker for crossover designation and interference, prior to the formation of physical crossovers, and previous work has suggested that crossover interference and crossover assurance are carried out as distinct functions by the ZMMs [108]. These observations indicate that crossover interference is established early, prior to dHJ resolution (reviewed in [109]). Interestingly, while *mlh3Δ* mutants lose dHJ resolution bias, residual interference in *mlh3Δ* mutants suggest that biased resolution is not required for interference. In contrast, a more severe loss of crossover interference in *exo1Δ* (Fig 4C) suggests a role prior to preserving resolution bias, analogous to ZMM proteins that designate crossovers and assure interference on the maturing dHJ. The interference role for Exo1 was also reflected in spore viability patterning, as only the full *exo1Δ* displayed a viability pattern consistent with non-disjunction. While it is not possible to use our data to precisely determine how crossover patterning is disrupted in *exo1Δ*, the strong interference defect and non-disjunction spore viability pattern seen in *exo1Δ* strains was similar to that seen for ZMM proteins that act early in imposing interference.

Does the 5′ to 3′ resection defect in meiotic DSB processing seen in *exo1Δ* and *exo1-D173A* strains [30] explain why *exo1Δ* mutants show an interference defect? A defect in Exo1-mediated resection could, for example, disrupt crossover interference by preventing the recruitment of ZMM factors that stabilize meiotic recombination intermediates that act in crossover interference. The following observations argue against this idea: (1) Msh5 association to chromatin appeared higher in an *exoΔ* background, arguing against the loss of a key ZMM recruitment factor that acts in crossover interference (Fig 6). (2) *exo1* point mutants that targeted the catalytic sites of Exo1 (e.g., *exo1-D173A*) displayed robust interference (S4 Table). (3) We observed suppression of the *exo1-G236D* crossover defect when a Group I catalytic mutation was also present (*exo1-D173A*,*G236D*; Fig 3B). This phenotype can be explained by a model in which *exo1*-Group I mutants are hyperactivated (rather than being defective) for the ZMM pathway, and the *exo1-G236D* mutation, which reduces Exo1 binding to DNA, suppresses the *exo1*-Group I mutant phenotype. Together, these observations, and previous work showing that joint molecule formation occurs at wild-type levels in *exo1Δ* mutants [30], suggest that the interference defect seen in *exo1Δ* mutants is not the result of resection-dependent joint molecule instability or defects in recruiting Msh4-Msh5.

We hypothesize that the increased Msh5 binding/focus intensity observed in *exo1Δ* is due to the delayed turnover of meiotic recombination intermediates. This argument is consistent with previous work by Zakharyevich and colleagues [30], who showed that while the appearance and levels of recombination intermediates (single-end invasion, interhomolog dHJs) were not reduced in *exo1Δ*, they persisted for a longer time (approximately 1 h in SK1 strains) compared to *wild-type*. Can we directly link enhanced Msh5 binding/focus intensity in *exo1Δ* to its reduced crossover interference phenotype? Given that both *Exo1*^*-/-*^ mouse oocytes [110] and *Exo1*^*D173A*^ [38] mouse spermatocytes are proficient in MLH1/MLH3 localization, Exo1 may have a unique role in the turnover of Mlh1-Mlh3, perhaps by negatively regulating its loading in a manner that is linked to its role in crossover interference. In such a model, the disruption of such loading in *exo1Δ* would result in meiotic recombination intermediates eventually being resolved by Mms4-Mus81 during Meiosis I and Slx1-Slx4 and Yen1 during Meiosis II to generate Class II crossovers that are interference independent.

While the resection role of Exo1 appears dispensable for maintaining genetic interference, it remains unclear how Exo1 contributes to crossover interference. Interestingly, Exo1 has been observed to interact with Msh2 through an Msh2-interacting-peptide box (SHIP; Fig 1C; [28]), suggesting the possibility that Msh4-Msh5 also interacts with Exo1. However, a direct interaction with Msh4-Msh5 has yet to be characterized and while Msh4-Msh5 localization is not dependent on Exo1 (Fig 6), we have not determined if such localization is critical for downstream Exo1 functions. As an aside, we attempted to improve *pEXO1-RAD27* complementation of crossover functions in *exo1Δ* strains by expressing Rad27 fused to the C-terminal region of Exo1 that contains the SHIP boxes; unfortunately, these constructs were not functional. Teasing apart how Exo1 coordinates roles in crossover selection and resolution will be critical to understand the mechanism of biased dHJ resolution.

## Materials and methods

### Media and yeast strains

*S*. *cerevisiae* SK1 yeast strains used in this study (S5 Table) were grown at 30 °C in either yeast extract peptone-dextrose (YPD) or synthetic complete media supplemented with 2% glucose [111]. When required, geneticin (Invitrogen, San Diego) or nourseothricin (Werner BioAgents, Germany) were added to media at recommended concentrations [112]. Meiotic crossing over was analyzed in the SK1 isogenic background using spore-autonomous assays to measure crossing over in the *CEN8-THR1* interval on Chromosome VIII (SKY3576/SKY3575 parental diploids, [54]) and in the SK1 congenic EAY1108/EAY1112 background (4 intervals on Chromosome XV, [57]). Sporulation media was prepared as described [57].

### Strain constructions

Mutant alleles were transformed into *S*. *cerevisiae* with integration plasmids, *geneXΔ*::*KANMX* PCR fragments or on *CEN6-ARSH4* and 2μ plasmids (S6 Table) using standard techniques [111,113]. To confirm integration events, genomic DNA from transformants was isolated as described previously [114]. Transformants bearing *EXO1*::*KANMX* and *exo1*::*KANMX* mutant derivatives were screened for integration by analyzing DNA fragments created by PCR using primers AO4061 and AO3838 (all primers in this study were purchased from Integrated DNA Technologies, Coralville, Iowa, United States of America). Integration of *exo1* alleles was confirmed by DNA sequencing of the DNA fragments created by PCR using primers AO3666 and AO3399 (S7 Table). To confirm integration of *geneXΔ*::*KANMX* mutations, primers that map outside of the *geneXΔ*::*KANMX* PCR fragment were used (S7 Table). At least 2 independent transformants for each genotype were made.

### exo1 integrating and EXO1, RAD27, and CDC9 expression plasmids

Plasmids created in this study are shown in S6 Table and the oligonucleotide primers used to make plasmids are shown in S7 Table. Genes expressed in plasmids are from the SK1 strain background [115].

pEAI422 (4.7 KB; *exo1Δ*::*KANMX)* was built using HiFi DNA Assembly (New England Biolabs). It contains a complete deletion of the *EXO1* open reading frame but retains 280 bp of 5′ flanking and 340 bp of flanking 3′ sequence. This plasmid was digested with *Spe*I and *Sma*I to release the *exo1Δ*::*KANMX* fragment prior to transformation.

pEAI423 (7.2KB; *EXO1-KANMX*) contains the entire *EXO1* gene with approximately 300 bp of promoter sequence and approximately 500 bp of sequence downstream of the stop codon linked to the *KANMX* marker. In this construct, there are approximately 300 base pairs of immediate downstream sequence to retain the small gene of unknown function that is immediately found after *EXO1*, followed by *KANMX*, followed by downstream homology. pEAI423 was created using HiFi assembly of the following DNA fragments: (1) *Bam*H1 digested pUC18. (2) An *EXO1* gene fragment made by PCR-amplifying SK1 genomic DNA with primers AO4030 and AO4031. (3) A *KANMX* gene fragment made by PCR-amplifying plasmid pFA6 [116] with AO4032 and AO4033. (4) Downstream *EXO1* sequences made by PCR-amplifying SK1 genomic DNA with AO4034 and AO4035. Integration of this construct confers a *wild-type EXO1* genotype. Derivatives of pEAI423 containing mutations in *EXO1* were constructed with the Q5 mutagenesis kit (New England Biolabs) using pEAI423 as template and the oligonucleotides shown in S7 Table. The sequence of the entire open reading frame of *EXO1* in *wild-type* and mutant constructs was confirmed by DNA sequencing in the Cornell Bioresource Center using primers AO275, AO643, AO694, AO804, AO2383, AO3886, AO4028. pEAI423, and mutant derivatives were digested with *Spe*I and *Nhe*I to introduce *EXO1*::*KANMX* or *exo1*::*KANMX* fragments into SKY3576 and SKY3575 by gene replacement.

pEAA726 (10.5 KB; *MLH3*, *CEN6-ARSH4*, *URA3*), an *MLH3* complementation vector, was created by ligating a *Bam*HI-*Sal*I *MLH3-KANMX* fragment from pEAA636 into the pRS416 (*ARS/CEN*, *URA3;* [117]) backbone digested with *Bam*HI and *Sal*I.

pEAA722 (6.4 KB; *RAD27*, *CEN6-ARSH4*, *URA3*), a *RAD27* complementation vector, was constructed in 2 steps. First, a fragment of the *RAD27* gene containing 259 bp upstream and 300 bp downstream sequence was created by PCR amplification of SK1 genomic DNA using primers AO4707 + AO4708. The resulting fragment was digested with *Spe*I + *Kpn*I and ligated into pRS416 digested with *Spe*I + *Kpn*I to create pEAA722.

pEAA715 (7.8 KB; *EXO1*, *CEN6-ARSH4*, *URA3*) was constructed in 2 steps. First, a fragment of the *EXO1* gene containing 400 bp upstream and downstream sequence was created by PCR amplification of SK1 genomic DNA using primers AO4631 and AO4636. The resulting fragment was digested with *Spe*I + *Kpn*I and ligated into pRS416 digested with *Spe*I + *Kpn*I to create pEAA715.

pEAA720 (6.8 KB), a *pEXO1-RAD27* (*EXO1* promoter driving *RAD27* expression), *CEN6-ARSH4*, *URA3* vector, was constructed by HiFi assembly (New England Biolabs) using the following fragments: (1) pRS416 (*CEN6-ARSH4*, *URA3)* digested with *Kpn*I + *Xba*I. (2) *EXO1* promoter region (400 bp immediately upstream ATG) amplified from the SK1 genome using AO4643 + AO4644. (3) The entire *RAD27* ORF amplified from the SK1 genomic DNA using AO4645 + AO4637. (4) The *EXO1* downstream region (400 bp immediately downstream of the stop codon) amplified from the SK1 genomic DNA using AO4638 + AO4636. *rad27* mutant alleles were constructed with the Q5 mutagenesis kit (New England Biolabs) using pEAA720 as template. The oligonucleotides used to make the alleles are shown in S7 Table. All *RAD27* plasmid constructs were confirmed by DNA sequencing.

pEAM327 (9.3 KB), a *CDC9*, *2μ*, *URA3* plasmid, was constructed in 2 steps. First a fragment of the *CDC9* ORF, containing 1,000 bp upstream and 400 bp downstream sequence was created by PCR amplification of SK1 genomic DNA using primers AO4783 and AO4784. The resulting fragment was digested with *Hind*III and *Kpn*I and then ligated to pRS426 (*2μ*, *URA3*), backbone also digested with *Hind*III and *Kpn*I to create pEAM327.

pEAM329 (8.8 KB) is a *2μ*, *URA3* plasmid that expresses *CDC9* from the *HOP1* promoter (*pHOP1-CDC9*). It was constructed through Hifi assembly using the following fragments: (1) A DNA backbone was created by PCR amplification of pEAM327 using primers AO4837 and AO4838; the resulting DNA fragment lacks the *CDC9* promoter. (2) A 500 bp DNA fragment of the *HOP1* promoter (up until the *HOP1* start codon) was created by PCR amplification of SK1 genomic DNA using primers AO4839 and AO4840. The 2 fragments were then assembled using Hifi Assembly to create pEAM329, which was confirmed by DNA sequencing.

### Tetrad analysis

Diploids derived from EAY1108/EAY1112 were sporulated using the zero*-*growth mating protocol [118]. Briefly, haploid parental strains were patched together, allowed to mate overnight on complete minimal plates, and then struck onto selection plates to select for diploids. The resulting diploids were then transferred from single colonies to sporulation plates where they were incubated at 30 °C for 3 days. Tetrads were dissected on minimal complete plates and then incubated at 30 °C for 3 to 4 days. Spore clones were replica-plated onto relevant selective plates and assessed for growth after an overnight incubation. Genetic map distances were determined by the formula of Perkins [119]. Interference calculations from three-point intervals were conducted as described [82,120,121]. Statistical analysis was done using the Stahl Laboratory Online Tools (https://elizabethhousworth.com/StahlLabOnlineTools/) and VassarStats (http://faculty.vassar.edu/lowry/VassarStats.html) and the Handbook of Biological Statistics (http://udel.edu/mcdonald/statintro.html).

Interference was measured by the Malkova method [60]. This method measures cM distances in the presence and absence of a neighboring crossover. The ratio of these 2 distances denotes the strength of interference, with a value closer to 1 indicating a loss of interference. Significance in the distribution of tetrads was measured using a G test [122] and values of *p* < 0.05 were considered indicative of interference. The COC was also measured for each interval by calculating the ratio of observed versus expected double crossovers.

### Spore-autonomous fluorescence assay

We analyzed crossover events between spore-autonomous fluorescence reporter constructs at the *CEN8-THR1* locus on Chromosome VIII (SKY3576, SKY3575; [56]). To produce diploid strains for analysis in the spore-autonomous fluorescence assay, haploid yeasts of opposite mating types were mated by patching together on YPD from freshly streaked colonies and allowed to mate for 4 h, and then transferred to tryptophan and leucine dropout minimal media plates to select for diploids. Diploids grown from single colonies were patched onto sporulation plates and incubated at 30 °C for approximately 72 h. Diploid strains containing *ARS-CEN* or *2μ* plasmids were also grown on selective media to maintain the plasmids until just prior to patching onto sporulation plates. Spores were treated with 0.5% NP40 and briefly sonicated before analysis using the Zeiss AxioImager.M2. At least 500 tetrads for each genotype were counted to determine the % tetratype. Two independent transformants were measured per allele. A statistically significant difference from *wild-type* and *exo1Δ* controls based on χ2 analysis was used to classify each allele as exhibiting a wild-type, intermediate, or null phenotype. We applied a Benjamini–Hochberg correction [123] at a 5% false discovery rate to minimize α inflation due to multiple comparisons. See S2 Data for the underlying datasets that show this correction.

### Sensitivity to methyl-methane sulfonate

Yeast strains were grown to saturation in YPD liquid media, after which they diluted in water and spotted in 10-fold serial dilutions (undiluted to 10^−5^) onto YPD media containing 0.04% MMS (v/v; Sigma). Plates were photographed after a 2-day incubation at 30 °C. S3 Fig is a representative image of at least 5 independent platings.

### Exo1 homology model

The crystal structure of human EXO1 in complex with 5′ recessed DNA (amino acids 2 to 356; [26]) was used to map residues in yeast Exo1 critical for function. A homology model was constructed (Fig 1B) using the Phyre2 software (http://www.sbg.bio.ic.ac.uk/phyre2/html/page.cgi?id=index). The predicted structure was aligned to human EXO1 (PDB ID: 3QEB) using Pymol (https://pymol.org/2/). Metal binding residues mutated in this study were D78, D171, and D173. Active site residues mutated were H36, K85, R92, and K121. Hydrophobic wedge residues mutated were S41, F58, and K61 and DNA-binding residues mutated were K185 and G236. For S1 Fig, the Exo1 protein sequence from *S*. *cerevisiae* was submitted to the BLASTP server at NCBI and run against the landmark database. A multiple-sequence alignment of Exo1 homologs from different model organisms was generated with MAFFT using default settings [124].

### Purification of Exo1

Exo1-FLAG variants (Exo1, exo1-D173A, exo1-G236D) were purified from pFastBac1 constructs (S6 Table) in the baculovirus/*Sf9* expression system as described by the manufacturer (Invitrogen, Waltham, Massachusetts, USA) with the following modifications [125]. Briefly, 250 ml of *Sf9* cell pellet was resuspended in 7.5 ml of a buffer containing 50 mM Tris (pH 7.9), 1 mM EDTA, 0.5 mM PMSF, 0.5 mM β-mercaptoethanol, 20 μg/mL leupeptin, and 0.25× Halt protease inhibitor cocktail (Thermo Fisher Scientific, Waltham, Massachusetts, USA). The suspension was incubated on ice for 15 min, after which NaCl was added to a final concentration of 100 mM and glycerol was added to a concentration of 18% (v/v) and incubated on ice for 30 min. The cells were centrifuged at 30,000xg for 30 min. The cleared lysate was applied to a 2 ml SP Sepharose Fast Flow column at a rate of approximately 15 ml/h. The column was washed with 10 ml of a buffer containing 50 mM Tris (pH 7.9), 10% glycerol, 100 mM NaCl, 0.5 mM PMSF, 5 mM β-mercaptoethanol, and 6.7 μg/ml leupeptin. Exo1 variant was eluted with the above buffer containing 700 mM NaCl. Fractions containing Exo1 protein variant were pooled and applied to 0.3 ml of M2 anti-FLAG agarose beads (Sigma-Aldrich, St. Louis, Missouri, USA) in batch, incubating with rotation for approximately 1.5 h at 4 °C. Unbound protein was isolated by centrifugation at 2,000 RPM for 5 min in a swinging bucket centrifuge at 4 °C. The resin was resuspended in 7 ml of buffer containing 20 mM Tris (pH 7.9), 150 mM NaCl, 10% glycerol, 0.1% NP40, 0.5 mM PMSF, 0.5 mM β-mercaptoethanol, 6.7 μg/ml leupeptin, and one-third of a Complete Protease Tablet (Roche, Basel, Switzerland) for every 100 ml of buffer and flowed into an empty column at approximately 15 ml/h, allowing to pack. The column was then washed with 0.6 ml of the above buffer excluding the NP40 (wash buffer II). Exo1-FLAG variants were eluted using wash buffer II containing 0.1 mg/ml 3x-FLAG peptide (Sigma). After applying elution buffer, the flow was stopped after the first 3 fractions were collected and incubated for approximately 1 h before resuming flow and collecting fractions. Fractions containing Exo1 variant were pooled, flash frozen in liquid nitrogen, and stored at −80 °C. All purification steps were performed at 4 °C. Protein concentration was determined by the method of Bradford [126].

### DNA binding assays

Exo1 DNA binding assays were performed in 60 μl reactions for 10 min at 30 °C. Each reaction contained 15 nM homoduplex (AO3878 annealed to AO3144; S7 Table) or a 19 nt 5′ flap (AO3145 and AO3940 annealed to AO3144) substrate (each consisting of 8% ^32^P-labeled and 92% unlabeled), 35 mM NaCl, 20 mM Tris 7.5, 0.04 mg/ml BSA, 0.01 mM EDTA, and 0.1 mM DTT. Reactions were analyzed by filter binding to KOH-treated nitrocellulose filters [127] using a Hoeffer Scientific Instruments FH225 Filtering unit (San Francisco, California, USA). A detailed description of the filter binding protocol can be found in Chi and Kolodner [128]. Experiments were performed in triplicate (each repeat was performed on a different day) and data are presented in Fig 2D as the mean +/- standard deviation. Oligonucleotides were purchased from IDT (Coralville, Iowa, USA) and AO3144 was labeled at the 5′ end with [γ−32P] ATP (Perkin Elmer) and T4 polynucleotide kinase (New England Biolabs). Oligonucleotides were mixed at equimolar ratios in 10 mM Tris (pH 8.0), 50 mM NaCl, 1 mM EDTA. The mixtures were heated to 95 °C in a heat block for 5 min and then slowly cooled by turning off the block to room temperature (approximately 3 h). The annealed oligonucleotide substrates were purified by gel filtration (HR S-200 spin columns, Amersham Biosciences) and verified by gel electrophoresis.

### Western blot analysis

Exo1 protein levels were determined in *EXO1-13MYC* strains by western blotting. Briefly, an *EXO1-13MYC-KANMX* integrating vector (pEAI517; S6 Table) was used to introduce *EXO1-13MYC* and mutant derivatives into yeast (S5 Table). pEAI517 was constructed from pEAI423 (*EXO1-KANMX*) and pFA6a-13MYC::KanMX6 [63]. pEAI423 was PCR amplified using AO5293 and AO5294 (S7 Table), and the *13MYC* tag from pFA6a-13MYC::KanMX6 was PCR amplified using AO5295 and AO5296. The PCR fragments were gel extracted (Qiagen) and joined using Hifi Assembly (New England Biolabs) to create pEAI517. pEAI517 was used as a template for Q5 mutagenesis (New England Biolabs) to make the *exo1-13MYC* integrating vectors shown in S6 Table. The integration vectors were DNA sequenced prior to use.

Strains containing *EXO1-13MYC* and mutant derivatives (S5 Table) were grown in YPD to mid-log phase, harvested and lysed by bead beating (50 ml cultures, 3 × 60 s) in 250 μl lysis buffer (50 mM Tris-HCL (pH 7.9), 120 mM KCL, 5 mM EDTA, 0.1% NP-40, 10% glycerol, 1 mM PMSF) and an equal volume of acid-washed glass beads (500 micron, Sigma G8772). The lysate was collected and pooled with an additional 250 μl wash of the glass beads with lysis buffer. The lysate was then centrifuged at 13,000 RPM for 30 min at 4 °C, and 2× Laemmli buffer (Bio-Rad) was then added to the supernatant and proteins were separated via 10% SDS-PAGE and transferred to a nitrocellulose membrane using wet transfer. *exo1-13MYC* tagged alleles were detected with Anti-Myc (1:1,000, 4A6, Sigma-Aldrich) and peroxidase conjugated Anti-Mouse IgG secondary antibody (1:10,000, Sigma-Aldrich). Glucose-6-phosphate dehydrogenase was detected using Anti-G6PDH antibody (1:5,000, Sigma-Aldrich) and peroxidase conjugated Anti-rabbit IgG secondary antibody (1:10,000, Invitrogen). The blots were developed using Clarity Western ECL substrate (Bio-Rad) and imaged using a Bio-Rad ChemiDoc MP imager.

### Nuclease assays

Exo1 nuclease reactions were performed on supercoiled 2.7 kb pUC18 DNA (Invitrogen), or pUC18 DNA nicked by incubation with Nt.BstNBI (New England Biolabs, Ipswich, Massachusetts, USA; [32,34]). Briefly, 20 μl reactions (0 to 30 nM Exo1 or mutant derivative with 3.6 nM plasmid DNA) were assembled in a buffer containing 20 mM HEPES-KOH (pH 7.5), 20 mM KCl, 0.2 mg/ml BSA, 1% glycerol, and 5 mM MgCl_2_. Reactions (37 °C, 1 h) were stopped by the addition of a stop mix solution containing final concentrations of 0.1% SDS, 14 mM EDTA, and 0.1 mg/ml Proteinase K (New England Biolabs) and incubated at 37 °C for 20 min. Products were resolved by 1.2% agarose gel containing 0.1 μg/mL ethidium bromide. Samples were prepared and gels were run as described previously [34]. Gel quantifications of independent reactions were performed using GelEval (FrogDance Software, v1.37) using negative control reactions as background.

### Chromatin immunoprecipitation

Yeast strains KTY753, KTY756, KTY757, NHY1162, and NHY1168 used in the ChIP-Seq, ChIP-qPCR, and Msh5 localization analyses (Fig 6) are all derivatives of the *S*. *cerevisiae* SK1 strain. The *exo1Δ*:: *KanMX4* marker in KTY753, KTY756, and KTY757 was created using homologous recombination based gene knockout approach in the NHY1162/1168 background [61]. The transformed colonies were verified by PCR using primers designed for the *EXO1* flanking regions. Msh5 ChIP was performed using polyclonal Msh5 antibody (generated in rabbit) and Protein A Sepharose beads (GE Healthcare, Chicago, Illinois, USA) on synchronized meiotic cultures as described in Nandanan and colleagues [85]. The immunoprecipitated DNA was collected at 3 h, 4 h, and 5 h post entry into meiosis and used for ChIP-qPCR and ChIP-Seq.

The DNA enrichment for the Msh5 ChIP-qPCR was estimated with reference to the input at each time point. Msh5 enrichment data for the *wild-type* was from Nandanan and colleagues [85]. ChIP-qPCR was performed on 2 independent biological replicates of Msh5 immunoprecipitated DNA samples from *exo1Δ* (3 h, 4 h, and 5 h). Errors bars are estimated using the standard deviation from 2 independent biological replicates. Msh5 binding was analyzed at representative DSB hotspots (*BUD23*, *ECM3*, *CCT6*), axes (*Axis I*, *Axis II*, *Axis III*), centromeres (*CENIII*, *CENVIII*), and DSB cold spot (*YCR093W*). Chromosomal coordinates for these regions and the primer sets used for the qPCR are described in Nandanan and colleagues [85].

Msh5 ChIP-Seq data from the Illumina platform were processed as described in Nandanan and colleagues [85]). The raw sequence data are deposited in the National Centre for Biotechnology Information Sequence Read Archive under accession number PRJNA780068 (https://www.ncbi.nlm.nih.gov/sra/?term=PRJNA780068). Genome-wide Msh5 binding plots in *exo1Δ* were generated by partitioning the genome into equal-sized bins of 10 bp. The number of Msh5 reads in each bin for each sample was calculated and normalized with its respective control sample (input) using NCIS as described in Nandanan and colleagues [85]. Read counts were smoothened using ksmooth (function in R) with a bandwidth of 1 kb. The normalization of reads, background subtraction, smoothening, and plotting were done using R (version 3.3).

To identity the Msh5 peaks in *exo1Δ*, we considered reads that are uniquely mapped in the genome. Since the *exo1Δ* ChIP-Seq replicates showed high correlation (r > 0.88), we used pooled replicates to identify the Msh5 peaks. Msh5 peaks were identified using MACS (Model-based Analysis for ChIP-Seq; http://liulab.dfci.harvard.edu/MACS/ [129]) as described in Nandanan and colleagues [85]. Peaks with *P* value > 10^−5^ were filtered out and the final Msh5 peaks are shown in S1 File.

### Cytological analysis of Msh5 foci

Chromosome spreads (3 h, 4 h, and 5 h) were prepared from synchronized meiotic cultures (3 h, 4 h, and 5 h) as described [108,130,131]. Msh5 staining was performed using primary antibody against Msh5 [108] at 1:500 dilution, followed by secondary antibody (Alexa fluor 488, Thermo Fisher Scientific) at 1:1,500 dilution. The Msh5 stained samples were imaged using an epi-fluorescence microscope (BX51, Olympus) with a 100× objective (NA, 1.3). Images were captured by the CCD camera (CoolSNAP, Roper) processed using iVision (Sillicon) software. To quantify Msh5 focus intensity, the mean fluorescence of a whole nucleus was quantified with Fiji (ImageJ). The final fluorescence intensity of Msh5 was normalized with DAPI intensity for each nucleus. Fluorescence intensity refers to pixel intensity per unit area on chromosome spreads.

## Supporting information

S1 Fig Alignment of Exo1 protein sequences from *S*. *cerevisiae* (accession # NP_014676), *S*. *pombe* (NP_596050.1), *H*. *sapiens* (NP_003677), *M*. *musculus* (NP_036142) and *D*. *melanogaster* (NP_477145). Sequence alignment of Exo1 from different species. Triangles indicate mutations made in this study. See Materials and methods for sequence alignment details.
(TIFF)

S2 Fig Spore viability profile of *wild-type* and the indicated *exo1* strains in the EAY1108/EAY1112 strain background. The percent of tetrads with 4, 3, 2, 1, and 0 viable spores are shown from the dissections presented in Fig 4 as well as the total number of tetrads dissected and the overall spore viability. Underlying data can be found in S6 Data.
(TIFF)

S3 Fig Sensitivity of *exo1* mutants to the DNA damaging agent MMS. *Wild-type* and the indicated *exo1* mutants were spotted in 10-fold serial dilutions onto YPD and YPD media containing 0.04% MMS (Materials and methods). Plates were photographed after a 2-day incubation at 30 °C. In the bottom most panel an *exo1Δ* strain (EAY4778) was transformed with an *ARS-CEN* vector containing no insert (pRS416), *EXO1* (pEAA715), or *RAD27* expressed from the *EXO1* promoter (*pEXO1-RAD27*, pEAA720). Underlying data can be found in S7 Data.
(TIFF)

S4 Fig mRNA seq and ribosome profiling of *EXO1*, *RAD27*, *CDC9*, and *HOP1* expression in SK1 meiosis. Data obtained from Brar and colleagues [74]. RPKM = Reads per kilobase of coding sequence per million mapped reads.
(TIFF)

S5 Fig Correlation analysis of the log2 of Msh5 reads (fold-change with reference to input) for *exo1Δ* replicates (a, b) at T = 3 h (panel A), 4 h (panel B), and 5 h (panel C). The Pearson’s correlation coefficient (r) is shown. Underlying data can be found in National Center for Biotechnology Information Sequence Read Archive, accession number PRJNA780068.
(TIFF)

S6 Fig Msh5 read counts in *wild-type* and *exo1Δ* at 25 DSB cold spots. (A) Boxplot comparing differences in average Msh5 reads in *wild-type* and *exo1Δ* mutant at overlapping Msh5 peak locations. Msh5 read counts were obtained from the Msh5 ChIP-Seq experiment presented in Fig 6B. Y axis shows the average of Msh5 read counts +/- 100 bp from the center of each peak (5 h) in *wild-type* and *exo1Δ*, and *p* value was calculated using Wilcoxon rank sum test and adjusted using Bonferroni correction, and *** indicates *p* values <0.001. (B) Zoomed-in region of the *YCR093W* cold spot ([88]; Fig 6B) showing very low Msh5 reads (unsmoothed) in both *wild-type* and *exo1Δ*. (C) Msh5 binding was compared in *wild-type* and *exo1Δ* at 25 cold spots [87,88] that were depleted for Msh5 in *wild-type*. These 25 were obtained by rank ordering 49 cold spots in Gerton and colleagues [87] and Shodhan and colleagues [88] based on Msh5 read counts in *wild-type*. The lowest 25 were then analyzed. The Y axis shows the Msh5 read count (unsmoothed) for *wild-type* and *exo1Δ* at 3, 4, and 5 h post-meiotic induction. The X axis indicates +/- 1 kb from the cold spot center. The average number of Msh5 read counts in *wild-type* (*WT*) and *exo1Δ* is presented for each time point (+/- 100 bp from cold spot center). (D) List of 49 cold spots in Gerton and colleagues [87] and Shodhan and colleagues [88] (left panel), 25 of which (right panel) were analyzed in this study and are presented in order from highest (*HXT1*) to lowest (*YGR289C*) Msh5 counts. Underlying data for S6 Fig can be found in National Center for Biotechnology Information Sequence Read Archive, accession number PRJNA780068.
(PDF)

S1 Table Structure function analysis of XPG family proteins. (PDF)

S2 Table (**A**) **Spore autonomous meiotic crossover analysis of *exo1* mutants**. Homozygous mutations were made by crossing 2 independently constructed strains with the *exo1* variants in the SKY3576 (containing cyan fluorescent protein; S5 Table) and SKY3575 (containing red fluorescent protein) backgrounds. Heterozygous mutations were made by crossing 2 independently constructed strains with *exo1* variants in the SKY3576 and EAY4151 (*exo1Δ*) backgrounds. Diploid strains were induced for meiosis and % tetratype in the *CEN8-THR1* interval was measured by determining the total tetratypes/sum of tetratypes and parental ditypes. At least 500 tetrads were counted for each allele, and unless indicated (*1 transformant analyzed), at least 2 transformants were analyzed for each background. Significance was assessed by Fisher’s exact test between mutant and *wild-type EXO1* and *exo1Δ* tetratype values. To minimize *α* inflation due to multiple comparisons, we applied a Benjamini–Hochberg correction at a 5% false discovery rate; +, indistinguishable from *wild-type*; -, indistinguishable from *exo1Δ;* INT, distinguishable from both *wild-type* and *exo1Δ*. (**B**) **Spore autonomous assay: *pEXO1-RAD27* complementation of *exo1****Δ* **and *mlh3****Δ* **strains**. Diploids of the indicated genotype that contain markers to measure crossing over in the *CEN8-THR1* interval (S5 Table) were transformed with the indicated plasmids (pEAA715-*EXO1*, *URA3*, *CEN6-ARSH4;* pRS416-*URA3*,*CEN6-ARSH4;* pEAA722-*RAD27*, *URA3*, *CEN6-ARSH4;* pEAA720-*pEXO1-RAD27*, *URA3*, *CEN6-ARSH4;* pEAA724-*pEXO1-rad27-D179A*, *URA3*, *CEN6-ARSH4;* pEAA727*-rad27-A45E*, *URA3*, *CEN6-ARSH4;* pEAA728*-rad27-R101A*, *URA3*, *CEN6-ARSH4;* pEAA729*-rad27-R105A*, *URA3*, *CEN6-ARSH4;* pEAA73*0-rad27-K130A*, *URA3*, *CEN6-ARSH4;* pEAA731*-rad27-H191E*, *URA3*, *CEN6-ARSH4*) and selected for plasmid retention. The resulting strains were induced for meiosis and % tetratype (single crossovers) in the *CEN8-THR1* interval was measured by determining the total tetratypes/sum of tetratypes and parental ditypes. At least 500 tetrads were counted for each allele/plasmid combination and at least 2 transformants were analyzed for each condition. Significance (presented in Fig 5A and 5C) was assessed by Fisher’s exact test between *exo1Δ* strains containing pRS416 (empty vector) and test conditions with the indicated plasmids. To minimize *α* inflation due to multiple comparisons, we applied a Benjamini–Hochberg correction at a 5% false discovery rate. The significance of % tetratype in *exo1-K185E* and *exo1-F447A*,*F448A (MIP)* strains containing pRS416 (empty vector) and pEAA720 (*pEXO1-RAD27*) was determined using Fisher’s exact test. N/A, not applicable. (**C**) **Effect of *pHOP1-CDC9* expression on meiotic crossing over in *exo1* strains**. Diploids of the annotated genotype were transformed with the indicated plasmid (pRS426-*URA3*, *2μ;* pEAM329*-pHOP1-CDC9*, *URA3*, *2μ*) and selected for diploidy and plasmid retention. Diploid strains were induced for meiosis and % tetratype in the *CEN8-THR1* interval was measured by determining the total tetratypes/sum of tetratypes and parental ditypes. At least 500 tetrads were counted for each allele/plasmid combination and at least 2 transformants were analyzed for each condition. Significance was assessed by Fisher’s exact test between pRS426 value and pEAM329 value and is shown in Fig 7A. (**D**) **Effect of *CDC9* alleles on meiotic crossing over in the *exo1-K61E* strain**. *exo1-K61E/exo1Δ* diploids were transformed with the indicated plasmid and selected for diploidy and plasmid retention. Diploid strains were induced for meiosis and % tetratype in the *CEN8-THR1* interval was measured by determining the total tetratypes/sum of tetratypes and parental ditypes. At least 500 tetrads were counted for each and at least 2 transformants were analyzed for each condition. Significance was assessed by Fisher’s exact test between the empty vector and each plasmid containing strain. A Benjamini–Hochberg correction at a 5% false discovery rate was applied (Fig 7B).
(DOCX)

S3 Table Genetic map distances (cM) and the distribution of parental and recombinant progeny for the EAY1108/EAY1112 strain background in *WT*, *mlh3Δ*, *msh5Δ*, and *exo1* strains on Chromosome XV. Mutants are isogenic derivatives of EAY1108/EAY1112. Genetic intervals correspond to the genetic distance calculated from tetrads +/- one standard error. Standard error was calculated using the Stahl Laboratory Online Tools website (https://elizabethhousworth.com/StahlLabOnlineTools/). For single spore analysis, data are shown as 95% confidence intervals around the recombination frequency. For tetrad analysis, the centimorgan (cM) map distance was calculated using the formula of Perkins [1]: 50{TT+(6NPD)}/(PD+TT+NPD). To compare to the tetrad data, recombination frequencies obtained from single spores (Parental/(Parental+Recombinant)) were multiplied by 100 to yield genetic map distances (cM).
(PDF)

S4 Table (**A**) **Interference measurements on Chromosome XV**. The Malkova ratio and coefficient of coincidence (COC, ratio of double crossovers observed/double crossovers expected) were performed for the indicated genotypes in the EAY1108/EAY1112 strain background (Materials and methods, strains listed in S5 Table). These methods were performed for intervals I (*URA3-LEU2-LYS2)*, II (*LEU2-LYS2-ADE2)*, and III *(LYS2-ADE2-HIS3)*. 0 = Absolute Interference; 1 = No interference. Significance of differences in tetrad distribution was assessed using a G test. Differences in distribution with *p* < 0.05 were considered to be significant evidence of interference. Intervals with ratios significantly above 1 were observed and denoted with * to indicate potential negative interference. Detailed analysis of the Malkova ratio calculation is presented in S4B Table. (**B**) **Detailed calculations of Malkova ratios presented in** S4A Table **and** Fig 4C. Crossover interference was analyzed using the Malkova method [1,2] for chromosome XV. For each genetic interval, tetrads were divided based on the presence or absence of a recombination event in a reference interval. For each reference interval, the map distance was measured in the adjacent intervals, thus obtaining 2 map distances for each interval. The significance of differences in tetrad distribution was assessed using a G test. Differences in distribution with *p* < 0.05 were considered to be evidence of interference. The data are presented as the average ratio of the 2 map distances in each neighboring interval, with a smaller ratio indicating stronger interference. An interval was considered to have a “loss of positive interference” phenotype when both adjacent intervals displayed no detectable positive interference. Ratios significantly greater than 1 are indicated with * to denote potential negative interference. TT, tetratype; NPD, nonparental ditype; PD, parental ditype.
(PDF)

S5 Table Strains used in this study. (DOCX)

S6 Table Plasmids used in this study. (DOCX)

S7 Table Oligonucleotides used in this study (shown 5′ to 3′). (DOCX)

S1 File Msh5 peaks pooled from 2 *exo1Δ* replicates at 5 h time point. (PDF)

S1 Data Underlying data for Fig 2. (PDF)

S2 Data Underlying PD and TT counts in the spore autonomous fluorescence assay for Figs 3B, 5A, 5B, 7A and 7B. (XLSX)

S3 Data Underlying data for Fig 3C. Chemiluminescence (α-myc signal) and colorimetric (molecular weight standard) data are presented, as well as a composite image.
(PPTX)

S4 Data Underlying numerical data for Fig 6A. (XLSX)

S5 Data Underlying numerical data for Fig 6D, Msh5 focus counts and intensity. (XLSX)

S6 Data Underlying data for S2 Fig. Spore viability in tetrads of the indicated genotypes. (XLSX)

S7 Data Underlying data for S3 Fig. Entire YPD and YPD-MMS plates. (PDF)

## Abbreviations

COC: coefficient of coincidence
dHJ: double Holliday junction
DSB: double-strand break
MIP: Mlh1-interaction protein
MMS: methyl-methane sulfonate
SEI: single-end invasion
