# Supplementary figures and images for: Exo1 protects DNA nicks from ligation to promote crossover formation during meiosis
Source: PLoS Biol. 2023 Apr 20;21(4):e3002085. doi: 10.1371/journal.pbio.3002085 (PMC10153752; doi:10.1371/journal.pbio.3002085)

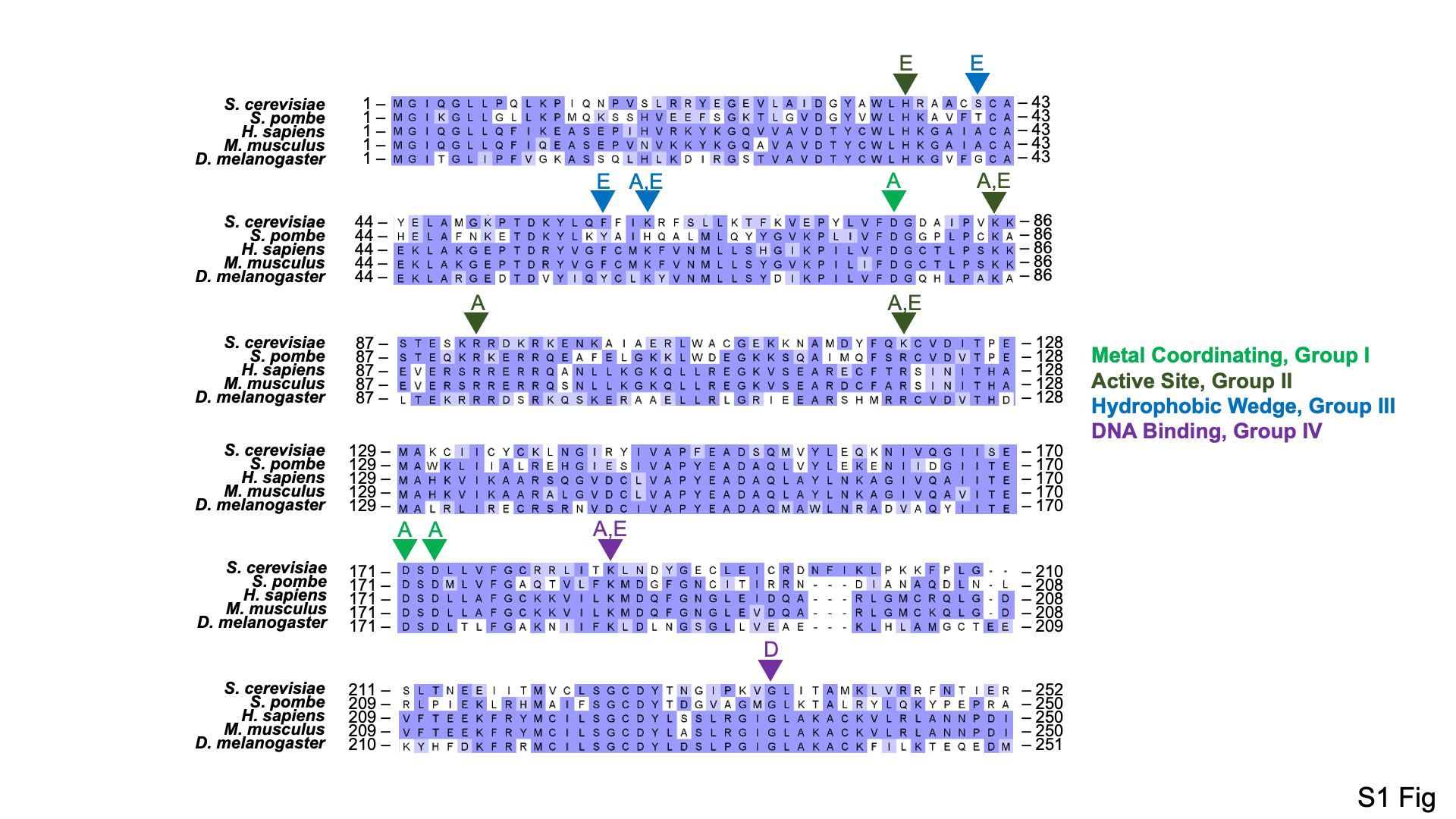

Supplement: S1 Fig — Sequence alignment of Exo1 from different species. Triangles indicate mutations made in this study. See Materials and methods for sequence alignment details. (TIFF) [file pbio.3002085.s001.tiff]

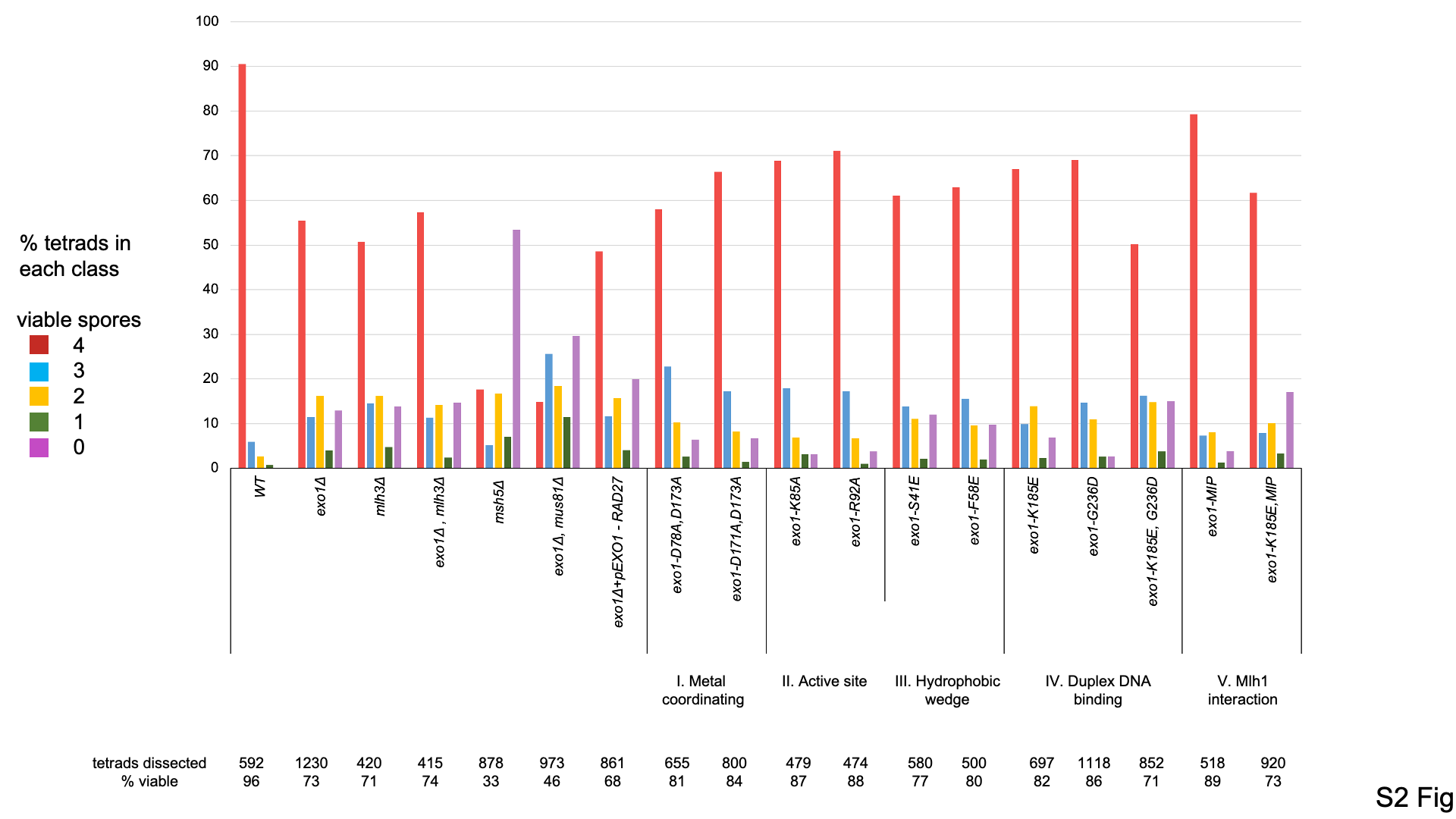

Supplement: S2 Fig — The percent of tetrads with 4, 3, 2, 1, and 0 viable spores are shown from the dissections presented in Fig 4 as well as the total number of tetrads dissected and the overall spore viability. Underlying data can be found in S6 Data. (TIFF) [file pbio.3002085.s002.tiff]

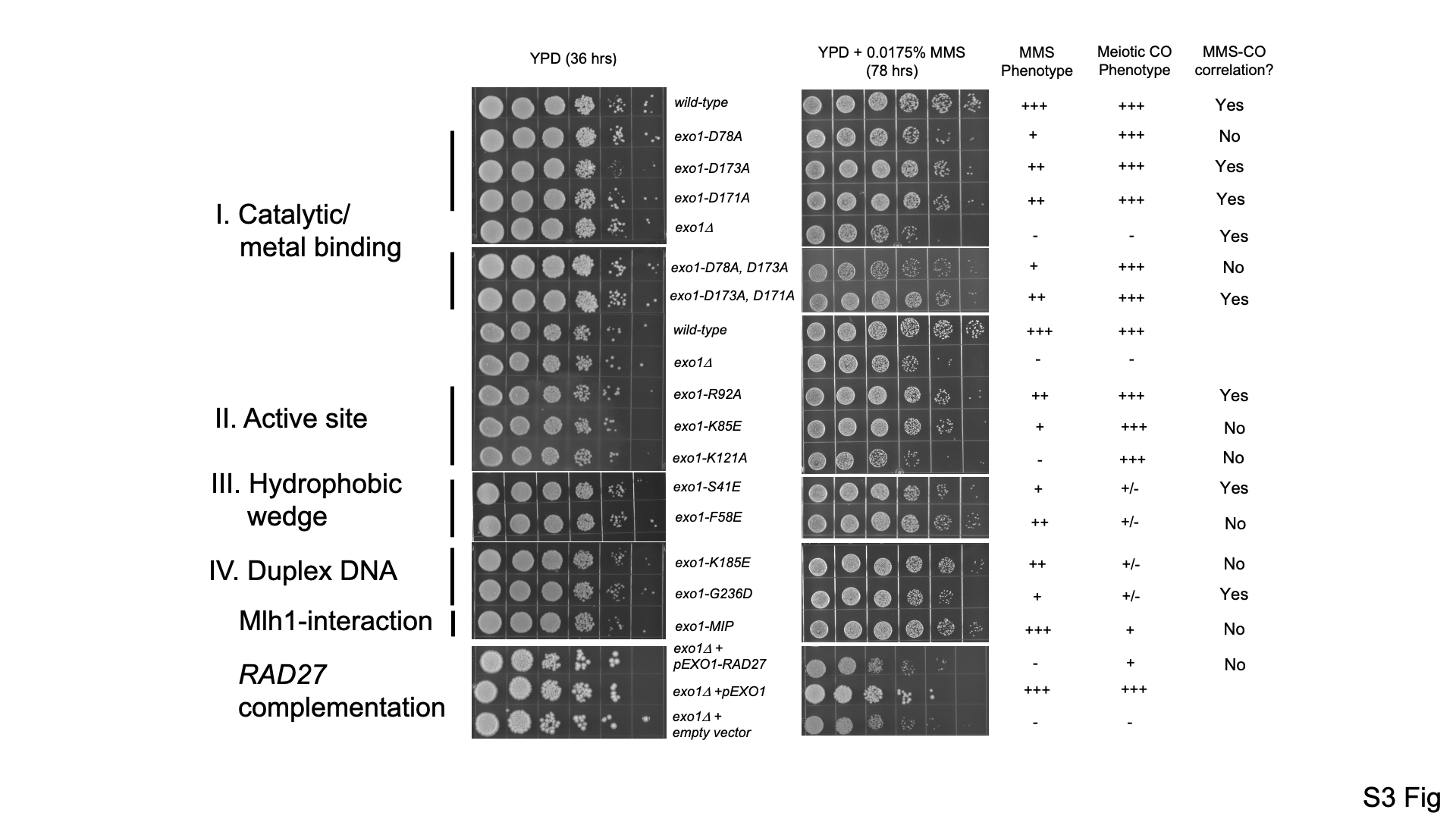

Supplement: S3 Fig — Wild-type and the indicated exo1 mutants were spotted in 10-fold serial dilutions onto YPD and YPD media containing 0.04% MMS (Materials and methods). Plates were photographed after a 2-day incubation at 30 °C. In the bottom most panel an exo1Δ strain (EAY4778) was transformed with an ARS-CEN vector containing no insert (pRS416), EXO1 (pEAA715), or RAD27 expressed from the EXO1 promoter (pEXO1-RAD27, pEAA720). Underlying data can be found in S7 Data. (TIFF) [file pbio.3002085.s003.tiff]

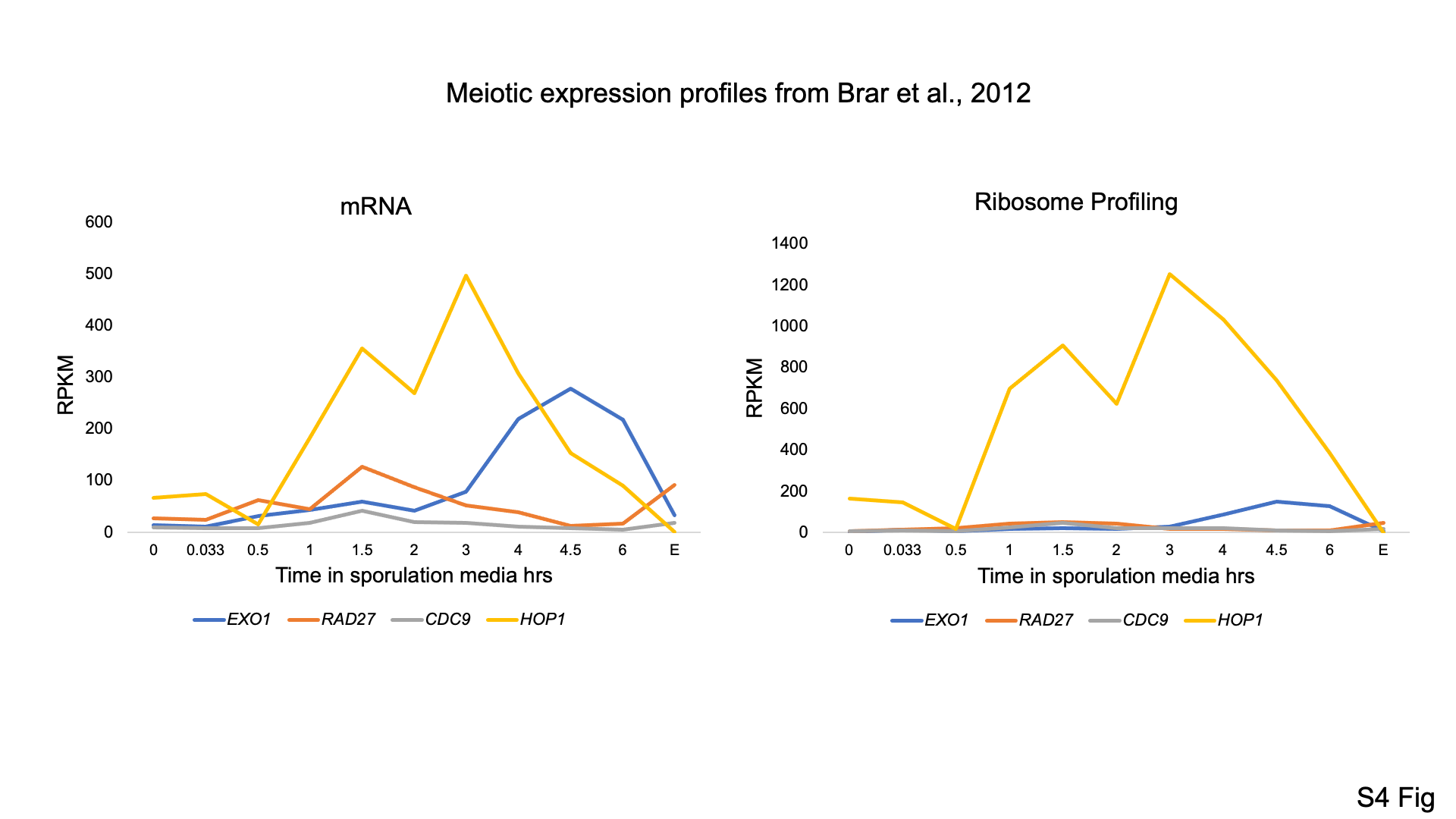

Supplement: S4 Fig — Data obtained from Brar and colleagues [74]. RPKM = Reads per kilobase of coding sequence per million mapped reads. (TIFF) [file pbio.3002085.s004.tiff]

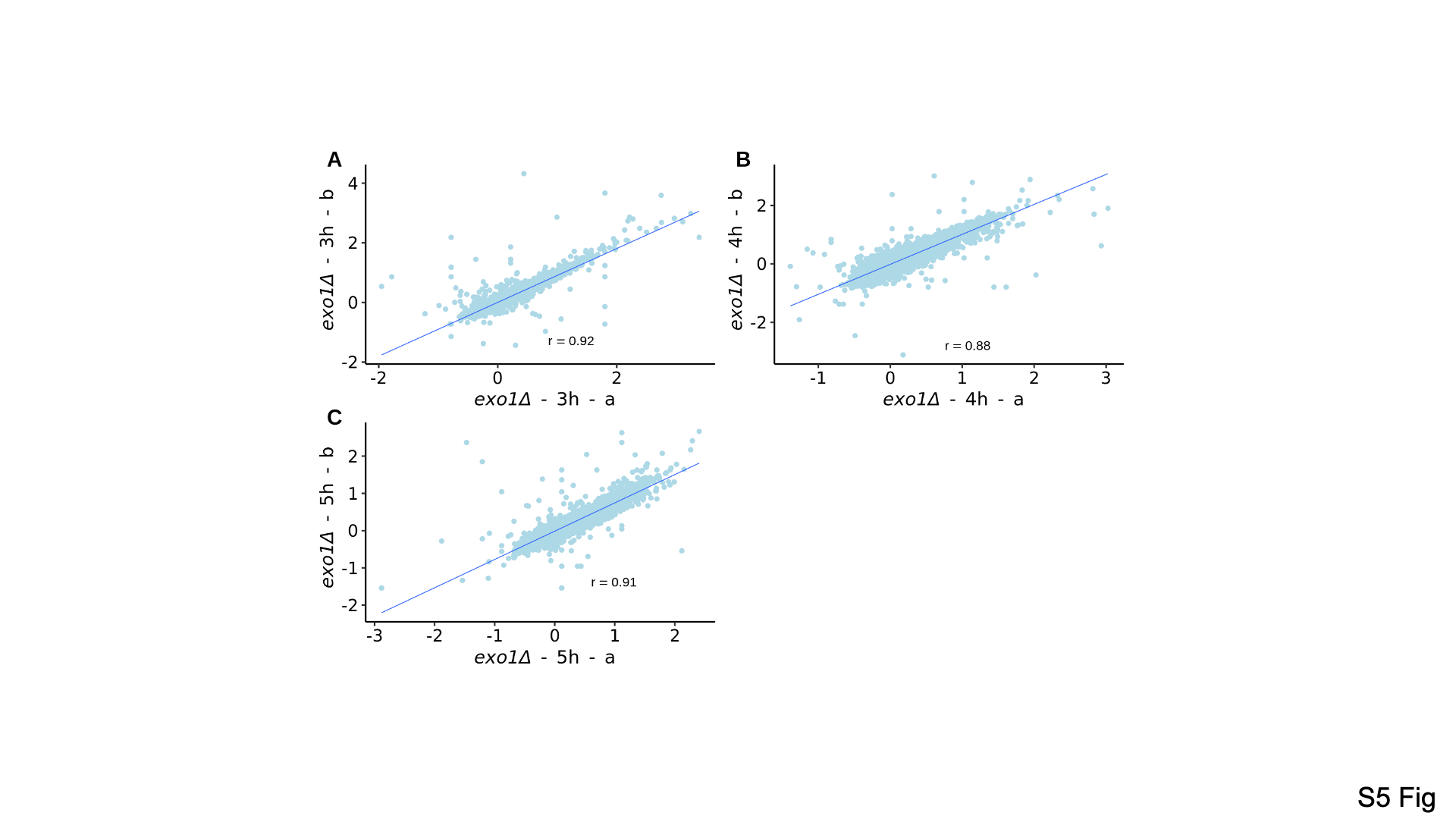

Supplement: S5 Fig — The Pearson’s correlation coefficient (r) is shown. Underlying data can be found in National Center for Biotechnology Information Sequence Read Archive, accession number PRJNA780068. (TIFF) [file pbio.3002085.s005.tiff]
