# Supplementary material for: Exo1 protects DNA nicks from ligation to promote crossover formation during meiosis
Source: PLoS Biol. 2023 Apr 20;21(4):e3002085. doi: 10.1371/journal.pbio.3002085 (PMC10153752; doi:10.1371/journal.pbio.3002085)
Supplement: S6 Fig — (A) Boxplot comparing differences in average Msh5 reads in wild-type and exo1Δ mutant at overlapping Msh5 peak locations. Msh5 read counts were obtained from the Msh5 ChIP-Seq experiment presented in Fig 6B. Y axis shows the average of Msh5 read counts +/- 100 bp from the center of each peak (5 h) in wild-type and exo1Δ, and p value was calculated using Wilcoxon rank sum test and adjusted using Bonferroni correction, and *** indicates p values <0.001. (B) Zoomed-in region of the YCR093W cold spot ([88]; Fig 6B) showing very low Msh5 reads (unsmoothed) in both wild-type and exo1Δ. (C) Msh5 binding was compared in wild-type and exo1Δ at 25 cold spots [87,88] that were depleted for Msh5 in wild-type. These 25 were obtained by rank ordering 49 cold spots in Gerton and colleagues [87] and Shodhan and colleagues [88] based on Msh5 read counts in wild-type. The lowest 25 were then analyzed. The Y axis shows the Msh5 read count (unsmoothed) for wild-type and exo1Δ at 3, 4, and 5 h post-meiotic induction. The X axis indicates +/- 1 kb from the cold spot center. The average number of Msh5 read counts in wild-type (WT) and exo1Δ is presented for each time point (+/- 100 bp from cold spot center). (D) List of 49 cold spots in Gerton and colleagues [87] and Shodhan and colleagues [88] (left panel), 25 of which (right panel) were analyzed in this study and are presented in order from highest (HXT1) to lowest (YGR289C) Msh5 counts. Underlying data for S6 Fig can be found in National Center for Biotechnology Information Sequence Read Archive, accession number PRJNA780068. (PDF) [file pbio.3002085.s006.pdf]

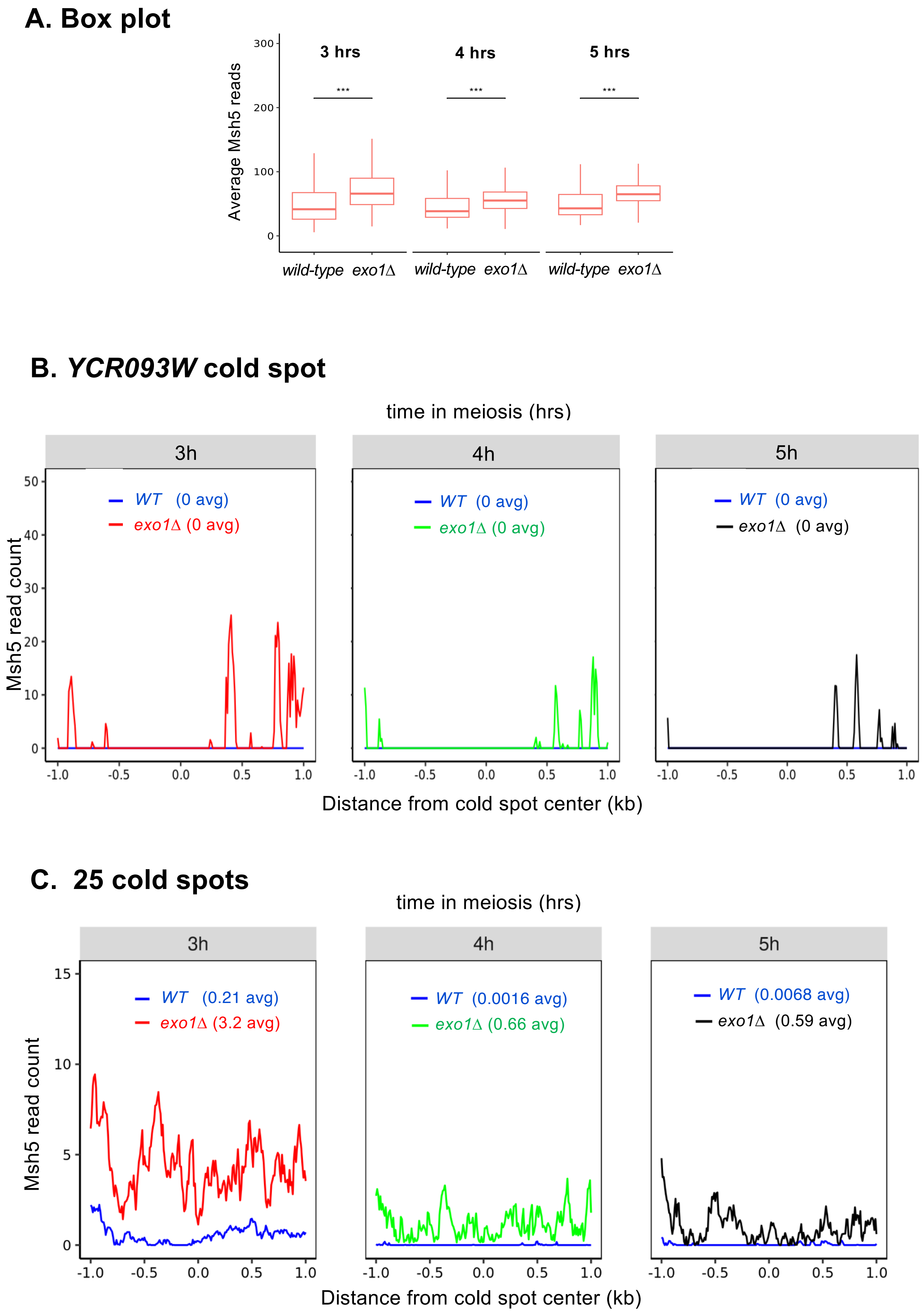
A. Box plot
Average Msh5 reads
300
200
100
0
3 hrs
4 hrs
5 hrs
***
wild-type exo1Δ wild-type exo1Δ wild-type exo1Δ
B. YCR093W cold spot
time in meiosis (hrs)
3h
4h
5h
WT (0 avg)
exo1Δ (0 avg)
Msh5 read count
50
40
30
20
10
0
-1.0 -0.5 0.0 0.5 1.0
Distance from cold spot center (kb)
C. 25 cold spots
time in meiosis (hrs)
3h
4h
5h
WT (0.21 avg)
exo1Δ (3.2 avg)
WT (0.0016 avg)
exo1Δ (0.66 avg)
WT (0.0068 avg)
exo1Δ (0.59 avg)
Msh5 read count
15
10
5
0
-1.0 -0.5 0.0 0.5 1.0
Distance from cold spot center (kb)

S6 Fig

D. Lists of DSB colds spots

Chromosomal coordinates of 49 DSB cold spots [86,87]

Loci	Chr	Start	End
YAR002W	1	152257	153876
YBR001C	2	238941	241283
YBR294W	2	789235	791814
YBR298C	2	802631	804475
YCL004W	3	109105	110670
YDL029W	4	399340	400638
YDR028C	4	497835	500879
YDR324C	4	1114433	1116763
YDR467C	4	1397583	1397909
YDR468C	4	1398026	1398700
YER060W	5	274567	276153
YGL257C	7	12481	14157
YGL186C	7	151037	152776
YGL132W	7	261580	261915
YGR008C	7	508110	508364
YGR009C	7	509047	511002
YGR143W	7	775193	777508
YGR289C	7	1073963	1075813
YHR111W	8	333072	334394
YIL067C	9	235724	237760
YIR023W	9	399777	402689
YIR028W	9	408468	410375
YIR029W	9	410807	411838
YJL214W	10	26887	28596
YJL001W	10	435163	435926
YJR001W	10	436802	438610
YJR004C	10	442909	444861
YJR032W	10	491081	492262
YJR091C	10	595061	598336
YKL169C	11	130685	131068
YKL170W	11	130634	131050
YKR101W	11	640540	642504
YKR102W	11	646356	649865
YLR005W	12	160049	161434
YMR154C	13	565999	568182
YNL233W	14	211922	214600
YNL235C	14	209548	209979
YNL237W	14	205188	206567
YNL238W	14	202428	204872
YNL183C	14	293137	295509
YNR070W	14	765375	769376
YOL147C	15	47933	48643
YOR003W	15	331455	332891
YOR008C	15	341278	342414
YOR022C	15	373710	375857
YOR130C	15	569929	570807
URA3	5	116167	116970
HXT1	8	290913	292625
YCR017C	3	144773	147634

URA3, HXT1, and YCR017C are from Shodhan et al. [87}; the other 46 are from Gerton et al. [86].

Chromosomal coordinates of 25 DSB cold spots analyzed in S6B Fig.

Chr	Position	Start	End	Loci
chrVIII	291770	290913	292625	*HXT1*
chrIII	109888	109105	110670	*YCL004W*
chrXV	374784	373710	375857	*YOR022C*
chrIX	411323	410807	411838	*YIR029W*
chrIX	236743	235724	237760	*YIL067C*
chrII	240113	238941	241283	*YBR001C*
chrXIV	294324	293137	295509	*YNL183C*
chrXV	48289	47933	48643	*YOL147C*
chrXI	648111	646356	649865	*YKR102W*
chrXIV	213262	211922	214600	*YNL233W*
chrIX	401234	399777	402689	*YIR023W*
chrVII	13320	12481	14157	*YGL257C*
chrXI	641523	640540	642504	*YKR101W*
chrVII	510025	509047	511002	*YGR009C*
chrII	790525	789235	791814	*YBR294W*
chrX	443886	442909	444861	*YJR004C*
chrXIV	203651	202428	204872	*YNL238W*
chrIII	146204	144773	147634	*YCR017C*
chrIV	1115599	1114433	1116763	*YDR324C*
chrIV	499358	497835	500879	*YDR028C*
chrVII	151907	151037	152776	*YGL186C*
chrXIV	767376	765375	769376	*YNR070W*
chrVII	776351	775193	777508	*YGR143W*
chrXIII	567091	565999	568182	*YMR154C*
chrVII	1074889	1073963	1075813	*YGR289C*
