# Supplementary material for: Exo1 protects DNA nicks from ligation to promote crossover formation during meiosis
Source: PLoS Biol. 2023 Apr 20;21(4):e3002085. doi: 10.1371/journal.pbio.3002085 (PMC10153752; doi:10.1371/journal.pbio.3002085)
Supplement: S1 Table — (PDF) [file pbio.3002085.s007.pdf]

S1 Table. Structure function analysis of XPG family proteins.

Mutant protein analyzed, citation	Domain location of mutated residue	Nuclease activity	DNA binding activity	*S. cerevisiae* EXO1 structural alignment with mutation analyzed in this study
**Human EXO1, Orans et al. [1]**				
EXO1-D173A (metal binding, Group I)	Active site metal coordinating	severely defective	not characterized	**D173A**
EXO1-Y32A (active site, Group II)	Gateway fraying residue	20-fold loss in catalytic activity	not characterized	Y32
EXO1-H36A (active site, Group II)	Gateway fraying residue	150-fold loss catalytic activity	not characterized	**H36A**
EXO1-R92A (active site, Group II)	Active site DNA interactor	severely defective	not characterized	**R92A**
EXO1-K85A (active site, Group II)	Active site DNA interactor	severely defective	not characterized	**K85A**
**Human EXO1, Lee et al. [2]**				
EXO1-D78A (metal binding, Group I)	Active site metal coordinating	206-fold reduction on dsDNA, 1035-fold reduction on flap cleavage	5-fold lower DNA flap binding	**D78A**
EXO1-D173A (metal binding, Group I)	Active site metal coordinating	56-fold reduction on dsDNA, 669-fold reduction on flap cleavage	wild-type DNA binding affinity	**D173A**
EXO1-D225A (metal binding, Group I)	Active site metal coordinating	97-fold reduction on dsDNA, 380-fold reduction on flap cleavage	5-fold higher DNA flap binding	D227
***S. cerevisiae* EXO1, Tran et al. [3] ; Amin et al. [4]**				
EXO1-D173A (metal binding Group I)	Active site metal coordinating	Severely defective in flap cleavage	not characterized	**D173A**
EXO1-G236D (DNA binding, Group IV)	Mutation in helix-two-turn-helix motif	Genetic phenotype-disrupts Exo1-dependent DNA mismatch repair	predicted to disrupt DNA binding	**G236D**
***S. cerevisiae* EXO1, Li et al. [5]**				
EXO1-K185A (DNA binding, Group IV)	Conserved duplex DNA recognition	Reduction in exonuclease activity on duplex DNA	not characterized	**K185A**
**Human FEN1, Tsutakawa et al. [6]**				
FEN1-D181A (metal binding, Group I)	Active site metal coordinating	>800-fold reduction in flap cleavage	not characterized	**D173A**
FEN1-Y40A (active site, Group II)	Gateway fraying residue	20-fold reduction in flap cleavage	not characterized	**H36A**
FEN1-K93A (active site, Group II)	Gateway DNA interactor	>400-fold reduction in flap cleavage	not characterized	**K85A**
FEN1-R100A (active site, Group II)	Gateway DNA interactor	>400-fold reduction in flap cleavage	not characterized	**R92A**
FEN1-R104A (active site, Group II)	Gateway DNA interactor	3-fold reduction in flap cleavage	not characterized	R96
FEN1-R129A (active site, Group II)	Gateway/cap DNA interactor	1.5-fold reduction in flap cleavage	not characterized	Q120
FEN1-R47A (hydrophobic wedge, Group III)	Hydrophobic wedge	30-fold reduction in flap cleavage	not characterized	C42
**Human FEN1, Tsutakawa et al. [7]**				
FEN1-R103A (active site, Group II)	Gateway DNA interactor	3-fold reduction in flap cleavage	not characterized	R95
FEN1-R104A (active site, Group II)	Gateway DNA interactor	20-fold reduction in flap cleavage	not characterized	R96
FEN1-R129A (active site, Group II)	Gateway/cap DNA interactor	20-fold reduction in flap cleavage	not characterized	Q120
FEN1-K132A (active site, Group II)	Cap phosphate steering/DNA interactor	5-fold reduction in flap cleavage	not characterized	V123
**Human FEN1, Song et al. [8]**				
FEN1-D34A (metal binding, Group I)	Catalytic metal coordinating	catalytic activity too low to measure	retains wild-type DNA binding/bending	D30
FEN1-D181A (metal binding, Group I)	Catalytic metal coordinating	catalytic activity too low to measure	retains wild-type DNA binding/bending	**D173A**
FEN1-Y40A (active site, Group II)	Gateway fraying residue	80-fold reduction in flap cleavage	retains wild-type DNA binding/bending	**H36A**
FEN1-K93A (active site, Group II)	Gateway DNA interactor	catalytic activity too low to measure	retains wild-type DNA binding/bending	**K85A**
FEN1-R100A (active site, Group II)	Gateway DNA interactor	catalytic activity too low to measure	retains wild-type DNA binding/bending	**R92A**
***S. cerevisiae* RAD2, Mietus et al. [9]**				
RAD2-Y36A (active site, Group II)	Gateway DNA interactor	wild-type catalytic activity	not characterized	**H36A**
RAD2-Q37A (active site, Group II)	Gateway DNA interactor	catalytically dead	not characterized	R37
RAD2-R60A (hydrophobic wedge, Group III)	Hydrophobic wedge	~2 fold loss in catalytic activity	not characterized	I60
RAD2-R61A (hydrophobic wedge, Group III)	Hydrophobic wedge	catalytic activity too low to measure	not characterized	**K61A**
**Human XPG, Gonzalez-Corrochano et al. [10]**				
XPG-D812A (metal binding, Group I)	Catalytic metal coordinating	catalytic activity too low to measure	wild-type DNA binding	**D173A**
XPG-K84A (active site, Group II)	Gateway DNA interactor	catalytic activity too low to measure	mild decrease in DNA binding	**K85A**
XPG-R91A (active site, Group II)	Gateway DNA interactor	~2 fold loss of catalytic activity	wild-type DNA binding	**R92A**
XPG-R92A (active site, Group II)	Gateway DNA interactor	~2 fold loss of catalytic activity	~2 fold decrease in binding	R93
XPG-R43A/R45A (hydrophobic wedge, Group III)	Hydrophobic wedge	~2 fold loss of catalytic activity	mild decrease in DNA binding	A43/K45
XPG-H60A (hydrophobic wedge, Group III)	Hydrophobic wedge	Mild decrease in catalytic activity	wild-type DNA binding	I60
XPG-R823A (DNA binding, Group IV)	B-pin residue	Wild-type catalytic activity	mild decrease in DNA binding	T184
XPG-K828E (DNA binding, Group IV)	B-pin residue	Wild-type catalytic activity	wild-type DNA binding	Y189

References

1. Orans J, McSweeney EA, Iyer RR, Hast MA, Hellinga HW, Modrich P, Beese LS. Structures of human exonuclease I DNA complexes suggest a unified mechanism for nuclease family. Cell. 2011;145: 212–223.

2. Lee B-I, Nguyen LH, Barsky D, Fernandes M, Wilson DM 3rd. Molecular interactions of human Exo1 with DNA. Nucleic Acids Res. 2002;30: 942-949.

3. Tran PT, Erdeniz N, Dudley S, Liskay RM. Characterization of nuclease-dependent functions of Exo1p in *Saccharomyces cerevisiae*. DNA Repair. 2002;1: 895-912.

4. Amin NS, Nguyen M, Oh S, Kolodner RD. *exo1*-dependent mutator mutation: Model system for studying functional interactions in mismatch repair. Mol Cell Biol. 2001;21: 5142–5155.

5. Li Y, Shen J, Niu H. DNA duplex recognition activates Exo1 nuclease activity. J Biol Chem. 2019;294: 11559–11567.

6. Tsutakawa SE, Classen S, Chapados BR, Arvai AS, Finger L, Guenther G, et al. Human flap endonuclease structures, DNA double-base flipping, and a unified understanding of the FEN1 superfamily. Cell. 2011;145: 198-211.

7. Tsutakawa SE, Thompson MJ, Arvai AS, Neil AJ, Shaw SJ, Algasaier SI, et al. Phosphate steering by Flap Endonuclease 1 promotes 5’-flap specificity and incision to prevent genome instability. Nat Commun. 2017;8: 15855. doi: 10.1038/ncomms15855.

8. Song B, Hamdan SM, Hingorani MM. Positioning the 5’-flap junction in the active site controls the rate of flap endonuclease 1–catalyzed DNA cleavage. J Biol Chem. 2018;293: 4792–4804.

9. Mietus M, Nowak E, Jaciuk M, Kustosz P, Studnicka J, Nowotny, M. Crystal structure of the catalytic core of Rad2: insights into the mechanism of substrate binding. Nucleic Acids Res. 2014;42: 10762-10775.

10. González-Corrochano R, Ruiz FM, Taylor NMI, Huecas S, Drakulic S, Spinola-Amilibia M, Fernandez-Tornero C. The crystal structure of human XPG, the xeroderma pigmentosum group G endonuclease, provides insight into nucleotide excision DNA repair. Nucleic Acids Res. 2020;48: 9943-9958.
