# Supplementary material for: Exo1 protects DNA nicks from ligation to promote crossover formation during meiosis
Source: PLoS Biol. 2023 Apr 20;21(4):e3002085. doi: 10.1371/journal.pbio.3002085 (PMC10153752; doi:10.1371/journal.pbio.3002085)
Supplement: S2 Table — (A) Spore autonomous meiotic crossover analysis of exo1 mutants. Homozygous mutations were made by crossing 2 independently constructed strains with the exo1 variants in the SKY3576 (containing cyan fluorescent protein; S5 Table) and SKY3575 (containing red fluorescent protein) backgrounds. Heterozygous mutations were made by crossing 2 independently constructed strains with exo1 variants in the SKY3576 and EAY4151 (exo1Δ) backgrounds. Diploid strains were induced for meiosis and % tetratype in the CEN8-THR1 interval was measured by determining the total tetratypes/sum of tetratypes and parental ditypes. At least 500 tetrads were counted for each allele, and unless indicated (*1 transformant analyzed), at least 2 transformants were analyzed for each background. Significance was assessed by Fisher’s exact test between mutant and wild-type EXO1 and exo1Δ tetratype values. To minimize α inflation due to multiple comparisons, we applied a Benjamini–Hochberg correction at a 5% false discovery rate; +, indistinguishable from wild-type; -, indistinguishable from exo1Δ; INT, distinguishable from both wild-type and exo1Δ. (B) Spore autonomous assay: pEXO1-RAD27 complementation of exo1Δ and mlh3Δ strains. Diploids of the indicated genotype that contain markers to measure crossing over in the CEN8-THR1 interval (S5 Table) were transformed with the indicated plasmids (pEAA715-EXO1, URA3, CEN6-ARSH4; pRS416-URA3,CEN6-ARSH4; pEAA722-RAD27, URA3, CEN6-ARSH4; pEAA720-pEXO1-RAD27, URA3, CEN6-ARSH4; pEAA724-pEXO1-rad27-D179A, URA3, CEN6-ARSH4; pEAA727-rad27-A45E, URA3, CEN6-ARSH4; pEAA728-rad27-R101A, URA3, CEN6-ARSH4; pEAA729-rad27-R105A, URA3, CEN6-ARSH4; pEAA730-rad27-K130A, URA3, CEN6-ARSH4; pEAA731-rad27-H191E, URA3, CEN6-ARSH4) and selected for plasmid retention. The resulting strains were induced for meiosis and % tetratype (single crossovers) in the CEN8-THR1 interval was measured by determining the total tetratypes/sum of tetratypes and parental ditypes. At least 500 tetrads [file pbio.3002085.s008.docx]

| **S2A Table. Spore Autonomous Meiotic Crossover Analysis of *exo1* mutants.** | | | |
| --- | --- | --- | --- |
| **Allele** | **%Tetratype** | **Tetrads Counted** | **Phenotype** |
| *EXO1/EXO1* | 39.0 | 1071 | + |
| *EXO1/exo1Δ* | 37.9 | 1071 | + |
| *EXO1-MYC13/exo1Δ* | 37.2 | 508 | + |
| *exo1Δ/exo1Δ* | 20.0 | 1054 | - |
| *rad27Δ/rad27Δ* | 38.6 | 580 | + |
| ***Metal Binding (Group I)*** |  |  |  |
| *exo1-D171A/exo1Δ* | 38.9 | 517 | + |
| *exo1-D171A,D173A/exo1Δ* | 39.1 | 511 | + |
| *exo1-D78A/exo1-D78A* | 39.7 | 531 | + |
| *exo1-D173A/exo1-D173A* | 37.4 | 519 | + |
| *exo1-D78A,D173A/ exo1-D78A,D173A* | 36.4 | 544 | + |
| ***Active Site DNA Interactors (Group II)*** |  |  |  |
| *exo1-H36E/exo1Δ* | 35.4 | 506 | + |
| *exo1-K85A/exo1Δ* | 34.8 | 526 | + |
| *exo1-K85E/exo1Δ* | 34.5 | 533 | + |
| *exo1-R92A/exo1Δ* | 34.8 | 506 | + |
| *exo1-K121A/exo1Δ* | 37.7 | 605 | + |
| *exo1-K121E/exo1Δ* | 34.5 | 765 | + |
| ***Hydrophobic Wedge (Group III)*** |  |  |  |
| *exo1-S41E/exo1Δ* | 28.4 | 506 | INT |
| *exo1-F58E/exo1Δ* | 27.8 | 507 | INT |
| *exo1-K61A/exo1Δ* | 35.1 | 525 | + |
| *exo1-K61E/exo1Δ* | 35.1 | 507 | + |
| *exo1-S41E,F58E/exo1Δ* | 24.6 | 504 | INT |
| *exo1-S41E,K61E/exo1Δ* | 24.5 | 506 | - |
| ***Duplex DNA Interactors (Group IV)*** |  |  |  |
| *exo1-K185A/exo1Δ* | 35.4 | 720 | + |
| *exo1-K185E/exo1Δ* | 24.5 | 649 | INT |
| *exo1-G236D/exo1-G236D* | 29.9 | 521 | INT |
| *exo1-G236D/exo1Δ* | 29.1 | 515 | INT |
| *exo1-G236D,K185E/exo1Δ* | 24.2 | 508 | - |
| ***Mlh1-Interacting (MIP, Group V)*** |  |  |  |
| *exo1- F447A,F448A/exo1-F447A,F448A* | 33.3 | 547 | INT |
| *exo1- F447A,F448A/exo1Δ* | 26.2 | 519 | INT |
| ***Double and Triple Mutants*** |  |  |  |
| *exo1-D171A,G236D/exo1Δ* | 31.1 | 552 | INT |
| *exo1-D173A,G236D/exo1Δ* | 32.7 | 618 | + |
| *exo1-D173A,G236D/ exo1-D173A,G236D* | 35.7 | 532 | + |
| *exo1-D173A,K185E,G236D/exo1Δ* | 22.4 | 553 | - |
| *exo1-G236D,F447A,F448A/exo1Δ* | 25.1 | 617 | INT |
| *exo1-K185E,F447A,F448A/exo1Δ* | 24.8 | 572 | INT |
| *exo1-D173A,G236D,F447A,F448A/exo1Δ* | 26.6 | 500 | INT |
| *exo1-R92A,K121A,K185A/exo1Δ* | 24.3 | 535 | - |

Homozygous mutations were made by crossing two independently constructed strains with the *exo1* variants in the SKY3576 (containing cyan fluorescent protein; S5 Table) and SKY3575 (containing red fluorescent protein) backgrounds. Heterozygous mutations were made by crossing two independently constructed strains with *exo1* variants in the SKY3576 and EAY4151 (*exo1Δ*) backgrounds. Diploid strains were induced for meiosis and % tetratype in the *CEN8-THR1* interval was measured, by determining the total tetratypes/sum of tetratypes and parental ditypes). At least 500 tetrads were counted for each allele, and unless indicated (*one transformant analyzed), at least two transformants were analyzed for each background. Significance was assessed by Fisher’s exact test between mutant and *wild-type* *EXO1 and exo1Δ* tetratype values. To minimize 𝜶 inflation due to multiple comparisons, we applied a Benjamini-Hochberg correction at a 5% false discovery rate. +, indistinguishable from *wild-type*; -, indistinguishable from *exo1Δ;* INT, distinguishable from both *wild-type* and *exo1Δ*.

**S2B Table. Spore autonomous assay: *pEXO1-RAD27* complementation of *exo1Δ* and *mlh3Δ* strains**

| **Genotype** | **Plasmid** | **%Tetratype** | **Tetrads Counted** | **Phenotype** |
| --- | --- | --- | --- | --- |
| *exo1Δ/exo1Δ* | *EXO1* | 34.1 | \| 557 \| \| --- \| | + |
| *exo1Δ/exo1Δ* | *empty vector* | 21.5 | 512 | - |
| *exo1Δ/exo1Δ* | *RAD27* | 22.6 | 1032 | - |
| *exo1Δ/exo1Δ* | *pEXO1-RAD27* | 29.9 | 521 | + |
| *exo1Δ/exo1Δ* | *pEXO1-rad27-D179A* | 28.8 | 510 | + |
| *exo1Δ/exo1Δ* | *pEXO1-rad27-A45E* | 22.4 | 511 | - |
| *exo1Δ/exo1Δ* | *pEXO1-rad27-R101A* | 29.7 | 542 | + |
| *exo1Δ/exo1Δ* | *pEXO1-rad27-R105A* | 28.7 | 521 | + |
| *exo1Δ/exo1Δ* | *pEXO1-rad27-K130A* | 28.9 | 505 | + |
| *exo1Δ/exo1Δ* | *pEXO1-rad27-H191E* | 24.0 | 530 | - |
| *mlh3Δ/mlh3Δ* | *MLH3* | 35.6 | 508 | + |
| *mlh3Δ/mlh3Δ* | *empty vector* | 22.5 | 528 | - |
| *mlh3Δ/mlh3Δ* | *pEXO1-RAD27* | 21.5 | 512 | - |
| *mlh3Δ/mlh3Δ* | *pEXO1-rad27-D179A* | 19.9 | 513 | - |
| *exo1-K185E/exo1Δ* | *empty vector* | 25.4 | 1538 | N/A |
| *exo1-K185E/exo1Δ* | *pEXO1-RAD27* | 29.0 | 1541 | N/A |
| *exo1-F447A,F448A/exo1Δ* | *empty vector* | 30.1 | 512 | N/A |
| *exo1-F447A,F448A/exo1Δ* | *pEXO1-RAD27* | 29.7 | 526 | N/A |

Diploids of the indicated genotype that contain markers to measure crossing over in the *CEN8-THR1* interval (S5 Table) were transformed with the indicated plasmids (pEAA715-*EXO1, URA3, CEN6-ARSH4;* pRS416-*URA3,CEN6-ARSH4;* pEAA722-*RAD27*, *URA3, CEN6-ARSH4;* pEAA720-*pEXO1-RAD27, URA3, CEN6-ARSH4;* pEAA724-*pEXO1-rad27-D179A, URA3, CEN6-ARSH4;* pEAA727*-rad27-A45E, URA3, CEN6-ARSH4;* pEAA728*-rad27-R101A, URA3, CEN6-ARSH4;* pEAA729*-rad27-R105A, URA3, CEN6-ARSH4;* pEAA730*-rad27-K130A, URA3, CEN6-ARSH4;* pEAA731*-rad27-H191E, URA3, CEN6-ARSH4*) and selected for plasmid retention. The resulting strains were induced for meiosis and % tetratype (single crossovers) in the *CEN8-THR1* interval was measured, by determining the total tetratypes/sum of tetratypes and parental ditypes. At least 500 tetrads were counted for each allele/plasmid combination, and at least two transformants were analyzed for each condition. Significance (presented in Figure 5A, C) was assessed by Fisher’s Exact Test between *exo1Δ* strains containing pRS416 (empty vector) and test conditions with the indicated plasmids. To minimize 𝜶 inflation due to multiple comparisons, we applied a Benjamini-Hochberg correction at a 5% false discovery rate. The significance of % tetratype in *exo1-K185E* and *exo1-F447A,F448A (MIP)* strains containing pRS416 (empty vector) and pEAA720 (*pEXO1-RAD27*) was determined using Fisher’s exact test. N/A, not applicable.

**S2C Table. Effect of *pHOP1-CDC9* expression on meiotic crossing over in *exo1* strains.**

| **Genotype** | **Plasmid** | **%Tetratype** | **Tetrads Counted** |
| --- | --- | --- | --- |
| *EXO1/exo1Δ* | *empty vector* | 41.3 | \| 520 \| \| --- \| |
| *EXO1/exo1Δ* | *pHOP1-CDC9* | 41.0 | 528 |
| *exo1Δ/exo1Δ* | *empty vector* | 21.6 | 519 |
| *exo1Δ/exo1Δ* | *pHOP1-CDC9* | 22.2 | 543 |
| *exo1-MIP/exo1Δ* | *empty vector* | 30.1 | 512 |
| *exo1-MIP/exo1Δ* | *pHOP1-CDC9* | 30.2 | 540 |
| *exo1-K61E/exo1Δ* | *empty vector* | 35.1 | 521 |
| *exo1-K61E/exo1Δ* | *pHOP1-CDC9* | 25.2 | 514 |
| *exo1-K85E/exo1Δ* | *empty vector* | 36.2 | 1529 |
| *exo1-K85E/exo1Δ* | *pHOP1-CDC9* | 33.3 | 1530 |
| *exo1-K185A/exo1Δ* | *empty vector* | 35.3 | 1536 |
| *exo1-K185A/exo1Δ* | *pHOP1-CDC9* | 31.3 | 1583 |
| *exo1-D173A/exo1Δ* | *empty vector* | 38.9 | 501 |
| *exo1-D173A/exo1Δ* | *pHOP1-CDC9* | 38.5 | 509 |

Diploids of the annotated genotype were transformed with the indicated plasmid (pRS426-*URA3, 2μ*; pEAM329*-pHOP1-CDC9, URA3, 2μ*) and selected for diploidy and plasmid retention. Diploid strains were induced for meiosis and % Tetratype in the *CEN8-THR1* interval was measured by determining the total tetratypes/sum of tetratypes and parental ditypes. At least 500 tetrads were counted for each allele/plasmid combination, and at least two transformants were analyzed for each condition. Significance was assessed by Fisher’s exact test between pRS426 value and pEAM329 value and is shown in Figure 7A.

**S2D Table. Effect of *CDC9* alleles on meiotic crossing over in the *exo1-K61E* strain.**

| **Genotype** | **Plasmid** | **%Tetratype** | **Tetrads Counted** |
| --- | --- | --- | --- |
| *exo1-K61E/exo1Δ* | *empty vector* | 37.0 | \| 500 \| \| --- \| |
| *exo1-K61E/exo1Δ* | *pHOP1-CDC9* | 26.0 | 511 |
| *exo1-K61E/exo1Δ* | *pHOP1-cdc9-F44A,F45A* | 27.1 | 510 |
| *exo1-K61E/exo1Δ* | *pHOP1-cdc9-K419A* | 34.7 | 504 |

*exo1-K61E/exo1Δ* diploids were transformed with the indicated plasmid and selected for diploidy and plasmid retention. Diploid strains were induced for meiosis and % Tetratype in the *CEN8-THR1* interval was measured by determining the total tetratypes/sum of tetratypes and parental ditypes. At least 500 tetrads were counted for each and at least two transformants were analyzed for each condition. Significance was assessed by Fisher’s exact test between the empty vector and each plasmid containing strain. A Benjamini-Hochberg correction at a 5% false discovery rate was applied (Figure 7B).
