# Supplementary material for: Exo1 protects DNA nicks from ligation to promote crossover formation during meiosis
Source: PLoS Biol. 2023 Apr 20;21(4):e3002085. doi: 10.1371/journal.pbio.3002085 (PMC10153752; doi:10.1371/journal.pbio.3002085)
Supplement: S3 Table — Mutants are isogenic derivatives of EAY1108/EAY1112. Genetic intervals correspond to the genetic distance calculated from tetrads +/- one standard error. Standard error was calculated using the Stahl Laboratory Online Tools website (https://elizabethhousworth.com/StahlLabOnlineTools/). For single spore analysis, data are shown as 95% confidence intervals around the recombination frequency. For tetrad analysis, the centimorgan (cM) map distance was calculated using the formula of Perkins [1]: 50{TT+(6NPD)}/(PD+TT+NPD). To compare to the tetrad data, recombination frequencies obtained from single spores (Parental/(Parental+Recombinant)) were multiplied by 100 to yield genetic map distances (cM). (PDF) [file pbio.3002085.s009.pdf]

S3 Table. Genetic map distances (cM) and the distribution of parental and recombinant progeny for the EAY1108/EAY1112 strain background in *WT, mlh3Δ, msh5Δ,* and *exo1* strains on Chromosome XV.

	Tetrads					Single spores			
Relevant genotype	Number analyzed	cM	PD	TT	NPD	Number analyzed	cM (95% CI)	Parental	Recombinant
URA3-LEU2:									
wild-type	501	22.4+/-1.5	292	206	3	2285	21.5 (19.8-23.2)	1794	491
exo1Δ	678	12.0+/-1.1	530	145	3	3598	11.0 (10.0-12.0)	3198	394
mlh3Δ	210	13.6+/-2.7	168	39	3	1191	10.5 (8.8-12.4)	1066	125
exo1Δ mlh3Δ	238	11+/-1.3	184	52	0	1221	10.4 (8.7-12.2)	1092	127
msh5Δ	151	10.9+/-1.7	118	33	0	1111	8.5 (6.9-10.2)	1017	94
exo1Δ mus81Δ	141	2.8+/-0.9	133	8	0	1796	1.3 (0.9-1.9)	1772	24
exo1-D78A,D173A	374	19.5+/-1.9	248	122	4	2113	16.8 (15.3-18.5)	1757	356
exo1-D171A,D173A	523	18.8+/-1.1	326	197	0	2681	19.0 (17.5-20.5)	2172	509
exo1-K85A	329	23.6+/-1.6	179	149	1	1659	22.8 (20.8-24.9)	1280	379
exo1-R92A	336	22.5+/-1.8	195	139	2	1663	21.9 (20.0-24.0)	1298	365
exo1-S41E	353	12.7+/-1.2	263	90	0	1796	13.4 (11.8-15.1)	1555	241
exo1-F58E	302	18.5+/-1.7	195	106	1	1600	17.3 (15.4-19.2)	1324	276
exo1-K185E	464	15.5+/-1.9	355	102	7	2285	13.3 (11.9-14.7)	1982	303
exo1-G236D	752	17.2+/-1.3	529	216	7	3855	15.7 (14.6-16.8)	3250	605
exo1-K185E G236D	410	16.3+/-1.5	286	122	2	2409	15.5 (14.1-17.0)	2033	374
exo1-MIP	411	14.8+/-1.6	304	104	3	1849	12.9 (11.4-14.4)	1610	239
exo1-K185E,MIP	560	13.8+/-1.3	420	137	3	2703	12.1 (10.9-13.4)	2375	328
exo1Δ + pRAD27 (EXO1 Promoter)	423	12.4+/-1.1	318	105	0	2332	12.2 (10.9-13.6)	2045	284
exo1Δ + pEXO1	208	21.9+/-2.5	127	79	2	1072	19.4 (17.1-21.9)	864	208
exo1Δ + pEmpty Vector	220	12.5+/-2.3	175	43	2	1149	10.2 (8.5-12.1)	1032	117
LEU2-LYS2:									
wild-type	501	28.7+/-1.5	233	264	4	2285	27.6 (25.7-29.4)	1655	630
exo1Δ	678	12.5+/-1.0	518	158	2	3598	12.4 (11.3-13.5)	3148	444
mlh3Δ	210	14.3+/-2.0	155	54	1	1191	13.1 (11.2-15.1)	1035	156
exo1Δ mlh3Δ	238	10.4+/-1.3	187	49	0	1221	11.5 (9.7-13.4)	1079	140
msh5Δ	151	9.6+/-1.7	122	29	0	1111	9.5 (7.9-11.4)	1005	106
exo1Δ mus81Δ	141	3.2+/-1.0	132	9	0	1796	2.6 (1.9-3.5)	1749	47
exo1-D78A,D173A	374	27.8+/-1.9	186	184	4	2113	25.3 (23.4-27.2)	1579	534
exo1-D171A.D173A	523	23.6+/-1.1	276	247	0	2681	22.8 (21.2-24.5)	2069	612
exo1-K85A	329	26.4+/-1.8	165	162	2	1659	24.6 (22.5-26.7)	1251	408
exo1-R92A	336	29.2+/-2.1	160	172	4	1663	25.7 (23.7-27.9)	1239	428
exo1-S41E	353	14.6+/-1.6	260	91	2	1796	14.3 (12.7-16.0)	1540	256
exo1-F58E	302	16.6+/-1.6	207	94	1	1600	17.5 (15.7-19.5)	1320	280
exo1-K185E	464	18.5+/-2.2	342	112	10	2285	13.6 (12.2-15.1)	1974	311
exo1-G236D	752	18.4+/-1.3	511	234	7	3855	17.4 (16.2-18.6)	3186	669
exo1-K185E G236D	410	15.1+/-1.5	296	112	2	2409	13.6 (12.2-15.0)	2080	327

exo1-MIP	411	13.9+/-1.6	312	96	3	1849	12.1 (11.0-14.0)	1626	223
exo1-K185E,MIP	560	15.7+/-1.1	389	170	1	2703	15.1 (13.7-16.5)	2296	407
exo1Δ + *pRAD27 (EXO1 Promoter)*	423	19.6+/-1.8	277	142	4	2332	16.7 (15.2-18.3)	1940	389
exo1Δ + *pEXO1*	208	26.2+/-2.9	114	91	3	1072	22.7 (20.2-25.3)	829	243
exo1Δ + *pEmpty Vector*	220	12.7+/-1.9	169	50	1	1149	12.5 (10.7-14.6)	1005	144
LYS2-ADE2:									
wild-type	501	14.8+/-1.2	358	142	1	2285	14.8 (13.4-16.3)	1947	338
exo1Δ	678	7.1+/-0.9	592	84	2	3598	6.9 (6.1 -7.7)	3345	257
mlh3Δ	210	5.5+/-1.1	187	23	0	1191	5.1 (3.9-6.5)	1130	61
exo1Δ mlh3Δ	238	6.6+/-1.6	210	25	1	1221	6.1 (4.8-7.5)	1145	74
msh5Δ	151	4.6+/-1.3	137	14	0	1111	3.7 (2.7-5.0)	1070	41
exo1Δ mus81Δ	141	1.4+/-0	137	4	0	1796	1.4 (0.9-2.1)	1770	26
exo1-D78A,D173A	374	12.4+/-1.5	291	81	2	2113	11.2 (9.9-12.6)	1877	236
exo1-D171A,D173A	523	11.3+/-1.2	415	106	2	2681	11.6 (10.4-12.9)	2370	311
exo1-K85A	329	11.1+/-1.3	256	73	0	1659	11.5 (10.0-13.2)	1468	191
exo1-R92A	336	9.2+/-1.1	274	62	0	1663	10.3 (8.9-11.9)	1495	172
exo1-S41E	353	8.8+/-1.0	291	62	0	1796	8.5 (7.2-9.9)	1644	152
exo1-F58E	302	11.4+/-1.2	233	69	0	1600	11.6(10.1-13.3)	1414	186
exo1-K185E	464	10.0+/-1.4	386	75	3	2285	8.2 (7.1-9.4)	2098	187
exo1-G236D	752	11.9+/-1.1	598	149	5	3855	10.2 (9.3-11.2)	3461	393
exo1-K185E G236D	410	6.6+/-1.3	366	42	2	2409	5.9 (4.9-6.9)	2266	141
exo1-MIP	411	7.8+/-0.9	347	64	0	1849	7.4 (6.3-8.7)	1712	137
exo1-K185E, MIP	560	7.1+/-0.7	481	79	0	2703	6.6 (5.7-7.6)	2524	179
exo1Δ + *pRAD27 (EXO1 Promoter)*	423	8.2+/-0.9	354	69	0	2332	8.2 (7.1-9.4)	2138	191
exo1Δ + *pEXO1*	208	15.8+/-2.5	152	54	2	1072	14.2 (12.1-16.4)	920	152
exo1Δ + *pEmpty Vector*	220	5.4+/-1.0	196	24	0	1149	6.5 (5.2-8.1)	1074	75
ADE2-HIS4:									
wild-type	501	39+/-2.1	170	319	12	2285	35.1 (33.1-37.1)	1483	802
exo1Δ	678	23.1+/-1.7	425	241	12	3598	19.7 (18.4-21.0)	2884	708
mlh3Δ	210	22.9+/-3.1	134	72	4	1191	18.9 (16.7-21.2)	966	225
exo1Δ mlh3Δ	238	25.8+/-3.1	139	92	5	1221	21.3 (19.0-23.7)	959	260
msh5Δ	151	16.9+/-3.4	110	39	2	1111	13.0 (11.0-15.1)	967	144
exo1Δ mus81Δ	141	2.5+/-0.9	134	7	0	1796	1.5 (1.0-2.2)	1769	27
exo1-D78A,D173A	374	41.2+/-2.9	136	224	14	2113	34.0 (31.9-36.0)	1395	718
exo1-D171A D173A	523	37.7+/-2.5	224	280	19	2681	30.4 (28.6-32.1)	1867	814
exo1-K85A	329	37.4+/-3.0	138	180	11	1659	30.7 (28.5-33.0)	1149	510

exo1-R92A	336	38.5+/-3.1	137	187	12	1663	31.5 (29.3-33.8)	1142	525
exo1-S41E	353	28.3+/-2.7	198	146	9	1796	24.2 (22.2-26.3)	1361	435
exo1-F58E	302	28.1+/-2.1	147	152	3	1600	27.4 (25.5-29.6)	1162	438
exo1-K185E	464	20.9+/-1.8	295	164	5	2285	19.2 (17.6-20.8)	1847	438
exo1-G236D	752	23.8+/-1.8	470	263	19	3855	20.0 (19.8-22.4)	3042	812
exo1-K185E G236D	410	23.3+/-2.1	254	149	7	2409	20.1 (18.5-21.7)	1923	484
exo1-MIP	411	23.2+/-1.7	235	173	3	1849	21.3 (19.8-23.5)	1456	393
exo1-K185E,MIP	560	21.7+/-1.6	347	207	6	2703	19.1 (17.6-20.6)	2188	515
exo1Δ + pRAD27 (EXO1 Promoter)	423	32.4+/-3.1	249	154	20	2332	23.9 (22.2-25.7)	1772	557
exo1Δ + pEXO1	208	36.1+/-3.3	83	120	5	1072	32.1 (29.3-35.0)	728	344
exo1Δ + pEmpty Vector	220	23.2+/-2.4	128	90	2	1149	21.2 (18.9-23.7)	905	244

Mutants are isogenic derivatives of EAY1108/EAY1112. Genetic intervals correspond to the genetic distance calculated from tetrads +/- one standard error. Standard error was calculated using the Stahl Laboratory Online Tools website (https://elizabethhousworth.com/StahlLabOnlineTools/). For single spore analysis, data are shown as 95% confidence intervals around the recombination frequency. For tetrad analysis the centimorgan (cM) map distance was calculated using the formula of Perkins [1]: 50{TT+(6NPD)}/(PD+TT+NPD). To compare to the tetrad data, recombination frequencies obtained from single spores (Parental/(Parental+Recombinant)) were multiplied by 100 to yield genetic map distances (cM).

Reference

1. Perkins DD. Biochemical mutants in the smut fungus *Ustilago maydis*. Genetics. 1949;34: 607–626.
