# Supplementary material for: Exo1 protects DNA nicks from ligation to promote crossover formation during meiosis
Source: PLoS Biol. 2023 Apr 20;21(4):e3002085. doi: 10.1371/journal.pbio.3002085 (PMC10153752; doi:10.1371/journal.pbio.3002085)
Supplement: S4 Table — (A) Interference measurements on Chromosome XV. The Malkova ratio and coefficient of coincidence (COC, ratio of double crossovers observed/double crossovers expected) were performed for the indicated genotypes in the EAY1108/EAY1112 strain background (Materials and methods, strains listed in S5 Table). These methods were performed for intervals I (URA3-LEU2-LYS2), II (LEU2-LYS2-ADE2), and III (LYS2-ADE2-HIS3). 0 = Absolute Interference; 1 = No interference. Significance of differences in tetrad distribution was assessed using a G test. Differences in distribution with p < 0.05 were considered to be significant evidence of interference. Intervals with ratios significantly above 1 were observed and denoted with * to indicate potential negative interference. Detailed analysis of the Malkova ratio calculation is presented in S4B Table. (B) Detailed calculations of Malkova ratios presented in S4A Table and Fig 4C. Crossover interference was analyzed using the Malkova method [1,2] for chromosome XV. For each genetic interval, tetrads were divided based on the presence or absence of a recombination event in a reference interval. For each reference interval, the map distance was measured in the adjacent intervals, thus obtaining 2 map distances for each interval. The significance of differences in tetrad distribution was assessed using a G test. Differences in distribution with p < 0.05 were considered to be evidence of interference. The data are presented as the average ratio of the 2 map distances in each neighboring interval, with a smaller ratio indicating stronger interference. An interval was considered to have a “loss of positive interference” phenotype when both adjacent intervals displayed no detectable positive interference. Ratios significantly greater than 1 are indicated with * to denote potential negative interference. TT, tetratype; NPD, nonparental ditype; PD, parental ditype. (PDF) [file pbio.3002085.s010.pdf]

S4A Table. Interference measurements on Chromosome XV.

Intervals	I	II	III	# tetrads	Interference
wild-type					
Malkova	0.48	0.43	0.90	501	Intervals I, II
C.O.C	0.66	0.52	0.92		
exo1Δ					
Malkova	1.07	0.72	1.52	678	No intervals
C.O.C	1.02	0.79	1.22		
exo1-D171A,D173A					
Malkova	0.51	0.57	0.75	523	All intervals
C.O.C	0.62	0.70	0.72		
exo1-D78A, D173A					
Malkova	0.56	0.23	0.68	374	Intervals I, II
C.O.C	0.62	0.36	0.89		
exo1-K85A					
Malkova	0.62	0.68	0.84	329	Interval I
C.O.C	0.79	0.80	0.87		
exo1-R92A					
Malkova	0.62	0.67	0.73	336	Interval I
C.O.C	0.83	0.80	0.87		
exo1-S41E					
Malkova	0.75	0.35	0.57	353	Interval II
C.O.C	0.84	0.43	0.74		
exo1-F58E					
Malkova	0.62	0.29	0.80	302	Interval II
C.O.C	0.74	0.37	0.90		
exo1-G236D					
Malkova	0.49	0.38	0.83	752	Intervals I, II
C.O.C	0.63	0.57	0.83		
exo1-K185E					
Malkova	0.59	0.50	1.62*	464	Intervals II
C.O.C.	0.73	0.59	1.20		
exo1-G236D,K185E					
Malkova	0.50	0.82	1.19	410	Intervals I
C.O.C	0.64	0.98	0.89		
exo1-MIP					
Malkova	0.92	0.48	0.59	411	Interval II
C.O.C.	0.66	0.58	0.69		
exo1Δ + *pEXO1-RAD27*					
Malkova	1.18	0.94	0.89	423	No intervals
C.O.C	1.13	1.10	0.92		

mlh3Δ					
Malkova	0.46	0.58	1.08	210	Interval I
C.O.C	0.63	0.66	0.96		
msh5Δ					
Malkova	1.29	0.93	2.12*	151	No intervals
C.O.C	1.1	1.12	1.84		

The Malkova ratio and coefficient of coincidence (COC, ratio of double crossovers observed/double crossovers expected) were performed for the indicated genotypes in the EAY1108/EAY1112 strain background (Materials and Methods, strains listed in S5 Table). These methods were performed for intervals I (*URA3-LEU2-LYS2)*, II (*LEU2-LYS2-ADE2)*, and III *(LYS2-ADE2-HIS3)*. 0 = Absolute Interference; 1= No interference. Significance of differences in tetrad distribution was assessed using a G test. Differences in distribution with $p<0.05$ were considered to be significant evidence of interference. Intervals with ratios significantly above 1 were observed and denoted with * to indicate potential negative interference. Detailed analysis of the Malkova ratio calculation is presented in S4B Table.

S4B. Table. Detailed calculations of Malkova Ratios presented in Figure 4C and S4A Table.

wild-type											Malkova Ratio	COC	Malkova Interference significance (G-test)
Reference Interval	*URA3-LEU2*		*LEU2-LYS2*			*LYS2-ADE2*			*ADE2-HIS3*	U-L-K	0.478252195	0.662	Yes
Measured Interval	*LEU2-LYS2*		*URA3-LEU2*	*LYS2-ADE2*		*LEU2-LYS2*	*ADE2-HIS3*		*LYS2-ADE2*	L-K-A	0.425192148	0.523	Yes
PD	98:191:3	PD	98:132:3	130:102:1	PD	130:226:2	114:238:6	PD	114:55:1	K-A-H	0.899864346	0.921	No
cM	35.8	cM	32.2	23.2	cM	33.2	38.3	cM	17.9				
TT+NPD	135:73:1	TT+NPD	194:74:0	228:40:0	TT+NPD	103:38:2	56:81:6	TT+NPD	244:87:0				
cM	18.9	cM	13.8	7.5	cM	17.5	40.9	cM	13.1				
p	<0.05	p	<0.05	<0.05	p	<0.05	0.061	p	0.117				
Ratio	0.52793296		0.42857143	0.32327586		0.52710843	1.06788512		0.73184358				

exo1Δ													
Reference Interval	*URA3-LEU2*		*LEU2-LYS2*			*LYS2-ADE2*			*ADE2-HIS3*	U-L-K	1.073990572	1.02	No
Measured Interval	*LEU2-LYS2*		*URA3-LEU2*	*LYS2-ADE2*		*LEU2-LYS2*	*ADE2-HIS3*		*LYS2-ADE2*	L-K-A	0.718743121	0.79	No
PD	405:124:1	PD	405:111:2	448:68:2	PD	467:122:3	378:204:10	PD	378:47:0	K-A-H	1.520831635	1.22	No
cM	12.3	cM	11.9	7.7	cM	11.8	22.3	cM	5.5				
TT+NPD	113:34:1	TT+NPD	125:34:1	144:16:0	TT+NPD	70:16:0	47:37:2	TT+NPD	214:37:2				
cM	13.5	cM	12.5	5	cM	9.3	28.5	cM	9.7				
p	0.68	p	0.93	0.32	p	0.6	0.26	p	0.75				
Ratio	1.09756098	Ratio	1.05042017	0.64935065	Ratio	0.78813559	1.27802691	Ratio	1.76363636				

exo1-D171A,D173A													
Reference Interval	*URA3-LEU2*		*LEU2-LYS2*			*LYS2-ADE2*			*ADE2-HIS3*	U-L-K	0.512755102	0.62	Yes
Measured Interval	*LEU2-LYS2*		*URA3-LEU2*	*LYS2-ADE2*		*LEU2-LYS2*	*ADE2-HIS3*		*LYS2-ADE2*	L-K-A	0.5737066	0.7	Yes
PD	119:170:0	PD	137:139:0	204:70:2	PD	204:211:0	161:239:15	PD	144:53:0	K-A-H	0.750420875	0.72	Yes
cM	29.4	cM	25.2	14.9	cM	25.4	39.6	cM	13.5				
TT+NPD	157:77:0	TT+NPD	189:58:0	211:36:0	TT+NPD	72:36:0	63:41:4	TT+NPD	271:53:2				
cM	16.5	cM	11.7	7.3	cM	16.7	30.1	cM	10				
p	<0.05	p	<0.05	<0.05	p	<0.05	<0.05	p	<0.05				
Ratio	0.56122449		0.46428571	0.48993289		0.65748031	0.76010101		0.74074074				

exo1-D78A,D173A													
Reference Interval	*URA3-LEU2*		*LEU2-LYS2*			*LYS2-ADE2*			*ADE2-HIS3*	U-L-K	0.562742813	0.62	Yes
Measured Interval	*LEU2-LYS2*		*URA3-LEU2*	*LYS2-ADE2*		*LEU2-LYS2*	*ADE2-HIS3*		*LYS2-ADE2*	L-K-A	0.230780264	0.36	Yes
PD	99:145:4	PD	99:87:0	118:66:2	PD	118:169:4	100:177:14	PD	100:35:1	K-A-H	0.676775367	0.89	No
cM	34.1	cM	23.4	21	cM	33.2	44.8	cM	15.1				
TT+NPD	87:39:0	TT+NPD	149:35:4	173:15:0	TT+NPD	68:15:0	36:47:0	TT+NPD	191:46:1				
cM	15.5	cM	15.7	4	cM	9	28.3	cM	10.9				
p	<0.05	p	<0.05	<0.05	p	<0.05	<0.05	p	0.32				
Ratio	0.45454545		0.67094017	0.19047619		0.27108434	0.63169643		0.7218543				

exo1-K85A													
Reference Interval	*URA3-LEU2*		*LEU2-LYS2*			*LYS2-ADE2*			*ADE2-HIS3*	U-L-K	0.616131933	0.79	Yes
Measured Interval	*LEU2-LYS2*		*URA3-LEU2*	*LYS2-ADE2*		*LEU2-LYS2*	*ADE2-HIS3*		*LYS2-ADE2*	L-K-A	0.682417174	0.8	No
PD	74:103:2	PD	74:90:1	121:44:0	PD	121:133:2	102:146:8	PD	102:36:0	K-A-H	0.842733915	0.87	No
cM	32.1	cM	29.1	13.3	cM	28.3	37.9	cM	13				
TT+NPD	91:59:0	TT+NPD	105:59:0	135:29:0	TT+NPD	44:29:0	36:34:03	TT+NPD	154:37:0				
cM	19.7	cM	18	8.8	cM	19.9	35.6	cM	9.7				
p	<0.05	p	<0.05	0.145	p	0.098	0.286	p	0.354				
Ratio	0.61370717		0.6185567	0.66165414		0.70318021	0.93931398		0.74615385				

exo1-R92A

Reference Interval	*URA3-LEU2*		*LEU2-LYS2*			*LYS2-ADE2*			*ADE2-HIS3*	U-L-K	0.61996791	0.83	Yes
Measured Interval	*LEU2-LYS2*		*URA3-LEU2*	*LYS2-ADE2*		*LEU2-LYS2*	*ADE2-HIS3*		*LYS2-ADE2*	L-K-A	0.666143306	0.8	No
PD	80:111:4	PD	80:78:2	124:36:0	PD	124:146:4	107:156:11	PD	107:30:0	K-A-H	0.734873712	0.87	No
cM	34.6	cM	28.1	11.3	cM	31	40.5	cM	10.9				
TT+NPD	80:61:0	TT+NPD	115:61:0	150:26:0	TT+NPD	36:26:0	30:31:01	TT+NPD	167:32:0				
cM	21.6	cM	17.3	7.4	cM	21	29.8	cM	8				
p	<0.05	p	<0.05	0.19	p	0.099	0.291	p	0.406				
Ratio	0.62427746		0.61565836	0.65486726		0.67741935	0.73580247		0.73394495				

exo1-S41E

Reference Interval	*URA3-LEU2*		*LEU2-LYS2*			*LYS2-ADE2*			*ADE2-HIS3*	U-L-K	0.751265823	0.84	No
Measured Interval	*LEU2-LYS2*		*URA3-LEU2*	*LYS2-ADE2*		*LEU2-LYS2*	*ADE2-HIS3*		*LYS2-ADE2*	L-K-A	0.348942253	0.43	Yes
PD	190:71:2	PD	190:70:0	205:55:0	PD	205:84:2	156:126:9	PD	156:42:0	K-A-H	0.567121573	0.74	No
cM	15.8	cM	13.5	10.6	cM	16.5	30.9	cM	10.6				
TT+NPD	70:20:0	TT+NPD	73:20:0	86:7:0	TT+NPD	55:7:0	42:20:0	TT+NPD	135:20:0				
cM	11.1	cM	10.8	3.8	cM	5.6	16.1	cM	6.5				
p	0.356	p	0.581	<0.05	p	<0.05	<0.05	p	0.12				
Ratio	0.70253165	Ratio	0.8	0.35849057	Ratio	0.33939394	0.5210356	Ratio	0.61320755				

exo1-F58E

Reference Interval	*URA3-LEU2*		*LEU2-LYS2*			*LYS2-ADE2*			*ADE2-HIS3*	U-L-K	0.618973214	0.74	No
Measured Interval	*LEU2-LYS2*		*URA3-LEU2*	*LYS2-ADE2*		*LEU2-LYS2*	*ADE2-HIS3*		*LYS2-ADE2*	L-K-A	0.290065265	0.37	Yes
PD	125:69:1	PD	125:81:1	146:61:0	PD	146:86:1	110:120:3	PD	110:37:0	K-A-H	0.800622051	0.9	No
cM	19.2	cM	21	14.7	cM	19.7	29.6	cM	12.6				
TT+NPD	82:25:0	TT+NPD	70:25:0	87:8:0	TT+NPD	61:8:0	37:32:0	TT+NPD	123:32:0				
cM	11.7	cM	13.2	4.2	cM	5.8	23.2	cM	10.3				
p	0.055	p	0.058	<0.05	p	<0.05	0.32	p	0.645				
Ratio	0.609375	Ratio	0.62857143	0.28571429	Ratio	0.29441624	0.78378378	Ratio	0.81746032				

exo1-G236D

Reference Interval	*URA3-LEU2*		*LEU2-LYS2*			*LYS2-ADE2*			*ADE2-HIS3*	U-L-K	0.486553337	0.63	Yes
Measured Interval	*LEU2-LYS2*		*URA3-LEU2*	*LYS2-ADE2*		*LEU2-LYS2*	*ADE2-HIS3*		*LYS2-ADE2*	L-K-A	0.377946298	0.57	Yes
PD	333:189:7	PD	333:172:6	341:160:10	PD	385:206:7	364:219:15	PD	364:103:3	K-A-H	0.82751938	0.83	No
cM	21.8	cM	20.4	21.5	cM	20.7	25.8	cM	12.9				
TT+NPD	178:45:0	TT+NPD	196:44:1	213:27:1	TT+NPD	126:28:0	106:44:4	TT+NPD	234:46:2				
cM	10.1	cM	10.4	6.8	cM	9.1	22.1	cM	10.3				
p	<0.05	p	<0.05	<0.05	p	<0.05	0.165	p	0.169				
Ratio	0.46330275	Ratio	0.50980392	0.31627907	Ratio	0.43961353	0.85658915	Ratio	0.79844961				

exo1-K185E

Reference Interval	*URA3-LEU2*		*LEU2-LYS2*			*LYS2-ADE2*			*ADE2-HIS3*	U-L-K	0.598083506	0.73	No
Measured Interval	*LEU2-LYS2*		*URA3-LEU2*	*LYS2-ADE2*		*LEU2-LYS2*	*ADE2-HIS3*		*LYS2-ADE2*	L-K-A	0.501305653	0.59	Yes
PD	254:92:9	PD	254:82:6	276:64:2	PD	276:100:10	251:133:2	PD	251:43:1	K-A-H	1.618238913	1.2	No*
cM	20.6	cM	17.3	11.1	cM	20.7	18.8	cM	8.3				
TT+NPD	88:20:1	TT+NPD	101:20:1	110:11:1	TT+NPD	66:12:0	44:31:3	TT+NPD	135:32:2				
cM	11.9	cM	10.7	7	cM	7.7	31.4	cM	13				
p	0.119	p	0.14	<0.05	p	<0.05	<0.05	p	0.257				
Ratio	0.5776699	Ratio	0.61849711	0.63063063	Ratio	0.37198068	1.67021277	Ratio	1.56626506				

exo1-K185E,G236D

Reference Interval	*URA3-LEU2*		*LEU2-LYS2*			*LYS2-ADE2*			*ADE2-HIS3*	U-L-K	0.503968254	0.64	Yes
Measured Interval	*LEU2-LYS2*		*URA3-LEU2*	*LYS2-ADE2*		*LEU2-LYS2*	*ADE2-HIS3*		*LYS2-ADE2*	L-K-A	0.817683881	0.98	No
PD	194:90:2	PD	194:100:2	264:30:2	PD	264:100:2	225:135:6	PD	225:29:0	K-A-H	1.186797121	0.89	No
cM	17.8	cM	18.9	7.1	cM	15.3	23.4	cM	5.7				
TT+NPD	102:22:0	TT+NPD	92:22:0	102:12:0	TT+NPD	32:12:0	29:14:1	TT+NPD	141:13:2				
cM	8.9	cM	9.6	5.3	cM	13.6	22.7	cM	8				
p	<0.05	p	<0.05	0.518	p	0.796	0.78	p	0.09				
Ratio	0.5		0.50793651	0.74647887		0.88888889	0.97008547		1.40350877				

exo1-MIP

Reference Interval	*URA3-LEU2*		*LEU2-LYS2*			*LYS2-ADE2*			*ADE2-HIS3*	U-L-K	0.917190776	0.66	No
Measured Interval	*LEU2-LYS2*		*URA3-LEU2*	*LYS2-ADE2*		*LEU2-LYS2*	*ADE2-HIS3*		*LYS2-ADE2*	L-K-A	0.482655502	0.58	Yes
PD	222:82:0	PD	222:87:2	256:55:0	PD	256:87:3	190:153:3	PD	190:45:0	K-A-H	0.592990891	0.69	No
cM	13.5	cM	15.9	8.8	cM	15.2	24.7	cM	9.6				
TT+NPD	90:14:3	TT+NPD	82:17:1	91:9:0	TT+NPD	56:9:0	45:20:0	TT+NPD	157:19:0				
cM	15	cM	11.5	4.5	cM	6.9	15.4	cM	5.4				
p	0.06	p	0.075	0.09	p	<0.05	0.066	p	0.063				
Ratio	1.11111111	Ratio	0.72327044	0.51136364	Ratio	0.45394737	0.62348178	Ratio	0.5625				

exo1Δ pEXO1-RAD27

Reference Interval	*URA3-LEU2*		*LEU2-LYS2*			*LYS2-ADE2*			*ADE2-HIS3*	U-L-K	1.182813355	1.13	No
Measured Interval	*LEU2-LYS2*		*URA3-LEU2*	*LYS2-ADE2*		*LEU2-LYS2*	*ADE2-HIS3*		*LYS2-ADE2*	L-K-A	0.93900866	1.1	No
PD	213:102:3	PD	213:64:0	232:45:0	PD	232:118:4	206:131:17	PD	206:43:0	K-A-H	0.887414293	0.92	No
cM	18.9	cM	11.6	8.1	cM	20.1	32.9	cM	8.6				
TT+NPD	64:40:1	TT+NPD	105:41:0	122:24:0	TT+NPD	45:24:0	43:23:3	TT+NPD	148:26:0				
cM	21.9	cM	14	8.2	cM	17.4	29.7	cM	7.5				
p	0.0529	p	0.534	0.999	p	0.482	0.815	p	0.815				
Ratio	1.15873016		1.20689655	1.01234568		0.86567164	0.90273556		0.87209302				

mlh3Δ

Reference Interval	*URA3-LEU2*		*LEU2-LYS2*			*LYS2-ADE2*			*ADE2-HIS3*	U-L-K	0.461415992	0.63	Yes
Measured Interval	*LEU2-LYS2*		*URA3-LEU2*	*LYS2-ADE2*	Interval	*LEU2-LYS2*	*ADE2-HIS3*		*LYS2-ADE2*	L-K-A	0.585081967	0.66	No
PD	120:47:1	PD	120:32:3	136:19:0	PD	136:50:1	119:65:3	PD	119:16:0	K-A-H	1.081273485	0.96	No
cM	15.8	cM	16.1	6.1	cM	15	22.2	cM	5.97				
TT+NPD	35:7:0	TT+NPD	48:7:0	51:4:0	TT+NPD	19:4:0	15:7:1	TT+NPD	67:8:0				
cM	8.3	cM	6.4	3.6	cM	8.7	28.3	cM	5.3				
p	0.23	p	<0.05	0.572	p	0.53	0.689	p	0.989				
Ratio	0.52531646	Ratio	0.39751553	0.59016393	Ratio	0.58	1.27477477	Ratio	0.88777219				

msh5Δ

Reference Interval	*URA3-LEU2*		*LEU2-LYS2*			*LYS2-ADE2*			*ADE2-HIS3*	U-L-K	1.289960965	1.104	No
Measured Interval	*LEU2-LYS2*		*URA3-LEU2*	*LYS2-ADE2*		*LEU2-LYS2*	*ADE2-HIS3*		*LYS2-ADE2*	L-K-A	0.932962963	1.116	No
PD	96:23:0	PD	96:25:0	108:13:0	PD	108:27:0	96:33:0	PD	96:17:0	K-A-H	2.119514628	1.841	No*
cM	9.7	cM	10.3	5.4	cM	10	12.8	cM	7.52				
TT+NPD	24:8:0	TT+NPD	22:8:0	27:3:0	TT+NPD	13:3:0	15:5:2	TT+NPD	31:7:0				
cM	12.5	cM	13.3	5	cM	9.4	38.6	cM	9.2				
p	0.787	p	0.783	0.993	p	0.99	<0.05	p	0.835				
Ratio	1.28865979		1.29126214	0.92592593		0.94	3.015625		1.22340426				

*Potential negative interfernence

U-L-K	*URA3-LEU2-LYS2*
L-K-A	*LEU2-LYS2-ADE2*
K-A-H	*LYS2--ADE2-HIS3*

Crossover interference was analyzed using the Malkova method [1,2] for chromosome XV. For each genetic interval, tetrads were divided based on the presence or absence of a recombination event in a reference interval. For each reference interval, the map distance was measured in the adjacent intervals, thus obtaining two map distances for each interval. The significance of differences in tetrad distribution was assessed using a G test. Differences in distribution, with p<0.05, were considered to be evidence of interference. The data are presented as the average ratio of the two map distances in each neighboring interval, with a smaller ratio indicating stronger interference. An interval was considered to have a “loss of positive interference” phenotype when both adjacent intervals displayed no detectable positive interference. Ratios significantly greater than 1 are indicated with * to denote potential negative interference. TT, tetratype; NPD. nonparental ditype; PD, parental ditype.

References

1. Malkova A, Swanson J, German M, McCusker JH, Housworth EA, Stahl FW, Haber JE. Gene conversion and crossing over along the 405-kb left arm of *Saccharomyces cerevisiae* chromosome VII. Genetics. 2004;168: 49–63.
2. Martini E, Diaz RL, Hunter N, Keeney S. Crossover homeostasis in yeast meiosis. Cell. 2006;126: 285–295.
