# Supplementary material for: Exo1 protects DNA nicks from ligation to promote crossover formation during meiosis
Source: PLoS Biol. 2023 Apr 20;21(4):e3002085. doi: 10.1371/journal.pbio.3002085 (PMC10153752; doi:10.1371/journal.pbio.3002085)
Supplement: S5 Table — (DOCX) [file pbio.3002085.s011.docx]

| **S5 Table. Strains used in this study.**  **A. *exo1* mutant analysis** | | |
| --- | --- | --- |
| **Strain** | **Genotype** | **Purpose** |
| **A. *exo1* mutant analysis, spore autonomous, SK1 isogenic background** |  |  |
| SKY3576 | *MATa, ho::LYS2, lys2, ura3, leu2::hisG, trp1::hisG, THR1::m‐Cerulean‐TRP1* | Integration of *exo1* mutant alleles |
| SKY3575 | *MATα,* *ho::LYS2, lys2, ura3, leu2::hisG, trp1::hisG, CEN8::tdTomato‐LEU2* | Integration of *exo1* mutant alleles |
| EAY4149-EAY4150 | Same as SKY3576, but *exo1Δ::KANMX* | *exo1Δ* negative control |
| EAY4151-EAY4153 | Same as SKY3575, but *exo1Δ::KANMX* | *exo1Δ* negative control |
| EAY5024-EAY5025 | Same as SKY3576, but *rad27Δ::KANMX* | Testing a role for Rad27 in meiotic crossing over |
| EAY5026-EAY5027 | Same as SKY3575, but *rad27Δ::KANMX* | Testing a role for Rad27 in meiotic crossing over |
| EAY4154-EAY4156 | Same as SKY3576, but *EXO1::KANMX* | *EXO1::KANMX* control |
| EAY4157-EAY4159 | Same as SKY3575, but *EXO1::KANMX* | *EXO1::KANMX* control |
| EAY4160-EAY4162 | Same as SKY3576, but *exo1-D78A::KANMX* | *D78A* Mutant |
| EAY4163-EAY4164 | Same as SKY3575, but *exo1- D78A::KANMX* | *D78A* Mutant |
| EAY4165-EAY4167 | Same as SKY3576, but *exo1-D173A::KANMX* | *D173A* mutant |
| EAY4168-EAY4170 | Same as SKY3575, but *exo1-D173A::KANMX* | *D173A* mutant |
| EAY4171-EAY4172 | Same as SKY3576, but *exo1-G236D::KANMX* | *G236D* mutant |
| EAY4173-EAY4174 | Same as SKY3575, but *exo1-G236D::KANMX* | *G236D* mutant |
| EAY4175-EAY4177 | Same as SKY3576, but *exo1-D78A,D173A::KANMX* | *D78A,D173A* mutant |
| EAY4178 | Same as SKY3575, but *exo1-D78A,D173A::KANMX* | *D78A,D173A* mutant |
| EAY4179 | Same as SKY3576, but *exo1-D173A,G236D::KANMX* | *D173A,G236D* mutant |
| EAY4180-EAY4181 | Same as SKY3575, but *exo1-D173A,G236D::KANMX* | *D173A,G236D* mutant |
| EAY4182-EAY4184 | Same as SKY3576, but *exo1-F447A,F448A::KANMX* | *F447A,F448A* mutant (MIP) |
| EAY4185-EAY4187 | Same as SKY3575, but *exo1-F447A,F448A::KANMX* | *F447A,F448A* mutant (MIP) |
| EAY4510-EAY4511 | Same as SKY3576, but *exo1-D171A::KANMX* | *D171A* mutant |
| EAY4512-EAY4513 | Same as SKY3576, but *exo1-D171A,D173A::KANMX* | *D171A,D173A* mutant |
| EAY4514-EAY4515 | Same as SKY3576, but *exo1-R92A::KANMX* | *R92A* mutant |
| EAY4516-EAY4517 | Same as SKY3576, but *exo1-K121A::KANMX* | *K121A* mutant |
| EAY4518-EAY4519 | Same as SKY3576, but *exo1-K121E::KANMX* | *K121E* mutant |
| EAY4520-EAY4521 | Same as SKY3576, but *exo1-K185A::KANMX* | *K185A* mutant |
| EAY4522-EAY4523 | Same as SKY3576, but *exo1-K185E::KANMX* | *K185E* mutant |
| EAY4524-EAY4525 | Same as SKY3576, but *exo1-D173A,G236D::KANMX* | *D173A,G236D* mutant |
| EAY4526-EAY4527 | Same as SKY3576, but *exo1-G236D,F447A,F448A::KANMX* | *G236D,F447A,F448A* mutant |
| EAY4528-EAY4529 | Same as SKY3576, but *exo1-D173A,G236D,F447A,F448A::KANMX* | *D173A,G236D,F447A,F448A* mutant |
| EAY4530-EAY4531 | Same as SKY3576, but *exo1-K185E,F447A,F448A::KANMX* | *K185E,F447A,F448A* mutant |
| EAY4532-EAY4533 | Same as SKY3576, but *exo1-D173A,K185E,G236D::KANMX* | *D173A,K185E,G236D* mutant |
| EAY4534-EAY4535 | Same as SKY3576, but *exo1-D171A,G236D::KANMX* | *D171A,G236D* mutant |
| EAY4536-EAY4537 | Same as SKY3576, but *exo1-K185E,G236D::KANMX* | *K185E,G236D* mutant |
| EAY4538-EAY4539 | Same as SKY3576, but *exo1-R92A,K121A,K185A::KANMX* | *R92A,K121A,K185A* mutant |
| EAY4805-EAY4806 | Same as SKY3576, but *exo1-H36E::KANMX* | *H36E* mutant |
| EAY4807-EAY4808 | Same as SKY3576, but *exo1-S41E::KANMX* | *S41E* mutant |
| EAY4809-EAY4812 | Same as SKY3576, but *exo1-F58E::KANMX* | *F58E* mutant |
| EAY4813-EAY4814 | Same as SKY3576, but *exo1-K61A::KANMX* | *K61A* mutant |
| EAY4815-EAY4817 | Same as SKY3576, but *exo1-K61E::KANMX* | *K61E* mutant |
| EAY4818-EAY4820 | Same as SKY3576, but *exo1-K85A::KANMX* | *K85A* mutant |
| EAY4821-EAY4822 | Same as SKY3576, but *exo1-K85E::KANMX* | *K85E* mutant |
| EAY4881-EAY4882 | Same as SKY3576, but *exo1-S41E,F58E::KANMX* | *S41E,F58E* mutant |
| EAY4883-EAY4884 | Same as SKY3576, but *exo1-S41E,K61E::KANMX* | *S41E,K61E* mutant |
| EAY5076-EAY5077 | Same as SKY3576, but *EXO1-13MYC::KANMX* | MYC tagged for Western blot |
| EAY5078-EAY5079 | Same as SKY3576, but *exo1-S41E-13MYC::KANMX* | *S41E* mutant MYC tagged for Western blot |
| EAY5080-EAY5081 | Same as SKY3576, but *exo1-F58E-13MYC::KANMX* | *F58E* mutant MYC tagged for Western blot |
| EAY5082-EAY5083 | Same as SKY3576, but *exo1-K185E-13MYC::KANMX* | *K185E* mutant MYC tagged for Western blot |
| EAY5084-EAY5085 | Same as SKY3576, but *exo1-G236D-13MYC::KANMX* | *G236D* mutant MYC tagged for Western blot |
| **B. *exo1* mutant analysis, tetrad analysis, SK1 congenic background** |  |  |
| EAY1108 | *MATa, trp1:hisG leu2::hisG ho::hisG ura3 lys2 URA3insertion@CENXV LEU2insertion@chromXV, LYS2 insertion at position 505193 on chromosome XV* |  |
| EAY1112 | *MATalpha, ura3, trp1::hisG, leu2::hisG, lys2, ho::hisG, ade2::hisG, his3∆::hisG, TRP1insertion@CENXV* |  |
| EAY1281 | Same as EAY1108 but *msh5Δ::NATMX* |  |
| EAY1282 | Same as EAY1112 but *msh5Δ::NATMX* |  |
| EAY1847 | Same as EAY1108 but *mlh3Δ::KANMX* |  |
| EAY1848 | Same as EAY1112 but *mlh3Δ::KANMX* |  |
| EAY4778 | Same as EAY1108 but *exo1Δ::KANMX* |  |
| EAY4779 | Same as EAY1112 but *exo1Δ::KANMX* |  |
| EAY4780 | Same as EAY1112 but *D171A,D173A::KANMX* |  |
| EAY4781 | Same as EAY1112 but *D78A,D173::KANMX* |  |
| EAY4782 | Same as EAY1112 but *exo1*-*K185E::KANMX* |  |
| EAY4783 | Same as EAY1112 but *exo1*-*G236D::KANMX* |  |
| EAY4784 | Same as EAY1112 but *exo1-S41E::KANMX* |  |
| EAY4785 | Same as EAY1112 but *exo1-F58E::KANMX* |  |
| EAY4786 | Same as EAY1112 but *exo1Δ::KANMX mus81Δ::NATMX* |  |
| EAY4787 | Same as EAY1108 but *exo1Δ::KANMX mus81Δ::NATMX* |  |
| EAY4788 | Same as EAY1112 but *exo1Δ::KANMX mlh3Δ::NATMX* |  |
| EAY4789 | Same as EAY1108 but *exo1Δ::KANMX mlh3Δ::NATMX* |  |
| EAY4790 | Same as EAY1112 but exo1-*MIP::KANMX* |  |
| EAY4791 | Same as EAY1112 but *exo1-MIP, K185E::KANMX* |  |
| EAY4792 | Same as EAY1112 but *exo1-G236D,D173A::KANMX* |  |
| EAY4880 | Same as EAY1112 but *exo1-K185E,G236D::KANMX* |  |
| EAY5013 | Same as EAY1112 but *exo1-R92A::KANMX* |  |
| EAY5015 | Same as EAY1112 but *exo1-K85A::KANMX* |  |
| **C. Msh5 ChIP-qPCR, ChIP-Seq, and localization studies, SK1 isogenic background** |  |  |
| NHY1162 | *MATα, ho::hisG, leu::hisG, ura3(∆Sma-Pst), his4X::LEU2-(NgoMIV)::URA3* | ChIP-qPCR, Msh5 localization |
| NHY1168 | *MATa, ho::hisG, leu2::hisG, ura3(∆Sma-Pst), HIS4::LEU2-(BamHI)* | ChIP-qPCR, Msh5 localization |
| KTY753 | *MATa/MATα, ho::hisG/ho::hisG, leu2::hisG/leu2::hisG, ura3(∆Sma-Pst)/ ura3(∆Sma-Pst), his4-X::LEU2-(NgoM IV)::URA3/HIS4::LEU2-(BamH1), exo1∆::KanMX4/exo1∆::KanMX4* | ChIP-qPCR, Msh5 localization |
| KTY756 | *MATα, ho::hisG, leu2::hisG, ura3(∆Sma-Pst), his4-X::LEU2-(NgoM IV)::URA3, exo1∆::KanMX4* | ChIP-qPCR, Msh5 localization |
| KTY757 | *MATa, ho::hisG, leu2::hisG, ura3(∆Sma-Pst), HIS4::LEU2-(BamH1), exo1∆::KanMX4* | ChIP-qPCR, Msh5 localization |
