# Supplementary material for: Exo1 protects DNA nicks from ligation to promote crossover formation during meiosis
Source: PLoS Biol. 2023 Apr 20;21(4):e3002085. doi: 10.1371/journal.pbio.3002085 (PMC10153752; doi:10.1371/journal.pbio.3002085)
Supplement: S6 Table — (DOCX) [file pbio.3002085.s012.docx]

| **S6 Table. Plasmids used in this study.** | | |
| --- | --- | --- |
| **Plasmid** | **Markers** | **Purpose** |
| pUC18 | *amp^R^* | Exo1 endonuclease assay substrate |
| pBR322 | *amp^R^* | Exo1 endonuclease assay substrate |
| pRS416 | *amp^R^, URA3, CEN6-ARSH4* | Empty vector control |
| pLZ259 | *amp^R^, NATMX, CEN6-ARSH4* | Empty vector control |
| pRS426 | *amp^R^, URA3, 2µ* | Empty vector control |
| pEAI422 | *amp^R^, KANMX* | Integration of *exo1Δ-KANMX* |
| pEAI423 | *amp^R^, KANMX* | Integration of *EXO1-KANMX* |
| pEAI442 | *amp^R^, KANMX* | Integration of *exo1-H36E* |
| pEAI471 | *amp^R^, KANMX* | Integration of *exo1-S41E* |
| pEAI472 | *amp^R^, KANMX* | Integration of *exo1-F58E* |
| pEAI473 | *amp^R^, KANMX* | Integration of *exo1-K61A* |
| pEAI474 | *amp^R^, KANMX* | Integration of *exo1-K61E* |
| pEAI444 | *amp^R^, KANMX* | Integration of *exo1-K85A* |
| pEAI475 | *amp^R^, KANMX* | Integration of *exo1-K85E* |
| pEAI476 | *amp^R^, KANMX* | Integration of *exo1-S41E,F58E* |
| pEAI478 | *amp^R^, KANMX* | Integration of *exo1-S41E,K61E* |
| pEAI424 | *amp^R^, KANMX* | Integration of *exo1-D78A* |
| pEAI445 | *amp^R^, KANMX* | Integration of *exo1-R92A* |
| pEAI446 | *amp^R^, KANMX* | Integration of *exo1-K121A* |
| pEAI448 | *amp^R^, KANMX* | Integration of *exo1-K121E* |
| pEAI447 | *amp^R^, KANMX* | Integration of *exo1-D171A* |
| pEAI425 | *amp^R^, KANMX* | Integration of *exo1-D173A* |
| pEAI450 | *amp^R^, KANMX* | Integration of *exo1-K185A* |
| pEAI451 | *amp^R^, KANMX* | Integration of *exo1-K185E* |
| pEAI426 | *amp^R^, KANMX* | Integration of *exo1-G236D* |
| pEAI437 | *amp^R^, KANMX* | Integration of *exo1-F447A,F448A (MIP)* |
| pEAI427 | *amp^R^, KANMX* | Integration of *exo1-D78A,D173A* |
| pEAI449 | *amp^R^, KANMX* | Integration of *exo1-D171A,D173A* |
| pEAI456 | *amp^R^, KANMX* | Integration of *exo1-D171A,G236D* |
| pEAI436 | *amp^R^, KANMX* | Integration of *exo1-D173A,G236D* |
| pEAI458 | *amp^R^, KANMX* | Integration of *exo1-D173A,G236D,F447A,F448A (MIP)* |
| pEAI452 | *amp^R^, KANMX* | Integration of *exo1-G236D,F447A,F448A (MIP)* |
| pEAI467 | *amp^R^, KANMX* | Integration of *exo1-K185E,F447A,F448A (MIP)* |
| pEAI460 | *amp^R^, KANMX* | Integration of *exo1-D173A,K185E,G236D* |
| pEAI461 | *amp^R^, KANMX* | Integration of *exo1-K185E,G236D* |
| pEAI466 | *amp^R^, KANMX* | Integration of *exo1-R92A,K121A,K185A* |
| pEAI517 | *amp^R^, KANMX* | Integration of *EXO1-13MYC* |
| pEAI518 | *amp^R^, KANMX* | Integration of *exo1-S41E-13MYC* |
| pEAI519 | *amp^R^, KANMX* | Integration of *exo1-F58E-13MYC* |
| pEAI520 | *amp^R^, KANMX* | Integration of *exo1-K185E-13MYC* |
| pEAI521 | *amp^R^, KANMX* | Integration of *exo1-G236D-13MYC* |
| pFA6a-13MYC::KanMX6 | *amp^R^, KANMX* | 13MYC containing vector from Longtine et al., 1998 |
| pEAA715 | *amp^R^, URA3, CEN6-ARSH4, EXO1* | *EXO1* complementation |
| pEAI483 | *amp^R^, NATMX, CEN6-ARSH4, EXO1* | *EXO1* complementation |
| pEAA726 | *amp^R^, URA3, CEN6-ARSH4, MLH3* | *MLH3* complementation |
| pEAA636 | *amp^R^, HIS3, CEN6-ARSH4, MLH3, KANMX* | *MLH3* complementation |
| pEAA722 | *amp^R^, URA3, CEN6-ARSH4, RAD27* | *RAD27* expression, native promoter |
| pEAA720 | *amp^R^, URA3, CEN6-ARSH4, pEXO1-RAD27* | *RAD27* expression under *EXO1* promoter |
| pEAI482 | *amp^R^, NATMX, CEN6-ARSH4, pEXO1-RAD27* | Expression of *RAD27* under *EXO1* promoter |
| pEAA727 | *amp^R^, URA3, CEN6-ARSH4, pEXO1-rad27-A45E* | *rad27-A45E expression under EXO1 promoter* |
| pEAA728 | *amp^R^, URA3, CEN6-ARSH4, pEXO1-rad27-R101A* | *rad27-R101A expression under EXO1 promoter* |
| pEAA729 | *amp^R^, URA3, CEN6-ARSH4, pEXO1-rad27-R105A* | *rad27-R105A expression under EXO1 promoter* |
| pEAA730 | *amp^R^, URA3, CEN6-ARSH4, pEXO1-rad27-K130A* | *rad27-K130A expression under EXO1 promoter* |
| pEAA724 | *amp^R^, URA3, CEN6-ARSH4, pEXO1-rad27-D179A* | *rad27-D179A expression under EXO1 promoter* |
| pEAA731 | *amp^R^, URA3, CEN6-ARSH4, pEXO1-rad27-H191E* | *rad27-H191E expression under EXO1 promoter* |
| pEAM327 | *amp^R^, URA3, 2μ, CDC9* | *CDC9* expression, native promoter |
| pEAM329 | *amp^R^, URA3, 2µ, pHOP1-CDC9* | Overexpression of *CDC9* under the *HOP1* promoter |
| pEAM330 | *amp^R^, URA3, 2µ, pHOP1-cdc-F44A,F45A* | Overexpression of *cdc9-F44A,F45A* under the *HOP1* promoter |
| pEAM331 | *amp^R^, URA3, 2µ, pHOP1-cdc9-K419A* | Overexpression of *cdc9-K419A* under the *HOP1* promoter |
| pFB-EXO1-FLAG | *amp^R^, Gm^R^, EXO1-FLAG* | *EXO1* expression from pFastBac (From Michael Liskay) |
| pFB-exo1-D173A-FLAG | *amp^R^, Gm^R^, exo1-D173A-FLAG* | *exo1-D173A* expression from pFastBac (From Michael Liskay) |
| pEAE422 | *amp^R^, Gm^R^, exo1-G236D-FLAG* | *exo1-G236D* expression from pFastBac |
| pEAE423 | *amp^R^, Gm^R^, exo1-D173A-G236D-FLAG* | *exo1-D173A,G236D* expression from pFastBac |
