# Supplementary material for: Exo1 protects DNA nicks from ligation to promote crossover formation during meiosis
Source: PLoS Biol. 2023 Apr 20;21(4):e3002085. doi: 10.1371/journal.pbio.3002085 (PMC10153752; doi:10.1371/journal.pbio.3002085)
Supplement: S7 Table — (DOCX) [file pbio.3002085.s013.docx]

| **S7 Table. Oligonucleotides used in this study (shown 5’ to 3’).** | | |
| --- | --- | --- |
| **Primer** | **Sequence (lowercase indicates bases being mutated)** | **Purpose** |
| AO257 | GGAGCTCGAAAAAACTGAAAG | *EXO1* Sequencing |
| AO643 | CGGATGTGATGTGAGAACTG | *EXO1* Sequencing |
| AO694 | CCTGCGCCGGTTGCATTCGAT | *EXO1* Sequencing |
| AO804 | AGAAGGCTTCTTACTCCAACC | *EXO1* Sequencing |
| AO2383 | GAGACGGTCACAGCTTGTCT | *EXO1* Sequencing |
| AO3397 | ATATACCTGAAGGAGCAAGGACCTG | *EXO1* Sequencing |
| AO3398 | TAGTGACAAATCACTGGAAGACGAA | *EXO1* Sequencing |
| AO3399 | GGAAAATCAACTGATAAGTACCTCC | *EXO1* Sequencing |
| AO3400 | ACCAGACACATACATTAATGAATAT | *EXO1* Sequencing |
| AO3401 | CCGAGTCTCAACTATCTACACAAAT | *EXO1* Sequencing |
| AO3402 | CAAATCACGCAAAGGCCATCACTGC | *EXO1* Sequencing |
| AO3666 | ATGGGTATCCAAGGTCTTCTTCC | *EXO1* Sequencing |
| AO4028 | GACATGGAGGCCCAGAATAC | *EXO1* Sequencing |
| AO3838 | TGGTCGGAAGAGGCATAAATTC | PCR Amplification of *EXO1* |
| AO4061 | TTTAAATTTTTTTCTTTATAGGGCATTATTTGTACT | PCR Amplification of *EXO1* |
| AO4583 | TGCATGGCTAgaaAGAGCAGCCT | *exo1* mutagenesis, *H36E* |
| AO4584 | TAGCCATCAATGGCTAACAC | *exo1* mutagenesis, *H36E* |
| AO4585 | AGCAGCCTGCgaaTGTGCTTATG | *exo1* mutagenesis, *S41E* |
| AO4586 | CTATGTAGCCATGCATAG | *exo1* mutagenesis, *S41E* |
| AO4587 | GTACCTCCAGgaaTTCATAAAAAGATTTAG | *exo1* mutagenesis, *F58E* |
| AO4588 | TTATCAGTTGATTTTCCCATTG | *exo1* mutagenesis, *F58E* |
| AO4589 | GTTTTTCATAgcaAGATTTAGTTTATTGAAAACC | *exo1* mutagenesis, *K61A* |
| AO4590 | GTTTTTCATAgaaAGATTTAGTTTATTGAAAAC | *exo1* mutagenesis, *K61E* |
| AO4591 | TGGAGGTACTTATCAGTTG | *exo1* mutagenesis, *K61A/E* |
| AO4388 | TTTGGTCTTCgctGGTGATGCCA | *exo1* mutagenesis, *D78A* |
| AO4389 | TACGGTTCAACTTTAAAGGTTTTC | *exo1* mutagenesis, *D78A* |
| AO4149 | TGCCATTCCAGTTgctAAGTCTACTG | *exo1* mutagenesis, *K85A* |
| AO4150 | TCACCATCGAAGACCAAATACGG | *exo1* mutagenesis, *K85A* |
| AO4020 | ATTCCAGTTgAAAAGTCTACTG | *exo1* mutagenesis, *K85E* |
| AO4021 | GGCATCACCATCGAAGACCA | *exo1* mutagenesis, *K85E* |
| AO4151 | TACTGAATCTAAAgctAGGGATAAGAG | *exo1* mutagenesis, *R92A* |
| AO4152 | GACTTTTTAACTGGAATGGCATCACC | *exo1* mutagenesis, *R92A* |
| AO4143 | GGACTATTTTCAAgctTGTGTCGAC | *exo1* mutagenesis, *K121A* |
| AO4144 | GGACTATTTTCAAgaaTGTGTCGAC | *exo1* mutagenesis, *K121E* |
| AO4145 | ATAGCATTTTTCTTTTCGCCACAGG | *exo1* mutagenesis, *K121A/E* |
| AO4161 | AATATCCGAAgctTCTGACCTCC | *exo1* mutagenesis, *D171A* |
| AO4162 | ATTCCTTGCACAATGTTTTTC | *exo1* mutagenesis, *D171A* |
| AO4075 | CGAAGATTCTgctCTCCTCGTCTTC | *exo1* mutagenesis, *D173A* |
| AO4076 | GATATTATTCCTTGCACAATG | *exo1* mutagenesis, *D173A* |
| AO4163 | tgctCTCCTCGTCTTCGGATGT | *exo1* mutagenesis, *D171A,D173A* |
| AO4164 | gaagcTTCGGATATTATTCCTTGCAC | *exo1* mutagenesis, *D171A,D173A* |
| AO4146 | ACGTCTCATTACGgctCTGAATGATTAC | *exo1* mutagenesis, *K185A* |
| AO4147 | ACGTCTCATTACGgaaCTGAATGATTAC | *exo1* mutagenesis, *K185E* |
| AO4148 | CGACATCCGAAGACGAGGAG | *exo1* mutagenesis, *K185A/E* |
| AO3887 | TCCCAAGGTTgacCTGATTACCG | *exo1* mutagenesis, *G236D* |
| AO3888 | ATTCCATTTGTATAGTCACAACC | *exo1* mutagenesis, *G236D* |
| AO3885 | AAGAAGCAAAgctgctAATAAACCCTCCATGACTG | *exo1* mutagenesis, *F447A,F448A* (MIP) |
| AO3886 | GTATCCTTCAACGTTTCTTG | *exo1* mutagenesis, *F447A,F448A* (MIP) |
| AO4908 | GTTTTTAATTgaaGTAAGACAGCAAGAC | *rad27* mutagenesis, *A45E* |
| AO4909 | TGATATAGAGACATAGAGGC | *rad27* mutagenesis, *A45E* |
| AO4910 | GTTGACAAAGgctTCTTCAAGAAGGGTGG | *rad27* mutagenesis, *R101A* |
| AO4911 | TCATGAGATTTCAAATCTGG | *rad27* mutagenesis, *R101A* |
| AO4912 | GTCTTCAAGAgctGTGGAAACAGAAAAAAAAC | *rad27* mutagenesis, *R105A* |
| AO4913 | CGCTTTGTCAACTCATGAG | *rad27* mutagenesis, *R105A* |
| AO4914 | AAGATTGGTGgctGTCTCCAAAGAGC | *rad27* mutagenesis, *K130A* |
| AO4915 | CTTTCTTGCTTCATCTTTTC | *rad27* mutagenesis, *K130A* |
| AO4827 | GctACACTCTGTTATAGAACACCCTTC | *rad27* mutagenesis, *D179A* |
| AO4828 | CATATCTTCACTTGCTGCGG | *rad27* mutagenesis, *D179A* |
| AO4916 | CTTGTTGAGAgaaTTGACTTTTTCAG | *rad27* mutagenesis, *H191E* |
| AO4917 | AAGGGTGTTCTATAACAG | *rad27* mutagenesis, *H191E* |
| AO4596 | GATTCAAACCACATCCGCC | *mlh3∆::NATMX* disruption primer set |
| AO4597 | GCTAAGCTCATTCGATTGTAAC | *mlh3∆::NATMX* disruption primer set |
| AO4598 | CTTAGAAAAGTTAGTCTCTGCTGAAC | *mlh3∆::NATMX* disruption primer set |
| AO4599 | CTAATTTTTGAAAGTGCAGTAAGACAG | *mlh3∆::NATMX* disruption primer set |
| AO4059 | ATCCGGCCCGAGAAG | *exo1∆::KANMX* disruption primer set |
| AO4060 | AGACCGCTAGCGGC | *exo1∆::KANMX* disruption primer set |
| AO4061 | TTTAAATTTTTTTCTTTATAGGGCATTATTTGTACT | *exo1∆::KANMX* disruption primer set |
| AO4062 | AAAAAAAAATGTGAATTGCACATGC | *exo1∆::KANMX* disruption primer set |
| AO5196 | CTGTACTATTGGCTTAGCTATG | *rad27Δ::KANMX* disruption primer set |
| AO5197 | TGCGATGGTTCCGATAT | *rad27Δ::KANMX* disruption primer set |
| AO5198 | CAGATACCCGATTGGGCA | *rad27Δ::KANMX* disruption primer set |
| AO5199 | CAGATCTAACAAAGCTAGGTG | *rad27Δ::KANMX* disruption primer set |
| AO4783 | GTCATAAGCTTCCGTCAACCTGCTAGCACTA | PCR amplification of *CDC9* |
| AO4784 | ACTGAGGTACCGGAAAAGGAACTGGAGATACCACA | PCR amplification of *CDC9* |
| AO4785 | TTTGGCTAGAgctgctACTTCCATGAAAAATAAGC | *cdc9* mutagenesis, *F44A,F45A* |
| AO4786 | GTGGCTTGTTTAGGCTTC | *cdc9* mutagenesis, *F44A,F45A* |
| AO4787 | GTCAGAATACgctTACGATGGTGAAAGG | *cdc9* mutagenesis, *K419A* |
| AO4788 | GTAAAAGTTTCTCCTTGAAATC | *cdc9* mutagenesis, *K419A* |
| AO5293 | gctcgaattcGCGGTCTACATTCGCTATC | Hifi assembly of *13MYC* tagged alleles |
| AO5294 | cggggatccgTTTACCTTTATAAACAAATTGGGAAAG | Hifi assembly of *13MYC* tagged alleles |
| AO5295 | taaaggtaaaCGGATCCCCGGGTTAATTAAC | Hifi assembly of *13MYC* tagged alleles |
| AO5296 | tgtagaccgcGAATTCGAGCTCGTTTAAACTG | Hifi assembly of *13MYC* tagged alleles |

AO3144 ACAGCTACCGAATTCTGACTTGCTAGGACATCTTTGCCCACGTTGACCC DNA binding

AO3145 TCGATAGTCTCTAGATAGCATGTCCTAGCAAGTCAGAATTCGGTAGCGTG DNA binding

AO3878 GGGTCAACGTGGGCAAAGATGTCCTAGCAAGTCAGAATTCGGTAGCGTG DNA binding

AO3940 GGGTCAACGTGGGCAAAG DNA binding
