# Supplementary material for: Exo1 protects DNA nicks from ligation to promote crossover formation during meiosis
Source: PLoS Biol. 2023 Apr 20;21(4):e3002085. doi: 10.1371/journal.pbio.3002085 (PMC10153752; doi:10.1371/journal.pbio.3002085)
Supplement: S1 File — (PDF) [file pbio.3002085.s014.pdf]

S1 File. Msh5 peaks pooled from two *exo1Δ* replicates at 5h time point.
Peak start and end positions are indicated.

Chromosome	Start	End	Width (bp)	Fold_enrichment
chrI	4276	4488	213	1.31382
chrI	4548	6042	1495	1.4886
chrI	6193	6653	461	1.40824
chrI	6712	7385	674	1.96069
chrI	8286	9255	970	1.46827
chrI	9310	11619	2310	2.1598
chrI	35135	35476	342	1.4203
chrI	39200	39647	448	1.26941
chrI	41217	45061	3845	3.49348
chrI	50749	52943	2195	2.72006
chrI	58197	58444	248	1.3319
chrI	64692	68990	4299	2.66683
chrI	69128	69404	277	1.32246
chrI	69480	69955	476	1.47223
chrI	70446	70566	121	1.20401
chrI	71209	72468	1260	1.50639
chrI	73076	73335	260	1.29976
chrI	73407	73730	324	1.34162
chrI	74585	75274	690	1.36204
chrI	76382	76651	270	1.28972
chrI	77861	78103	243	1.23347
chrI	79007	81397	2391	3.71092
chrI	82239	83223	985	1.34096
chrI	91823	96672	4850	4.68187
chrI	100766	101032	267	1.30177
chrI	101093	101676	584	1.69953
chrI	108281	108508	228	1.25356
chrI	110358	110689	332	1.24348
chrI	117636	118574	939	1.58503
chrI	125233	125390	158	1.31198
chrI	126069	129681	3613	3.21292
chrI	130033	135522	5490	3.00341
chrI	135600	135713	114	1.28004
chrI	136265	139098	2834	1.29738
chrI	139274	139525	252	1.43436
chrI	139579	139713	135	1.30595
chrI	141556	141897	342	1.79477
chrI	142191	142298	108	1.20168
chrI	143201	143453	253	1.34998
chrI	146233	148410	2178	3.1186
chrI	151079	152323	1245	4.34484
chrI	154080	154254	175	1.23486
chrI	155615	157264	1650	2.83659
chrI	159507	160197	691	1.34749
chrI	166372	166985	614	2.098
chrI	168238	172923	4686	4.1023
chrI	174933	175163	231	1.32513
chrI	175487	176684	1198	2.53725

chrI	179031	179156	126	1.27766
chrI	179261	179915	655	1.44072
chrI	180232	180501	270	1.28431
chrI	181557	182281	725	1.30471
chrI	183993	184470	478	1.33031
chrI	184525	184903	379	1.28502
chrI	184995	185214	220	1.31314
chrI	185860	186785	926	1.48941
chrI	187128	187335	208	1.42933
chrI	189819	190118	300	2.22257
chrI	190717	192107	1391	1.53279
chrI	192556	193317	762	1.42632
chrI	194618	194728	111	1.2415
chrI	195921	196693	773	2.34447
chrI	196947	197410	464	2.25291
chrI	197705	198148	444	1.85823
chrI	198336	198456	121	1.43637
chrI	198712	198860	149	1.68773
chrI	202794	203493	700	1.27755
chrII	41	245	205	1.63838
chrII	9467	9584	118	1.74468
chrII	9708	10037	330	1.40699
chrII	10100	11343	1244	1.64857
chrII	13608	14259	652	1.50668
chrII	24648	24777	130	1.25757
chrII	38541	38666	126	1.27565
chrII	43482	43665	184	1.27766
chrII	43796	44871	1076	1.65516
chrII	44949	45057	109	1.32983
chrII	45270	46858	1589	1.6071
chrII	48278	48458	181	1.20646
chrII	48941	49945	1005	1.41281
chrII	50189	52046	1858	2.32725
chrII	52215	52366	152	1.25557
chrII	63107	63265	159	1.22562
chrII	66566	67973	1408	3.34081
chrII	68855	69797	943	1.56895
chrII	69868	70092	225	1.35802
chrII	70578	72027	1450	2.16993
chrII	72734	73106	373	1.32789
chrII	74310	75079	770	1.8057
chrII	75169	75735	567	1.39952
chrII	78445	78569	125	1.29373
chrII	78642	80739	2098	3.5506
chrII	92368	92630	263	1.30006
chrII	92712	93941	1230	1.77276
chrII	104959	105760	802	1.79395
chrII	115490	119895	4406	1.46418
chrII	126219	126506	288	1.31682
chrII	126615	128070	1456	2.20377
chrII	141838	142030	193	1.21534
chrII	143902	144338	437	1.32142
chrII	145556	146959	1404	4.36036

chrII	147264	147713	450	1.3962
chrII	149408	152167	2760	2.70512
chrII	153160	153372	213	1.20394
chrII	159626	160505	880	8.83918
chrII	161217	161392	176	1.30716
chrII	161491	162220	730	1.55438
chrII	162281	163169	889	1.63419
chrII	170587	171284	698	1.39912
chrII	171399	171652	254	1.43894
chrII	173664	175479	1816	3.62005
chrII	181599	182094	496	1.31928
chrII	183678	183846	169	1.25763
chrII	185339	186373	1035	1.63493
chrII	186436	186755	320	1.17671
chrII	192721	192937	217	1.23617
chrII	193721	196401	2681	1.39719
chrII	196455	197444	990	1.7093
chrII	198134	198413	280	1.56157
chrII	198582	199159	578	1.48756
chrII	205861	206442	582	1.45574
chrII	211005	212997	1993	3.5417
chrII	216075	216405	331	1.19092
chrII	216756	217627	872	1.35786
chrII	218697	218834	138	1.21046
chrII	234860	235410	551	1.49689
chrII	236484	236602	119	1.35484
chrII	236749	237195	447	1.34757
chrII	237249	238907	1659	4.33143
chrII	244173	244641	469	1.46426
chrII	247690	250997	3308	2.84503
chrII	251053	251370	318	1.19109
chrII	251491	252194	704	1.4072
chrII	252323	252504	182	1.32019
chrII	256630	257223	594	1.56923
chrII	267265	267596	332	1.37099
chrII	267903	268023	121	1.24219
chrII	270544	270941	398	1.28369
chrII	278273	278858	586	2.38168
chrII	281444	281747	304	1.34101
chrII	282450	285010	2561	2.22546
chrII	287193	288763	1571	1.61235
chrII	288857	289181	325	1.32371
chrII	290163	290594	432	1.25912
chrII	290663	291300	638	1.42132
chrII	291380	293027	1648	1.3236
chrII	301247	303223	1977	2.00167
chrII	303735	303923	189	1.32387
chrII	304176	306209	2034	1.76784
chrII	308392	309034	643	1.35806
chrII	311516	311832	317	2.12809
chrII	316941	317072	132	1.29976
chrII	319546	321492	1947	2.83055
chrII	321561	321720	160	1.47283

chrII	321798	321906	109	1.28171
chrII	323215	324208	994	1.79797
chrII	324290	324724	435	1.39233
chrII	326977	327106	130	1.39632
chrII	327399	327832	434	1.45204
chrII	330957	332429	1473	1.78422
chrII	332765	332889	125	1.2877
chrII	332951	333074	124	1.35841
chrII	333775	334677	903	2.34713
chrII	337742	337975	234	1.21812
chrII	341191	341366	176	1.19634
chrII	341456	342091	636	2.45289
chrII	342335	342915	581	1.55066
chrII	344245	344716	472	1.40005
chrII	344792	344923	132	1.21981
chrII	349231	350826	1596	1.89312
chrII	350905	352416	1512	1.58542
chrII	353106	353214	109	1.25155
chrII	353303	353544	242	1.47253
chrII	353752	353871	120	1.34798
chrII	356201	356430	230	1.2497
chrII	360261	360608	348	2.37725
chrII	364450	364608	159	1.30579
chrII	364671	364881	211	1.24753
chrII	372131	372694	564	2.65756
chrII	373057	373231	175	1.26087
chrII	390932	391066	135	1.27088
chrII	392721	393372	652	1.50697
chrII	394519	396194	1676	3.14303
chrII	413083	413217	135	1.20962
chrII	413277	414156	880	1.76757
chrII	417898	418024	127	1.24342
chrII	418698	419141	444	1.39112
chrII	419673	419859	187	1.16352
chrII	431003	431251	249	1.37952
chrII	431344	431873	530	1.21494
chrII	434165	434482	318	1.26762
chrII	442503	444422	1920	1.97074
chrII	449050	450774	1725	1.58357
chrII	450828	450990	163	1.24954
chrII	451057	451227	171	1.28168
chrII	452183	452433	251	1.31099
chrII	452781	453467	687	1.34424
chrII	460099	460220	122	1.36204
chrII	460289	461107	819	1.84482
chrII	461162	461403	242	1.22324
chrII	461649	462665	1017	2.35845
chrII	462762	463039	278	1.30579
chrII	466643	466769	127	1.20012
chrII	467133	467255	123	1.2345
chrII	467331	467455	125	1.31772
chrII	468429	468981	553	1.22744
chrII	469052	470594	1543	2.18569

chrII	473286	473684	399	1.30029
chrII	479110	481647	2538	2.19185
chrII	483099	483584	486	1.33089
chrII	486836	486967	132	1.2209
chrII	488272	488408	137	1.25971
chrII	490222	490509	288	1.30579
chrII	490713	491467	755	1.86426
chrII	492591	492864	274	1.38614
chrII	493004	493147	144	1.21849
chrII	493400	493517	118	1.28495
chrII	498703	499981	1279	2.48301
chrII	500653	502450	1798	2.25088
chrII	504368	504880	513	8.70033
chrII	512644	514021	1378	1.5535
chrII	514111	516497	2387	1.59848
chrII	516608	518464	1857	2.98724
chrII	526644	526928	285	1.23497
chrII	530443	530794	352	1.28972
chrII	531258	531430	173	1.20735
chrII	532163	532272	110	1.22382
chrII	532927	533193	267	1.22707
chrII	539835	540436	602	1.17091
chrII	540605	541479	875	2.51752
chrII	541782	542153	372	1.25298
chrII	544422	544993	572	9.938
chrII	548334	548822	489	1.57582
chrII	549877	549985	109	1.20385
chrII	550262	551113	852	1.49442
chrII	562413	562613	201	1.32789
chrII	562697	562888	192	1.48458
chrII	563093	563212	120	1.29976
chrII	564455	564683	229	1.26721
chrII	564790	564916	127	1.20326
chrII	565336	565449	114	1.28109
chrII	566065	566222	158	1.21137
chrII	567267	567394	128	1.21137
chrII	567447	567837	391	1.30378
chrII	569550	569687	138	1.38614
chrII	569844	570386	543	1.53131
chrII	573126	573861	736	1.95266
chrII	581736	581882	147	1.22742
chrII	584390	584817	428	1.36204
chrII	585820	586184	365	1.39771
chrII	586293	586472	180	1.19988
chrII	586545	586793	249	1.38254
chrII	607658	607845	188	1.2013
chrII	608490	609236	747	1.40299
chrII	609674	609790	117	1.26267
chrII	610269	610486	218	1.37565
chrII	611986	612210	225	1.32853
chrII	622499	623071	573	1.2555
chrII	631384	632750	1367	1.92453
chrII	633584	637997	4414	2.52426

chrII	638079	638209	131	1.21338
chrII	641946	642982	1037	2.39182
chrII	643076	643333	258	1.34303
chrII	651692	652047	356	1.34597
chrII	652993	653248	256	1.2221
chrII	654464	656402	1939	3.45532
chrII	656591	656741	151	1.26159
chrII	658144	658965	822	1.6011
chrII	661970	662785	816	2.07212
chrII	662858	663182	325	1.55
chrII	664884	665031	148	1.30893
chrII	666076	666477	402	1.29623
chrII	666558	667155	598	1.23483
chrII	668078	668808	731	2.15771
chrII	672909	674500	1592	2.50912
chrII	674566	674699	134	1.33177
chrII	674944	676871	1928	1.16594
chrII	679421	680089	669	1.49926
chrII	681596	681860	265	1.20749
chrII	689701	690757	1057	1.77587
chrII	694220	698873	4654	1.43169
chrII	702218	702855	638	1.27304
chrII	704296	704473	178	1.41829
chrII	704608	705655	1048	1.50065
chrII	706907	707231	325	1.40325
chrII	707284	708461	1178	1.97368
chrII	708522	708652	131	1.25749
chrII	712894	713502	609	1.41226
chrII	714262	714519	258	1.27871
chrII	717088	717197	110	1.23749
chrII	717259	719028	1770	2.14551
chrII	722724	722859	136	1.22543
chrII	726281	726452	172	1.23314
chrII	731408	731552	145	1.31784
chrII	731638	733386	1749	2.84461
chrII	733913	734671	759	1.46039
chrII	735294	736127	834	1.37862
chrII	736214	736668	455	1.48012
chrII	737579	738217	639	1.32387
chrII	738278	738894	617	1.42244
chrII	739101	739363	263	1.26762
chrII	739590	739875	286	1.27315
chrII	739974	740082	109	1.34798
chrII	740201	740542	342	1.27164
chrII	741787	742691	905	1.61294
chrII	742964	743176	213	1.22711
chrII	744552	744697	146	1.1935
chrII	744809	744923	115	1.23749
chrII	744993	745203	211	1.20735
chrII	745343	746425	1083	1.46523
chrII	750526	752085	1560	2.35042
chrII	760639	761752	1114	4.51359
chrII	761931	763451	1521	2.33234

chrII	776507	776613	107	1.22942
chrII	777638	778530	893	1.41427
chrII	783374	783930	557	1.3969
chrII	784235	784754	520	1.52283
chrII	785335	785868	534	1.29927
chrII	785955	786363	409	1.28186
chrII	786653	786875	223	1.34375
chrII	787064	787273	210	1.28738
chrII	788683	789112	430	1.31663
chrII	789224	789367	144	1.23803
chrII	792028	792399	372	1.23146
chrII	796019	796527	509	1.87632
chrII	796667	797285	619	1.54804
chrII	797385	797719	335	1.25972
chrII	798063	798553	491	1.27582
chrII	801959	802829	871	1.64472
chrIII	11245	11453	209	1.46248
chrIII	14138	14781	644	1.20284
chrIII	28951	29400	450	1.38435
chrIII	29537	30274	738	1.24806
chrIII	30337	30589	253	1.23892
chrIII	33305	33543	239	1.37262
chrIII	33607	34040	434	1.33793
chrIII	37222	38530	1309	1.94462
chrIII	38600	38713	114	1.38858
chrIII	38776	39124	349	1.3699
chrIII	41126	41376	251	1.39402
chrIII	41482	41848	367	1.21828
chrIII	42026	42320	295	1.18624
chrIII	42448	42851	404	1.21678
chrIII	43059	43215	157	1.27094
chrIII	43281	45177	1897	1.83411
chrIII	45232	45496	265	1.27538
chrIII	48778	48899	122	1.25762
chrIII	49420	49628	209	1.22138
chrIII	51373	51638	266	1.43363
chrIII	54219	55624	1406	3.06559
chrIII	56359	57490	1132	1.28066
chrIII	60903	61067	165	1.25492
chrIII	63193	66387	3195	3.49919
chrIII	72995	73140	146	1.25464
chrIII	73209	73891	683	1.39812
chrIII	74177	74354	178	1.23829
chrIII	75422	75857	436	1.2604
chrIII	78704	78881	178	1.26249
chrIII	81982	82373	392	1.69351
chrIII	83202	83542	341	1.17918
chrIII	90957	93328	2372	3.22052
chrIII	95979	96110	132	1.40878
chrIII	99931	102401	2471	2.44641
chrIII	113928	115261	1334	3.32318
chrIII	120875	122365	1491	1.60069
chrIII	130070	131270	1201	2.61752

chrIII	131482	131595	114	1.25078
chrIII	131686	133227	1542	1.66811
chrIII	143968	144763	796	1.55745
chrIII	151386	151523	138	1.69078
chrIII	156957	158326	1370	1.52804
chrIII	162499	162613	115	1.22405
chrIII	163028	163483	456	1.46652
chrIII	172099	173727	1629	1.52567
chrIII	190027	192217	2191	2.08169
chrIII	193331	195093	1763	2.22464
chrIII	201215	201322	108	1.25757
chrIII	202046	202215	170	1.2415
chrIII	205120	205266	147	1.23146
chrIII	205505	206349	845	1.34373
chrIII	206413	207296	884	1.54485
chrIII	207541	207800	260	1.44842
chrIII	207867	208033	167	1.43911
chrIII	208125	208365	241	1.31181
chrIII	209392	209592	201	1.38815
chrIII	209826	213121	3296	2.58747
chrIII	216610	221028	4419	3.53366
chrIII	222264	222473	210	1.2174
chrIII	222794	223092	299	1.31985
chrIII	223154	223309	156	1.24351
chrIII	223646	225626	1981	1.56203
chrIII	225710	225931	222	1.37007
chrIII	228317	228803	487	1.3778
chrIII	228888	230148	1261	1.74198
chrIII	230840	234499	3660	3.71647
chrIII	235294	235952	659	1.49812
chrIII	236007	236836	830	5.41131
chrIII	238645	239015	371	1.83511
chrIII	239517	243343	3827	2.06457
chrIII	244031	244149	119	1.22141
chrIII	244211	244418	208	1.23347
chrIII	244521	245092	572	1.45646
chrIII	245170	245591	422	1.24235
chrIII	246009	247333	1325	3.19216
chrIII	248104	249215	1112	1.64528
chrIII	249339	250605	1267	1.70154
chrIII	252564	252834	271	1.46047
chrIII	257850	258491	642	1.53681
chrIII	258851	260601	1751	3.01217
chrIII	262551	263073	523	5.4365
chrIII	272090	273343	1254	2.02498
chrIII	273396	273801	406	1.41628
chrIII	273873	274078	206	1.25767
chrIII	275796	278242	2447	2.5493
chrIII	286394	286778	385	1.2636
chrIII	286850	286978	129	1.33592
chrIII	287687	288958	1272	1.95868
chrIII	290647	291573	927	1.54686
chrIII	291631	291951	321	1.48056

chrIII	292119	293020	902	1.86828
chrIII	293724	294389	666	1.77898
chrIII	294664	295014	351	1.52677
chrIII	295287	295413	127	1.47654
chrIII	296098	297267	1170	1.7156
chrIII	298775	299033	259	1.26561
chrIII	300316	300568	253	1.33994
chrIII	306693	306800	108	1.17884
chrIII	308595	308768	174	1.13584
chrIII	313524	313643	120	1.18849
chrIV	19438	19859	422	1.33029
chrIV	20122	20241	120	1.32136
chrIV	20636	20888	253	1.3982
chrIV	25175	27018	1844	3.76469
chrIV	31724	31875	152	1.37007
chrIV	31950	32068	119	1.26736
chrIV	32128	32359	232	1.3046
chrIV	32423	32768	346	1.44709
chrIV	32929	33292	364	1.38012
chrIV	33468	34108	641	1.39095
chrIV	38196	39083	888	1.93749
chrIV	39139	39245	107	1.22788
chrIV	42254	42757	504	8.6734
chrIV	45920	48483	2564	4.18549
chrIV	49851	50455	605	1.51471
chrIV	50547	51119	573	1.85422
chrIV	52713	52838	126	1.235
chrIV	53059	54773	1715	3.79398
chrIV	61541	62106	566	1.33212
chrIV	62385	62554	170	1.2517
chrIV	65283	65618	336	1.3138
chrIV	65853	66227	375	1.1923
chrIV	66307	66516	210	1.24769
chrIV	70419	70907	489	1.23058
chrIV	87787	88038	252	1.42431
chrIV	91793	93977	2185	2.34238
chrIV	94054	94392	339	1.16802
chrIV	94625	94754	130	1.26159
chrIV	95502	96313	812	1.89038
chrIV	98076	98522	447	3.48235
chrIV	99783	99896	114	1.20878
chrIV	104259	104479	221	1.32963
chrIV	116875	117300	426	1.28502
chrIV	117444	117694	251	1.47104
chrIV	117770	118154	385	2.20915
chrIV	118513	119207	695	2.22588
chrIV	129798	130385	588	1.62923
chrIV	132801	132940	140	1.39453
chrIV	143311	145056	1746	3.09773
chrIV	145119	145542	424	1.29655
chrIV	155380	156855	1476	3.23022
chrIV	157396	158012	617	1.26594
chrIV	158227	159922	1696	1.67243

chrIV	160547	161461	915	1.59105
chrIV	162772	163367	596	1.50266
chrIV	163461	165199	1739	1.7394
chrIV	167024	167295	272	1.3683
chrIV	169400	169771	372	1.71159
chrIV	169953	170278	326	1.37208
chrIV	171798	172004	207	1.33793
chrIV	180990	181889	900	1.68949
chrIV	187769	189049	1281	1.99484
chrIV	189102	189326	225	1.27645
chrIV	191213	191386	174	1.25309
chrIV	192320	192958	639	1.21197
chrIV	193198	193328	131	1.27164
chrIV	193578	194929	1352	3.3458
chrIV	197970	199779	1810	1.94663
chrIV	204243	205604	1362	2.60354
chrIV	210843	210966	124	1.21539
chrIV	212309	212764	456	1.56616
chrIV	217716	217977	262	1.31988
chrIV	227376	227486	111	1.32473
chrIV	229992	230205	214	1.3599
chrIV	230618	231111	494	1.5107
chrIV	231536	232810	1275	2.28011
chrIV	233707	233999	293	1.31063
chrIV	234067	234589	523	1.17529
chrIV	235469	236395	927	1.7237
chrIV	242454	242571	118	1.29947
chrIV	245157	245328	172	1.27592
chrIV	252889	254628	1740	2.08953
chrIV	255690	257023	1334	1.53681
chrIV	262240	264998	2759	4.77827
chrIV	269596	270485	890	1.33568
chrIV	272522	272761	240	1.23414
chrIV	279562	281198	1637	2.14752
chrIV	281421	281721	301	1.25411
chrIV	285116	285245	130	1.25078
chrIV	292105	293707	1603	1.45182
chrIV	297248	298604	1357	1.71373
chrIV	302366	302743	378	1.56842
chrIV	303218	303750	533	1.37533
chrIV	306238	306929	692	2.19573
chrIV	308356	308596	241	1.22226
chrIV	309389	310198	810	2.02096
chrIV	310560	310683	124	1.34565
chrIV	311208	311372	165	1.28972
chrIV	311662	312735	1074	1.67751
chrIV	313049	313269	221	1.24351
chrIV	313352	313810	459	1.26019
chrIV	313896	315624	1729	2.21233
chrIV	331303	332464	1162	1.58505
chrIV	332823	333639	817	1.70584
chrIV	334061	334381	321	1.3065
chrIV	335786	336780	995	1.40041

chrIV	337964	338950	987	1.60639
chrIV	339163	339279	117	1.20534
chrIV	354357	357312	2956	2.27408
chrIV	357417	357540	124	1.33606
chrIV	372235	373088	854	1.52723
chrIV	373355	374168	814	1.42833
chrIV	374226	374333	108	1.25512
chrIV	375948	376327	380	1.28215
chrIV	376406	377265	860	1.48604
chrIV	377390	378326	937	1.43035
chrIV	378395	378758	364	1.36769
chrIV	385290	385864	575	1.56816
chrIV	386217	386405	189	1.5183
chrIV	386523	386970	448	2.28973
chrIV	387051	388347	1297	1.49744
chrIV	400144	401746	1603	3.02202
chrIV	413381	413504	124	1.42782
chrIV	413561	415267	1707	1.91923
chrIV	425588	425697	110	1.20386
chrIV	433929	434038	110	1.23818
chrIV	436593	436818	226	1.21238
chrIV	439919	441323	1405	1.90532
chrIV	441386	441492	107	1.19202
chrIV	446541	446668	128	1.21616
chrIV	446762	446895	134	1.28699
chrIV	448849	448960	112	1.23764
chrIV	449026	450110	1085	4.96506
chrIV	456237	457380	1144	1.39888
chrIV	465162	465559	398	2.72817
chrIV	470122	470248	127	1.26159
chrIV	470469	470833	365	1.37572
chrIV	470942	471178	237	1.22839
chrIV	475369	476425	1057	2.32229
chrIV	476487	476656	170	1.41486
chrIV	491641	491862	222	1.45854
chrIV	492280	492753	474	1.70093
chrIV	507673	507917	245	1.19791
chrIV	508053	508259	207	1.30008
chrIV	508995	510667	1673	1.95868
chrIV	538304	539276	973	6.91541
chrIV	548411	548532	122	1.20661
chrIV	550205	553289	3085	2.52894
chrIV	554811	555986	1176	2.01306
chrIV	556053	556502	450	1.36002
chrIV	578237	578500	264	1.19117
chrIV	582232	582542	311	1.45596
chrIV	582595	582972	378	1.30509
chrIV	583854	584466	613	1.57407
chrIV	584665	585062	398	1.50357
chrIV	591247	591360	114	1.22399
chrIV	591652	591860	209	1.3823
chrIV	593797	594454	658	1.23748
chrIV	597268	597382	115	1.34642

chrIV	601686	601792	107	1.26459
chrIV	601918	602036	119	1.30729
chrIV	602328	602449	122	1.27159
chrIV	606216	608031	1816	3.54371
chrIV	611950	613247	1298	1.42467
chrIV	613643	614146	504	1.34357
chrIV	615570	615713	144	1.2569
chrIV	615780	616169	390	1.19158
chrIV	616280	617086	807	1.17339
chrIV	617165	617274	110	1.23445
chrIV	621041	621798	758	1.26497
chrIV	621859	622651	793	1.62406
chrIV	622768	622947	180	1.2922
chrIV	623491	625249	1759	1.88899
chrIV	627704	628608	905	1.78319
chrIV	629093	629601	509	1.39713
chrIV	630211	630393	183	1.2182
chrIV	630570	632185	1616	1.66123
chrIV	636862	637235	374	1.31712
chrIV	637310	637581	272	1.24436
chrIV	639299	641059	1761	3.0676
chrIV	652988	653099	112	1.22949
chrIV	654563	654760	198	1.43637
chrIV	655036	655410	375	1.42166
chrIV	655512	656544	1033	1.45889
chrIV	657070	657862	793	1.95674
chrIV	657933	658040	108	1.22673
chrIV	660249	661795	1547	2.24214
chrIV	668514	668656	143	1.30579
chrIV	672842	673837	996	1.55891
chrIV	674958	675649	692	1.19941
chrIV	676029	676642	614	1.40422
chrIV	676888	678034	1147	1.86225
chrIV	678142	678373	232	1.36003
chrIV	697631	701427	3797	3.8481
chrIV	703775	705107	1333	1.55541
chrIV	712282	714160	1879	3.02571
chrIV	715039	715396	358	1.41418
chrIV	715748	716126	379	1.31307
chrIV	720958	721203	246	1.26494
chrIV	730670	730788	119	1.2149
chrIV	733367	735459	2093	2.00669
chrIV	748578	749015	438	1.25398
chrIV	751877	752668	792	1.33391
chrIV	752860	753052	193	1.19537
chrIV	753112	753349	238	1.62319
chrIV	753503	753651	149	1.2174
chrIV	756015	756261	247	1.21137
chrIV	763307	764799	1493	2.49707
chrIV	764881	765156	276	1.26824
chrIV	765215	765742	528	3.6076
chrIV	765803	766913	1111	1.7136
chrIV	767705	767815	111	1.27365

chrIV	767877	768071	195	1.27967
chrIV	768530	769002	473	1.25162
chrIV	769709	769880	172	1.25607
chrIV	770086	770224	139	1.62594
chrIV	770279	770683	405	1.52496
chrIV	779003	780354	1352	1.56293
chrIV	780643	780762	120	1.36806
chrIV	781134	782518	1385	1.2154
chrIV	783643	784090	448	1.415
chrIV	784167	784292	126	1.39369
chrIV	784350	786207	1858	1.48318
chrIV	791273	792745	1473	2.53524
chrIV	793575	793748	174	1.30005
chrIV	805893	806046	154	1.22098
chrIV	806271	806408	138	1.39751
chrIV	810593	811044	452	1.28306
chrIV	818189	818416	228	1.21936
chrIV	825285	826738	1454	1.87228
chrIV	828799	829923	1125	1.50144
chrIV	830005	836950	6946	3.16788
chrIV	839132	839417	286	1.20377
chrIV	839790	841101	1312	2.46493
chrIV	842455	842713	259	1.2202
chrIV	843032	843422	391	1.29574
chrIV	843668	843786	119	1.30177
chrIV	845254	845744	491	1.35971
chrIV	853673	853858	186	1.26762
chrIV	853930	854247	318	1.35324
chrIV	861896	862542	647	1.39128
chrIV	862713	863031	319	1.28732
chrIV	863156	863390	235	1.28369
chrIV	863475	865289	1815	2.83055
chrIV	894025	894445	421	1.7307
chrIV	894532	895154	623	1.22031
chrIV	895215	895380	166	1.31966
chrIV	898822	900595	1774	3.60024
chrIV	921109	922617	1509	2.0496
chrIV	926643	926767	125	1.26991
chrIV	928848	932420	3573	2.47296
chrIV	934637	934793	157	1.38572
chrIV	934878	935068	191	1.26844
chrIV	936860	938277	1418	2.47104
chrIV	955159	956264	1106	1.76281
chrIV	957445	957610	166	1.26871
chrIV	957753	957945	193	1.4922
chrIV	958009	958185	177	1.29612
chrIV	959423	959545	123	1.26158
chrIV	959614	959853	240	1.2986
chrIV	959986	961105	1120	1.50458
chrIV	962912	964431	1520	2.0802
chrIV	965047	966000	954	1.22689
chrIV	967404	967544	141	1.27092
chrIV	967608	967987	380	1.21606

chrIV	993113	993969	857	1.53327
chrIV	994029	994932	904	1.46047
chrIV	1004703	1005945	1243	1.83725
chrIV	1013108	1013657	550	1.33043
chrIV	1016906	1017134	229	1.40582
chrIV	1017353	1017476	124	1.34661
chrIV	1021370	1021523	154	1.23361
chrIV	1021750	1021939	190	1.30851
chrIV	1022006	1023102	1097	1.75217
chrIV	1023506	1023954	449	1.78764
chrIV	1024530	1025013	484	1.21271
chrIV	1025239	1025351	113	1.28175
chrIV	1042318	1044183	1866	2.64744
chrIV	1044252	1045752	1501	1.48707
chrIV	1049289	1049481	193	1.1968
chrIV	1049771	1050228	458	1.3607
chrIV	1053712	1054084	373	1.32789
chrIV	1054182	1054413	232	1.22744
chrIV	1054487	1054605	119	1.33897
chrIV	1054663	1054928	266	1.4403
chrIV	1055220	1056055	836	1.28124
chrIV	1056113	1057072	960	2.03741
chrIV	1057577	1058413	837	1.46851
chrIV	1058516	1058639	124	1.24223
chrIV	1060912	1061239	328	1.33793
chrIV	1066929	1069486	2558	2.26522
chrIV	1077495	1080920	3426	2.24486
chrIV	1081135	1081332	198	1.28268
chrIV	1087092	1087364	273	1.77908
chrIV	1088832	1089814	983	1.83413
chrIV	1101759	1101908	150	1.34798
chrIV	1101967	1102222	256	1.29574
chrIV	1105314	1106228	915	1.74124
chrIV	1106301	1106518	218	1.29493
chrIV	1106647	1106766	120	1.19755
chrIV	1110285	1110500	216	1.29775
chrIV	1111410	1111518	109	1.22744
chrIV	1111575	1111776	202	1.2415
chrIV	1119671	1121249	1579	2.75823
chrIV	1132633	1133432	800	1.3859
chrIV	1140956	1141432	477	1.35594
chrIV	1144773	1145549	777	1.65577
chrIV	1146007	1146368	362	1.27765
chrIV	1146446	1147874	1429	1.7598
chrIV	1149612	1150545	934	1.46302
chrIV	1151674	1152022	349	1.57096
chrIV	1153067	1153805	739	1.26191
chrIV	1153902	1154083	182	1.39016
chrIV	1161415	1161618	204	1.47848
chrIV	1161680	1161892	213	1.47491
chrIV	1162175	1163073	899	1.42553
chrIV	1164533	1164695	163	1.37409
chrIV	1165421	1165839	419	1.35244

chrIV	1166148	1167394	1247	1.74454
chrIV	1168234	1168470	237	1.28203
chrIV	1168568	1168674	107	1.25331
chrIV	1168777	1168936	160	1.21569
chrIV	1169050	1170161	1112	1.42291
chrIV	1189528	1190051	524	2.92071
chrIV	1190517	1190631	115	1.20029
chrIV	1190808	1192336	1529	2.67707
chrIV	1201222	1201678	457	1.28972
chrIV	1212951	1213063	113	1.20999
chrIV	1213186	1213337	152	1.22227
chrIV	1213830	1214286	457	1.27069
chrIV	1220663	1221267	605	1.33793
chrIV	1233866	1233983	118	1.35092
chrIV	1234164	1234287	124	1.18856
chrIV	1236532	1236945	414	1.39827
chrIV	1237015	1237571	557	1.23923
chrIV	1237704	1238249	546	1.47757
chrIV	1238350	1238502	153	1.41354
chrIV	1238579	1238934	356	1.40297
chrIV	1241018	1241237	220	1.29373
chrIV	1241531	1242537	1007	1.68266
chrIV	1247581	1248660	1080	1.74373
chrIV	1252005	1252916	912	1.40636
chrIV	1253933	1255901	1969	3.22273
chrIV	1263203	1263718	516	1.46894
chrIV	1265803	1266559	757	1.6542
chrIV	1266642	1266901	260	1.26823
chrIV	1267407	1267633	227	1.19658
chrIV	1268808	1271392	2585	2.44481
chrIV	1271478	1273698	2221	1.93658
chrIV	1273780	1273961	182	1.21696
chrIV	1278562	1280171	1610	2.60621
chrIV	1283384	1284974	1591	3.5196
chrIV	1288591	1288932	342	1.29815
chrIV	1289389	1289636	248	1.22017
chrIV	1291889	1293165	1277	1.76375
chrIV	1294067	1294547	481	1.57745
chrIV	1294628	1295040	413	1.41996
chrIV	1295538	1295797	260	1.3161
chrIV	1311935	1312211	277	1.22195
chrIV	1314064	1315945	1882	3.49721
chrIV	1322228	1322574	347	1.29399
chrIV	1323262	1324463	1202	1.57929
chrIV	1329073	1329278	206	1.31784
chrIV	1332504	1333308	805	1.44239
chrIV	1333702	1334001	300	1.21941
chrIV	1334087	1334212	126	1.25356
chrIV	1338575	1338764	190	1.23937
chrIV	1339335	1339837	503	1.39108
chrIV	1340265	1341391	1127	1.57072
chrIV	1341975	1342102	128	1.34155
chrIV	1343131	1345585	2455	1.57124

chrIV	1357332	1357472	141	1.3078
chrIV	1357599	1357854	256	1.2636
chrIV	1379234	1380612	1379	3.96759
chrIV	1385211	1385466	256	1.19653
chrIV	1395189	1395461	273	1.37007
chrIV	1397565	1399981	2417	1.49103
chrIV	1402953	1403148	196	1.34374
chrIV	1404020	1404156	137	1.23749
chrIV	1404263	1404543	281	1.19417
chrIV	1406973	1407365	393	1.56092
chrIV	1413846	1415211	1366	2.00633
chrIV	1415919	1416326	408	1.30579
chrIV	1416476	1416594	119	1.30378
chrIV	1416649	1416889	241	1.25159
chrIV	1417053	1417188	136	1.25424
chrIV	1417292	1417758	467	1.54284
chrIV	1418008	1418763	756	1.45445
chrIV	1419844	1420970	1127	1.63658
chrIV	1421079	1421195	117	1.37409
chrIV	1421260	1421528	269	1.46966
chrIV	1421766	1421876	111	1.23146
chrIV	1422063	1422180	118	1.30981
chrIV	1422340	1422702	363	1.26561
chrIV	1423751	1425481	1731	2.65376
chrIV	1433942	1435764	1823	3.32716
chrIV	1435923	1436201	279	1.2704
chrIV	1436314	1436516	203	1.26383
chrIV	1437393	1439191	1799	2.65577
chrIV	1449885	1450325	441	1.61038
chrIV	1450442	1450727	286	1.42896
chrIV	1452242	1453555	1314	1.91477
chrIV	1453883	1454501	619	1.46415
chrIV	1455981	1456911	931	1.87431
chrIV	1456996	1457304	309	1.41025
chrIV	1457658	1458086	429	1.22342
chrIV	1458686	1459437	752	1.32642
chrIV	1461895	1462298	404	1.47454
chrIV	1467908	1468641	734	1.40941
chrIV	1474467	1474798	332	1.30981
chrIV	1474934	1475651	718	1.44842
chrIV	1476082	1477676	1595	1.90076
chrIV	1480189	1481365	1177	1.43589
chrIV	1484876	1485414	539	1.36831
chrIV	1487097	1487345	249	1.25679
chrIV	1489385	1489640	256	1.30018
chrIV	1490113	1490371	259	1.65438
chrIV	1495476	1496398	923	1.4011
chrIV	1499437	1499618	182	1.242
chrIV	1502812	1502970	159	1.30039
chrIX	28782	28925	144	1.20253
chrIX	29852	30075	224	1.2636
chrIX	30134	30756	623	1.63726
chrIX	30861	31064	204	1.35609

chrIX	32362	33169	808	2.57515
chrIX	33232	33616	385	1.90033
chrIX	36400	36604	205	1.31818
chrIX	37058	37465	408	1.16045
chrIX	38906	39043	138	1.3519
chrIX	41600	41734	135	1.54491
chrIX	41832	42192	361	1.32402
chrIX	45351	45731	381	1.31728
chrIX	46133	47227	1095	1.57768
chrIX	50482	54368	3887	3.58188
chrIX	54753	55043	291	1.44239
chrIX	55150	55574	425	1.33994
chrIX	56282	57736	1455	1.66739
chrIX	78529	78763	235	1.29732
chrIX	79058	79223	166	1.2506
chrIX	79727	81466	1740	2.39863
chrIX	85068	86067	1000	1.50638
chrIX	87180	89269	2090	2.28619
chrIX	89337	89996	660	1.21105
chrIX	94617	94875	259	1.26159
chrIX	96984	97164	181	1.29574
chrIX	102280	102636	357	1.99385
chrIX	104851	105178	328	1.20333
chrIX	105441	106883	1443	3.46737
chrIX	126215	126400	186	1.27164
chrIX	130473	130596	124	1.32113
chrIX	131620	131820	201	1.2905
chrIX	131979	132568	590	1.34727
chrIX	133929	137013	3085	2.55872
chrIX	137258	137660	403	1.27114
chrIX	141329	141528	200	1.24909
chrIX	141753	141997	245	1.18298
chrIX	142070	142445	376	1.32252
chrIX	143511	144922	1412	2.13547
chrIX	154692	157833	3142	2.14149
chrIX	160640	160767	128	1.39548
chrIX	160846	161494	649	1.4406
chrIX	162547	163575	1029	1.82208
chrIX	167119	167650	532	1.39911
chrIX	175272	175405	134	1.34597
chrIX	181854	182835	982	1.52648
chrIX	183575	183689	115	1.2093
chrIX	185727	188024	2298	1.89205
chrIX	188100	188532	433	1.27077
chrIX	190864	191527	664	1.36437
chrIX	191599	192090	492	1.38312
chrIX	195101	196007	907	1.68748
chrIX	214150	215039	890	1.67132
chrIX	217137	218078	942	2.02306
chrIX	227496	227641	146	1.331
chrIX	228869	230123	1255	1.52537
chrIX	230208	230480	273	1.21751
chrIX	232097	232341	245	1.20783

chrIX	248946	249494	549	2.38889
chrIX	249926	250059	134	1.30014
chrIX	251277	252760	1484	2.26087
chrIX	255947	256123	177	1.21573
chrIX	264160	266651	2492	3.10978
chrIX	267448	269333	1886	2.40123
chrIX	270267	270861	595	1.4123
chrIX	270934	273548	2615	1.8281
chrIX	273604	273723	120	1.23749
chrIX	274636	274780	145	1.27766
chrIX	276999	277237	239	1.26762
chrIX	277695	277824	130	1.32101
chrIX	278062	278295	234	1.25048
chrIX	279079	280667	1589	2.22185
chrIX	287115	288514	1400	1.95868
chrIX	289906	290101	196	1.25155
chrIX	291509	291754	246	1.2762
chrIX	292493	293430	938	1.46383
chrIX	293488	294777	1290	1.48458
chrIX	295244	296918	1675	3.76263
chrIX	299337	300218	882	1.61293
chrIX	300652	302160	1509	1.91753
chrIX	303605	304409	805	1.47839
chrIX	304467	304655	189	1.22519
chrIX	316733	317165	433	1.50522
chrIX	317850	318685	836	2.61961
chrIX	326633	326809	177	1.21762
chrIX	326922	328741	1820	2.43479
chrIX	330916	334103	3188	3.05989
chrIX	334285	334523	239	1.23808
chrIX	334643	334941	299	1.29002
chrIX	335138	336092	955	1.49191
chrIX	336525	341542	5018	2.34053
chrIX	341716	342911	1196	1.57588
chrIX	347732	348384	653	1.52491
chrIX	349060	351251	2192	2.13797
chrIX	355181	356237	1057	3.56214
chrIX	364424	365727	1304	1.72381
chrIX	376996	377641	646	1.34742
chrIX	382243	382526	284	2.07709
chrIX	383543	383669	127	1.22826
chrIX	385368	385635	268	1.25628
chrIX	385846	386146	301	1.34335
chrIX	389602	389909	308	1.47742
chrIX	390005	390278	274	1.58333
chrIX	393565	393690	126	1.28463
chrIX	395001	395328	328	1.4287
chrIX	402616	402999	384	1.26159
chrIX	415607	415997	391	2.96936
chrIX	419204	420019	816	2.0355
chrV	17679	18284	606	1.3619
chrV	18545	18685	141	1.24036
chrV	24927	26000	1074	1.57096

chrV	26091	26261	171	1.37811
chrV	26402	30411	4010	2.10925
chrV	33227	33470	244	1.38197
chrV	33674	34179	506	3.78815
chrV	35386	37002	1617	2.39881
chrV	40092	40432	341	1.27001
chrV	41592	42598	1007	19.03911
chrV	45134	45262	129	1.33173
chrV	47961	48893	933	1.29533
chrV	50732	51106	375	1.22222
chrV	51864	52783	920	1.57699
chrV	53092	53276	185	1.199
chrV	54320	54606	287	1.301
chrV	59027	59630	604	1.87632
chrV	66566	66822	257	1.30378
chrV	66936	67110	175	1.32186
chrV	68310	68926	617	1.55717
chrV	69025	70215	1191	1.73276
chrV	73563	73721	159	1.32012
chrV	76976	79541	2566	2.04217
chrV	79887	79994	108	1.38029
chrV	96117	97387	1271	2.19413
chrV	97656	97871	216	1.22927
chrV	100466	100950	485	1.52994
chrV	101190	102591	1402	1.91017
chrV	108326	108463	138	1.25155
chrV	114741	116150	1410	1.38577
chrV	117140	117523	384	1.34791
chrV	117621	118121	501	1.6822
chrV	118370	118519	150	1.33994
chrV	122886	123547	662	5.8768
chrV	130766	130925	160	1.28206
chrV	133111	133251	141	1.21166
chrV	133326	133553	228	1.23579
chrV	134813	135421	609	1.94618
chrV	135492	135598	107	1.37011
chrV	135978	136374	397	1.28717
chrV	137576	138222	647	1.40942
chrV	138478	138600	123	1.25418
chrV	138918	139124	207	1.39131
chrV	139688	140019	332	1.30826
chrV	140073	140322	250	1.69538
chrV	145645	146019	375	1.52661
chrV	146091	146291	201	1.2858
chrV	148332	148600	269	1.32004
chrV	148704	148921	218	1.18669
chrV	151469	152668	1200	3.20768
chrV	155806	156337	532	1.31853
chrV	156753	156993	241	1.34108
chrV	157265	157741	477	1.61375
chrV	157823	158059	237	1.33602
chrV	158131	158338	208	1.42676
chrV	158553	159161	609	1.43923

chrV	160052	160332	281	1.30864
chrV	160463	160737	275	1.34022
chrV	160801	161169	369	1.27684
chrV	162871	163142	272	1.29391
chrV	163954	164874	921	1.75845
chrV	165144	167636	2493	1.84767
chrV	186237	187392	1156	2.22517
chrV	187494	187919	426	1.5511
chrV	187976	190139	2164	2.49707
chrV	198738	199015	278	1.22957
chrV	199090	199241	152	1.27638
chrV	202164	202751	588	1.27099
chrV	205359	207336	1978	2.68189
chrV	207677	207917	241	1.25521
chrV	208028	208606	579	1.44364
chrV	212313	212420	108	1.19324
chrV	212523	212779	257	1.26141
chrV	213620	213847	228	1.22284
chrV	213922	214120	199	1.21941
chrV	215344	215473	130	1.22744
chrV	216787	218719	1933	4.01641
chrV	222713	222878	166	1.29832
chrV	222970	223105	136	1.20419
chrV	223263	223456	194	1.32993
chrV	225035	225265	231	1.23887
chrV	225407	225710	304	1.89567
chrV	225897	226573	677	1.53251
chrV	229254	229406	153	1.34396
chrV	231831	232174	344	1.23949
chrV	234451	236364	1914	2.93824
chrV	236468	238088	1621	2.02366
chrV	242658	242784	127	1.25183
chrV	243183	243600	418	1.5589
chrV	253315	253584	270	1.25356
chrV	255468	257125	1658	2.47515
chrV	257218	258076	859	1.29123
chrV	260832	261076	245	1.25135
chrV	264636	264845	210	1.24603
chrV	267982	268243	262	1.36393
chrV	273440	274973	1534	1.90785
chrV	307323	308979	1657	2.48501
chrV	309119	309785	667	1.38012
chrV	310866	311883	1018	1.22721
chrV	319078	319291	214	1.35601
chrV	319376	319592	217	1.30887
chrV	322189	322542	354	1.34595
chrV	323869	324816	948	2.1435
chrV	324877	325152	276	1.22744
chrV	325443	325824	382	1.33592
chrV	327751	328164	414	1.41583
chrV	328731	328853	123	1.26656
chrV	330119	330498	380	1.25336
chrV	331531	333319	1789	2.1484

chrV	334816	335653	838	1.43767
chrV	335738	335854	117	1.23859
chrV	338843	340149	1307	1.94663
chrV	357529	358039	511	1.38213
chrV	359803	360926	1124	1.51873
chrV	366571	368637	2067	2.46737
chrV	372898	373258	361	1.3958
chrV	373404	373961	558	1.6011
chrV	377690	378844	1155	1.33088
chrV	387795	388076	282	1.27967
chrV	390444	390550	107	1.21053
chrV	397669	398075	407	1.34195
chrV	402614	402787	174	1.34396
chrV	402858	402980	123	1.32387
chrV	403216	403332	117	1.24351
chrV	406262	406401	140	1.22977
chrV	406468	407066	599	1.48129
chrV	407138	407887	750	1.39573
chrV	408176	408423	248	1.29327
chrV	408763	409418	656	1.41747
chrV	409825	410209	385	1.4748
chrV	412706	413948	1243	1.39377
chrV	414009	414243	235	1.28842
chrV	414459	414923	465	1.40849
chrV	415954	416102	149	1.33659
chrV	416326	417203	878	1.24406
chrV	420018	421858	1841	3.11348
chrV	422720	422923	204	1.28923
chrV	423402	423954	553	1.65936
chrV	424269	425476	1208	1.68817
chrV	434063	434179	117	1.27365
chrV	434362	434526	165	1.79797
chrV	449685	450364	680	1.7136
chrV	452796	454042	1247	1.57713
chrV	454777	455079	303	1.22938
chrV	455140	455298	159	1.25642
chrV	455382	455501	120	1.29004
chrV	457113	458550	1438	2.37289
chrV	461155	462419	1265	2.15141
chrV	462547	462937	391	1.30592
chrV	463056	463381	326	1.36478
chrV	465768	467037	1270	2.01
chrV	467454	468126	673	1.21506
chrV	468678	469148	471	1.6915
chrV	473759	475070	1312	1.54887
chrV	475128	475290	163	1.35601
chrV	475483	475598	116	1.23749
chrV	475711	476861	1151	2.00742
chrV	476925	477113	189	1.19803
chrV	482483	482735	253	1.29976
chrV	482810	483162	353	1.51727
chrV	483321	483563	243	1.33391
chrV	483618	484417	800	1.37292

chrV	484778	484996	219	1.3131
chrV	486468	487326	859	1.26777
chrV	487479	487802	324	1.59434
chrV	488231	489007	777	1.68711
chrV	490389	490825	437	1.20135
chrV	490956	491085	130	1.27544
chrV	517762	517868	107	1.28735
chrV	520332	523914	3583	4.20852
chrV	525861	526580	720	1.2659
chrV	526971	527227	257	1.2636
chrV	527447	527897	451	1.27164
chrV	528019	528219	201	1.24722
chrV	528321	530448	2128	3.90531
chrV	545214	545511	298	2.3581
chrV	548403	549573	1171	1.52009
chrV	549627	550821	1195	1.73812
chrV	554080	554344	265	1.41269
chrV	554518	554884	367	1.32361
chrV	555123	556007	885	1.59306
chrV	556068	556347	280	1.25356
chrV	558696	559613	918	13.4648
chrVI	30139	31592	1454	1.42075
chrVI	31756	32839	1084	1.29102
chrVI	32988	33438	451	1.56895
chrVI	35946	37406	1461	1.46458
chrVI	38115	39574	1460	1.64328
chrVI	39957	40065	109	1.35199
chrVI	42012	45180	3169	2.41872
chrVI	48736	48989	254	1.29775
chrVI	50872	53469	2598	2.17025
chrVI	54510	55285	776	3.07307
chrVI	58940	59227	288	1.23428
chrVI	59333	59446	114	1.21137
chrVI	62698	63833	1136	1.99082
chrVI	64560	64810	251	1.20424
chrVI	67826	69854	2029	4.90976
chrVI	74031	77720	3690	3.57561
chrVI	78124	78846	723	1.37208
chrVI	79115	79383	269	1.19514
chrVI	79492	79600	109	1.20291
chrVI	79655	79888	234	1.28004
chrVI	80005	80463	459	1.4874
chrVI	82212	82355	144	1.28249
chrVI	82408	82821	414	1.28739
chrVI	82891	83010	120	1.43235
chrVI	83180	84072	893	2.51314
chrVI	84127	84962	836	2.04707
chrVI	85087	85232	146	1.21941
chrVI	86232	86350	119	1.23949
chrVI	86691	87156	466	1.32555
chrVI	87426	87549	124	1.19643
chrVI	92645	93218	574	1.579
chrVI	93285	99246	5962	2.4127

chrVI	100096	100311	216	1.25363
chrVI	100604	100738	135	1.41025
chrVI	110908	111126	219	1.20277
chrVI	111458	111627	170	1.28837
chrVI	111987	112312	326	1.26442
chrVI	112437	112630	194	1.30896
chrVI	112895	113019	125	1.21539
chrVI	113271	113528	258	1.32789
chrVI	113809	114546	738	1.38107
chrVI	114722	115052	331	1.29173
chrVI	115262	115435	174	1.27967
chrVI	118405	119083	679	1.38915
chrVI	130071	130227	157	1.40154
chrVI	130296	130420	125	1.17926
chrVI	130483	134911	4429	3.41396
chrVI	134964	135298	335	1.25743
chrVI	136552	136700	149	1.19181
chrVI	143975	144200	226	1.36405
chrVI	144364	145346	983	1.53628
chrVI	147787	148539	753	2.64774
chrVI	148645	149161	517	2.50223
chrVI	152325	152532	208	1.2839
chrVI	154168	155757	1590	1.91822
chrVI	155810	155990	181	1.47389
chrVI	156106	156224	119	1.27187
chrVI	156960	157867	908	1.65849
chrVI	158043	158461	419	1.44486
chrVI	161475	161753	279	1.29704
chrVI	166269	167275	1007	1.33403
chrVI	167588	167990	403	1.30149
chrVI	169977	170102	126	1.28703
chrVI	170702	170865	164	1.21467
chrVI	170958	174429	3472	3.23147
chrVI	174516	174651	136	1.21835
chrVI	190721	191488	768	1.49598
chrVI	191649	195130	3482	1.81276
chrVI	195956	197463	1508	2.03631
chrVI	197564	197794	231	1.21445
chrVI	197852	198104	253	1.2147
chrVI	198575	202347	3773	2.95211
chrVI	202839	204814	1976	2.12451
chrVI	205315	206098	784	1.40488
chrVI	207557	208103	547	1.41427
chrVI	213370	213490	121	1.25372
chrVI	215430	217993	2564	3.69237
chrVI	221281	221443	163	1.64529
chrVI	223206	223438	233	1.43022
chrVI	225327	225630	304	1.70355
chrVI	226204	226615	412	1.21083
chrVI	226948	227786	839	1.67141
chrVI	230183	230851	669	1.52381
chrVI	230960	232650	1691	2.13948
chrVI	233573	234056	484	3.59873

chrVI	237334	240235	2902	2.24037
chrVI	240899	241144	246	1.24552
chrVI	247628	249284	1657	1.53786
chrVI	253183	253776	594	1.41419
chrVI	254964	255082	119	1.39509
chrVI	256048	256266	219	1.38976
chrVI	263558	263687	130	1.36995
chrVI	263802	264347	546	1.28647
chrVI	265407	265568	162	1.22759
chrVI	265690	265798	109	1.25552
chrVI	269851	270037	187	1.34597
chrVII	814	997	184	2.12625
chrVII	1147	1373	227	1.40623
chrVII	21015	21200	186	1.42336
chrVII	22937	23330	394	1.19076
chrVII	23680	26147	2468	2.30873
chrVII	40651	40891	241	1.27766
chrVII	40985	41341	357	1.23003
chrVII	51795	53167	1373	2.8426
chrVII	53223	53485	263	1.33396
chrVII	53978	54086	109	1.22543
chrVII	55869	56020	152	1.43235
chrVII	56075	56214	140	1.39418
chrVII	56322	57015	694	1.4203
chrVII	61530	65067	3538	1.46252
chrVII	67159	67518	360	3.68516
chrVII	72471	72845	375	1.36499
chrVII	74963	76468	1506	1.59241
chrVII	76526	78859	2334	2.28952
chrVII	79576	79717	142	1.28678
chrVII	79825	81067	1243	1.72967
chrVII	81598	81998	401	1.33867
chrVII	84039	84722	684	6.55744
chrVII	89190	89302	113	1.3319
chrVII	89533	90608	1076	1.67944
chrVII	90684	91039	356	1.2512
chrVII	91171	91283	113	1.30832
chrVII	93821	94950	1130	1.87622
chrVII	101250	103174	1925	3.82696
chrVII	107211	107438	228	1.22197
chrVII	107686	107877	192	1.22059
chrVII	111787	112518	732	1.59607
chrVII	114160	114963	804	1.21033
chrVII	115214	115412	199	1.75594
chrVII	115610	117962	2353	2.3685
chrVII	123104	123334	231	1.22626
chrVII	123605	123923	319	1.19794
chrVII	138862	140203	1342	3.00925
chrVII	140327	140691	365	1.78391
chrVII	144011	144164	154	1.2636
chrVII	144771	145980	1210	2.08926
chrVII	160046	160212	167	1.20718
chrVII	162783	163928	1146	1.72877

chrVII	165754	166159	406	1.52449
chrVII	166276	166604	329	1.49935
chrVII	167279	167587	309	1.19176
chrVII	170223	172390	2168	4.62213
chrVII	174892	175413	522	1.41427
chrVII	183691	184553	863	1.46573
chrVII	186151	186271	121	1.41192
chrVII	186434	186571	138	1.22935
chrVII	186640	187335	696	1.92809
chrVII	187440	187861	422	1.74159
chrVII	190480	190630	151	1.2536
chrVII	191413	192065	653	1.41346
chrVII	195643	196939	1297	1.26943
chrVII	197025	197188	164	1.34281
chrVII	197263	198293	1031	1.55069
chrVII	198481	198852	372	1.36536
chrVII	199417	199594	178	1.21198
chrVII	199656	199781	126	1.29221
chrVII	213560	214226	667	1.39302
chrVII	219894	222123	2230	3.58188
chrVII	224391	224571	181	1.23749
chrVII	224763	226789	2027	1.92208
chrVII	229657	230423	767	7.54381
chrVII	231141	233242	2102	2.41872
chrVII	240268	241588	1321	2.53524
chrVII	245650	245887	238	1.35048
chrVII	247892	248712	821	1.34195
chrVII	248805	249256	452	1.30495
chrVII	249322	249589	268	1.27823
chrVII	249844	250428	585	1.20915
chrVII	252550	253229	680	1.33592
chrVII	253973	254182	210	1.31782
chrVII	254511	254618	108	1.20579
chrVII	255301	256049	749	1.48864
chrVII	257005	258039	1035	1.61114
chrVII	258681	259187	507	1.26616
chrVII	259269	262188	2920	2.01292
chrVII	262273	262395	123	1.22744
chrVII	272342	273631	1290	1.72854
chrVII	273688	274663	976	1.74574
chrVII	274744	275268	525	2.15355
chrVII	277498	277733	236	1.22979
chrVII	278071	278733	663	1.31051
chrVII	296578	296787	210	1.22945
chrVII	297340	299055	1716	2.11452
chrVII	300087	300205	119	1.30243
chrVII	300805	301720	916	1.45747
chrVII	308087	308196	110	1.2637
chrVII	312351	312459	109	1.20379
chrVII	325954	326215	262	1.50467
chrVII	328773	329227	455	1.45445
chrVII	329508	329730	223	1.22176
chrVII	330026	330318	293	1.18889

chrVII	330413	330520	108	1.2375
chrVII	330818	330989	172	1.18924
chrVII	334400	334650	251	1.29173
chrVII	341401	341965	565	1.40422
chrVII	345969	346287	319	1.34905
chrVII	348820	350882	2063	1.63022
chrVII	353326	353569	244	1.34341
chrVII	357211	357373	163	1.31261
chrVII	358606	359193	588	1.36882
chrVII	359246	359653	408	1.56685
chrVII	359734	360770	1037	1.37911
chrVII	361818	362169	352	1.22849
chrVII	371670	372444	775	1.37922
chrVII	373979	374538	560	1.34798
chrVII	379354	380397	1044	1.64946
chrVII	384823	384942	120	1.27334
chrVII	388277	388383	107	1.23344
chrVII	388626	388748	123	1.26561
chrVII	388897	389302	406	1.53681
chrVII	393925	395325	1401	1.57553
chrVII	395436	395861	426	1.37546
chrVII	395997	396246	250	1.34319
chrVII	396920	397678	759	1.42786
chrVII	398006	400278	2273	1.92581
chrVII	400377	401523	1147	1.88234
chrVII	406397	407390	994	1.68057
chrVII	413044	413189	146	1.39532
chrVII	424738	425205	468	1.37223
chrVII	426486	426640	155	1.25303
chrVII	427624	427978	355	1.46851
chrVII	428590	429625	1036	1.49927
chrVII	429955	430598	644	1.38213
chrVII	431154	431260	107	1.19743
chrVII	431502	432287	786	2.10407
chrVII	434144	434376	233	1.3923
chrVII	436735	437427	693	1.31986
chrVII	438891	439656	766	1.42558
chrVII	439733	440591	859	1.24097
chrVII	440823	441647	825	1.78043
chrVII	441751	441927	177	1.20735
chrVII	442960	444021	1062	2.35234
chrVII	448015	448630	616	1.57096
chrVII	452119	452793	675	1.71761
chrVII	456198	458445	2248	1.31781
chrVII	458547	460064	1518	1.2376
chrVII	460167	460482	316	1.22945
chrVII	461174	463155	1982	1.25591
chrVII	463998	465566	1569	1.65343
chrVII	465691	466157	467	1.47454
chrVII	468017	469868	1852	5.22718
chrVII	470249	470419	171	1.23559
chrVII	470514	470705	192	1.27156
chrVII	470762	471158	397	1.26208

chrVII	471369	473699	2331	4.85675
chrVII	473762	474790	1029	2.02074
chrVII	475784	477106	1323	1.81445
chrVII	479571	479677	107	1.20033
chrVII	482384	482903	520	1.37946
chrVII	482999	484398	1400	1.82839
chrVII	485609	486214	606	1.4763
chrVII	488869	490864	1996	3.03577
chrVII	491862	491969	108	1.30515
chrVII	492096	492255	160	1.31764
chrVII	496430	497677	1248	3.45141
chrVII	504136	504878	743	1.45364
chrVII	507825	508448	624	1.31618
chrVII	520410	521252	843	1.30433
chrVII	530925	531313	389	1.30431
chrVII	553586	554743	1158	1.71122
chrVII	556751	557061	311	1.47814
chrVII	557631	557921	291	1.70043
chrVII	576530	577765	1236	1.24624
chrVII	582039	582270	232	1.31588
chrVII	585598	586977	1380	2.39764
chrVII	592785	593039	255	1.56593
chrVII	594008	594221	214	1.19513
chrVII	599021	599965	945	1.64155
chrVII	603802	604098	297	1.32528
chrVII	604162	605308	1147	1.75318
chrVII	608944	609097	154	1.18264
chrVII	609182	613216	4035	4.45288
chrVII	615528	616231	704	1.51233
chrVII	616615	616758	144	1.20231
chrVII	619391	619811	421	1.29761
chrVII	623979	625345	1367	1.91574
chrVII	625638	625904	267	1.22374
chrVII	626012	626120	109	1.27834
chrVII	626762	626953	192	1.3838
chrVII	627034	627272	239	1.68748
chrVII	627466	627819	354	1.65319
chrVII	628021	628383	363	1.22744
chrVII	628974	629096	123	1.29976
chrVII	630445	632382	1938	2.88479
chrVII	636027	636133	107	1.19731
chrVII	636491	636742	252	1.21547
chrVII	637375	637610	236	1.30527
chrVII	639146	640425	1280	2.28532
chrVII	640510	641628	1119	1.32764
chrVII	642328	643621	1294	1.78593
chrVII	643985	644715	731	1.4203
chrVII	649235	649355	121	1.27967
chrVII	649489	649782	294	1.28972
chrVII	650305	650723	419	1.40176
chrVII	654141	654281	141	1.38361
chrVII	659485	660027	543	1.32588
chrVII	660113	660330	218	1.354

chrVII	661516	661692	177	1.34597
chrVII	662085	662329	245	1.40222
chrVII	666002	666232	231	1.24014
chrVII	670374	670557	184	1.18867
chrVII	672348	672898	551	1.44463
chrVII	675056	675456	401	1.31181
chrVII	675690	676206	517	1.69351
chrVII	676285	676430	146	1.39193
chrVII	676998	678786	1789	1.77394
chrVII	681702	683339	1638	3.0013
chrVII	694581	694819	239	1.32989
chrVII	696053	696301	249	1.25912
chrVII	696556	697800	1245	1.58183
chrVII	699263	699523	261	1.30013
chrVII	701255	701641	387	1.45967
chrVII	702076	702311	236	1.26357
chrVII	702474	702688	215	1.30977
chrVII	713085	713432	348	1.3319
chrVII	724144	725834	1691	2.68591
chrVII	726000	726111	112	1.19366
chrVII	727755	728484	730	1.6011
chrVII	730613	730832	220	1.19528
chrVII	732386	733699	1314	1.65557
chrVII	734020	734553	534	1.5036
chrVII	734719	735097	379	1.38213
chrVII	738611	738801	191	1.26159
chrVII	739415	740198	784	1.42518
chrVII	746926	747137	212	1.31764
chrVII	747217	747326	110	1.19705
chrVII	747447	749682	2236	1.98428
chrVII	763200	763780	581	1.78354
chrVII	765106	766603	1498	2.09931
chrVII	772056	772274	219	1.39896
chrVII	774175	774301	127	1.28572
chrVII	777656	778254	599	1.36715
chrVII	778469	778775	307	1.29092
chrVII	783305	783422	118	1.22158
chrVII	783540	784159	620	1.78993
chrVII	784249	784646	398	1.37588
chrVII	784717	786096	1380	1.7496
chrVII	789563	789777	215	1.20253
chrVII	789872	791636	1765	1.61293
chrVII	792430	792599	170	1.19639
chrVII	798446	799119	674	1.23952
chrVII	801645	803181	1537	2.24949
chrVII	804267	805759	1493	1.78793
chrVII	808093	808326	234	1.31515
chrVII	809666	809924	259	1.21202
chrVII	833195	833611	417	1.636
chrVII	833692	833819	128	1.34882
chrVII	836649	837171	523	5.7355
chrVII	840615	841238	624	1.30973
chrVII	844702	845259	558	1.30281

chrVII	846007	846612	606	1.39222
chrVII	846769	850173	3405	2.76586
chrVII	850330	850436	107	1.25125
chrVII	850498	850987	490	1.50504
chrVII	859054	859188	135	1.19558
chrVII	869782	870118	337	1.39418
chrVII	870407	870753	347	1.4383
chrVII	871358	871502	145	1.23793
chrVII	882592	882712	121	1.23389
chrVII	883847	884156	310	1.49663
chrVII	888138	888350	213	1.26996
chrVII	894395	894597	203	1.29912
chrVII	894705	894894	190	1.24361
chrVII	895064	895635	572	1.35875
chrVII	899490	900845	1356	1.6473
chrVII	902673	902787	115	1.23085
chrVII	902845	902969	125	1.27878
chrVII	903142	903340	199	1.43462
chrVII	904464	904609	146	1.24102
chrVII	904663	904807	145	1.28706
chrVII	905167	905518	352	1.20837
chrVII	908594	910833	2240	1.50458
chrVII	913074	913268	195	1.21875
chrVII	913788	914042	255	1.23261
chrVII	914591	915711	1121	2.6312
chrVII	920613	920860	248	1.39654
chrVII	920947	921081	135	1.23146
chrVII	921861	921993	133	1.23405
chrVII	922417	923226	810	1.28996
chrVII	923286	923507	222	1.30981
chrVII	935108	936890	1783	2.53323
chrVII	937238	937352	115	1.36503
chrVII	939382	939665	284	1.30307
chrVII	940262	940985	724	1.42737
chrVII	941078	941275	198	1.19459
chrVII	948196	949859	1664	2.87474
chrVII	949958	950335	378	1.27967
chrVII	950397	951469	1073	1.71818
chrVII	959044	959195	152	1.16736
chrVII	959443	959695	253	1.27117
chrVII	959927	960415	489	1.26914
chrVII	960540	961767	1228	1.74092
chrVII	962756	963277	522	1.31304
chrVII	967976	968105	130	1.19932
chrVII	968334	969101	768	1.2306
chrVII	969225	969455	231	1.22792
chrVII	970335	970566	232	1.26777
chrVII	973778	973980	203	1.30111
chrVII	977310	977494	185	1.32535
chrVII	977578	977824	247	1.40778
chrVII	986870	987079	210	1.26031
chrVII	990516	991558	1043	1.67264
chrVII	991780	991898	119	1.19457

chrVII	992591	994420	1830	1.44843
chrVII	997909	998302	394	1.37409
chrVII	1002267	1002422	156	1.19995
chrVII	1005676	1006826	1151	1.50869
chrVII	1006894	1007263	370	1.32387
chrVII	1007318	1008012	695	1.29266
chrVII	1010541	1015322	4782	2.90408
chrVII	1017439	1017687	249	1.28509
chrVII	1019165	1019685	521	1.45833
chrVII	1021178	1021285	108	1.27967
chrVII	1024685	1024791	107	1.23146
chrVII	1037093	1040284	3192	1.97908
chrVII	1048630	1048909	280	1.37409
chrVII	1049821	1049976	156	1.30378
chrVII	1050169	1051060	892	1.37064
chrVII	1056744	1059118	2375	3.69237
chrVII	1065299	1065697	399	1.48285
chrVII	1066581	1066754	174	1.18082
chrVII	1066860	1067335	476	1.2837
chrVIII	7772	7936	165	1.97475
chrVIII	18480	19162	683	1.41319
chrVIII	20442	22743	2302	2.03321
chrVIII	22843	23896	1054	1.64389
chrVIII	25735	26720	986	1.44503
chrVIII	27409	29335	1927	2.37653
chrVIII	31860	32019	160	1.25155
chrVIII	32354	32504	151	1.33204
chrVIII	32569	32767	199	1.38462
chrVIII	32924	33040	117	1.34195
chrVIII	41233	41371	139	1.25356
chrVIII	41468	41583	116	1.24351
chrVIII	44311	44572	262	1.22711
chrVIII	45218	46976	1759	2.82105
chrVIII	47265	47993	729	1.33346
chrVIII	56441	57345	905	1.72318
chrVIII	58415	60687	2273	2.82802
chrVIII	64456	64645	190	1.34597
chrVIII	65382	65846	465	1.23674
chrVIII	66956	67977	1022	1.56895
chrVIII	69681	70351	671	1.33561
chrVIII	70408	70640	233	1.34396
chrVIII	71122	72692	1571	1.97877
chrVIII	74943	75071	129	1.39711
chrVIII	75243	75964	722	1.54581
chrVIII	76113	76652	540	1.37028
chrVIII	78511	79356	846	1.5773
chrVIII	79948	81115	1168	1.57146
chrVIII	81184	81290	107	1.22148
chrVIII	92354	92510	157	1.36405
chrVIII	105116	106241	1126	4.02896
chrVIII	109144	109274	131	1.24771
chrVIII	109328	110969	1642	2.15061
chrVIII	119042	120346	1305	2.56027

chrVIII	120472	120943	472	1.23931
chrVIII	121003	122544	1542	2.47905
chrVIII	123203	123317	115	1.19328
chrVIII	134564	134740	177	1.19246
chrVIII	135598	137209	1612	2.38168
chrVIII	138008	138183	176	1.29606
chrVIII	138244	139114	871	1.45383
chrVIII	139174	140398	1225	1.47855
chrVIII	140701	144565	3865	2.22788
chrVIII	144669	145117	449	1.3319
chrVIII	147783	148187	405	1.46248
chrVIII	156514	158434	1921	2.9772
chrVIII	158499	158669	171	1.20299
chrVIII	158752	159322	571	1.20726
chrVIII	159412	159943	532	1.29976
chrVIII	160097	161819	1723	2.12908
chrVIII	183462	187546	4085	2.90479
chrVIII	189126	191310	2185	2.59457
chrVIII	192455	192801	347	1.28972
chrVIII	194940	195533	594	1.78695
chrVIII	195588	195871	284	1.29373
chrVIII	196648	199377	2730	1.74187
chrVIII	201146	201327	182	1.19657
chrVIII	201477	202135	659	1.26275
chrVIII	202195	202325	131	1.28312
chrVIII	204163	204485	323	1.50414
chrVIII	206554	206669	116	1.23385
chrVIII	209705	209824	120	1.18657
chrVIII	210033	211232	1200	1.46031
chrVIII	211295	212278	984	2.21381
chrVIII	221105	221250	146	1.22784
chrVIII	232056	232340	285	1.32291
chrVIII	233274	233459	186	1.21941
chrVIII	236679	237343	665	1.40159
chrVIII	238223	238329	107	1.21137
chrVIII	241777	242005	229	1.40412
chrVIII	247506	249358	1853	2.77751
chrVIII	251738	252787	1050	1.84417
chrVIII	255872	256414	543	1.8323
chrVIII	257364	258852	1489	2.34709
chrVIII	262241	262382	142	1.33502
chrVIII	267813	268238	426	1.25733
chrVIII	271521	271822	302	1.27565
chrVIII	279498	280387	890	1.4828
chrVIII	281776	282632	857	1.65428
chrVIII	282836	283135	300	1.26561
chrVIII	314152	314509	358	1.39606
chrVIII	322966	323878	913	1.50829
chrVIII	325951	327428	1478	1.88636
chrVIII	327494	329161	1668	1.68351
chrVIII	330046	330718	673	1.33971
chrVIII	334164	335074	911	1.46449
chrVIII	335672	335797	126	1.32186

chrVIII	335999	336763	765	1.18907
chrVIII	339728	339945	218	1.25557
chrVIII	340177	340414	238	1.22254
chrVIII	340472	340709	238	1.28369
chrVIII	341817	342153	337	1.52071
chrVIII	342317	342487	171	1.24351
chrVIII	343590	343872	283	1.26561
chrVIII	343965	344248	284	1.27251
chrVIII	352237	352449	213	1.37651
chrVIII	352867	353088	222	1.31222
chrVIII	353164	353298	135	1.1825
chrVIII	353467	353674	208	1.42027
chrVIII	354359	354618	260	1.26743
chrVIII	358632	358975	344	1.35786
chrVIII	360905	361144	240	1.25114
chrVIII	361295	363620	2326	2.14312
chrVIII	363848	365165	1318	1.33664
chrVIII	370015	370294	280	1.33744
chrVIII	377107	377233	127	1.26254
chrVIII	377395	378113	719	1.25294
chrVIII	392009	392812	804	2.09328
chrVIII	394004	394633	630	1.579
chrVIII	396873	397214	342	2.85382
chrVIII	398495	398942	448	1.35601
chrVIII	399146	399377	232	1.39619
chrVIII	400995	401325	331	2.00569
chrVIII	402000	402140	141	1.19412
chrVIII	409983	411824	1842	2.11927
chrVIII	413091	413581	491	1.40222
chrVIII	413655	414289	635	1.48257
chrVIII	419101	420224	1124	1.51438
chrVIII	420290	420486	197	1.30981
chrVIII	422134	424978	2845	3.23756
chrVIII	461084	464123	3040	2.4611
chrVIII	464221	464443	223	1.37924
chrVIII	464569	464872	304	1.29736
chrVIII	465242	465393	152	1.26109
chrVIII	470381	471337	957	1.69418
chrVIII	472293	473608	1316	1.59105
chrVIII	473887	474136	250	1.18355
chrVIII	474190	476254	2065	2.38055
chrVIII	484614	484889	276	1.26378
chrVIII	484948	485234	287	1.45043
chrVIII	485931	486112	182	1.22141
chrVIII	486261	486434	174	1.25796
chrVIII	486560	486682	123	1.33793
chrVIII	488168	488787	620	7.16848
chrVIII	492052	492294	243	1.3319
chrVIII	493201	493324	124	1.22183
chrVIII	493855	493984	130	1.3319
chrVIII	494049	494191	143	1.354
chrVIII	494260	496611	2352	2.07947
chrVIII	496670	496783	114	1.2857

chrVIII	511931	512830	900	9.88725
chrVIII	514247	514745	499	1.50668
chrVIII	514911	518670	3760	2.08725
chrVIII	518961	519286	326	1.34841
chrVIII	519495	519638	144	1.21534
chrVIII	519877	520628	752	1.93257
chrVIII	520855	521725	871	1.8515
chrVIII	555846	556082	237	1.64211
chrX	21268	21481	214	1.21137
chrX	22081	23067	987	1.17985
chrX	24028	24355	328	1.30378
chrX	24617	24742	126	1.19339
chrX	27690	27808	119	1.28068
chrX	27884	28553	670	1.53815
chrX	30808	31738	931	1.49254
chrX	35931	37592	1662	2.59613
chrX	39069	41703	2635	2.68791
chrX	55713	57351	1639	1.70958
chrX	58182	58856	675	1.59708
chrX	59795	59981	187	1.37811
chrX	60302	61359	1058	1.59219
chrX	66887	68043	1157	2.41269
chrX	68098	70158	2061	1.53975
chrX	70583	70693	111	1.38082
chrX	70758	71005	248	1.33063
chrX	75132	75313	182	1.39804
chrX	75882	77094	1213	1.97274
chrX	85785	86026	242	1.34373
chrX	86933	87757	825	1.59306
chrX	91216	91442	227	1.27766
chrX	91653	92153	501	1.79998
chrX	97860	98421	562	1.30613
chrX	98759	100226	1468	1.64347
chrX	101978	102091	114	1.25152
chrX	102161	102276	116	1.35699
chrX	106338	107604	1267	1.56298
chrX	121985	122126	142	1.29373
chrX	122191	122367	177	1.34798
chrX	125184	126803	1620	2.03031
chrX	134932	135766	835	1.25006
chrX	144648	145539	892	1.37259
chrX	145713	146034	322	1.24636
chrX	146484	148472	1989	1.93867
chrX	148817	148973	157	1.25155
chrX	150521	150897	377	2.18496
chrX	160655	160905	251	1.20434
chrX	161263	161380	118	1.21495
chrX	161447	161817	371	1.39225
chrX	161882	162446	565	1.39201
chrX	163236	166049	2814	2.5935
chrX	171672	173461	1790	1.99006
chrX	173544	173801	258	1.42637
chrX	186669	186888	220	1.29878

chrX	195436	195545	110	1.19345
chrX	209140	210502	1363	3.30666
chrX	221178	221304	127	1.19733
chrX	221948	222202	255	1.25324
chrX	222331	222540	210	1.22775
chrX	223285	223391	107	1.23921
chrX	226862	227084	223	1.26661
chrX	228161	229139	979	1.4074
chrX	233506	235052	1547	2.54469
chrX	235966	237976	2011	2.2032
chrX	241528	241672	145	1.33775
chrX	241791	242294	504	1.18022
chrX	250571	252404	1834	3.01336
chrX	252475	252603	129	1.27164
chrX	254141	254472	332	1.33192
chrX	257353	257493	141	1.25557
chrX	260769	261787	1019	1.64071
chrX	265795	266224	430	1.53802
chrX	266293	267189	897	1.85614
chrX	268984	270785	1802	2.33443
chrX	270879	271110	232	1.36234
chrX	272932	275231	2300	2.22185
chrX	277837	277951	115	1.24607
chrX	278324	278441	118	1.38398
chrX	280073	282228	2156	3.02504
chrX	282847	284275	1429	2.27036
chrX	284779	284972	194	1.20196
chrX	285218	285349	132	1.39418
chrX	285412	285657	246	1.35802
chrX	290832	291193	362	1.29476
chrX	298373	300116	1744	3.12987
chrX	300448	300585	138	1.23749
chrX	302471	303159	689	1.5171
chrX	303254	303524	271	1.33647
chrX	305152	305691	540	1.25785
chrX	307177	308545	1369	2.33836
chrX	314305	314719	415	1.28144
chrX	315019	315136	118	1.26963
chrX	316854	317022	169	1.31583
chrX	322131	327223	5093	2.43735
chrX	327682	328280	599	1.35154
chrX	329619	329848	230	1.23252
chrX	332777	332927	151	1.20949
chrX	337051	338235	1185	1.51274
chrX	346554	348379	1826	2.17316
chrX	348463	348696	234	1.25634
chrX	348803	349428	626	1.55398
chrX	358028	358238	211	1.41178
chrX	360116	360879	764	1.5696
chrX	360935	361525	591	1.20567
chrX	367765	369614	1850	4.56625
chrX	369861	370080	220	1.23347
chrX	374249	374360	112	1.20838

chrX	380585	380710	126	1.24299
chrX	380766	381809	1044	1.44595
chrX	383333	386816	3484	1.87086
chrX	386899	387711	813	1.30819
chrX	391144	396289	5146	2.44598
chrX	396360	396467	108	1.23889
chrX	396834	397235	402	1.30247
chrX	397989	398380	392	1.34869
chrX	402402	402757	356	1.86991
chrX	408296	408435	140	1.32767
chrX	408497	410503	2007	3.30251
chrX	416427	416647	221	1.2589
chrX	416910	417100	191	1.26379
chrX	417384	417656	273	1.32059
chrX	430795	431860	1066	2.01651
chrX	433046	433339	294	1.32346
chrX	435798	436681	884	3.73228
chrX	440274	440388	115	1.23749
chrX	440480	441289	810	1.59429
chrX	453099	453674	576	1.64851
chrX	457675	457825	151	1.28948
chrX	458124	458398	275	1.51374
chrX	462650	462949	300	1.32985
chrX	463650	467102	3453	2.51613
chrX	467255	467428	174	1.28972
chrX	467894	468325	432	1.31181
chrX	471086	472380	1295	1.95312
chrX	491948	492147	200	1.20534
chrX	492393	492519	127	1.21941
chrX	499773	501222	1450	2.05765
chrX	509730	510661	932	1.82774
chrX	511579	511804	226	1.2857
chrX	515932	516687	756	1.83409
chrX	518336	518583	248	1.40601
chrX	518847	519087	241	1.37581
chrX	519397	519507	111	1.22526
chrX	526295	526475	181	1.26491
chrX	537464	537751	288	1.52086
chrX	539093	539492	400	1.27713
chrX	545862	545996	135	1.38369
chrX	546311	546423	113	1.1987
chrX	549585	549703	119	1.21539
chrX	549883	552564	2682	1.81788
chrX	552676	553882	1207	1.43436
chrX	554781	555200	420	2.11388
chrX	557356	558042	687	1.57096
chrX	558736	559239	504	1.47855
chrX	559385	559755	371	1.2561
chrX	565910	566021	112	1.2636
chrX	566195	567903	1709	1.47176
chrX	567959	575860	7902	2.30416
chrX	576456	576644	189	1.18986
chrX	577608	577859	252	1.20931

chrX	577962	578216	255	1.29707
chrX	578273	579551	1279	1.62998
chrX	579750	579946	197	1.22298
chrX	580290	580575	286	1.31244
chrX	580948	581097	150	1.19728
chrX	589657	591249	1593	3.24182
chrX	598618	598897	280	1.2303
chrX	602310	603401	1092	1.70263
chrX	604422	605928	1507	1.72313
chrX	606296	606904	609	2.10271
chrX	607242	607398	157	1.26008
chrX	609024	609229	206	1.61832
chrX	622356	623418	1063	1.70924
chrX	623528	623850	323	1.44112
chrX	625001	625161	161	1.2328
chrX	628419	630428	2010	1.35451
chrX	632342	633920	1579	2.97409
chrX	635184	635339	156	1.19031
chrX	637157	637265	109	1.31523
chrX	637772	638003	232	1.23528
chrX	639358	639852	495	6.60229
chrX	640149	640426	278	1.29754
chrX	640815	641259	445	1.46138
chrX	641868	642059	192	1.19593
chrX	642150	642463	314	1.33018
chrX	643269	644686	1418	1.41095
chrX	646619	646961	343	2.1732
chrX	647666	647814	149	1.32027
chrX	651844	654912	3069	2.20462
chrX	672646	674461	1816	1.38126
chrX	688632	690633	2002	2.41671
chrX	691002	691247	246	1.42271
chrX	697679	698686	1008	1.31784
chrX	700570	700694	125	1.29173
chrX	701235	701375	141	1.19731
chrX	702854	703080	227	1.24351
chrX	705091	706686	1596	1.29083
chrX	707672	707807	136	1.34936
chrX	711256	712624	1369	2.75421
chrX	717925	718062	138	1.20846
chrX	718204	718424	221	1.40757
chrX	721143	721338	196	1.27467
chrX	721409	722142	734	1.65382
chrX	722202	722518	317	1.33576
chrX	724402	724518	117	1.26762
chrXI	2913	3306	394	1.24954
chrXI	7658	7871	214	1.48938
chrXI	8422	9343	922	1.54917
chrXI	16372	16494	123	1.21005
chrXI	19497	20077	581	1.18563
chrXI	21540	21793	254	1.30862
chrXI	21930	22658	729	1.56368
chrXI	24046	26580	2535	1.89072

chrXI	26647	27419	773	1.54284
chrXI	30848	31454	607	1.24351
chrXI	33902	35068	1167	2.02698
chrXI	38607	38902	296	1.28331
chrXI	39206	39554	349	1.27159
chrXI	41692	43609	1918	3.37295
chrXI	46501	46644	144	1.31206
chrXI	49050	50756	1707	1.70583
chrXI	50903	51638	736	1.33391
chrXI	53219	53579	361	1.36405
chrXI	54006	54137	132	1.30177
chrXI	54817	56722	1906	5.34381
chrXI	66944	67529	586	1.65132
chrXI	67958	68081	124	1.30868
chrXI	68200	68574	375	1.91565
chrXI	68633	68742	110	1.27766
chrXI	76713	78186	1474	2.08247
chrXI	78425	78537	113	1.23583
chrXI	79121	80576	1456	1.86779
chrXI	80985	81316	332	1.31158
chrXI	81610	81725	116	1.21425
chrXI	85249	86826	1578	3.17809
chrXI	91832	92155	324	1.40016
chrXI	92466	92649	184	1.40965
chrXI	100149	100894	746	1.40623
chrXI	106681	107072	392	1.54469
chrXI	107138	107366	229	1.22018
chrXI	109243	109464	222	1.29134
chrXI	109589	109957	369	1.88755
chrXI	110084	110931	848	2.01104
chrXI	117969	119266	1298	1.25766
chrXI	120438	120681	244	1.28771
chrXI	126978	127449	472	2.69602
chrXI	129801	131764	1964	2.51507
chrXI	158609	160227	1619	1.79639
chrXI	164313	164426	114	1.25442
chrXI	164974	165532	559	1.23699
chrXI	166571	168012	1442	1.50869
chrXI	168487	168664	178	1.3315
chrXI	168763	168958	196	1.4668
chrXI	171012	171140	129	1.213
chrXI	184247	186981	2735	1.96366
chrXI	191045	191167	123	1.31623
chrXI	194585	195190	606	1.22505
chrXI	195305	196541	1237	1.61181
chrXI	202358	202571	214	1.35896
chrXI	205384	205981	598	1.41294
chrXI	212098	212708	611	1.64328
chrXI	216802	217285	484	2.25056
chrXI	217550	217664	115	1.39821
chrXI	218172	219092	921	1.48941
chrXI	219192	219396	205	1.20986
chrXI	220956	221975	1020	1.95851

chrXI	228920	229124	205	1.20974
chrXI	231110	231584	475	1.66607
chrXI	231881	232293	413	1.32387
chrXI	233753	233893	141	1.31305
chrXI	235747	236295	549	1.43436
chrXI	236384	236579	196	1.30579
chrXI	236847	239663	2817	2.75421
chrXI	245288	245593	306	1.43347
chrXI	246111	246302	192	1.19401
chrXI	247334	249223	1890	6.99824
chrXI	252581	254342	1762	3.03489
chrXI	254650	254864	215	1.20972
chrXI	261055	261238	184	1.29072
chrXI	263930	266820	2891	3.52786
chrXI	277156	277396	241	1.29173
chrXI	279156	279404	249	1.25818
chrXI	279548	279845	298	1.20936
chrXI	280484	280941	458	1.61516
chrXI	281073	281432	360	1.27967
chrXI	286309	286595	287	1.28415
chrXI	291067	291819	753	1.47547
chrXI	292330	293746	1417	1.74358
chrXI	299233	299913	681	1.30117
chrXI	302693	302861	169	1.26138
chrXI	308289	309692	1404	1.64271
chrXI	310291	310497	207	1.21581
chrXI	313028	313180	153	1.38209
chrXI	315976	316168	193	1.25111
chrXI	324937	327897	2961	2.2783
chrXI	330328	331650	1323	1.60511
chrXI	333145	333532	388	1.40021
chrXI	333607	334134	528	1.42426
chrXI	334379	334518	140	1.42528
chrXI	334784	334906	123	1.37885
chrXI	335066	335687	622	1.60913
chrXI	336173	336313	141	1.23066
chrXI	340854	341401	548	1.71986
chrXI	342465	342810	346	1.36999
chrXI	350744	351111	368	1.29633
chrXI	369201	371646	2446	2.15947
chrXI	371876	372320	445	1.46092
chrXI	373970	374807	838	1.56084
chrXI	374870	375144	275	1.36806
chrXI	377430	377840	411	1.37811
chrXI	377911	379633	1723	2.7964
chrXI	379778	380098	321	1.45043
chrXI	387498	387694	197	1.32559
chrXI	388711	390241	1531	1.83806
chrXI	393230	394137	908	7.99208
chrXI	394213	394830	618	1.36539
chrXI	394888	395002	115	1.20333
chrXI	398188	399317	1130	1.57498
chrXI	401194	403253	2060	1.89457

chrXI	409630	409797	168	1.21935
chrXI	411221	414044	2824	2.16654
chrXI	419390	421602	2213	1.84201
chrXI	428801	428970	170	1.30577
chrXI	429412	429653	242	1.36115
chrXI	429763	429937	175	1.29063
chrXI	432762	433309	548	1.64579
chrXI	439565	440879	1315	3.46438
chrXI	444951	445214	264	1.21941
chrXI	445485	446165	681	1.35819
chrXI	446279	446475	197	1.26166
chrXI	446583	447402	820	1.79236
chrXI	447464	448525	1062	1.67321
chrXI	453348	454994	1647	3.66364
chrXI	456296	456712	417	1.24251
chrXI	469854	470065	212	1.2401
chrXI	470167	470400	234	1.31221
chrXI	470809	472820	2012	2.90102
chrXI	472897	473484	588	1.34731
chrXI	478047	478192	146	1.29373
chrXI	478670	478880	211	1.28913
chrXI	481753	482384	632	1.48659
chrXI	484676	486177	1502	2.0453
chrXI	487199	487805	607	3.68875
chrXI	496587	498283	1697	3.75866
chrXI	498344	498724	381	1.20858
chrXI	500220	501880	1661	1.66739
chrXI	515060	515394	335	1.28853
chrXI	516729	516835	107	1.22694
chrXI	517086	517433	348	1.26038
chrXI	518834	519452	619	2.53232
chrXI	520041	525921	5881	1.99382
chrXI	526109	527477	1369	1.4818
chrXI	527557	528050	494	1.38962
chrXI	530370	530934	565	1.8061
chrXI	531011	535509	4499	2.73814
chrXI	557341	557457	117	1.19915
chrXI	557520	557648	129	1.21695
chrXI	559767	560131	365	1.25265
chrXI	560206	560492	287	1.25155
chrXI	561066	561263	198	1.24753
chrXI	564163	564380	218	1.30177
chrXI	564462	565739	1278	1.7819
chrXI	567056	567632	577	1.43056
chrXI	570001	570812	812	1.6039
chrXI	571405	571542	138	1.17989
chrXI	571633	571889	257	1.30253
chrXI	573038	573387	350	1.25356
chrXI	573482	576448	2967	2.76425
chrXI	576600	576822	223	1.27365
chrXI	579495	579631	137	1.35082
chrXI	585939	586343	405	1.29336
chrXI	586418	588103	1686	2.01826

chrXI	588462	588572	111	1.22342
chrXI	588863	591413	2551	4.68757
chrXI	591565	591781	217	1.23548
chrXI	591838	592195	358	1.21338
chrXI	595415	595652	238	1.27902
chrXI	595710	597468	1759	2.33898
chrXI	602241	604313	2073	2.25801
chrXI	604846	604954	109	1.20586
chrXI	605045	605492	448	1.21696
chrXI	608407	608863	457	2.88846
chrXI	612091	612339	249	1.34597
chrXI	617726	617932	207	1.26963
chrXI	626014	626120	107	1.21303
chrXI	629901	630056	156	1.34462
chrXI	630291	630913	623	3.04945
chrXI	630967	631750	784	1.67484
chrXI	631829	632039	211	1.23314
chrXI	632228	634342	2115	2.94607
chrXI	634831	635276	446	1.43715
chrXI	635547	636187	641	1.55159
chrXI	637718	638961	1244	2.3002
chrXII	22327	23285	959	2.26779
chrXII	29062	31779	2718	4.83182
chrXII	31879	33022	1144	2.63197
chrXII	36119	37348	1230	1.75046
chrXII	39028	39364	337	1.30902
chrXII	39758	40357	600	1.35207
chrXII	40908	41203	296	1.2807
chrXII	41357	41463	107	1.20936
chrXII	51555	53515	1961	3.36467
chrXII	53607	53992	386	1.5428
chrXII	63998	64129	132	1.44641
chrXII	64902	65040	139	1.33906
chrXII	65134	65271	138	1.29185
chrXII	65439	65735	297	1.58117
chrXII	69769	71328	1560	1.86426
chrXII	72527	72950	424	1.35065
chrXII	73528	74138	611	1.28098
chrXII	76767	76971	205	1.42833
chrXII	84015	84476	462	2.21954
chrXII	86148	88523	2376	2.40792
chrXII	91594	91990	397	1.52364
chrXII	95082	95739	658	2.01895
chrXII	97344	98392	1049	1.51838
chrXII	104203	106177	1975	4.2408
chrXII	107874	107985	112	1.24954
chrXII	108425	108989	565	1.37036
chrXII	113627	116140	2514	1.89841
chrXII	116196	116888	693	1.54887
chrXII	120152	121947	1796	2.26067
chrXII	122027	122504	478	1.6539
chrXII	124561	124669	109	1.25608
chrXII	124725	125368	644	2.20341

chrXII	125489	126373	885	1.64445
chrXII	127174	127285	112	1.36748
chrXII	127494	127929	436	1.40878
chrXII	127997	129621	1625	1.88636
chrXII	129691	129926	236	1.29055
chrXII	130388	130696	309	1.35762
chrXII	130931	131481	551	1.56847
chrXII	133155	134414	1260	1.48781
chrXII	138003	138189	187	1.22713
chrXII	138877	139193	317	1.25315
chrXII	139258	139946	689	1.60997
chrXII	140196	140747	552	1.39517
chrXII	141564	141679	116	1.26801
chrXII	149855	151942	2088	4.41979
chrXII	159450	159670	221	1.24531
chrXII	160164	160273	110	1.23112
chrXII	160440	160865	426	1.18935
chrXII	160924	162130	1207	2.12028
chrXII	164012	164395	384	3.50609
chrXII	169938	170250	313	1.53092
chrXII	172283	172509	227	1.22458
chrXII	177357	177475	119	1.32448
chrXII	179437	180058	622	2.6839
chrXII	180959	183262	2304	2.47095
chrXII	183328	184125	798	1.27876
chrXII	184219	184571	353	1.28063
chrXII	184657	185452	796	1.72087
chrXII	195485	195591	107	1.25356
chrXII	209446	212255	2810	2.4589
chrXII	224844	225103	260	1.33999
chrXII	231367	232065	699	1.24638
chrXII	233971	234641	671	1.30318
chrXII	243362	243542	181	1.20959
chrXII	243910	244042	133	1.20512
chrXII	247977	248190	214	1.21832
chrXII	252975	254263	1289	3.57591
chrXII	256791	257006	216	1.35802
chrXII	257069	257285	217	1.35601
chrXII	257353	260526	3174	2.28567
chrXII	263170	263765	596	1.72431
chrXII	264037	264298	262	1.25129
chrXII	265365	268775	3411	2.42809
chrXII	270933	271055	123	1.28644
chrXII	271314	271489	176	1.27967
chrXII	280724	282629	1906	1.81404
chrXII	282978	283637	660	1.38422
chrXII	283793	283899	107	1.20534
chrXII	285159	286279	1121	1.53681
chrXII	287570	288386	817	1.4615
chrXII	288555	290584	2030	1.89741
chrXII	291630	291739	110	1.24566
chrXII	292062	292336	275	1.24753
chrXII	300110	300376	267	1.40867

chrXII	305614	307932	2319	3.31067
chrXII	317700	318455	756	1.35802
chrXII	322235	324254	2020	2.23636
chrXII	325687	327492	1806	1.81002
chrXII	329380	329689	310	2.46844
chrXII	335566	337389	1824	3.90568
chrXII	340636	342316	1681	2.18311
chrXII	381874	383489	1616	2.26578
chrXII	386422	386528	107	1.31985
chrXII	387833	388599	767	1.58645
chrXII	394453	395298	846	1.72061
chrXII	395356	395505	150	1.43235
chrXII	395614	395904	291	1.51519
chrXII	402125	402746	622	1.37214
chrXII	415351	415650	300	1.18994
chrXII	415718	416334	617	1.31894
chrXII	420880	422095	1216	2.01135
chrXII	426904	427076	173	1.4088
chrXII	428900	429931	1032	1.53279
chrXII	436149	438024	1876	1.72015
chrXII	447242	447501	260	1.20641
chrXII	449526	449789	264	1.33047
chrXII	459848	460080	233	1.14852
chrXII	506917	507163	247	1.36606
chrXII	507249	507439	191	1.49864
chrXII	507521	508563	1043	1.7518
chrXII	512734	512929	196	1.2417
chrXII	514588	514793	206	1.19758
chrXII	515285	515489	205	1.28541
chrXII	520003	521058	1056	1.70403
chrXII	529862	531458	1597	3.5196
chrXII	546722	547703	982	1.5623
chrXII	560495	561108	614	1.32186
chrXII	561191	561297	107	1.20132
chrXII	562778	562983	206	1.22744
chrXII	563225	564707	1483	1.71761
chrXII	568096	568223	128	1.24122
chrXII	568358	568504	147	1.2691
chrXII	568579	568690	112	1.25798
chrXII	570501	570881	381	1.41051
chrXII	570955	571066	112	1.23243
chrXII	573985	574095	111	1.22924
chrXII	577814	578721	908	1.7258
chrXII	578781	580309	1529	1.23179
chrXII	581346	581634	289	1.47804
chrXII	581690	581809	120	1.23759
chrXII	588892	589547	656	6.60443
chrXII	589648	590758	1111	1.29732
chrXII	590843	591263	421	1.20853
chrXII	606572	607594	1023	1.61951
chrXII	611009	611723	715	1.35209
chrXII	611808	612519	712	1.77976
chrXII	612574	612813	240	1.36528

chrXII	616394	616513	120	1.25958
chrXII	620343	620481	139	1.28771
chrXII	620612	620793	182	1.21884
chrXII	622353	623802	1450	1.89239
chrXII	623887	625794	1908	2.07299
chrXII	625932	626378	447	1.41226
chrXII	626463	626812	350	1.40259
chrXII	627606	628303	698	1.48415
chrXII	628508	628958	451	1.49241
chrXII	636711	636933	223	1.24996
chrXII	637028	637139	112	1.2142
chrXII	657648	657788	141	1.21291
chrXII	657964	658128	165	1.21546
chrXII	660031	660219	189	1.23305
chrXII	662003	663591	1589	3.33615
chrXII	674393	674527	135	1.22677
chrXII	674803	676153	1351	1.4497
chrXII	676824	679399	2576	1.75965
chrXII	679851	680004	154	1.21843
chrXII	680131	680241	111	1.28503
chrXII	680310	680547	238	1.29622
chrXII	681091	681499	409	1.36673
chrXII	681984	682108	125	1.23438
chrXII	682201	684267	2067	1.59164
chrXII	693793	696423	2631	1.64749
chrXII	696533	696963	431	1.32645
chrXII	699552	699661	110	1.30568
chrXII	703863	704112	250	1.35275
chrXII	704232	704608	377	1.42318
chrXII	705662	706166	505	1.43034
chrXII	706241	706456	216	1.27967
chrXII	707851	708075	225	1.20653
chrXII	708143	708554	412	1.37409
chrXII	716847	717809	963	1.46395
chrXII	717894	718163	270	1.53162
chrXII	723391	723553	163	1.43562
chrXII	728608	729241	634	1.32561
chrXII	729333	729514	182	1.3871
chrXII	731462	731607	146	1.38012
chrXII	733263	734806	1544	2.88764
chrXII	734898	735468	571	1.31083
chrXII	746422	747763	1342	1.58834
chrXII	747921	749973	2053	1.92077
chrXII	759030	759831	802	2.62801
chrXII	759910	760548	639	1.71159
chrXII	762591	762842	252	1.279
chrXII	764816	764965	150	1.19067
chrXII	768626	769161	536	1.34539
chrXII	769296	769606	311	1.27967
chrXII	775378	777040	1663	2.15957
chrXII	780980	781302	323	1.44735
chrXII	782870	783455	586	1.90469
chrXII	789992	790110	119	1.20221

chrXII	790227	790776	550	1.48198
chrXII	793742	793929	188	1.77288
chrXII	794006	795214	1209	1.96439
chrXII	799019	800961	1943	2.55731
chrXII	802356	802482	127	1.23246
chrXII	809312	810098	787	1.33143
chrXII	815527	816890	1364	1.67502
chrXII	817858	818015	158	1.28369
chrXII	821411	823755	2345	1.96984
chrXII	827994	828501	508	1.65032
chrXII	828732	829131	400	1.45445
chrXII	833620	833752	133	1.21309
chrXII	833855	833972	118	1.19161
chrXII	834025	834138	114	1.29393
chrXII	835656	838349	2694	2.15628
chrXII	838842	840016	1175	1.57797
chrXII	844178	844568	391	1.43165
chrXII	844647	844781	135	1.3078
chrXII	845635	848047	2413	1.85623
chrXII	851459	852580	1122	1.71678
chrXII	852655	852773	119	1.2934
chrXII	852840	853066	227	1.24641
chrXII	855045	855702	658	1.29556
chrXII	855824	855948	125	1.38196
chrXII	856019	856238	220	1.27661
chrXII	861260	862311	1052	1.59507
chrXII	866131	869203	3073	3.16565
chrXII	870040	870195	156	1.27164
chrXII	875177	875342	166	1.41579
chrXII	881290	882732	1443	2.14752
chrXII	884679	885018	340	1.21202
chrXII	887846	889333	1488	3.07162
chrXII	892313	894206	1894	1.66005
chrXII	895201	896633	1433	2.12944
chrXII	899180	899424	245	1.38915
chrXII	904333	905118	786	1.46851
chrXII	909145	911193	2049	2.11909
chrXII	927843	928811	969	1.50869
chrXII	929500	929653	154	1.32607
chrXII	929780	929886	107	1.27967
chrXII	929945	930319	375	1.21941
chrXII	930617	931148	532	1.37409
chrXII	931255	931979	725	1.26028
chrXII	933335	933452	118	1.20308
chrXII	934641	934870	230	1.24554
chrXII	940888	941160	273	1.46441
chrXII	947210	947756	547	1.66538
chrXII	949701	949811	111	1.18534
chrXII	950825	951390	566	1.51345
chrXII	952487	954528	2042	1.68281
chrXII	962648	962907	260	1.34652
chrXII	963125	963325	201	1.26213
chrXII	964572	964749	178	1.20229

chrXII	970975	971172	198	1.20936
chrXII	971239	972524	1286	2.00489
chrXII	975138	975897	760	1.74907
chrXII	976079	976224	146	1.19025
chrXII	999288	1002840	3553	2.93866
chrXII	1002957	1003208	252	1.54686
chrXII	1004130	1004691	562	1.61315
chrXII	1005898	1006267	370	1.47454
chrXII	1006375	1006554	180	1.26159
chrXII	1006655	1007860	1206	1.67342
chrXII	1010702	1011298	597	1.44853
chrXII	1011441	1011730	290	1.41822
chrXII	1012166	1012343	178	1.1949
chrXII	1012566	1013274	709	1.20393
chrXII	1014622	1016443	1822	1.79194
chrXII	1017520	1017945	426	1.49864
chrXII	1018042	1018289	248	1.4203
chrXII	1024360	1026010	1651	1.62549
chrXII	1026131	1027315	1185	1.85824
chrXII	1027516	1027714	199	1.23217
chrXII	1038223	1039953	1731	4.00918
chrXII	1042919	1043058	140	1.22002
chrXII	1043167	1043791	625	1.34525
chrXII	1044018	1044429	412	1.45504
chrXII	1044483	1044590	108	1.21338
chrXII	1051723	1051998	276	1.38107
chrXII	1052171	1052395	225	1.30814
chrXII	1052603	1052709	107	1.35281
chrXII	1052788	1053017	230	1.45517
chrXII	1053078	1053281	204	1.498
chrXII	1054420	1056630	2211	1.94969
chrXII	1056688	1056813	126	1.44232
chrXII	1057316	1057864	549	1.37368
chrXII	1058829	1059282	454	1.46851
chrXII	1064946	1065090	145	1.81179
chrXIII	18457	20526	2070	1.70757
chrXIII	20582	20715	134	1.21137
chrXIII	25461	25820	360	1.24225
chrXIII	26783	26946	164	1.25048
chrXIII	27031	28256	1226	1.59763
chrXIII	37704	38618	915	1.59327
chrXIII	38976	40973	1998	1.89641
chrXIII	41276	41545	270	1.24223
chrXIII	43716	43867	152	1.35009
chrXIII	46112	46550	439	1.41811
chrXIII	46616	46975	360	1.2415
chrXIII	49409	49673	265	1.2093
chrXIII	49727	50573	847	1.75346
chrXIII	50693	51015	323	1.41473
chrXIII	51195	51415	221	1.27345
chrXIII	51544	51851	308	1.40824
chrXIII	54312	55536	1225	1.79234
chrXIII	56377	57061	685	1.53681

chrXIII	57142	59315	2174	2.64171
chrXIII	71856	72088	233	1.36964
chrXIII	73491	75117	1627	3.21195
chrXIII	75297	75541	245	1.33146
chrXIII	76318	77671	1354	1.78292
chrXIII	80050	80224	175	1.36595
chrXIII	82433	83790	1358	1.29876
chrXIII	84794	86037	1244	2.04707
chrXIII	87871	88094	224	1.32636
chrXIII	88390	88562	173	1.2204
chrXIII	88641	88782	142	1.3078
chrXIII	88965	89388	424	1.25757
chrXIII	91042	91434	393	1.5587
chrXIII	94135	94252	118	1.40021
chrXIII	94407	95141	735	1.41438
chrXIII	95208	96215	1008	1.70355
chrXIII	97439	97552	114	1.36528
chrXIII	108441	109129	689	1.26457
chrXIII	110198	110666	469	1.49412
chrXIII	111004	113249	2246	2.05759
chrXIII	117623	117841	219	1.2214
chrXIII	117988	120425	2438	1.65398
chrXIII	137261	137435	175	1.21171
chrXIII	144616	145637	1022	1.64328
chrXIII	145708	146139	432	1.2068
chrXIII	147006	147712	707	1.99484
chrXIII	148431	149128	698	1.29599
chrXIII	152713	153372	660	1.63324
chrXIII	158251	158978	728	4.18147
chrXIII	159400	159556	157	1.31419
chrXIII	161600	162315	716	2.43319
chrXIII	162376	162650	275	1.56092
chrXIII	163599	165340	1742	2.1989
chrXIII	166764	167277	514	1.37601
chrXIII	167330	168053	724	1.35026
chrXIII	168121	168311	191	1.39197
chrXIII	168905	169412	508	1.57332
chrXIII	170299	170844	546	1.70489
chrXIII	172731	172840	110	1.27851
chrXIII	172915	173227	313	1.24731
chrXIII	173292	174684	1393	1.79701
chrXIII	182826	182997	172	1.36806
chrXIII	183145	183394	250	1.39619
chrXIII	183667	183876	210	1.36606
chrXIII	190183	190403	221	1.3078
chrXIII	191682	191948	267	1.38414
chrXIII	193012	193465	454	1.26249
chrXIII	193770	195636	1867	1.78862
chrXIII	202242	202892	651	1.50703
chrXIII	203668	203809	142	1.22788
chrXIII	208956	209532	577	6.42146
chrXIII	211149	211464	316	1.30763
chrXIII	211541	212585	1045	2.03502

chrXIII	220114	220314	201	1.39019
chrXIII	220368	220498	131	1.50327
chrXIII	220682	221394	713	1.50071
chrXIII	221498	221613	116	1.24085
chrXIII	222518	222625	108	1.36165
chrXIII	222695	222935	241	1.35802
chrXIII	223378	223546	169	1.31324
chrXIII	226789	227281	493	1.4223
chrXIII	228870	229426	557	1.20656
chrXIII	233208	233475	268	1.28674
chrXIII	233762	233868	107	1.23146
chrXIII	233931	234475	545	1.2857
chrXIII	234574	234997	424	1.31181
chrXIII	235560	237510	1951	4.31751
chrXIII	238469	239888	1420	2.33234
chrXIII	250872	251620	749	1.79636
chrXIII	252331	253914	1584	7.66496
chrXIII	255535	257053	1519	2.80597
chrXIII	258510	258638	129	1.38378
chrXIII	258695	258911	217	1.39834
chrXIII	267371	268626	1256	5.57447
chrXIII	276615	277970	1356	2.69699
chrXIII	278053	278259	207	1.23286
chrXIII	294449	295839	1391	2.4738
chrXIII	296564	296757	194	1.23539
chrXIII	297108	297628	521	1.43095
chrXIII	298836	299299	464	1.32827
chrXIII	299911	302831	2921	2.62341
chrXIII	316943	317337	395	1.40824
chrXIII	317640	317777	138	1.31246
chrXIII	318890	319740	851	1.61772
chrXIII	325158	325284	127	1.3305
chrXIII	327622	328940	1319	1.86426
chrXIII	329007	329278	272	1.25557
chrXIII	329333	329722	390	1.26453
chrXIII	331286	331403	118	1.20979
chrXIII	331584	332536	953	1.66957
chrXIII	334722	335248	527	1.59684
chrXIII	338575	342018	3444	1.42877
chrXIII	346518	346798	281	1.25449
chrXIII	346875	346987	113	1.3066
chrXIII	353536	353735	200	1.31895
chrXIII	356521	356980	460	1.17699
chrXIII	362943	363855	913	2.96277
chrXIII	379764	380210	447	1.2436
chrXIII	386060	386714	655	1.44038
chrXIII	386794	387010	217	1.3083
chrXIII	387156	390935	3780	3.32361
chrXIII	391014	391504	491	1.4223
chrXIII	394011	396514	2504	1.72115
chrXIII	396588	397756	1169	1.53617
chrXIII	398100	398209	110	1.26174
chrXIII	398653	398841	189	1.25356

chrXIII	399442	399710	269	1.18544
chrXIII	399850	400070	221	1.27794
chrXIII	403371	404779	1409	2.33403
chrXIII	408837	408961	125	1.36831
chrXIII	410745	411362	618	1.37042
chrXIII	411581	411811	231	1.22081
chrXIII	411891	412577	687	1.34239
chrXIII	414825	416801	1977	2.23635
chrXIII	420779	420932	154	1.29976
chrXIII	425231	425364	134	1.23383
chrXIII	425738	427061	1324	1.80801
chrXIII	430964	431452	489	1.69837
chrXIII	432062	432208	147	1.40976
chrXIII	434739	435099	361	1.19957
chrXIII	441973	442136	164	1.28972
chrXIII	442308	443869	1562	2.1584
chrXIII	444634	445482	849	1.46329
chrXIII	449807	450081	275	1.2415
chrXIII	453158	453395	238	1.23157
chrXIII	459195	460105	911	1.579
chrXIII	460299	460408	110	1.3514
chrXIII	467179	469252	2074	3.33398
chrXIII	469334	469468	135	1.24994
chrXIII	472916	473187	272	1.77927
chrXIII	483835	483986	152	1.22744
chrXIII	489773	491363	1591	2.66983
chrXIII	491437	492244	808	1.47977
chrXIII	498845	500038	1194	2.01091
chrXIII	500438	500729	292	1.29749
chrXIII	501258	501768	511	1.33402
chrXIII	506711	507206	496	1.42004
chrXIII	507424	509524	2101	1.85109
chrXIII	509637	509881	245	1.21698
chrXIII	511015	511136	122	1.20204
chrXIII	511337	511583	247	1.30581
chrXIII	513560	513815	256	1.28936
chrXIII	514173	514282	110	1.37484
chrXIII	519828	521438	1611	2.69468
chrXIII	521619	521842	224	1.22486
chrXIII	522685	522829	145	1.2164
chrXIII	523809	523917	109	1.30159
chrXIII	523981	524131	151	1.38132
chrXIII	531540	532086	547	1.45242
chrXIII	532141	532608	468	1.48406
chrXIII	532902	533938	1037	1.43958
chrXIII	535474	535643	170	1.20785
chrXIII	538264	538520	257	1.24861
chrXIII	538712	539215	504	1.30475
chrXIII	540241	541009	769	1.82985
chrXIII	542504	542819	316	1.3133
chrXIII	542950	543521	572	1.35101
chrXIII	549652	550210	559	2.20979
chrXIII	553712	555048	1337	1.78751

chrXIII	556062	557236	1175	1.46207
chrXIII	564365	564491	127	1.27712
chrXIII	570275	570678	404	1.28705
chrXIII	577658	578090	433	1.55212
chrXIII	578207	579704	1498	2.78759
chrXIII	596235	597323	1089	1.35199
chrXIII	597991	598155	165	1.24954
chrXIII	605681	606129	449	3.1872
chrXIII	608462	608805	344	1.43484
chrXIII	621726	622123	398	1.43112
chrXIII	622347	622868	522	1.73949
chrXIII	623157	623271	115	1.23524
chrXIII	623399	623647	249	1.18397
chrXIII	634646	635381	736	1.34565
chrXIII	635806	636295	490	1.24166
chrXIII	640020	641764	1745	3.21898
chrXIII	662643	662784	142	1.22672
chrXIII	665435	667456	2022	1.72619
chrXIII	667516	668220	705	1.39837
chrXIII	668433	670777	2345	2.48386
chrXIII	674374	675996	1623	1.95229
chrXIII	676730	677456	727	1.89641
chrXIII	677519	677631	113	1.36204
chrXIII	689226	689335	110	1.31112
chrXIII	689404	689707	304	1.29526
chrXIII	690024	691245	1222	1.75809
chrXIII	696718	697149	432	1.42632
chrXIII	697357	697471	115	1.19731
chrXIII	697878	699461	1584	2.76425
chrXIII	700278	703491	3214	2.77601
chrXIII	703654	703846	193	1.23347
chrXIII	712257	712553	297	1.36619
chrXIII	712625	712840	216	1.21968
chrXIII	713280	717100	3821	1.97877
chrXIII	717673	718963	1291	1.9848
chrXIII	725452	726018	567	1.29775
chrXIII	738831	738958	128	1.23111
chrXIII	739800	740068	269	1.31448
chrXIII	740931	741921	991	1.39571
chrXIII	748030	750352	2323	1.83716
chrXIII	750514	752683	2170	5.43186
chrXIII	752750	753294	545	1.63332
chrXIII	759619	760001	383	1.42246
chrXIII	761527	762747	1221	2.08649
chrXIII	762867	763268	402	1.18466
chrXIII	763842	764094	253	1.20543
chrXIII	774104	774608	505	1.9478
chrXIII	774736	775988	1253	1.88414
chrXIII	778295	779662	1368	1.77029
chrXIII	784447	784579	133	1.21877
chrXIII	788698	788927	230	1.24167
chrXIII	789797	789930	134	1.39154
chrXIII	796454	796777	324	1.21093

chrXIII	797129	797300	172	1.29976
chrXIII	797414	799660	2247	3.09333
chrXIII	801359	802979	1621	1.81605
chrXIII	803049	803616	568	1.61717
chrXIII	814998	815765	768	1.20124
chrXIII	825648	827392	1745	2.02829
chrXIII	839435	840444	1010	2.06314
chrXIII	840508	840762	255	1.26762
chrXIII	841150	841380	231	1.29331
chrXIII	841653	842646	994	1.21917
chrXIII	849652	850959	1308	1.3626
chrXIII	851043	851437	395	1.41842
chrXIII	851956	853409	1454	2.00698
chrXIII	853992	854408	417	1.31194
chrXIII	854535	855997	1463	2.05913
chrXIII	856948	857056	109	1.30418
chrXIII	857608	859146	1539	1.51255
chrXIII	859209	859326	118	1.4444
chrXIII	860336	862352	2017	7.75106
chrXIII	863545	865346	1802	1.89513
chrXIII	866827	867201	375	1.22365
chrXIII	872507	873104	598	12.97886
chrXIII	874222	874339	118	1.2415
chrXIII	886989	887921	933	1.37753
chrXIII	887975	889937	1963	3.59594
chrXIII	901326	901661	336	2.84646
chrXIII	902454	902695	242	1.38687
chrXIII	903712	904155	444	1.33187
chrXIII	904315	904441	127	1.25885
chrXIII	905904	906090	187	1.26688
chrXIII	906204	906375	172	1.16493
chrXIII	906852	906986	135	1.25317
chrXIII	914892	915190	299	1.5961
chrXIV	17428	17924	497	1.3186
chrXIV	27045	27188	144	1.26762
chrXIV	28319	28428	110	1.253
chrXIV	37802	39585	1784	1.67141
chrXIV	39812	40683	872	1.52677
chrXIV	40757	41077	321	1.40222
chrXIV	41650	43631	1982	2.04707
chrXIV	44801	45717	917	1.32989
chrXIV	48920	50089	1170	2.0089
chrXIV	53638	54767	1130	1.69953
chrXIV	56374	56570	197	1.22543
chrXIV	61565	61707	143	1.31382
chrXIV	70574	72639	2066	3.78057
chrXIV	76056	76616	561	1.53079
chrXIV	78268	78610	343	1.23146
chrXIV	79983	81155	1173	1.34373
chrXIV	82218	83045	828	1.50125
chrXIV	83648	84320	673	1.5348
chrXIV	87356	87472	117	1.45606
chrXIV	94643	95692	1050	2.04296

chrXIV	95951	96167	217	1.26292
chrXIV	96301	96488	188	1.55137
chrXIV	110152	110950	799	1.44999
chrXIV	116367	117713	1347	1.5231
chrXIV	120887	123692	2806	2.21582
chrXIV	124155	124365	211	1.28168
chrXIV	125114	127295	2182	3.22429
chrXIV	143678	144039	362	1.50065
chrXIV	144244	144826	583	1.56895
chrXIV	154716	155764	1049	1.76784
chrXIV	155841	158514	2674	2.20779
chrXIV	164578	164817	240	1.24572
chrXIV	166412	167680	1269	1.81806
chrXIV	169376	169640	265	1.21962
chrXIV	171036	171255	220	1.29678
chrXIV	174248	174385	138	1.20329
chrXIV	178535	180372	1838	1.7904
chrXIV	180437	180860	424	1.22682
chrXIV	180930	181430	501	1.5569
chrXIV	181499	182148	650	1.34998
chrXIV	184980	185719	740	1.43706
chrXIV	186317	186569	253	1.20534
chrXIV	186632	187790	1159	1.84533
chrXIV	188001	188424	424	1.51471
chrXIV	191192	191586	395	1.31098
chrXIV	195113	196779	1667	2.58948
chrXIV	196894	198948	2055	1.55371
chrXIV	199242	199454	213	1.24021
chrXIV	205024	205566	543	1.52979
chrXIV	209154	209380	227	1.29574
chrXIV	210143	211065	923	1.23451
chrXIV	214516	214631	116	1.3105
chrXIV	214719	216420	1702	2.40447
chrXIV	216672	217122	451	1.47684
chrXIV	217245	217552	308	1.62844
chrXIV	230163	230279	117	1.19991
chrXIV	230454	230628	175	1.36671
chrXIV	233014	233565	552	1.46449
chrXIV	233622	233791	170	1.26239
chrXIV	233851	234795	945	2.04557
chrXIV	234903	235042	140	1.20432
chrXIV	235433	236009	577	1.21941
chrXIV	254038	254321	284	1.21649
chrXIV	254723	254853	131	1.24351
chrXIV	255390	255684	295	1.36242
chrXIV	255744	257964	2221	2.39461
chrXIV	260628	261126	499	1.40824
chrXIV	262283	262649	367	1.29574
chrXIV	262889	263466	578	1.48773
chrXIV	263684	263929	246	1.19608
chrXIV	271001	271175	175	1.21165
chrXIV	273110	273688	579	1.47936
chrXIV	283145	283416	272	1.26965

chrXIV	285963	286314	352	1.38815
chrXIV	291166	293527	2362	2.74626
chrXIV	299318	299595	278	1.3348
chrXIV	299686	299924	239	1.21568
chrXIV	309590	310986	1397	2.76309
chrXIV	315203	317283	2081	2.82863
chrXIV	317352	317604	253	1.26504
chrXIV	334674	335646	973	1.34326
chrXIV	342933	344046	1114	2.04568
chrXIV	347953	348075	123	1.29516
chrXIV	352696	352863	168	1.18644
chrXIV	355131	355458	328	1.29293
chrXIV	357133	357399	267	1.33753
chrXIV	357468	357578	111	1.28224
chrXIV	358805	358984	180	1.26819
chrXIV	359056	361207	2152	1.79861
chrXIV	365066	365767	702	1.4077
chrXIV	365847	366391	545	1.40143
chrXIV	366859	367135	277	1.31583
chrXIV	367997	368799	803	1.52476
chrXIV	373469	373588	120	1.25907
chrXIV	373726	374049	324	1.36254
chrXIV	379050	380070	1021	1.71512
chrXIV	381045	381367	323	1.34687
chrXIV	389149	390763	1615	3.09468
chrXIV	391151	391429	279	1.26159
chrXIV	391531	392791	1261	1.94746
chrXIV	392849	393161	313	1.26992
chrXIV	393236	393417	182	1.24552
chrXIV	394612	394763	152	1.20534
chrXIV	397197	398929	1733	2.01803
chrXIV	401841	402542	702	1.46052
chrXIV	405682	406116	435	3.69355
chrXIV	406243	406363	121	1.28225
chrXIV	409370	410504	1135	1.8502
chrXIV	412894	413119	226	1.29059
chrXIV	413984	414640	657	1.31382
chrXIV	414740	414976	237	1.35822
chrXIV	415159	415265	107	1.25391
chrXIV	415368	416163	796	1.55414
chrXIV	416339	417345	1007	1.38128
chrXIV	420433	421130	698	1.4584
chrXIV	423920	424083	164	1.21539
chrXIV	426474	427870	1397	1.70746
chrXIV	434334	434640	307	1.4383
chrXIV	434702	435541	840	1.18597
chrXIV	435637	436102	466	1.2857
chrXIV	436457	436875	419	1.25733
chrXIV	436936	438984	2049	2.02911
chrXIV	440363	440826	464	1.5509
chrXIV	443185	443441	257	1.27082
chrXIV	463115	463355	241	1.2415
chrXIV	466894	467286	393	1.38414

chrXIV	469085	469645	561	1.83815
chrXIV	469774	470708	935	2.68791
chrXIV	475232	476672	1441	1.84571
chrXIV	476747	477093	347	1.25933
chrXIV	485885	487141	1257	1.8281
chrXIV	492667	494343	1677	2.2118
chrXIV	494430	495043	614	1.44038
chrXIV	503561	503737	177	1.26829
chrXIV	503913	504019	107	1.30491
chrXIV	504998	506024	1027	2.1435
chrXIV	506918	507151	234	1.48283
chrXIV	511691	511811	121	1.2857
chrXIV	511871	512360	490	1.48458
chrXIV	518420	518567	148	1.25998
chrXIV	531681	532729	1049	1.54868
chrXIV	532814	534503	1690	1.77404
chrXIV	534562	535464	903	2.11538
chrXIV	538303	538563	261	1.44644
chrXIV	538936	540473	1538	1.88636
chrXIV	540726	541504	779	1.41829
chrXIV	542222	542850	629	1.42632
chrXIV	543089	543364	276	1.51873
chrXIV	549758	550130	373	1.33711
chrXIV	556752	558085	1334	1.88284
chrXIV	558911	559032	122	1.23744
chrXIV	560202	560676	475	1.28167
chrXIV	560783	561576	794	1.68043
chrXIV	568352	568492	141	1.45043
chrXIV	575451	575748	298	1.45368
chrXIV	576297	576424	128	1.19667
chrXIV	581322	581438	117	1.18933
chrXIV	582920	584394	1475	2.05992
chrXIV	584472	584685	214	1.28291
chrXIV	585542	585666	125	1.34782
chrXIV	585868	586106	239	1.26254
chrXIV	586646	586856	211	1.19187
chrXIV	586971	587294	324	1.25379
chrXIV	587591	587969	379	1.53114
chrXIV	588049	588493	445	1.415
chrXIV	591592	591808	217	1.31785
chrXIV	604332	604822	491	1.49061
chrXIV	604966	605218	253	1.40981
chrXIV	605274	605621	348	1.39271
chrXIV	605867	607117	1251	1.41873
chrXIV	611324	612567	1244	2.29403
chrXIV	615135	615378	244	1.23542
chrXIV	615477	616809	1333	1.69304
chrXIV	617731	617907	177	1.22416
chrXIV	628038	629742	1705	4.3434
chrXIV	638726	638877	152	1.25348
chrXIV	643067	645142	2076	2.05707
chrXIV	645466	645810	345	1.20936
chrXIV	646407	646808	402	1.18791

chrXIV	647162	649271	2110	2.20578
chrXIV	651231	651447	217	1.32712
chrXIV	651502	651742	241	1.32869
chrXIV	652138	652669	532	1.30796
chrXIV	652756	652932	177	1.20534
chrXIV	653895	655180	1286	1.70734
chrXIV	663697	664319	623	9.49892
chrXIV	666328	666456	129	1.19688
chrXIV	672121	673656	1536	1.78011
chrXIV	674641	674750	110	1.26561
chrXIV	674850	675151	302	1.28771
chrXIV	675206	675318	113	1.27565
chrXIV	676112	676710	599	1.52476
chrXIV	676769	677917	1149	1.63726
chrXIV	678010	679336	1327	1.7844
chrXIV	679695	681328	1634	3.02742
chrXIV	690026	690744	719	1.47253
chrXIV	691343	693009	1667	1.81458
chrXIV	696592	697980	1389	2.01292
chrXIV	698551	698947	397	1.3319
chrXIV	699114	699277	164	1.24753
chrXIV	699869	700090	222	1.22945
chrXIV	710844	710952	109	1.23143
chrXIV	711009	712277	1269	1.84819
chrXIV	712374	716675	4302	1.94996
chrXIV	718667	718894	228	1.27565
chrXIV	722315	723438	1124	1.41446
chrXIV	729944	730107	164	1.20534
chrXIV	730176	731493	1318	1.87029
chrXIV	738797	740476	1680	1.25804
chrXIV	749557	750634	1078	2.9569
chrXIV	753933	754199	267	1.22141
chrXIV	754422	754670	249	1.52275
chrXIV	754845	755020	176	1.64881
chrXIV	755593	755999	407	1.23548
chrXIV	758377	759069	693	1.2887
chrXIV	759682	759849	168	1.31699
chrXIV	764360	764533	174	1.22342
chrXIV	770460	770664	205	1.27496
chrXIV	770910	772193	1284	1.29238
chrXV	35563	35673	111	1.23317
chrXV	35858	36110	253	1.29764
chrXV	36184	36390	207	1.54363
chrXV	45246	45645	400	1.36003
chrXV	59638	60610	973	1.71962
chrXV	60670	60779	110	1.33793
chrXV	77694	78665	972	1.62815
chrXV	80925	81182	258	1.37966
chrXV	81457	81686	230	1.24716
chrXV	81765	82015	251	1.32584
chrXV	82081	82534	454	1.50271
chrXV	83104	83485	382	2.62408
chrXV	87388	87929	542	1.53008

chrXV	89086	90398	1313	2.06114
chrXV	91243	91521	279	1.24351
chrXV	96227	96426	200	1.25793
chrXV	96589	96709	121	1.24941
chrXV	103377	104070	694	1.228
chrXV	106453	106580	128	1.3151
chrXV	108270	108442	173	1.2508
chrXV	110255	110699	445	1.6483
chrXV	112722	112936	215	1.25424
chrXV	113004	113306	303	1.38543
chrXV	113598	113762	165	1.31399
chrXV	127240	128338	1099	1.48719
chrXV	128520	128764	245	1.31353
chrXV	129228	129623	396	1.35638
chrXV	131779	133916	2138	3.19373
chrXV	135314	137130	1817	2.00087
chrXV	137193	138078	886	1.57096
chrXV	138149	138263	115	1.34998
chrXV	138989	139112	124	1.29093
chrXV	149226	151604	2379	3.3609
chrXV	154264	154443	180	1.2099
chrXV	154662	155054	393	1.3864
chrXV	180309	180603	295	1.42024
chrXV	180660	180782	123	1.2085
chrXV	185400	186627	1228	1.524
chrXV	187182	187933	752	1.29249
chrXV	188465	188621	157	1.22141
chrXV	188701	191432	2732	4.09817
chrXV	192669	193967	1299	2.06515
chrXV	194125	194830	706	1.41226
chrXV	194897	195080	184	1.49262
chrXV	195459	195701	243	1.31784
chrXV	195783	197074	1292	2.23792
chrXV	197238	197565	328	1.60913
chrXV	197652	197758	107	1.27766
chrXV	197990	200812	2823	2.09377
chrXV	204278	204467	190	1.23949
chrXV	205626	205761	136	1.29775
chrXV	206762	206868	107	1.34965
chrXV	213298	214100	803	1.79395
chrXV	215913	217504	1592	2.00764
chrXV	218640	219568	929	1.26553
chrXV	222589	222695	107	1.19949
chrXV	222918	223433	516	1.25035
chrXV	223488	223629	142	1.27021
chrXV	223726	224906	1181	1.74172
chrXV	231443	231985	543	1.54822
chrXV	238709	238822	114	1.2876
chrXV	239213	241629	2417	1.85285
chrXV	242035	243751	1717	1.78499
chrXV	246497	247662	1166	1.52052
chrXV	247756	249144	1389	1.69014
chrXV	250795	251660	866	1.45577

chrXV	264796	268921	4126	2.21638
chrXV	269012	269290	279	1.28935
chrXV	269484	270172	689	1.91028
chrXV	279413	282147	2735	2.98716
chrXV	282257	282612	356	1.3212
chrXV	284530	286650	2121	2.71832
chrXV	287763	288147	385	1.24947
chrXV	288334	288526	193	1.22692
chrXV	288621	288903	283	1.21727
chrXV	288969	289261	293	1.2369
chrXV	290382	290567	186	1.23559
chrXV	292092	292479	388	4.01775
chrXV	294028	295240	1213	1.66149
chrXV	298764	299216	453	1.30393
chrXV	299447	299662	216	1.38295
chrXV	299952	300403	452	1.31495
chrXV	300533	300778	246	1.38176
chrXV	305150	305256	107	1.26873
chrXV	307713	309707	1995	1.90704
chrXV	309924	310570	647	1.32069
chrXV	310633	311307	675	1.2829
chrXV	311905	312047	143	1.30645
chrXV	312534	312918	385	1.34163
chrXV	321180	322506	1327	2.26719
chrXV	325798	327242	1445	2.57197
chrXV	332982	334916	1935	1.46975
chrXV	340670	340875	206	1.25159
chrXV	372115	373057	943	1.7178
chrXV	391898	392381	484	1.53561
chrXV	412726	412955	230	1.38546
chrXV	413822	414921	1100	1.82063
chrXV	416450	416561	112	1.2288
chrXV	418313	418707	395	1.24214
chrXV	424194	424765	572	2.78589
chrXV	432834	433403	570	1.30653
chrXV	433656	433852	197	1.23347
chrXV	448700	448831	132	1.22945
chrXV	451192	452379	1188	2.14752
chrXV	455140	456536	1397	2.41269
chrXV	461287	461565	279	1.27365
chrXV	461619	462170	552	1.63579
chrXV	466199	466783	585	1.49404
chrXV	468652	469906	1255	2.02661
chrXV	473465	473780	316	1.21604
chrXV	476223	477297	1075	1.55489
chrXV	480146	480859	714	1.75779
chrXV	480998	481423	426	1.25294
chrXV	481494	481746	253	1.27216
chrXV	481985	484872	2888	2.54347
chrXV	485226	485497	272	1.36806
chrXV	485625	487435	1811	2.32841
chrXV	487515	488522	1008	1.57097
chrXV	489410	489688	279	1.21539

chrXV	489851	490389	539	1.43637
chrXV	493672	498359	4688	2.66424
chrXV	503764	503877	114	1.30981
chrXV	503934	505134	1201	1.56523
chrXV	505409	506418	1010	1.56519
chrXV	506692	506798	107	1.31671
chrXV	506862	507101	240	1.39216
chrXV	507165	507861	697	1.23839
chrXV	511336	511449	114	1.38061
chrXV	513115	513441	327	1.35245
chrXV	531562	531677	116	1.28996
chrXV	537175	540184	3010	2.55373
chrXV	546394	546567	174	1.30981
chrXV	548194	549752	1559	2.02179
chrXV	550842	550957	116	1.26556
chrXV	551169	551362	194	1.2191
chrXV	551527	552923	1397	1.68077
chrXV	563659	564841	1183	2.32631
chrXV	571087	571290	204	1.22743
chrXV	579778	580249	472	1.44705
chrXV	581148	582601	1454	3.03144
chrXV	582665	582793	129	1.32186
chrXV	583572	584043	472	1.7164
chrXV	586028	588091	2064	1.56712
chrXV	588870	589119	250	1.20936
chrXV	589208	590719	1512	2.67586
chrXV	600812	601314	503	1.40222
chrXV	601448	601605	158	1.22348
chrXV	602327	602689	363	1.24752
chrXV	606107	606536	430	3.84773
chrXV	607066	607282	217	1.23863
chrXV	607879	609242	1364	1.74396
chrXV	609790	610026	237	1.25356
chrXV	612139	612966	828	1.46047
chrXV	619210	619734	525	2.30393
chrXV	619842	620084	243	1.21682
chrXV	624221	624761	541	1.80601
chrXV	624849	625216	368	1.29741
chrXV	625320	625546	227	1.38403
chrXV	625621	627214	1594	2.12808
chrXV	631144	632529	1386	4.24334
chrXV	632600	632706	107	1.42297
chrXV	632802	633492	691	1.46851
chrXV	633578	633791	214	1.20095
chrXV	635983	637203	1221	2.031
chrXV	638130	638353	224	1.25557
chrXV	638478	639280	803	1.29466
chrXV	646986	647534	549	1.19229
chrXV	649751	649863	113	1.29976
chrXV	651045	652367	1323	1.88435
chrXV	656776	657434	659	1.32309
chrXV	658246	660511	2266	2.00752
chrXV	660773	662941	2169	1.93166

chrXV	663073	663802	730	1.47253
chrXV	665384	665786	403	1.21539
chrXV	670206	670666	461	1.67912
chrXV	670984	671446	463	2.17873
chrXV	677977	678139	163	1.25155
chrXV	678404	678523	120	1.26963
chrXV	680788	681007	220	1.3078
chrXV	681134	681298	165	1.24694
chrXV	697036	698876	1841	2.34335
chrXV	700214	701738	1525	2.83055
chrXV	701895	702153	259	1.22697
chrXV	710864	710985	122	1.28168
chrXV	715003	716074	1072	1.74976
chrXV	717988	718783	796	1.65132
chrXV	725135	726899	1765	1.50466
chrXV	727631	730389	2759	1.95642
chrXV	734075	734489	415	1.27565
chrXV	734567	734968	402	1.28771
chrXV	735274	739224	3951	3.06358
chrXV	745962	746417	456	1.33404
chrXV	746488	746596	109	1.22779
chrXV	746720	747227	508	1.28981
chrXV	751062	752525	1464	2.25511
chrXV	756079	756326	248	1.26963
chrXV	759316	759498	183	1.2857
chrXV	761459	762139	681	1.69825
chrXV	766232	768659	2428	1.92252
chrXV	768713	771384	2672	2.15358
chrXV	775197	775344	148	1.24753
chrXV	775397	775700	304	1.23172
chrXV	777671	779156	1486	2.91932
chrXV	779524	779688	165	1.19448
chrXV	788323	789365	1043	1.66818
chrXV	789766	790132	367	1.39281
chrXV	793306	794648	1343	2.00557
chrXV	796800	797345	546	3.15129
chrXV	798220	798800	581	1.51672
chrXV	798936	799108	173	1.20073
chrXV	803633	803745	113	1.23524
chrXV	803816	804229	414	1.32548
chrXV	804305	804978	674	1.77788
chrXV	815292	816144	853	1.50504
chrXV	821524	822859	1336	2.43462
chrXV	824128	824370	243	1.22025
chrXV	824479	824586	108	1.33445
chrXV	824938	826179	1242	1.58111
chrXV	826265	826385	121	1.26871
chrXV	826442	828015	1574	1.73603
chrXV	831867	832067	201	1.27049
chrXV	832342	832532	191	1.38361
chrXV	833655	833797	143	1.20534
chrXV	833900	834475	576	1.49239
chrXV	836279	836477	199	1.34396

chrXV	837171	837943	773	1.3209
chrXV	838702	839002	301	1.26476
chrXV	842607	842719	113	1.23308
chrXV	849411	849525	115	1.14321
chrXV	854652	855506	855	1.79988
chrXV	860676	860792	117	1.19787
chrXV	865639	865757	119	1.23749
chrXV	873891	874663	773	1.42081
chrXV	876043	876594	552	1.27977
chrXV	876857	877020	164	1.33274
chrXV	877081	877716	636	1.255
chrXV	883059	883168	110	1.23749
chrXV	887442	889064	1623	1.26019
chrXV	889120	890688	1569	1.83614
chrXV	891111	892617	1507	1.47896
chrXV	892815	893067	253	1.39386
chrXV	905485	905628	144	1.24883
chrXV	906196	906331	136	1.25545
chrXV	908560	908677	118	1.19648
chrXV	915231	915615	385	1.36029
chrXV	917972	918793	822	2.37653
chrXV	925071	925636	566	11.30818
chrXV	930531	931158	628	1.44819
chrXV	931572	932795	1224	1.69581
chrXV	935631	937705	2075	3.2981
chrXV	939208	939493	286	1.26201
chrXV	939601	939836	236	1.20776
chrXV	942985	944643	1659	2.36379
chrXV	944720	945121	402	1.37811
chrXV	945378	945576	199	1.24969
chrXV	945709	946608	900	1.43235
chrXV	956890	957089	200	1.22803
chrXV	957664	957922	259	1.30854
chrXV	958413	958634	222	1.39346
chrXV	958834	959094	261	1.23749
chrXV	959966	960100	135	1.28536
chrXV	960429	961308	880	1.75844
chrXV	965315	967112	1798	3.07717
chrXV	967192	968242	1051	1.661
chrXV	968516	968683	168	1.23534
chrXV	977940	978237	298	1.48659
chrXV	980814	980972	159	1.24552
chrXV	982810	987174	4365	2.42475
chrXV	987388	988470	1083	1.91298
chrXV	988833	989069	237	1.30019
chrXV	989299	989614	316	1.34247
chrXV	989750	990685	936	1.39896
chrXV	991257	991384	128	1.24314
chrXV	991466	991605	140	1.34408
chrXV	991667	991910	244	1.21056
chrXV	992264	992486	223	1.37486
chrXV	993241	993369	129	1.19165
chrXV	995097	995806	710	1.42105

chrXV	1004579	1005012	434	1.38213
chrXV	1006445	1007019	575	1.33217
chrXV	1007133	1007409	277	1.39063
chrXV	1009229	1009407	179	1.41429
chrXV	1010602	1010833	232	1.28972
chrXV	1011544	1014072	2529	3.34483
chrXV	1014144	1014367	224	1.31784
chrXV	1027726	1028769	1044	1.37347
chrXV	1039182	1039423	242	1.29626
chrXV	1039529	1041820	2292	1.5328
chrXV	1041891	1042128	238	1.37688
chrXV	1043410	1043518	109	1.2424
chrXV	1047785	1048020	236	1.37983
chrXV	1048087	1048923	837	1.60743
chrXV	1048982	1049459	478	1.51143
chrXV	1052733	1053472	740	1.31918
chrXV	1053792	1054031	240	1.18191
chrXV	1057810	1057929	120	1.46047
chrXV	1058127	1058391	265	1.40865
chrXV	1058461	1058607	147	1.22444
chrXV	1059252	1059414	163	1.29825
chrXV	1059784	1060192	409	1.67944
chrXV	1060277	1060613	337	1.34691
chrXVI	22926	23099	174	1.32143
chrXVI	37879	38206	328	1.3669
chrXVI	39572	40936	1365	1.54048
chrXVI	41036	41425	390	1.43929
chrXVI	41563	41720	158	1.25803
chrXVI	42679	43003	325	1.28287
chrXVI	43127	43620	494	1.35748
chrXVI	43677	43826	150	1.21291
chrXVI	44116	44458	343	1.32704
chrXVI	45554	47697	2144	2.01127
chrXVI	50375	52238	1864	2.65175
chrXVI	52323	52674	352	1.3023
chrXVI	52851	53306	456	1.39351
chrXVI	55770	55907	138	1.37438
chrXVI	70529	72205	1677	1.85348
chrXVI	72425	75353	2929	1.73676
chrXVI	82125	82326	202	1.2798
chrXVI	89376	91260	1885	3.56781
chrXVI	115312	115526	215	1.22389
chrXVI	119713	121128	1416	1.90846
chrXVI	121406	121579	174	1.34803
chrXVI	122043	122299	257	1.37007
chrXVI	122401	122636	236	1.22744
chrXVI	124691	126451	1761	1.71315
chrXVI	132513	132904	392	1.46659
chrXVI	132979	133097	119	1.32935
chrXVI	133212	133463	252	1.34841
chrXVI	137784	140538	2755	1.6496
chrXVI	140654	140798	145	1.20132
chrXVI	147847	149018	1172	1.81393

chrXVI	149854	150682	829	2.62631
chrXVI	150919	151092	174	1.2556
chrXVI	152447	152998	552	1.40021
chrXVI	153613	153885	273	1.30981
chrXVI	162092	162271	180	1.38012
chrXVI	167045	169533	2489	2.79855
chrXVI	170024	170368	345	1.21284
chrXVI	170469	170761	293	1.23311
chrXVI	170858	171181	324	1.28412
chrXVI	171907	172177	271	1.26762
chrXVI	172280	172427	148	1.31643
chrXVI	172508	172795	288	1.36167
chrXVI	173116	173922	807	1.39872
chrXVI	181035	183547	2513	1.78079
chrXVI	184497	184704	208	1.24941
chrXVI	184854	184960	107	1.27099
chrXVI	191371	191478	108	1.22012
chrXVI	191565	191684	120	1.33391
chrXVI	192520	193759	1240	1.5495
chrXVI	194202	194613	412	1.32989
chrXVI	195205	195497	293	1.31124
chrXVI	198211	198460	250	1.25573
chrXVI	209664	210171	508	1.43235
chrXVI	210318	211027	710	1.64128
chrXVI	211123	211479	357	1.32335
chrXVI	212110	212789	680	1.50869
chrXVI	212887	214941	2055	4.14191
chrXVI	215380	216197	818	1.50378
chrXVI	218593	218723	131	1.24562
chrXVI	218957	219155	199	1.21994
chrXVI	219406	220910	1505	1.67911
chrXVI	223831	224025	195	1.29574
chrXVI	226810	227821	1012	1.34481
chrXVI	228058	229221	1164	1.64331
chrXVI	231940	233403	1464	1.76984
chrXVI	238663	239362	700	1.45345
chrXVI	249826	251279	1454	3.5429
chrXVI	254897	255058	162	1.23289
chrXVI	255156	255628	473	1.41167
chrXVI	255692	255848	157	1.29976
chrXVI	255934	256898	965	1.29208
chrXVI	260835	260959	125	1.21584
chrXVI	273665	273969	305	1.33478
chrXVI	274179	274287	109	1.25833
chrXVI	275122	275251	130	1.22389
chrXVI	275335	276909	1575	2.29145
chrXVI	277311	277577	267	1.3181
chrXVI	279283	280915	1633	1.70978
chrXVI	282432	282637	206	1.27417
chrXVI	282911	283774	864	2.30622
chrXVI	297151	297331	181	1.32485
chrXVI	305750	306722	973	1.68607
chrXVI	308039	309098	1060	5.19863

chrXVI	309855	310019	165	1.26775
chrXVI	315948	318444	2497	1.72529
chrXVI	318616	319006	391	1.29134
chrXVI	320541	320659	119	1.24988
chrXVI	321094	321472	379	1.33433
chrXVI	323030	324300	1271	1.99886
chrXVI	328934	329593	660	3.41519
chrXVI	331154	332547	1394	2.58144
chrXVI	339532	341682	2151	1.81008
chrXVI	341799	341986	188	1.23949
chrXVI	349252	350132	881	1.50266
chrXVI	352131	352515	385	1.4394
chrXVI	352576	352707	132	1.26561
chrXVI	357573	357741	169	1.26159
chrXVI	357885	358658	774	1.43637
chrXVI	358877	359177	301	1.85824
chrXVI	359302	359497	196	1.42833
chrXVI	359672	360048	377	1.61315
chrXVI	360208	360363	156	1.23193
chrXVI	370937	371362	426	1.25067
chrXVI	371736	374364	2629	2.15578
chrXVI	374654	374861	208	1.28459
chrXVI	376640	377264	625	1.85422
chrXVI	381279	381430	152	1.41959
chrXVI	381542	381761	220	1.32965
chrXVI	382651	382763	113	1.21593
chrXVI	383112	384353	1242	1.61109
chrXVI	384444	385053	610	1.36806
chrXVI	395798	397848	2051	2.783
chrXVI	397915	398620	706	1.61315
chrXVI	422466	423170	705	1.53008
chrXVI	423819	423959	141	1.27363
chrXVI	425955	426361	407	1.17823
chrXVI	426951	427235	285	1.20814
chrXVI	427750	427977	228	1.24692
chrXVI	428107	428739	633	1.43896
chrXVI	431831	432701	871	1.75103
chrXVI	432770	433051	282	1.41393
chrXVI	436032	436266	235	1.26561
chrXVI	436627	436854	228	1.43436
chrXVI	443159	443410	252	1.53882
chrXVI	444501	446382	1882	2.15145
chrXVI	450177	450440	264	1.2857
chrXVI	450542	450757	216	1.21214
chrXVI	452605	452965	361	1.20102
chrXVI	463561	464854	1294	1.84511
chrXVI	469860	469972	113	1.22941
chrXVI	471751	473587	1837	2.35042
chrXVI	473735	474640	906	1.40422
chrXVI	474695	475774	1080	1.97081
chrXVI	475869	476031	163	1.21229
chrXVI	476105	476256	152	1.21941
chrXVI	485956	486529	574	1.48888

chrXVI	486717	486823	107	1.22402
chrXVI	486883	489057	2175	1.33085
chrXVI	490322	490520	199	1.31583
chrXVI	490591	492088	1498	1.72375
chrXVI	498012	498525	514	1.44495
chrXVI	498805	499172	368	1.31562
chrXVI	499408	500547	1140	1.45037
chrXVI	500627	500888	262	1.58612
chrXVI	501669	501915	247	1.25821
chrXVI	502010	502241	232	1.45432
chrXVI	503091	503274	184	1.1889
chrXVI	503445	503551	107	1.21186
chrXVI	503965	504077	113	1.30652
chrXVI	509501	510773	1273	2.23253
chrXVI	511485	512461	977	2.00087
chrXVI	516623	517139	517	1.24036
chrXVI	517448	517570	123	1.27017
chrXVI	517635	517756	122	1.21456
chrXVI	523597	523935	339	1.23249
chrXVI	524371	525765	1395	2.31567
chrXVI	525857	526837	981	1.70237
chrXVI	527618	528131	514	1.3351
chrXVI	528511	528895	385	1.34352
chrXVI	529605	530014	410	1.35679
chrXVI	531972	534574	2603	2.52482
chrXVI	534627	534752	126	1.28641
chrXVI	534840	536034	1195	1.19741
chrXVI	538556	538775	220	1.1996
chrXVI	539329	539732	404	1.28796
chrXVI	540118	540357	240	1.32387
chrXVI	540538	540761	224	1.26561
chrXVI	540859	541176	318	1.3078
chrXVI	541237	542584	1348	1.58503
chrXVI	543331	543440	110	1.20936
chrXVI	549297	551225	1929	2.26913
chrXVI	551482	551661	180	1.35675
chrXVI	555375	556564	1190	3.3167
chrXVI	561045	561613	569	1.40061
chrXVI	577016	578080	1065	1.56955
chrXVI	578262	578562	301	1.31633
chrXVI	599764	601277	1514	2.63769
chrXVI	601402	601813	412	1.26378
chrXVI	607482	608256	775	1.46875
chrXVI	608816	609036	221	1.2572
chrXVI	613050	613624	575	1.42172
chrXVI	614866	615017	152	1.23095
chrXVI	618493	619865	1373	1.93264
chrXVI	619992	620553	562	1.25739
chrXVI	622853	623155	303	1.37129
chrXVI	623224	624778	1555	1.51796
chrXVI	627572	627705	134	1.41951
chrXVI	633764	633998	235	1.28168
chrXVI	636659	637978	1320	1.93658

chrXVI	638034	638537	504	1.22595
chrXVI	638797	638921	125	1.29309
chrXVI	645417	646712	1296	1.69892
chrXVI	648739	649018	280	1.31577
chrXVI	649102	650471	1370	3.71647
chrXVI	650603	651033	431	1.37208
chrXVI	651195	651490	296	1.22543
chrXVI	651580	651736	157	1.27164
chrXVI	651800	652080	281	1.36204
chrXVI	653027	653724	698	1.77788
chrXVI	653840	654667	828	1.33044
chrXVI	654911	655620	710	1.354
chrXVI	656143	656412	270	1.56494
chrXVI	657096	657304	209	1.38316
chrXVI	657588	657760	173	1.18323
chrXVI	657997	658124	128	1.32797
chrXVI	658338	658506	169	1.24351
chrXVI	659988	660102	115	1.23347
chrXVI	660429	661474	1046	2.09127
chrXVI	673381	673489	109	1.2803
chrXVI	673659	673924	266	1.1859
chrXVI	674004	674235	232	1.21279
chrXVI	674349	674783	435	1.45113
chrXVI	674899	676274	1376	1.57595
chrXVI	678850	679386	537	2.28037
chrXVI	680741	680945	205	1.25663
chrXVI	682836	683081	246	1.27113
chrXVI	683166	683401	236	1.29425
chrXVI	685107	685746	640	1.237
chrXVI	686218	686659	442	1.39438
chrXVI	687044	687153	110	1.21969
chrXVI	687244	687610	367	1.26337
chrXVI	687783	689468	1686	1.81818
chrXVI	691323	693091	1769	2.15008
chrXVI	693147	696231	3085	2.39929
chrXVI	702033	702855	823	1.68145
chrXVI	704815	705094	280	1.37148
chrXVI	708008	708945	938	1.51471
chrXVI	709003	709239	237	1.34597
chrXVI	710410	711660	1251	1.4665
chrXVI	712611	713935	1325	1.42219
chrXVI	715054	716811	1758	2.83858
chrXVI	718821	719360	540	1.72081
chrXVI	720582	720712	131	1.30769
chrXVI	728317	729323	1007	1.72766
chrXVI	732915	733831	917	2.21381
chrXVI	743910	744148	239	1.28168
chrXVI	745436	745621	186	1.20333
chrXVI	745711	745885	175	1.31985
chrXVI	748748	750112	1365	1.66337
chrXVI	750209	750983	775	1.36832
chrXVI	751224	751913	690	1.37791
chrXVI	752766	752886	121	1.21263

chrXVI	753259	753490	232	1.26221
chrXVI	753709	754120	412	1.24954
chrXVI	758647	758856	210	1.2015
chrXVI	766862	768464	1603	2.13517
chrXVI	768710	769200	491	1.79797
chrXVI	771002	771523	522	1.46896
chrXVI	773079	773535	457	1.75779
chrXVI	773794	774122	329	1.61114
chrXVI	775161	775429	269	1.23491
chrXVI	776020	776845	826	1.4318
chrXVI	780186	780955	770	1.4203
chrXVI	781142	781263	122	1.29976
chrXVI	784588	786458	1871	2.41796
chrXVI	786647	786800	154	1.21943
chrXVI	789505	790616	1112	2.0033
chrXVI	791036	791252	217	1.2857
chrXVI	792346	792469	124	1.23923
chrXVI	794004	794192	189	1.28455
chrXVI	795817	796129	313	1.31382
chrXVI	803111	803486	376	1.32387
chrXVI	803544	804530	987	2.66983
chrXVI	810563	810681	119	1.71059
chrXVI	812061	813448	1388	2.00575
chrXVI	815073	816155	1083	1.58904
chrXVI	822693	822821	129	1.26
chrXVI	824151	824328	178	1.29146
chrXVI	824413	825082	670	1.4874
chrXVI	829763	830301	539	1.52656
chrXVI	830387	830613	227	1.18658
chrXVI	830842	831499	658	1.41659
chrXVI	835031	835183	153	1.41741
chrXVI	835237	836451	1215	1.96873
chrXVI	837391	837690	300	1.27966
chrXVI	839612	840388	777	1.4627
chrXVI	841178	841293	116	1.19881
chrXVI	841604	841728	125	1.19949
chrXVI	860521	861838	1318	1.65453
chrXVI	863195	863420	226	1.23361
chrXVI	863481	864729	1249	2.29906
chrXVI	876276	876484	209	1.65146
chrXVI	876560	876745	186	1.24234
chrXVI	876826	877759	934	1.46237
chrXVI	878809	878920	112	1.2857
chrXVI	885502	887360	1859	1.83579
chrXVI	887486	889639	2154	1.81404
chrXVI	890275	891293	1019	1.43838
chrXVI	891700	892095	396	1.31784
chrXVI	893383	894555	1173	1.74793
chrXVI	901456	902217	762	4.62977
chrXVI	902301	902683	383	1.23146
chrXVI	909154	910884	1731	1.82707
chrXVI	911205	912117	913	1.5622
chrXVI	916224	917876	1653	2.89043

chrXVI	917986	918399	414	1.31382
chrXVI	920365	920556	192	1.36774
chrXVI	921003	922433	1431	1.60373
chrXVI	922497	923610	1114	1.57059
chrXVI	923847	924794	948	2.07033
chrXVI	925196	925868	673	1.50181
chrXVI	925946	926845	900	1.53613
chrXVI	927906	928303	398	1.40085
