# Supplementary material for: Exo1 protects DNA nicks from ligation to promote crossover formation during meiosis
Source: PLoS Biol. 2023 Apr 20;21(4):e3002085. doi: 10.1371/journal.pbio.3002085 (PMC10153752; doi:10.1371/journal.pbio.3002085)
Supplement: S1 Data — (PDF) [file pbio.3002085.s015.pdf]

S1 Data. Underlying data for Fig 2

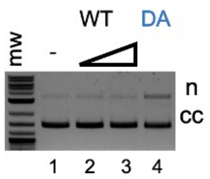

Figure 2A
full gel image

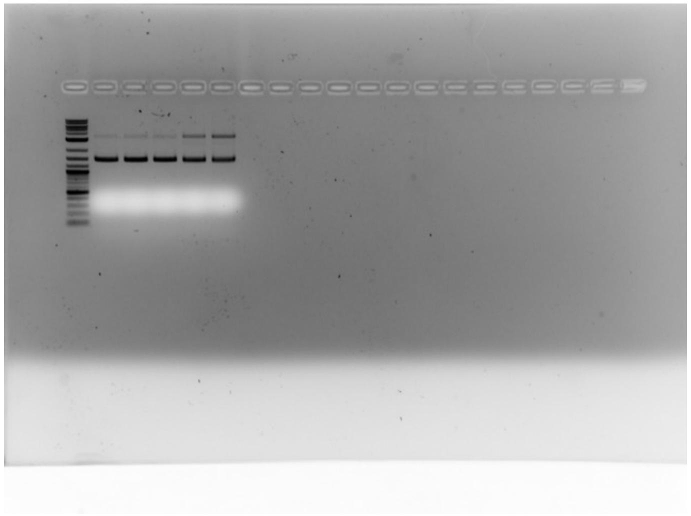

Figure 2A
full gel image labeled

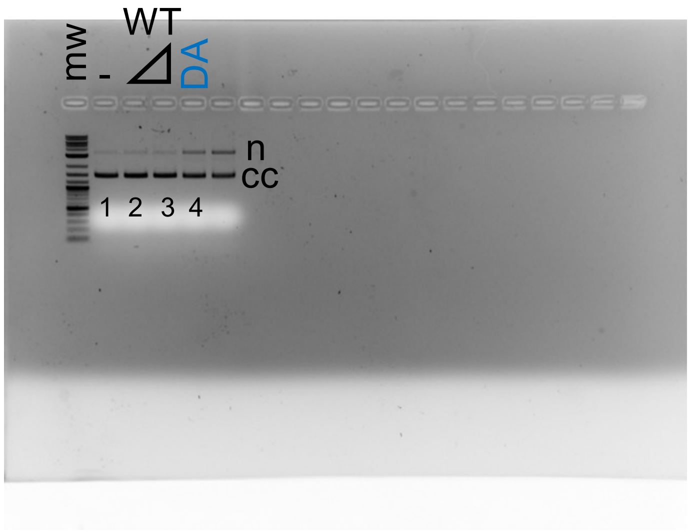

S1 Data for Fig 2A

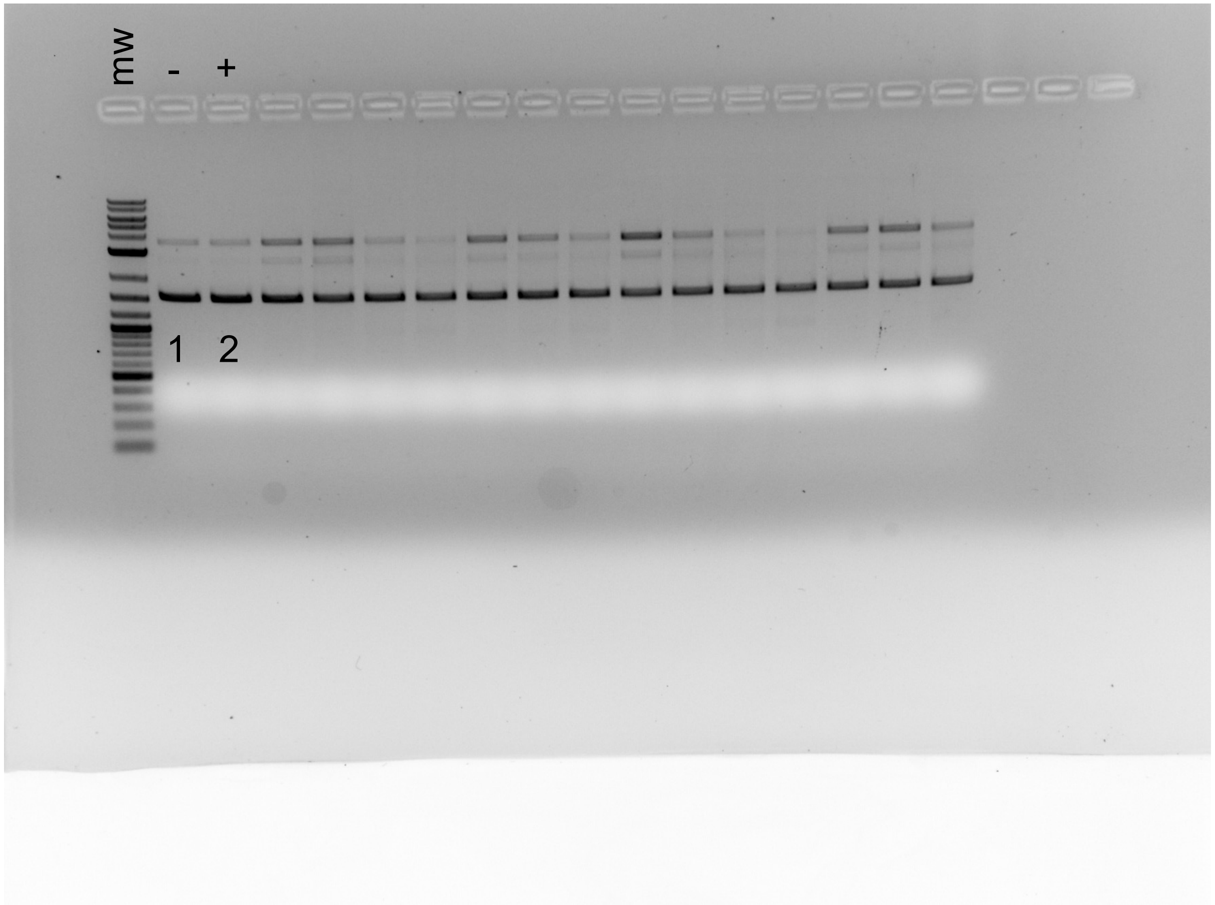

Wild-type Exo1 does not nick closed circular DNA

3.6 nM closed circular substrate with 2 mM ATP and 5 mM $MgCl_2$. Where + (lane 2), wild-type Exo1 is 10 nM.

S1 Data, replicate result for Fig 2A

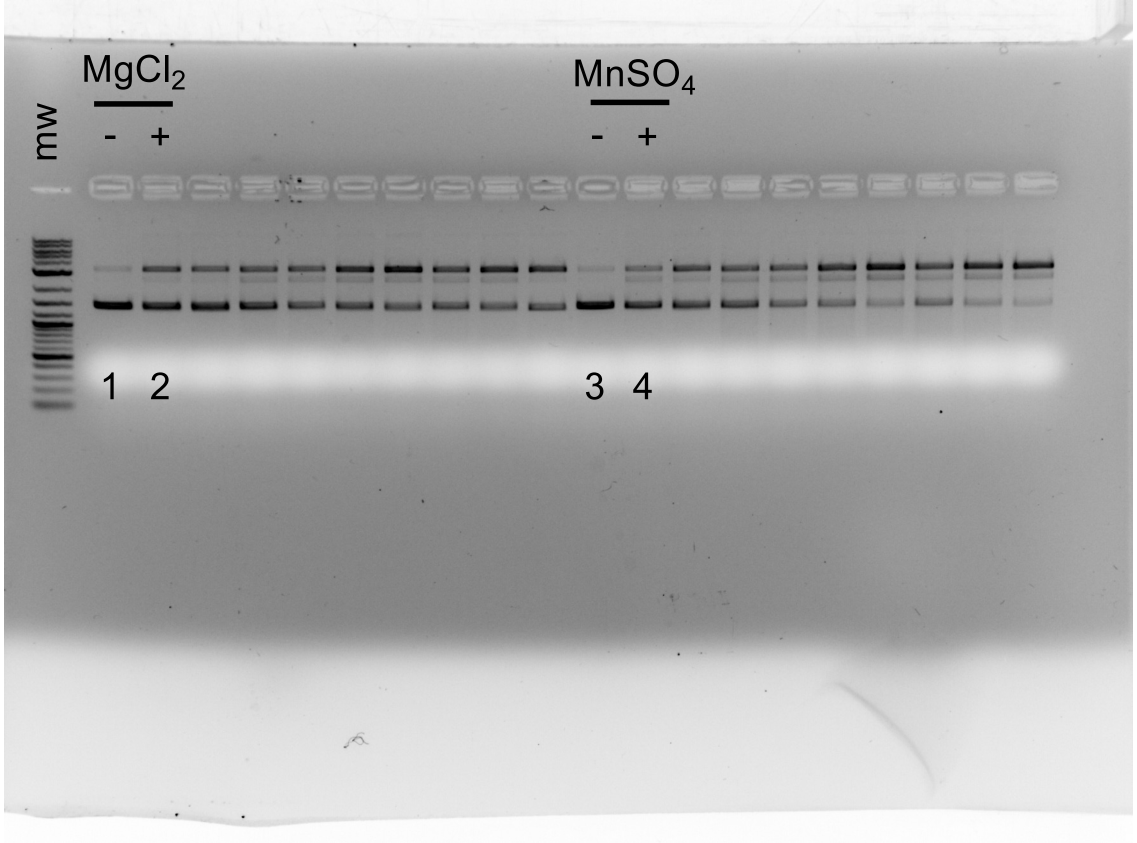

exo1-D173A nicks closed circular DNA

3.6 nM closed circular substrate with 5 mM $MgCl_2$ or $MnSO_4$ where indicated (no ATP). Where + (lanes 2 and 4), exo1-D173A is 20 nM.

S1 Data, replicate result for Fig 2A

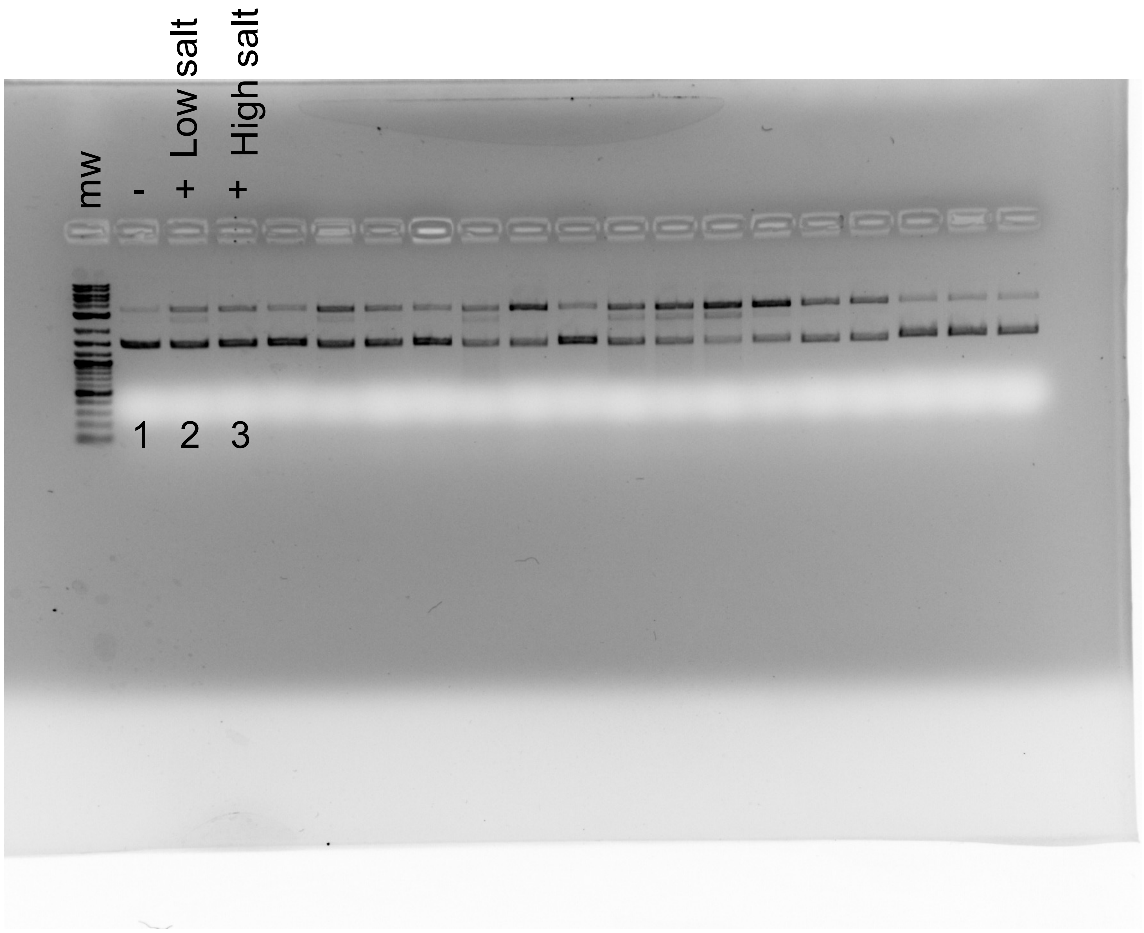

exo1-D173A nicks closed circular DNA

3.6 nM closed circular substrate with 5 mM $MnSO_4$ (no ATP). Where + (lane 2-3), exo1-D173A is 20 nM. Low salt condition is 20 mM KCl, 0 mM NaCl. High salt is 20 mM KCl, 100 mM NaCl.

Note that $MnSO_4$ was used here

S1 Data, replicate result for Fig 2A

	10/23/18	10/23/18	10/15/18	10/15/18		
Concentration (nM)	D173A	D173A	D173A	D173A	AVERAGE	STD DEV
0	0.00	0.00	0.00	0.00	0.00	0.00
5	0.02	0.07	0.02	0.05	0.04	0.02
10	0.06	0.05	0.05	0.08	0.06	0.02
20	0.10	0.09	0.08	0.11	0.09	0.01
	10/23/18	10/23/18	10/15/18	10/15/18		
Concentration (nM)	G236D	G236D	G236D	G236D	AVERAGE	STD DEV
0	0.00	0.00	0.00	0.00	0.00	0.00
5	0.02	0.00	0.00	0.02	0.01	0.01
10	0.04	0.01	0.03	0.03	0.03	0.01
20	0.08	0.03	0.05	0.06	0.06	0.02
	10/23/18	10/23/18	10/15/18	10/15/18		
Concentration (nM)	D173AG236D	D173AG236D	D173AG236D	D173AG236D	AVERAGE	STD DEV
0	0.00	0.00	0.00	0.00	0	0
5	0.03	0.03	0.00	0.00	0.02	0.02
10	0.06	0.04	0.00	0.00	0.02	0.03
20	0.06	0.06	0.01	0.04	0.04	0.02

Assay conditions
20 mM Hepes pH 7.5
20 mM KCl
1% glycerol
5 mM $MgCl_2$
3.6 nM pUC18
0.2 mg/ml BSA

Fraction of nicked DNA at each concentration with average +/- standard deviation shown

S1 Data, endonuclease data for Fig 2B

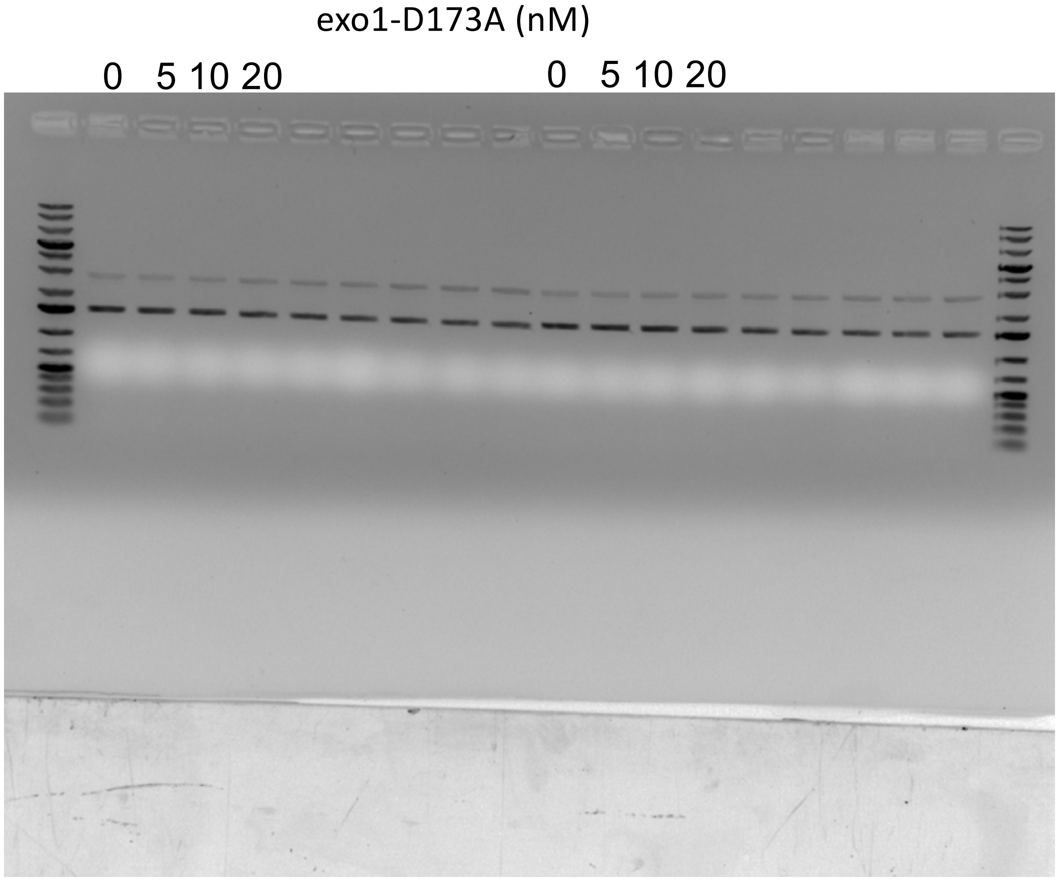

S1 Data, Fig 2B source file, 10/15/18, exo1-D173A

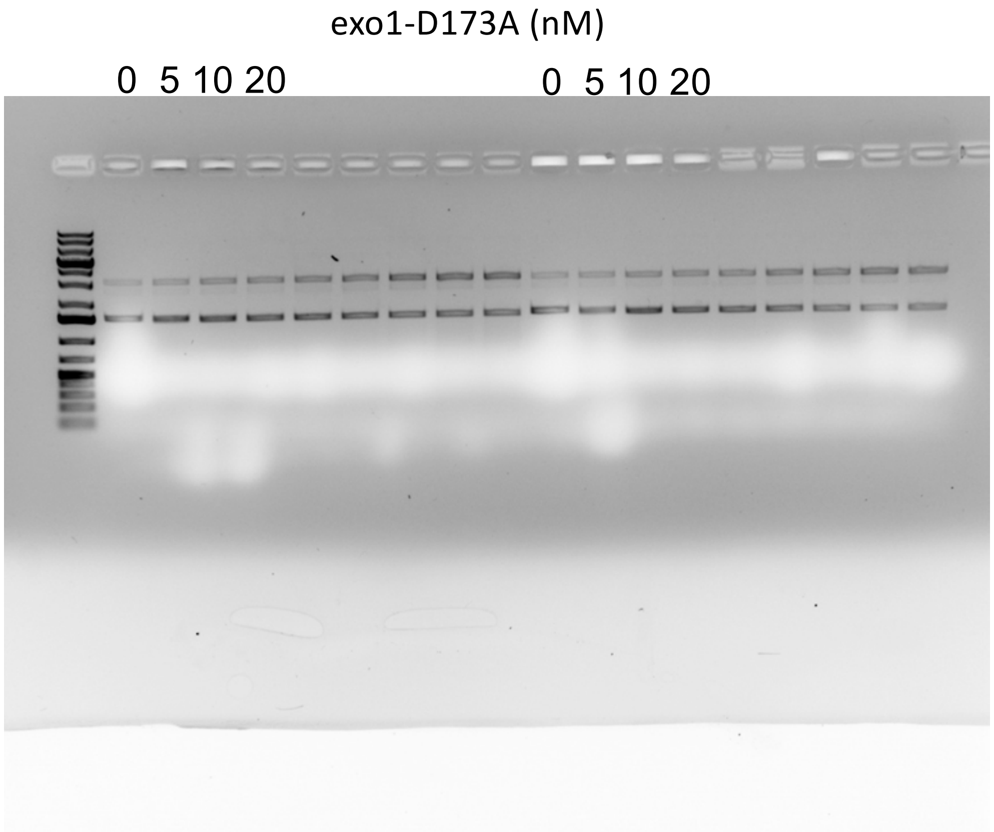

S1 Data, Fig 2B source file, 10/23/18, exo1-D173A

exo1-G236D (nM)

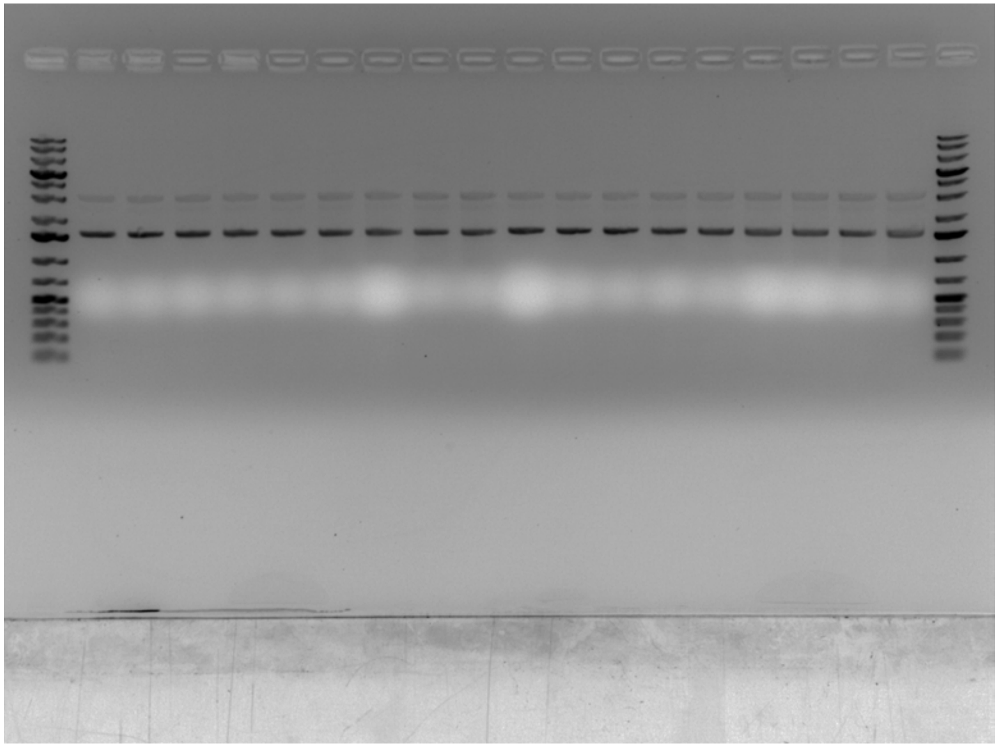

S1 Data, Fig 2B source file, 10/15/18, exo1-G236D

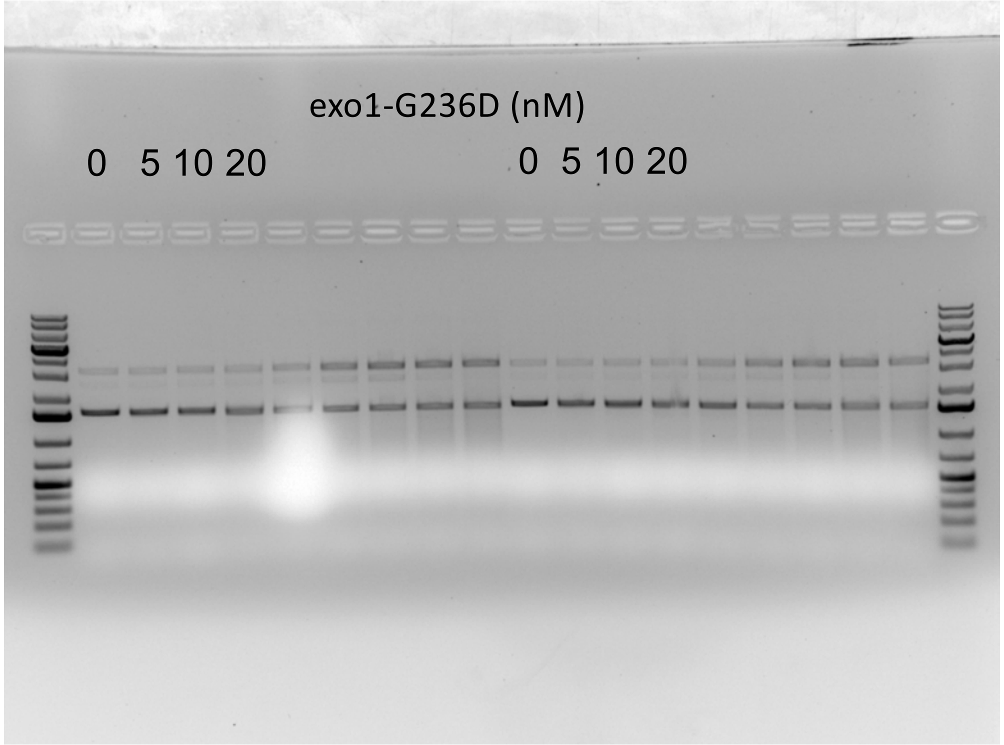

S1 Data, Fig 2B source file, 10/23/18, exo1-G236D

exo1-D173A,G236D (nM)

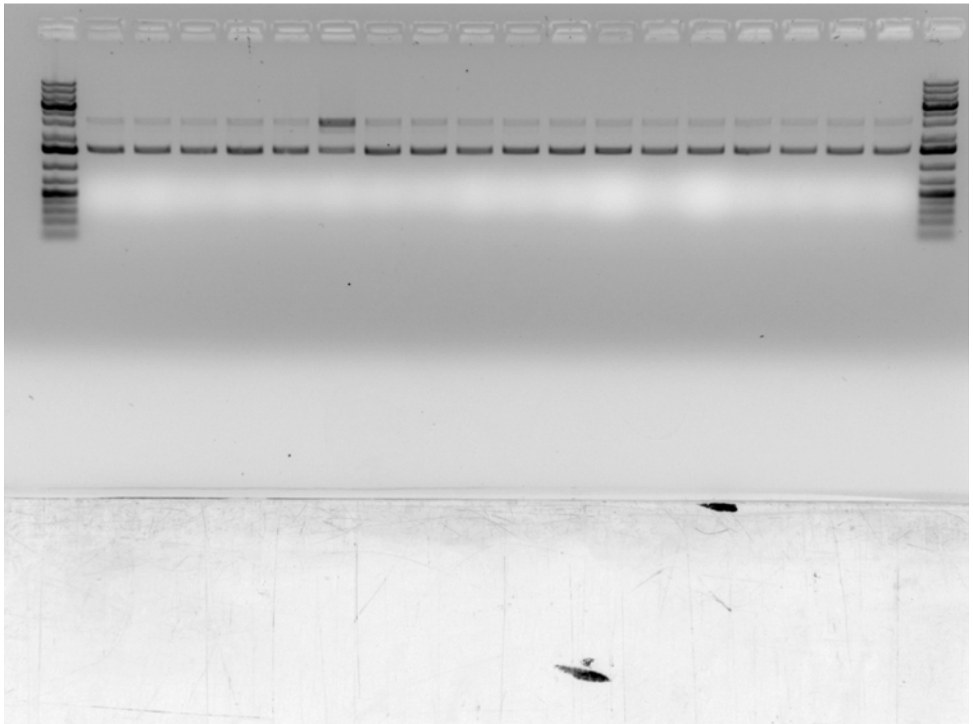

S1 Data, Fig 2B source file, 10/15/18, exo1-D173A,G236D

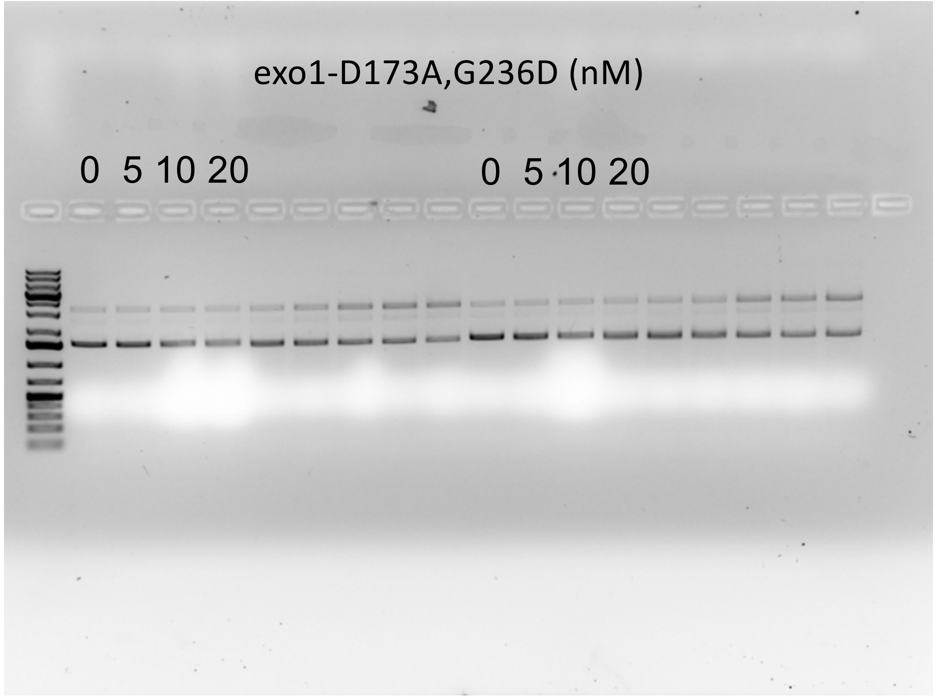

S1 Data, Fig 2B source file, 10/23/18, exo1-D173A,G236D

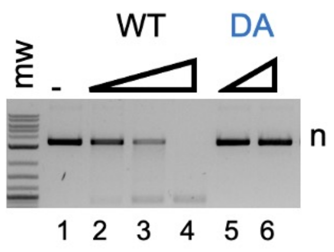

Figure 2C
full gel image

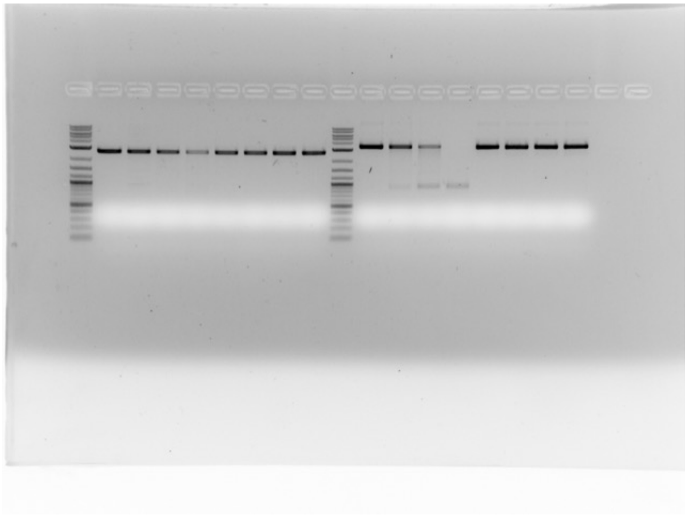

Figure 2C
full gel image labeled

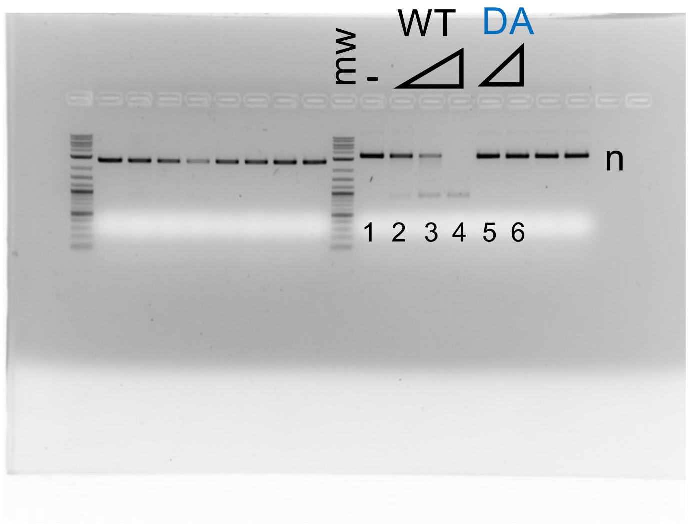

S1 Data for Fig 2C

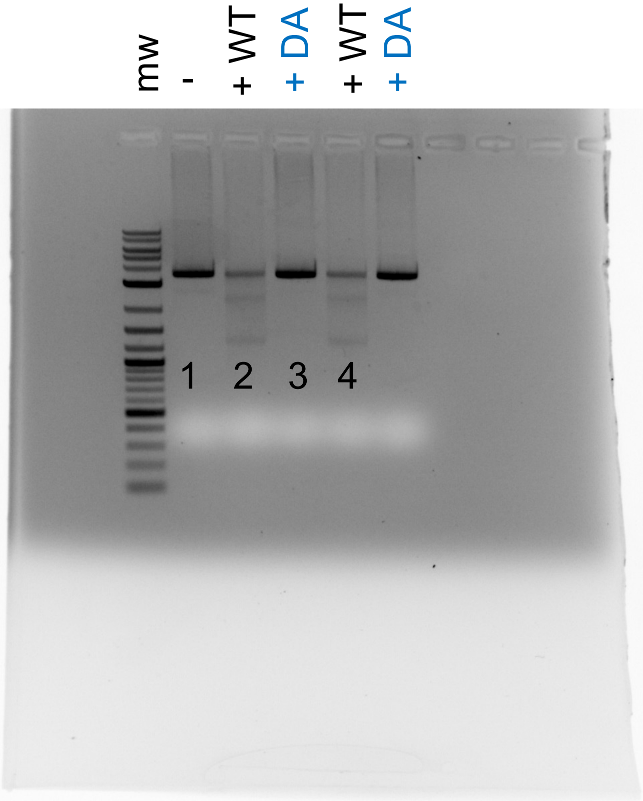

exo1-D173A does not show exonuclease activity

3.6 nM nicked circular substrate (4 nicks) 5 mM $MgCl_2$ (no ATP). Wild-type Exo1 and exo1-D173A are both 10 nM. Lanes 3-4 are duplicate reactions of lanes 2-3.

S1 Data, replicate result for Fig 2C

		% total binding						% total binding				
		1/14/23	1/15/23	1/16/23	Mean	Std Dev		1/14/23	1/15/23	1/16/23	Mean	Std Dev
		D173A	D173A	D173A	D173A	D173A		D173AG236D	D173AG236D	D173AG236D	D173AG236D	D173AG236D
Substrate	nM protein											
homoduplex	0	0	0	0	0	0		0	0	0	0	0
homoduplex	5	0.74	0.7	0.74	0.73	0.019		0	0.25	0.43	0.23	0.031
homoduplex	12.5	0.62	2	1.6	1.4	0.58		0	0.36	0.31	0.22	0.16
homoduplex	25	1.4	2.2	1.6	1.7	0.34		0.19	0.45	1.5	0.71	0.57
homoduplex	50	2	3	2.9	2.6	0.45		0.13	0.49	1	0.54	0.36
flap	0	0	0	0	0	0		0	0	0	0	0
flap	5	1.1	0.16	1.1	0.79	0.44		0.3	0.21	0	0.17	0.13
flap	12.5	3.2	3.2	2.8	3.1	0.19		0.55	0.7	0	0.42	0.3
flap	25	5.4	6.4	6.4	6.1	0.47		0.71	0.33	0	0.35	0.29
flap	50	8.7	9	7.5	8.4	0.65		0.62	0.76	0.59	0.66	0.074

Assay Conditions (30°C 10 minutes)
15 nM homoduplex or 5’ flap substrate (^{32}P-labeled)
35 mM NaCl
20 mM Tris 7.5
0.04 mg/ml BSA
0.01 mM EDTA
0.1 mM DTT

S1 Data, DNA binding assays for Fig 2D
