# Supplementary material for: Exo1 protects DNA nicks from ligation to promote crossover formation during meiosis
Source: PLoS Biol. 2023 Apr 20;21(4):e3002085. doi: 10.1371/journal.pbio.3002085 (PMC10153752; doi:10.1371/journal.pbio.3002085)
Supplement: S3 Data — Chemiluminescence (α-myc signal) and colorimetric (molecular weight standard) data are presented, as well as a composite image. (PPTX) [file pbio.3002085.s017.pptx]

## Slide 1
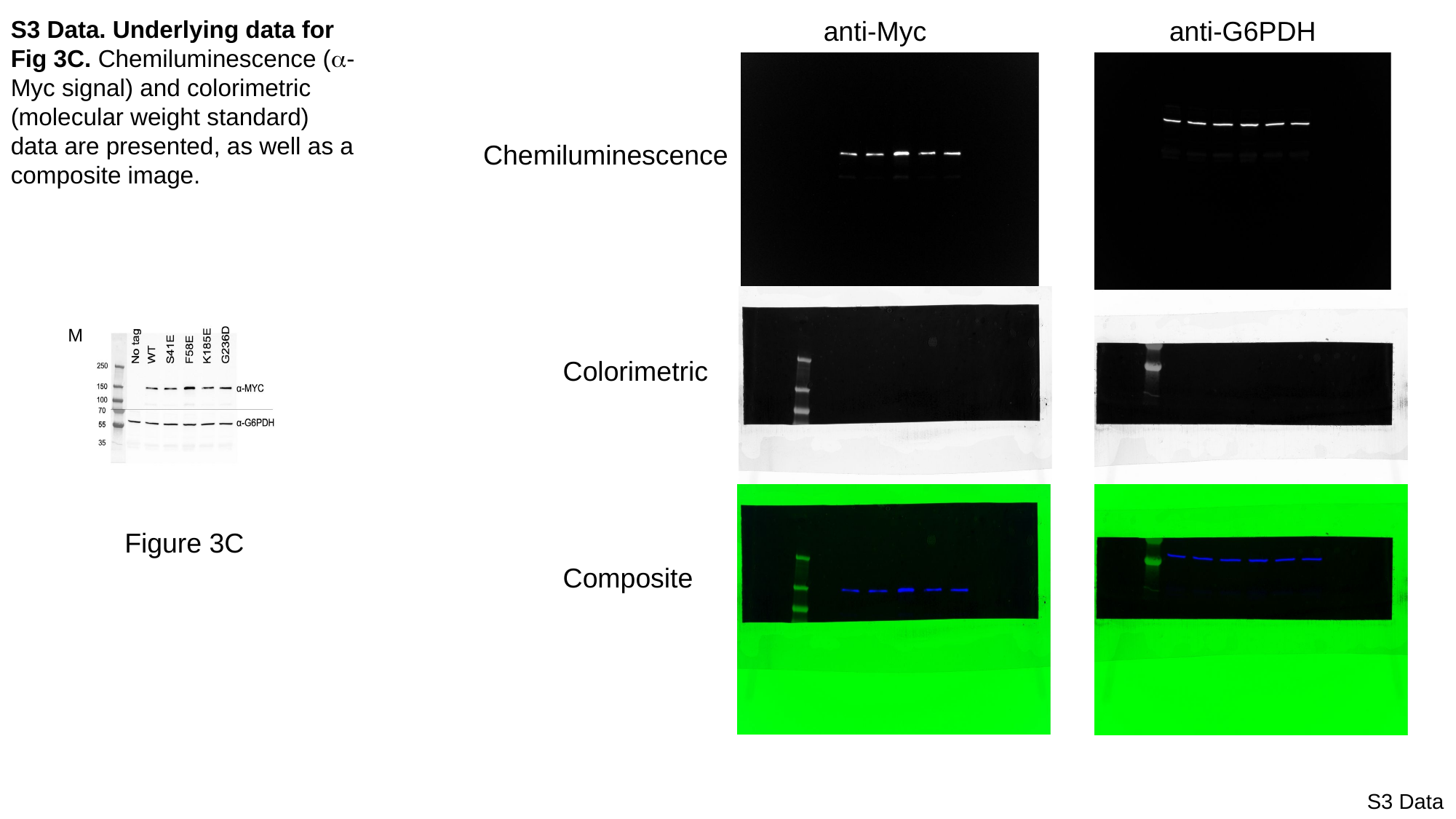

S3 Data. Underlying data for Fig 3C. Chemiluminescence (a-Myc signal) and colorimetric (molecular weight standard) data are presented, as well as a composite image.
anti-Myc
anti-G6PDH
Chemiluminescence
M
Colorimetric
Figure 3C
Composite
S3 Data
