# Supplementary material for: Exo1 protects DNA nicks from ligation to promote crossover formation during meiosis
Source: PLoS Biol. 2023 Apr 20;21(4):e3002085. doi: 10.1371/journal.pbio.3002085 (PMC10153752; doi:10.1371/journal.pbio.3002085)
Supplement: S7 Data — (PDF) [file pbio.3002085.s021.pdf]

S7 Data. Underlying data for S3 Fig. Entire YPD and YPD-MMS plates

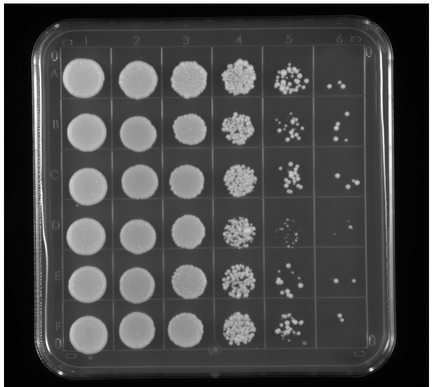

exo1-D78A, D173A
wild-type
exo1-D78A
exo1-D173A
exo1-D171A
exo1Δ

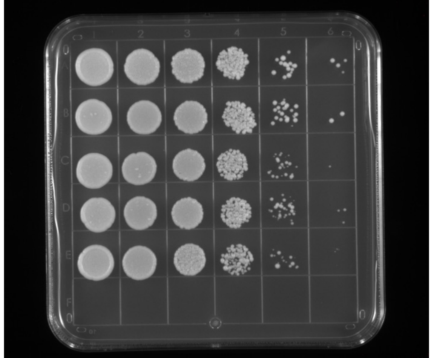

exo1-D78A, D173A
exo1-D173A, D171A
exo1-G236D
exo1-MIP
exo1Δ mus81Δ
empty

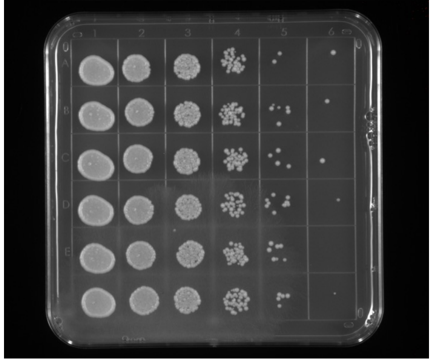

exo1Δ
wild-type
exo1Δ
exo1-R92A
exo1-K85E
exo1-K121A

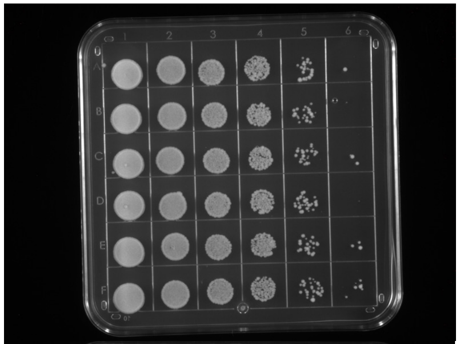

wild-type
exo1-S41E
exo1-F58E
exo1Δ mus81Δ
exo1Δ mus81Δ
mus81Δ

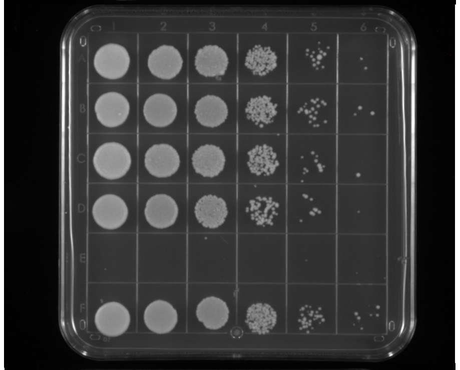

exo1-K185E
exo1-G236D
exo1-MIP
exo1Δ mus81Δ
Empty lane
mus81Δ

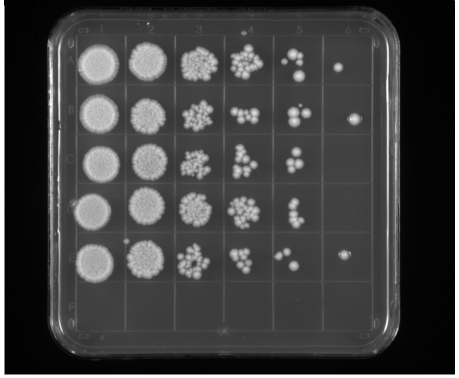

wild-type
exo1Δ
exo1Δ + pEXO1-RAD27
exo1Δ +pEXO1
exo1Δ + empty vector
empty

Full YPD Plates

Full MMS Plates

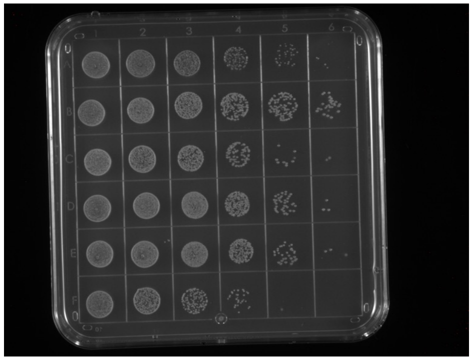

exo1-D78A, D173A
wild-type
exo1-D78A
exo1-D173A
exo1-D171A
exo1Δ

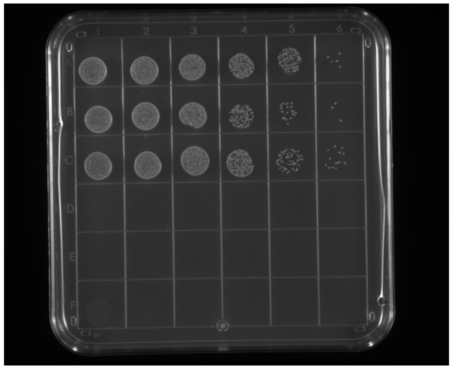

exo1-K185E
exo1-S41E
exo1-F58E
exo1Δ mus81Δ
exo1Δ mus81Δ
mus81Δ

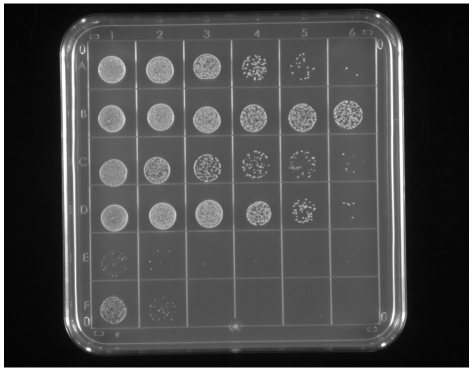

exo1-G236D
exo1-MIP
exo1-D78A, D173A
exo1-D173A, D171A
exo1Δ mus81Δ
mus81Δ

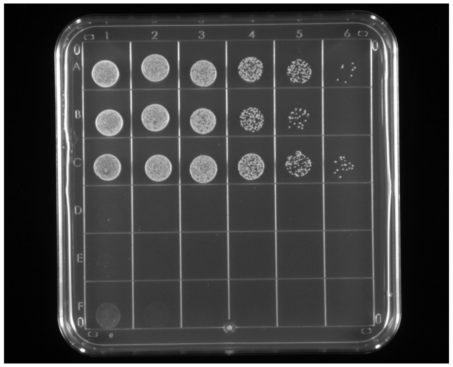

exo1-K185E
exo1-G236D
exo1-MIP
exo1Δ mus81Δ
exo1Δ mus81Δ
mus81Δ

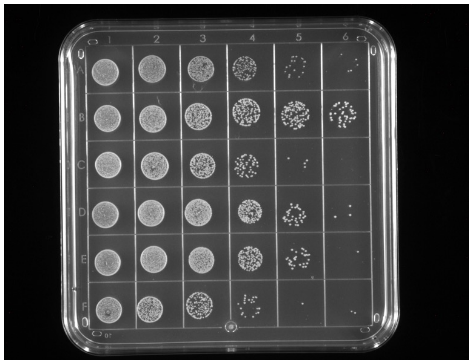

exo1Δ
wild-type
exo1Δ
exo1-R92A
exo1-K85E
exo1-K121A

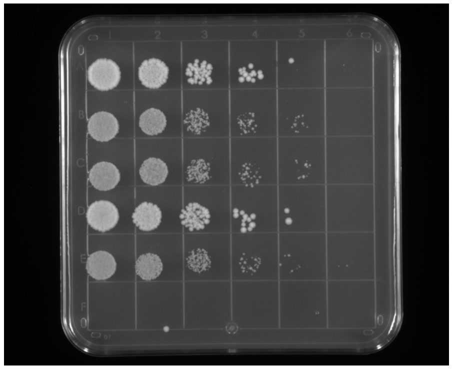

wild-type
exo1Δ
exo1Δ + pEXO1-RAD27
exo1Δ +pEXO1
exo1Δ + empty vector
empty
